# Multiple adverse outcomes associated with antipsychotic use in people with dementia: population based matched cohort study

**DOI:** 10.1136/bmj-2023-076268

**Published:** 2024-04-17

**Authors:** Pearl L H Mok, Matthew J Carr, Bruce Guthrie, Daniel R Morales, Aziz Sheikh, Rachel A Elliott, Elizabeth M Camacho, Tjeerd van Staa, Anthony J Avery, Darren M Ashcroft

**Affiliations:** 1 Centre for Pharmacoepidemiology and Drug Safety, Division of Pharmacy and Optometry, University of Manchester, Manchester, M13 9PT, UK; 2 Manchester Academic Health Science Centre, Manchester, UK; 3 NIHR Greater Manchester Patient Safety Research Collaboration, University of Manchester, Manchester, UK; 4 Advanced Care Research Centre, Usher Institute, College of Medicine and Veterinary Medicine, University of Edinburgh, Edinburgh, UK; 5 Population Health and Genomics, University of Dundee, Dundee, UK; 6 Usher Institute, College of Medicine and Veterinary Medicine, University of Edinburgh, Edinburgh, UK; 7 Nuffield Department of Primary Care Health Sciences, University of Oxford, Oxford, UK; 8 Manchester Centre for Health Economics, Division of Population Health, Manchester, UK; 9 Division of Informatics, Imaging and Data Sciences, University of Manchester, Manchester, UK; 10 Centre for Primary Care, School of Medicine, University of Nottingham, Nottingham, UK

## Abstract

**Objective:**

To investigate risks of multiple adverse outcomes associated with use of antipsychotics in people with dementia.

**Design:**

Population based matched cohort study.

**Setting:**

Linked primary care, hospital and mortality data from Clinical Practice Research Datalink (CPRD), England.

**Population:**

Adults (≥50 years) with a diagnosis of dementia between 1 January 1998 and 31 May 2018 (n=173 910, 63.0% women). Each new antipsychotic user (n=35 339, 62.5% women) was matched with up to 15 non-users using incidence density sampling.

**Main outcome measures:**

The main outcomes were stroke, venous thromboembolism, myocardial infarction, heart failure, ventricular arrhythmia, fracture, pneumonia, and acute kidney injury, stratified by periods of antipsychotic use, with absolute risks calculated using cumulative incidence in antipsychotic users versus matched comparators. An unrelated (negative control) outcome of appendicitis and cholecystitis combined was also investigated to detect potential unmeasured confounding.

**Results:**

Compared with non-use, any antipsychotic use was associated with increased risks of all outcomes, except ventricular arrhythmia. Current use (90 days after a prescription) was associated with elevated risks of pneumonia (hazard ratio 2.19, 95% confidence interval (CI) 2.10 to 2.28), acute kidney injury (1.72, 1.61 to 1.84), venous thromboembolism (1.62, 1.46 to 1.80), stroke (1.61, 1.52 to 1.71), fracture (1.43, 1.35 to 1.52), myocardial infarction (1.28, 1.15 to 1.42), and heart failure (1.27, 1.18 to 1.37). No increased risks were observed for the negative control outcome (appendicitis and cholecystitis). In the 90 days after drug initiation, the cumulative incidence of pneumonia among antipsychotic users was 4.48% (4.26% to 4.71%) versus 1.49% (1.45% to 1.53%) in the matched cohort of non-users (difference 2.99%, 95% CI 2.77% to 3.22%).

**Conclusions:**

Antipsychotic use compared with non-use in adults with dementia was associated with increased risks of stroke, venous thromboembolism, myocardial infarction, heart failure, fracture, pneumonia, and acute kidney injury, but not ventricular arrhythmia. The range of adverse outcomes was wider than previously highlighted in regulatory alerts, with the highest risks soon after initiation of treatment.

## Introduction

Dementia is a clinical syndrome characterised by progressive cognitive decline and functional disability, with estimates suggesting that by 2050 around 152.8 million people globally will be affected.^1^ Behavioural and psychological symptoms of dementia are common aspects of the disease and include features such as apathy, depression, aggression, anxiety, irritability, delirium, and psychosis. Such symptoms can negatively impact the quality of life of patients and their carers and are associated with early admission to care.^2^ ^3^ Antipsychotics are commonly prescribed for the management of behavioural and psychological symptoms of dementia, despite longstanding concerns about their safety.^4^ ^5^ ^6^ During the covid-19 pandemic, the proportion of people with dementia prescribed antipsychotics increased, possibly owing to worsened behavioural and psychological symptoms of dementia linked to lockdown measures or reduced availability of non-pharmaceutical treatment options.^7^ According to guidelines from the UK’s National Institute for Health and Care Excellence, antipsychotics should only be prescribed for the treatment of behavioural and psychological symptoms of dementia if non-drug interventions have been ineffective, if patients are at risk of harming themselves or others or are experiencing agitation, hallucinations, or delusions causing them severe distress.^8^ Antipsychotics should at most be prescribed at the lowest effective dose and for the shortest possible time. Only two antipsychotics, risperidone (an atypical, or second generation, antipsychotic) and haloperidol (a typical, or first generation, antipsychotic), are licensed in the UK for the treatment of behavioural and psychological symptoms of dementia,^9^ although others have been commonly prescribed off-label.^5^ ^10^

Based on evidence from clinical trials of risperidone, the US Food and Drug Administration (FDA) first issued a warning in 2003 about the increased risks of cerebrovascular adverse events (eg, stroke, transient ischaemic attack) associated with use of atypical antipsychotics in older adults with dementia.^11^ A meta-analysis of 17 trials among such patients subsequently found a 1.6-1.7-fold increased risk of mortality with atypical antipsychotics compared with placebo, which led the FDA to issue a “black box” warning in 2005 for all atypical antipsychotics.^11^ This warning was extended to typical antipsychotics in 2008, after two observational studies reported that the risk of death associated with their use among older people might be even greater than for atypical antipsychotics.^12^ ^13^ ^14^ The increased risks for stroke and mortality have been consistently reported by many observational studies and meta-analyses since,^11^ ^15^ ^16^ ^17^ ^18^ ^19^ ^20^ ^21^ and they have led to regulatory safety warnings and national interventions in the UK, US, and Europe, aiming to reduce inappropriate prescribing of these drugs for the treatment of behavioural and psychological symptoms of dementia.^8^ ^11^ ^22^ ^23^ ^24^ ^25^ ^26^ Other adverse outcomes have also been investigated in observational studies,^27^ ^28^ ^29^ although, with the exception of pneumonia,^14^ ^30^ ^31^ ^32^ the evidence is less conclusive or is more limited among people with dementia. For example, inconsistent or limited evidence has been found for risks of myocardial infarction,^33^ ^34^ ventricular arrhythmia,^35^ ^36^ venous thromboembolism,^37^ ^38^ ^39^ ^40^ fracture,^41^ ^42^ ^43^ and acute kidney injury.^44^ ^45^ ^46^ Most studies also reported only one outcome or type of outcomes. Examining multiple adverse events in a single cohort is needed to give a more comprehensive estimate of the total potential harm associated with use of antipsychotics in people with dementia.

Using linked primary and secondary care data in England, we investigated the risks of a range of adverse outcomes potentially associated with antipsychotic use in a large cohort of adults with dementia—namely, stroke, venous thromboembolism, myocardial infarction, heart failure, ventricular arrhythmia, fracture, pneumonia, and acute kidney injury. We report both relative and absolute risks.

## Methods

### Data sources

The study used anonymised electronic health records from Clinical Practice Research Datalink (CPRD). In the UK, residents are required to be registered with a primary care general practice to receive care from the NHS. The NHS is a publicly funded healthcare service, free at the point of use. More than 98% of the UK population are registered with a general practice, and their electronic health records are transferred when they change practice.^47^ ^48^ Community prescribing is most often done by the general practitioner, including antipsychotic treatment recommended by specialists. CPRD data are sourced from more than 2000 general practices covering around 20% of the UK population, and include information on diagnoses, primary healthcare contacts, prescribed drugs, laboratory test results, and referrals to secondary healthcare services.^47^ ^48^ CPRD contains two databases: Aurum and GOLD. CPRD Aurum includes data from contributing general practices in England that use the EMIS Web patient management software, and CPRD GOLD consists of patient data from practices across all four UK nations that use the Vision system. Both datasets are broadly representative of the UK population.^47^ ^48^ ^49^ Primary care data from general practices in England can be linked to other datasets, including hospital admissions in Hospital Episode Statistics, and mortality and index of multiple deprivation data from the Office for National Statistics (ONS). Individual patients can opt-out of sharing their records with CPRD, and individual patient consent was not required as all data were deidentified.

### Study population

We delineated two cohorts, one each from Aurum and GOLD. For the latter, we included patients from English practices only because linkage to hospital admission and mortality data were required in our analyses. To ensure that the study dataset would not contain any duplicate patient records, we used the bridging file provided by CPRD to identify English practices that have migrated from the GOLD to the Aurum dataset, and removed such practices from the GOLD dataset. For both cohorts, we included patients who had a first dementia diagnosis code between 1 January 1998 and 31 May 2018. Dementia was identified from Read, SNOMED, or EMIS codes used in the databases (see supplementary appendix). We defined the date of first dementia diagnosis as the date of first dementia code. Patients needed to be aged 50 years or over at the time of dementia diagnosis, have been registered with the CPRD practice for at least a year, not be prescribed an antipsychotic in the 365 days before their first dementia code, and have records that were eligible for linkage to Hospital Episodes Statistics, mortality, and index of multiple deprivation data. In addition, because anticholinesterases (such as donepezil, rivastigmine, and galantamine) may sometimes be prescribed to patients showing signs of dementia before their first dementia code, we excluded patients with an anticholinesterase prescription before their first dementia code. Supplementary figures S1 and S2 show how the two cohorts for Aurum and GOLD, respectively, were delineated.

### Study design

*Matched cohort design*—We implemented a matched cohort design. Supplementary figure S3 shows the study design graphically.^50^ For the Aurum and GOLD cohorts separately, patients who used antipsychotics were defined as patients in each cohort issued with an antipsychotic prescription after (or on the same day as) the date of their first dementia diagnosis, with the date of first antipsychotic prescription being the index date after which outcomes were measured. For each outcome, follow-up began from the date of the first antipsychotic prescription (the index date) and ended on the earliest of date of first diagnosis of outcome (ie, the earliest recording of the outcome whether it was from the patient’s primary or secondary care or mortality records), death, transfer out of the general practice, last data collection date of the general practice, two years from the date of antipsychotics initiation, or 31 May 2018. Because patients who have experienced an outcome were potentially at higher risk of subsequently experiencing the same event, which could confound any risks associated with antipsychotic use, we excluded those with a history of the specific outcome under investigation before the index date from the analysis of that outcome. For example, we excluded patients with a record of stroke before the index date from the analysis of stroke, but they would still be eligible for the study of other outcomes. For the analysis of acute kidney injury, patients with a diagnosis of end stage kidney disease before the index date were also excluded, and a diagnosis of end stage kidney disease after the index date was an additional condition for end of follow-up.^44^

*Matched comparators*—Each patient who used antipsychotics on or after the date of their first dementia diagnosis was matched using incidence density sampling with up to 15 randomly selected patients who had the same date of first dementia diagnosis (or up to 56 days after) and who had not been prescribed an antipsychotic before diagnosis. Incidence density sampling involves matching on sampling time, with each antipsychotic user in our study being matched to one or more comparators who were eligible for an antipsychotic but had not become a user at the time of matching.^51^ The selection of comparators was done with replacement—that is, an individual could be used as a comparator in multiple matched sets. In our study, this meant that patients were eligible to be a non-user matched comparator up to the date of their first antipsychotic prescription. We excluded matched comparators with a history of the specific outcome under investigation before the index date from the analysis of that event. For each outcome, follow-up of matched comparators began on the same day as the patient to whom they were matched (the index date) and ended on the earliest of date of their first antipsychotic prescription (if any), or date of one of the end of follow-up events described earlier for the antipsychotic users.

### Use of antipsychotics

We included both typical and atypical antipsychotics, identified by product codes in Aurum and GOLD (see supplementary appendix for list of drugs included). Senior author DMA (pharmacist) reviewed the code lists. As previous studies have shown a temporal association between antipsychotic use and development of adverse outcomes,^30^ ^31^ ^52^ we treated use of antipsychotics as a time varying variable, classified as current, recent, and past use. Current use was defined as the first 90 days from the date of an antipsychotic prescription, recent use as up to 180 days after current use ended, and past use as the time after the recent use period had ended. If a patient was issued another prescription during the 90 days after their last prescription, their current use period would be extended by 90 days from the date of their latest prescription. For example, if a patient had two prescriptions and the second was issued 60 days after the first, their current use period would be a total of 150 days: 60 days after the first prescription plus 90 days after the second. At the end of the 150 days current use period, the next 180 days would be the recent use period, and the time after this recent use period would be past use. As patients could have multiple prescriptions over time, they could move between the three antipsychotic use categories during follow-up, and they could therefore be defined as current, recent, or past users more than once. See the supplementary appendix for further information on how this definition is applied.

In post hoc analyses, we also investigated typical versus atypical antipsychotics, and specific drug substances: haloperidol, risperidone, quetiapine, and other antipsychotics (as a combined category).

### Outcomes

Outcomes were stroke, venous thromboembolism (including deep vein thrombosis and pulmonary embolism), myocardial infarction, heart failure, ventricular arrhythmia, fracture, pneumonia, and acute kidney injury. With the exceptions of pneumonia and acute kidney injury, outcomes were identified by Read, SNOMED, or EMIS codes in the primary care records, and by ICD-10 (international classification of diseases, 10th revision) codes from linked secondary care data from Hospital Episodes Statistics, and cause of death data from the ONS mortality records. For pneumonia and acute kidney injury, we only included those that were diagnosed in hospitals or as a cause of death, ascertained from Hospital Episodes Statistics and ONS data.

We also investigated appendicitis and cholecystitis combined as an unrelated (negative control) outcome to detect potential unmeasured confounding.^53^ These outcomes were chosen because evidence of an association with antipsychotic use is lacking from the literature. We identified appendicitis and cholecystitis from Read, SNOMED, EMIS, and ICD-10 codes. Clinicians (BG, AJA, DRM) checked all code lists (see supplementary appendix).

### Covariates

We used propensity score methods to control for imbalances in measurable patient characteristics between antipsychotic users and their matched non-users, with personal characteristics, lifestyle, comorbidities, and prescribed drugs included in the propensity score models. A counterfactual framework for causal inference was applied to estimate the average treatment effect adjusting for inverse probability of treatment weights generated from the propensity score models.^54^ ^55^ Selection of covariates was informed by the literature, based on their potential associations with antipsychotic initiation and study outcomes.^31^ ^34^ ^44^ ^56^ ^57^ All variables were assessed before the index date (see supplementary figure S3). Variables for personal characteristics included sex, age at dementia diagnosis, age at start of follow-up, ethnicity, and index of multiple deprivation fifths based on the location of the general practice. Comorbidities were derived as dichotomous variables and included a history of hypertension, types 1 and 2 diabetes mellitus, chronic obstructive pulmonary disease, rheumatoid arthritis, moderate or severe renal disease, moderate or severe liver disease, atrial fibrillation, cancer, and serious mental illness (bipolar disorders, schizophrenia, schizoaffective disorders, and other psychotic disorders). Lifestyle factors included smoking status and alcohol use. Medication covariates were represented as dichotomous indicators, defined by at least two prescriptions for each of the following drugs in the 12 months before the index date: antiplatelets, oral anticoagulants, angiotensin converting enzyme inhibitors or angiotensin II receptor blockers, alpha blockers, beta blockers, calcium channel blockers, diuretics, lipid lowering drugs, insulin and antidiabetic drugs, non-steroidal anti-inflammatory drugs, antidepressants, benzodiazepines, and lithium. We also included the following potential confounders for the investigations of venous thromboembolism and fracture: prescriptions for hormone replacement therapy and selective oestrogen receptor modulators (for venous thromboembolism),^58^ ^59^ a history of inflammatory bowel disease (for pneumonia and fracture),^60^ ^61^ and prescriptions for immunosuppressants, oral corticosteroids, and inhaled corticosteroids (for pneumonia).^62^ ^63^

### Statistical analysis

For each patient included in the study, we derived a propensity score representing the patient’s probability of receiving antipsychotic treatment. Propensity scores were estimated using multivariable logistic regression, with antipsychotic use as the dependent variable. Predictors included personal characteristics, lifestyle, comorbidities, and prescribed drugs. Patients with missing information on ethnicity, index of multiple deprivation, smoking, or alcohol use were grouped into an unknown category for each of these variables and included in the propensity score models. We used the Hosmer-Lemeshow test and likelihood ratio test to test the fit of the models, and interaction terms were included to improve the model fit.^64^ The derived scores were used as inverse probability of treatment weights to reweigh the data, balancing the distribution of baseline covariates between antipsychotic users and non-users (matched comparators)—that is, standardised differences <0.1 after weighting.^65^ Propensity score models were run for each outcome, and for the Aurum and GOLD cohorts separately. For further information, see the supplementary appendix section on propensity score methods to control for potential confounding.

Analyses for estimating harms were then conducted after combining (appending) the Aurum and GOLD datasets. We used Cox regression survival analyses to estimate the risks of each outcome associated with antipsychotic use relative to the comparator cohort, and we report the results as hazard ratios. Use of an antipsychotic was treated as a time varying variable. To account for the matched design, we fitted stratified models according to the matched sets and used robust variance estimation. In all models, we also included a covariate indicating whether the patient was from the Aurum or GOLD cohort and calculated hazard ratios with adjustments for inverse probability of treatment weights. Cox regression assumes proportional hazards—that is, the relative hazard of the outcome remains constant during the follow-up period.^66^ We assessed this assumption using the Grambsch-Therneau test based on the Schoenfeld residuals.^67^ Because this assumption did not hold for all outcomes examined, in addition to reporting the hazard ratios pertaining to the whole follow-up period, we estimated hazard ratios separately for the several time windows: the first seven days, 8-30 days, 31-180 days, 181-365 days, and 366 days to two years (see supplementary appendix for an illustration of stratification of follow-up time). For each outcome, we calculated the incidence rate and the number needed to harm (NNH) over the first 180 days as well as two years after start of follow-up. The NNH represents the number of patients needed to be treated with an antipsychotic for one additional patient to experience the outcome compared with no treatment. We also calculated cumulative incidence percentages (absolute risks) for each outcome accounting for competing mortality risks based on previous recommendations.^68^ These were calculated at 90 days, 180 days, 365 days, and two years after start of follow-up for antipsychotic users and their matched comparators separately. We also reported the difference in cumulative incidence between antipsychotic users and their matched comparators at these time points. Analyses were conducted using Stata/MP v16.1.

#### Sensitivity analyses

We investigated two other definitions of antipsychotic use as sensitivity analyses: the first 60 days as current use followed by 120 days of recent use, and a current use period of 30 days followed by a recent use period of 60 days. We also conducted the following post hoc sensitivity analyses. Firstly, as levomepromazine is often prescribed in palliative care to treat distressing symptoms in the last days of life,^69^ we censored individuals at the time of their first levomepromazine prescription. Secondly, we used Fine-Gray subdistribution hazard regression models to estimate the hazard of each adverse outcome, accounting for the competing risks of death.^70^ These results were reported as subhazard ratios. Thirdly, we compared the incidence rates and hazards of adverse outcomes for male versus female individuals. For these sex specific analyses, we modified the existing matched cohort by excluding non-user comparators who were of a different sex from the antipsychotic user to whom they were matched. We then derived a new propensity score for each individual by excluding sex as a covariate in the propensity score models. Incidence rate ratios and corresponding 95% confidence intervals (CIs) for male versus female individuals were calculated using the ‘iri’ command in Stata. To investigate whether hazards of each adverse outcome associated with antipsychotic use differed by sex, we fitted Cox regression models with sex, antipsychotic use, and their interaction as covariates. Sex specific hazard ratios and ratios of male to female hazard ratios were reported.

### Patient and public involvement

This study is part of a National Institute of Health and Care Research funded programme (RP-PG-1214-20012): Avoiding patient harm through the application of prescribing safety indicators in English general practices (PRoTeCT). Two patient and public involvement members in the project team contributed to the study design and protocol of this study. Our study was not, however, coproduced with people with dementia or their carers.

## Results

### Characteristics of study population

A total of 173 910 adults (63.0% women) with dementia were eligible for inclusion in the study: 139 772 (62.9% women) in the Aurum dataset and 34 138 (63.4% women) in GOLD. The mean age at dementia diagnosis for individuals in both cohorts was 82.1 years (standard deviation (SD) 7.9 years), and the median age was 83 years (interquartile range (IQR) 78-88 years in Aurum and 78-87 years in GOLD). A total of 35 339 individuals (62.5% women; 28 187 in Aurum, 62.6% women; 7152 in GOLD, 62.5% women) were prescribed an antipsychotic during the study period, and a matched set was generated for each of these individuals. The mean number of days between first dementia diagnosis and date of a first antipsychotic prescription was 693.8 ((SD 771.1), median 443 days) in Aurum and 576.6 ((SD 670.0), median 342 days) in GOLD. A total of 544 203 antipsychotic prescriptions (433 694 in Aurum, 110 509 in GOLD) were issued, of which 25.3% were for a typical antipsychotic and 74.7% for an atypical antipsychotic. The most prescribed antipsychotics were risperidone (29.8% of all prescriptions), quetiapine (28.7%), haloperidol (10.5%), and olanzapine (8.8%), which together accounted for almost 80% of all prescriptions (see supplementary table S1).

Since we excluded people with a history of the event before the start of follow-up, the number of individuals and matched sets included in analysis varies by outcome. Table 1 shows the baseline characteristics of patients for the analysis of stroke, before and after inverse probability of treatment weighting. Antipsychotic users were more likely than their matched comparators to have a history of serious mental illness and to be prescribed antidepressants or benzodiazepines in the 12 months before start of follow-up. After inverse probability of treatment weighting, standardised differences were <0.1 for all covariates. Baseline characteristics of individuals included in the analyses of other outcomes were similar to those reported for stroke (see supplementary tables S2-S9).

**Table 1:** Baseline characteristics of antipsychotic users and matched comparators included in the analysis of stroke (CPRD Aurum and GOLD combined data). Values are number (percentage) unless stated otherwise

Characteristics	Before IPT weighting				After IPT weighting		
	Antipsychotic users (n=24 696)	Matched comparators (n=344 232)	Standardised difference		Antipsychotic users (n=24 696)	Matched comparators (n=344 232)	Standardised difference
**Personal**							
Sex:							
Male	8797 (35.6)	117 350 (34.1)	0.032		8422 (34.1)	117 702 (34.2)	−0.002
Female	15 899 (64.4)	226 882 (65.9)	−0.032		16 274 (65.9)	226 530 (65.8)	0.002
Mean (SD) age at dementia diagnosis (years)	81.1 (8.2)	80.5 (8.0)	0.079		80.5 (8.0)	80.5 (8.0)	0.003
Mean (SD) age at start of follow-up (years)	82.7 (8.1)	81.8 (8.0)	0.118		81.8 (8.0)	81.9 (8.0)	−0.003
Ethnicity:							
White	18 054 (73.1)	260 176 (75.6)	−0.057		18 384 (74.4)	259 573 (75.4)	−0.022
Non-white	482 (2.0)	8603 (2.5)	−0.037		627 (2.5)	8476 (2.5)	0.005
Unknown	6160 (24.9)	75 453 (21.9)	0.071		5685 (23.0)	76 183 (22.1)	0.021
Index of multiple deprivation (fifth):							
1 (least deprived)	5380 (21.8)	79 223 (23.0)	−0.029		5594 (22.7)	78 936 (22.9)	−0.007
2	5505 (22.3)	78 571 (22.8)	−0.013		5599 (22.7)	78 443 (22.8)	−0.003
3	5270 (21.3)	69 935 (20.3)	0.025		4984 (20.2)	70 165 (20.4)	−0.005
4	4550 (18.4)	63 054 (18.3)	0.003		4569 (18.5)	63 083 (18.3)	0.004
5 (most deprived)	3971 (16.1)	53 182 (15.4)	0.017		3932 (15.9)	53 338 (15.5)	0.012
Unknown	20 (0.1)	267 (0.1)	0.001		18 (0.1)	268 (0.1)	−0.001
**Lifestyle**							
Smoking status:							
Current smoker	4133 (16.7)	58 369 (17.0)	−0.006		4215 (17.1)	58 327 (16.9)	0.003
Former smoker	10 661 (43.2)	149 402 (43.4)	−0.005		10 579 (42.8)	149 328 (43.4)	−0.011
Never smoker	8344 (33.8)	116 942 (34.0)	−0.004		8428 (34.1)	116 902 (34.0)	0.004
Unknown	1558 (6.3)	19 519 (5.7)	0.027		1474 (6.0)	19 675 (5.7)	0.011
Alcohol use:							
None	3926 (15.9)	51 991 (15.1)	0.022		3831 (15.5)	52 183 (15.2)	0.010
Light intake	2809 (11.4)	40 192 (11.7)	−0.009		2858 (11.6)	40 120 (11.7)	−0.003
Former intake	1019 (4.1)	12 400 (3.6)	0.027		898 (3.6)	12 521 (3.6)	0.000
Moderate intake	9180 (37.2)	133 861 (38.9)	−0.035		9400 (38.1)	133 444 (38.8)	−0.014
Heavy intake	1049 (4.2)	15 632 (4.5)	−0.014		1126 (4.6)	15 566 (4.5)	0.002
Unknown	6713 (27.2)	90 156 (26.2)	0.022		6582 (26.7)	90 398 (26.3)	0.009
Comorbidities*							
Hypertension	9295 (37.6)	134 476 (39.1)	−0.029		9543 (38.6)	134 138 (39.0)	−0.007
Diabetes	3339 (13.5)	47 883 (13.9)	−0.011		3411 (13.8)	47 788 (13.9)	−0.002
COPD	4416 (17.9)	57 819 (16.8)	0.029		4229 (17.1)	58 073 (16.9)	0.007
Rheumatoid arthritis	501 (2.0)	6744 (2.0)	0.005		498 (2.0)	6761 (2.0)	0.004
Moderate or severe renal disease	5263 (21.3)	67 575 (19.6)	0.042		4787 (19.4)	67 949 (19.7)	−0.009
Moderate or severe liver disease	168 (0.7)	2462 (0.7)	−0.004		179 (0.7)	2454 (0.7)	0.001
Atrial fibrillation	3057 (12.4)	39 256 (11.4)	0.030		2827 (11.4)	39 479 (11.5)	−0.001
Cancer	4001 (16.2)	45 669 (13.3)	0.083		3313 (13.4)	46 342 (13.5)	−0.001
Serious mental illness	569 (2.3)	3336 (1.0)	0.105		297 (1.2)	3659 (1.1)	0.011
**Prescribed drugs**							
Antiplatelets	8296 (33.6)	114 445 (33.2)	0.007		8247 (33.4)	114 528 (33.3)	0.003
Oral anticoagulants	1494 (6.0)	19 948 (5.8)	0.011		1421 (5.8)	20 006 (5.8)	−0.002
ACE inhibitors or ARB	5806 (23.5)	90 494 (26.3)	−0.064		6381 (25.8)	89 845 (26.1)	−0.006
Alpha blockers	1549 (6.3)	22 513 (6.5)	−0.011		1583 (6.4)	22 447 (6.5)	−0.005
Beta blockers	4489 (18.2)	61 824 (18.0)	0.006		4417 (17.9)	61 874 (18.0)	−0.002
Calcium channel blockers	4356 (17.6)	67 714 (19.7)	−0.052		4814 (19.5)	67 240 (19.5)	−0.001
Diuretics	6944 (28.1)	95 753 (27.8)	0.007		6886 (27.9)	95 822 (27.8)	0.001
Lipid lowering drugs	6622 (26.8)	100 465 (29.2)	−0.053		7091 (28.7)	99 902 (29.0)	−0.007
Insulin and antidiabetic drugs	2230 (9.0)	33 032 (9.6)	−0.019		2357 (9.5)	32 898 (9.6)	−0.001
NSAID	2970 (12.0)	40 659 (11.8)	0.007		2937 (11.9)	40 712 (11.8)	0.002
Antidepressants	8585 (34.8)	90 079 (26.2)	0.188		6676 (27.0)	92 073 (26.7)	0.006
Benzodiazepines	3686 (14.9)	22 119 (6.4)	0.278		1766 (7.2)	24 089 (7.0)	0.005
Lithium	73 (0.3)	818 (0.2)	0.011		75 (0.3)	833 (0.2)	0.012

See supplementary tables S2 to S9 for baseline characteristics for other outcomes.

ACE=angiotensin converting enzyme; ARB=angiotensin receptor blocker; COPD=chronic obstructive pulmonary disease; CPRD=Clinical Practice Research Datalink; IPT=inverse probability of treatment; NSAID=non-steroidal anti-inflammatory drug; SD=standard deviation.

* History of the condition.

### Incidence rates and relative hazards of adverse outcomes

#### All antipsychotics

**Fig 1:**
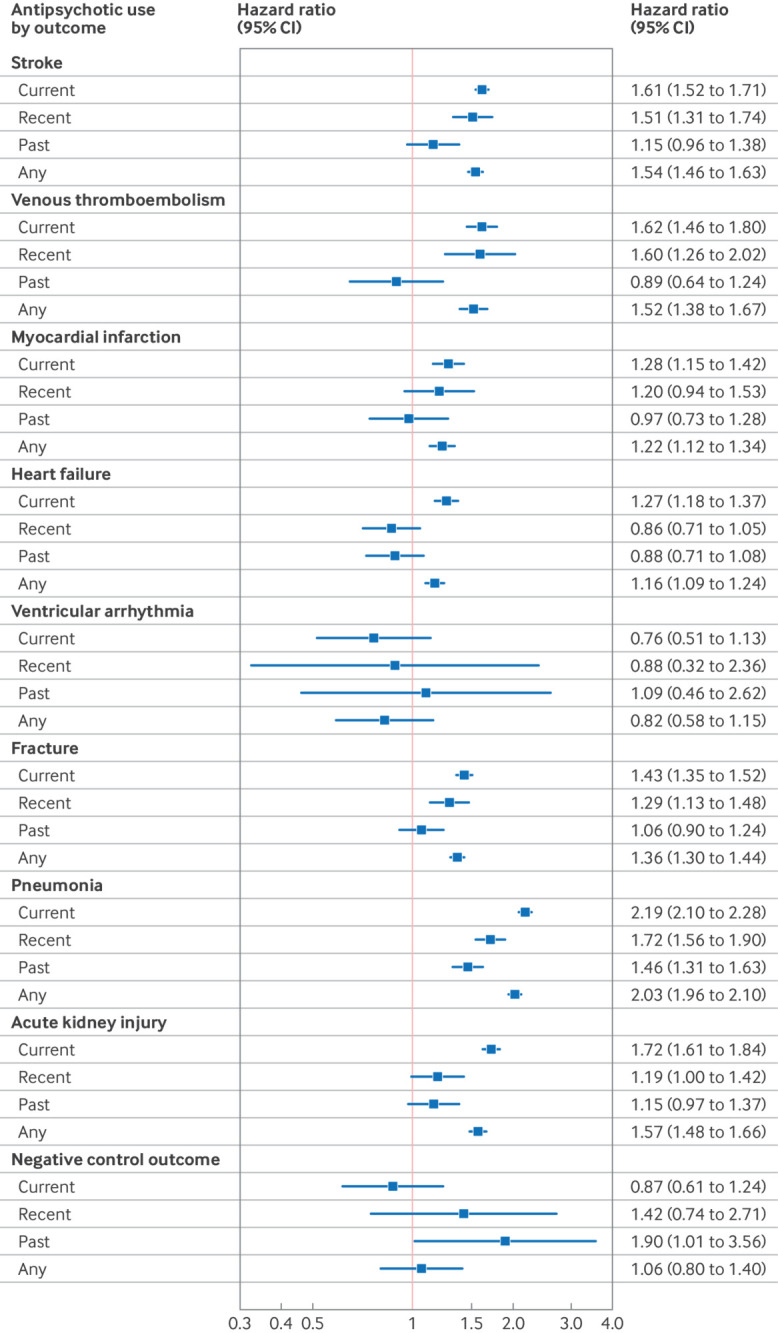
Hazard ratios (adjusted for inverse probability of treatment weights) of adverse outcomes associated with current, recent, and past antipsychotic use; with current use being defined as the first 90 days from the date of an antipsychotic prescription, recent use as up to 180 days after current use ended, and past use as after recent use. CI=confidence interval

In the two years after initiation of antipsychotics, the highest incidence rates of adverse outcomes were for pneumonia, fracture, and stroke, and ventricular arrhythmias were rare (table 2). Figure 1 shows the hazard ratios of adverse outcomes associated with current, recent, past, and any use of antipsychotics versus non-use (ie, matched comparators). Except for ventricular arrhythmia, any use of antipsychotics was associated with increased risks for all adverse outcomes, ranging from a hazard ratio of 2.03 (95% CI 1.96 to 2.10) for pneumonia to 1.16 (1.09 to 1.24) for heart failure. Current use (ie, prescribed in the previous 90 days) was associated with high risks for pneumonia (2.19, 2.10 to 2.28), acute kidney injury (1.72, 1.61 to 1.84), venous thromboembolism (1.62, 1.46 to 1.80), and stroke (1.61, 1.52 to 1.71). Recent antipsychotic use (ie, in the 180 days after current use ended) was also associated with increased risk for these outcomes, as well as for fracture, but past use of antipsychotics (ie, after recent use ended) was not associated with increased risks of the adverse outcomes examined, except for pneumonia. For the negative control outcome (appendicitis and cholecystitis), no significant associations were found with current, recent, or any antipsychotic use, but a statistically significant association was observed with past use (1.90, 1.01 to 3.56).

**Table 2:** Incidence rate (per 10 000 person years) and number needed to harm of adverse outcomes associated with antipsychotic use during the first 180 days and two years of follow-up period

	180 days after start of follow-up					Two years after start of follow-up			
	No of outcomes	Person years	Incidence rate per 10 000 person years (95% CI)	NNH (95% CI)		No of outcomes	Person years	Incidence rate per 10 000 person years (95% CI)	NNH (95% CI)
**Stroke**									
Antipsychotic user	673	9075	741.6 (687.6 to 799.8)	29 (25 to 35)		1493	24 555	608.0 (578.0 to 639.7)	41 (36 to 47)
Matched comparators	6046	151 712	398.5 (388.6 to 408.7)			16 694	460 387	362.6 (357.1 to 368.2)	
**Venous thromboembolism**									
Antipsychotic user	218	11 181	195.0 (170.7 to 222.7)	107 (83 to 149)		494	30 315	163.0 (149.2 to 178.0)	167 (134 to 221)
Matched comparators	1950	192 168	101.5 (97.1 to 106.1)			6035	585 379	103.1 (100.5 to 105.7)	
**Myocardial infarction**									
Antipsychotic user	206	10 817	190.4 (166.1 to 218.3)	167 (116 to 301)		502	29 518	170.1 (155.8 to 185.6)	254 (183 to 413)
Matched comparators	2420	185 230	130.6 (125.5 to 136.0)			7380	564 626	130.7 (127.8 to 133.7)	
**Heart failure**									
Antipsychotic user	476	10 466	454.8 (415.7 to 497.6)	63 (50 to 86)		978	28 603	341.9 (321.2 to 364.0)	166 (122 to 260)
Matched comparators	5275	177 578	297.1 (289.1 to 305.2)			15 278	542 612	281.6 (277.1 to 286.1)	
**Ventricular arrhythmia**									
Antipsychotic user	16	11 807	13.6 (8.3 to 22.1)	NA†		40	32 143	12.4 (9.1 to 17.0)	NA†
Matched comparators	321	204 697	15.7 (14.1 to 17.5)			886	623 749	14.2 (13.3 to 15.2)	
**Fracture**									
Antipsychotic user	626	7587	825.1 (762.9 to 892.3)	40 (32 to 54)		1574	20 255	777.1 (739.6 to 816.4)	45 (38 to 55)
Matched comparators	7088	123 179	575.4 (562.2 to 589.0)			20 764	373 288	556.2 (548.7 to 563.9)	
**Pneumonia**									
Antipsychotic user	1849	10 909	1694.9 (1619.4 to 1774.0)	9 (9 to 10)		3807	29 659	1283.6 (1243.5 to 1325.0)	15 (14 to 16)
Matched comparators	11 160	185 609	601.3 (590.2 to 612.5)			34 209	566 545	603.8 (597.5 to 610.3)	
**Acute kidney injury**									
Antipsychotic user	657	11 213	585.9 (542.8 to 632.5)	35 (30 to 42)		1300	30 583	425.1 (402.6 to 448.8)	84 (70 to 105)
Matched comparators	5706	190 438	299.6 (292.0 to 307.5)			17 848	582 080	306.6 (302.2 to 311.2)	
Negative control outcome*									
Antipsychotic user	19	11 090	17.1 (10.9 to 26.9)	NA†		53	30 192	17.6 (13.4 to 23.0)	NA†
Matched comparators	344	191 110	18.0 (16.2 to 20.0)			1026	582 200	17.6 (16.6 to 18.7)	

CI=confidence interval; NA=not applicable; NNH=number needed to harm.

* Appendicitis and cholecystitis.

† No significant difference between incidence rate for antipsychotic users and incidence rate for matched comparators.

Table 2 shows that the NNH ranged from 9 (95% CI 9 to 10) for pneumonia to 167 (116 to 301) for myocardial infarction during the first 180 days after initiation of antipsychotics, and from 15 (14 to 16) for pneumonia to 254 (183 to 413) for myocardial infarction after two years. These figures suggest that over the 180 days after drug initiation, use of antipsychotics might be associated with one additional case of pneumonia for every nine patients treated, and one additional case of myocardial infarction for every 167 patients treated. At two years, there might be one additional case of pneumonia for every 15 patients treated, and one additional case of myocardial infarction for every 254 patients treated.

Table 3 shows hazard ratios stratified by follow-up time (except for ventricular arrhythmia and the negative control where the number of patients was very low). For almost all outcomes, relative hazards were highest in the first seven days after initiation of antipsychotic treatment. Risks for pneumonia were particularly increased in the first seven days (9.99, 8.78 to 11.40) and remained substantial afterwards (3.39, 3.04 to 3.77, 8-30 days). No increased risks for heart failure were found for current users after 180 days from start of treatment, nor for myocardial infarction one year after drug initiation. However, risks for stroke, venous thromboembolism, fracture, pneumonia, and acute kidney injury remained increased among continuous antipsychotic users up to two years after initiation of treatment.

**Table 3:** Hazard ratios (adjusted for IPT weights) of adverse outcomes associated with current, recent, and past antipsychotic use stratified by follow-up period

Antipsychotic use	Follow-up period (hazard ratio (95% CI))				
	0-7 days	8-30 days	31-180 days	181-365 days	366 days-2 years
**Stroke**					
Current	3.75 (3.00 to 4.69)	1.57 (1.28 to 1.92)	1.54 (1.39 to 1.70)	1.52 (1.34 to 1.73)	1.55 (1.38 to 1.74)
Recent	-	-	1.72 (1.35 to 2.20)	1.47 (1.19 to 1.82)	1.32 (0.98 to 1.79)
Past	-	-	-	1.66 (1.15 to 2.39)	1.04 (0.85 to 1.28)
**Venous thromboembolism**					
Current	2.05 (1.19 to 3.56)	1.92 (1.36 to 2.70)	1.67 (1.41 to 1.99)	1.39 (1.10 to 1.75)	1.61 (1.33 to 1.96)
Recent	-	-	2.14 (1.46 to 3.15)	1.27 (0.86 to 1.89)	1.58 (1.01 to 2.48)
Past	-	-	-	0.62 (0.23 to 1.65)	0.95 (0.67 to 1.35)
**Myocardial infarction**					
Current	2.33 (1.41 to 3.83)	1.61 (1.15 to 2.26)	1.27 (1.06 to 1.52)	1.39 (1.13 to 1.70)	1.02 (0.83 to 1.27)
Recent	-	-	0.89 (0.52 to 1.52)	1.34 (0.96 to 1.88)	1.28 (0.80 to 2.03)
Past	-	-	-	1.25 (0.70 to 2.23)	0.91 (0.66 to 1.25)
**Heart failure**					
Current	2.85 (2.15 to 3.78)	1.95 (1.59 to 2.40)	1.32 (1.17 to 1.49)	1.12 (0.95 to 1.31)	0.97 (0.82 to 1.14)
Recent	-	-	0.99 (0.72 to 1.37)	0.80 (0.59 to 1.09)	0.80 (0.54 to 1.20)
Past	-	-	-	1.11 (0.74 to 1.67)	0.81 (0.63 to 1.04)
**Fracture**					
Current	2.22 (1.66 to 2.98)	1.49 (1.22 to 1.83)	1.37 (1.24 to 1.52)	1.29 (1.14 to 1.46)	1.53 (1.38 to 1.71)
Recent	-	-	1.07 (0.82 to 1.41)	1.28 (1.04 to 1.58)	1.61 (1.25 to 2.07)
Past	-	-	-	1.02 (0.69 to 1.50)	1.07 (0.89 to 1.28)
**Pneumonia**					
Current	9.99 (8.78 to 11.40)	3.39 (3.04 to 3.77)	2.03 (1.89 to 2.17)	1.79 (1.64 to 1.95)	1.71 (1.58 to 1.85)
Recent	-	-	1.93 (1.63 to 2.29)	1.77 (1.53 to 2.05)	1.40 (1.14 to 1.72)
Past	-	-	-	1.38 (1.07 to 1.79)	1.48 (1.32 to 1.67)
**Acute kidney injury**					
Current	3.79 (2.96 to 4.87)	2.61 (2.17 to 3.13)	2.03 (1.84 to 2.25)	1.27 (1.09 to 1.48)	1.26 (1.10 to 1.44)
Recent	-	-	1.36 (1.03 to 1.81)	1.05 (0.80 to 1.38)	1.23 (0.85 to 1.79)
Past	-	-	-	1.47 (1.03 to 2.09)	1.08 (0.88 to 1.32)

Current use is defined as the first 90 days from the date of an antipsychotic prescription, recent use as up to 180 days after current use ended, and past use as after recent use (not reported for ventricular arrhythmia and negative control outcome (appendicitis and cholecystitis) because of small numbers).

CI=confidence interval; IPT=inverse probability of treatment.

#### Types of antipsychotics

During the current use period of 90 days after a prescription, both typical and atypical antipsychotics were associated with increased risks of all adverse outcomes compared with non-use, except for ventricular arrhythmia and the negative control (see supplementary table S10). Hazards were higher when current use of typical antipsychotics was directly compared with atypical antipsychotics for stroke (1.23, 1.09 to 1.40), heart failure (1.18, 1.01 to 1.39), fracture (1.22, 1.08 to 1.38), pneumonia (1.92, 1.77 to 2.08), and acute kidney injury (1.22, 1.05 to 1.42), but no significant differences between the two types of drug were found for the risks of venous thromboembolism or myocardial infarction.

Supplementary table S11 shows the risks of adverse outcomes associated with haloperidol (the most prescribed typical antipsychotic) and with risperidone and quetiapine (the two most prescribed atypical antipsychotics). Current use of risperidone and haloperidol compared with non-use was associated with increased risks of all adverse outcomes except for ventricular arrhythmia and the negative control. Current use of quetiapine compared with non-use was associated with increased risks for fracture, pneumonia, and acute kidney injury. Among current users of haloperidol or risperidone, risks for fracture, pneumonia, and acute kidney injury were higher for haloperidol versus risperidone, but risks for stroke, venous thromboembolism, myocardial infarction, and heart failure were similar for both drugs. With the exceptions of myocardial infarction, ventricular arrhythmia, and the negative control, risks of all adverse outcomes were higher for haloperidol than for quetiapine, especially for pneumonia (2.53, 2.21 to 2.89) and venous thromboembolism (1.99, 1.33 to 2.97). Among current users of quetiapine compared with risperidone, there were no significant differences in risks for myocardial infarction, heart failure, or fracture. However, risks for stroke (0.64, 0.53 to 0.78), venous thromboembolism (0.49, 0.36 to 0.68), pneumonia (0.72, 0.63 to 0.81), and acute kidney injury (0.81, 0.67 to 0.96) were lower for quetiapine than for risperidone.

### Absolute risks of adverse outcomes

Cumulative incidence for all outcomes examined was higher for antipsychotic users versus matched comparators, except for ventricular arrhythmia and the negative control (table 4). The absolute risk, as well as risk difference, was particularly large for pneumonia. In the 90 days after initiation of an antipsychotic, the cumulative incidence of pneumonia among antipsychotic users was 4.48% (95% CI 4.26% to 4.71%) *v* 1.49% (1.45% to 1.53%) in the matched cohort of non-users (difference 2.99%, 95% CI 2.77% to 3.22%). At one year, this increased to 10.41% (10.05% to 10.78%) for antipsychotic users compared with 5.63% (5.55% to 5.70%) for non-users (difference 4.78%, 4.41% to 5.16%).

**Table 4:** Cumulative incidence of adverse outcomes associated with antipsychotic use at 90, 180, and 365 days and at two years after start of follow-up

	90 days			180 days			365 days			2 years	
	No	Cumulative incidence (% (95% CI))		No	Cumulative incidence (% (95% CI))		No	Cumulative incidence (% (95% CI))		No	Cumulative incidence (% (95% CI))
**Stroke**											
Antipsychotic user	412	1.74 (1.59 to 1.91)		673	2.96 (2.75 to 3.19)		1041	4.89 (4.61 to 5.19)		1493	7.75 (7.38 to 8.14)
Matched comparators	3419	1.04 (1.01 to 1.08)		6046	1.91 (1.86 to 1.96)		10 420	3.50 (3.43 to 3.56)		16 694	6.28 (6.19 to 6.37)
Difference		0.70 (0.54 to 0.87)			1.06 (0.84 to 1.28)			1.39 (1.10 to 1.70)			1.47 (1.08 to 1.87)
**Venous thromboembolism**											
Antipsychotic user	114	0.39 (0.32 to 0.46)		218	0.79 (0.69 to 0.90)		329	1.25 (1.12 to 1.39)		494	2.09 (1.91 to 2.28)
Matched comparators	1052	0.26 (0.24 to 0.27)		1950	0.49 (0.47 to 0.51)		3607	0.97 (0.94 to 1.00)		6035	1.83 (1.78 to 1.88)
Difference		0.13 (0.06 to 0.21)			0.30 (0.20 to 0.41)			0.29 (0.15 to 0.43)			0.26 (0.08 to 0.46)
**Myocardial infarction**											
Antipsychotic user	130	0.46 (0.39 to 0.54)		206	0.75 (0.65 to 0.86)		350	1.39 (1.25 to 1.54)		502	2.19 (2.00 to 2.39)
Matched comparators	1319	0.33 (0.31 to 0.35)		2420	0.63 (0.61 to 0.66)		4429	1.23 (1.20 to 1.27)		7380	2.31 (2.26 to 2.37)
Difference		0.13 (0.06 to 0.21)			0.12 (0.02 to 0.23)			0.16 (0.01 to 0.31)			−0.13 (−0.32 to 0.08)
**Heart failure**											
Antipsychotic user	311	1.15 (1.03 to 1.27)		476	1.81 (1.66 to 1.97)		712	2.89 (2.69 to 3.11)		978	4.37 (4.10 to 4.64)
Matched comparators	2824	0.74 (0.72 to 0.77)		5275	1.43 (1.39 to 1.47)		9462	2.73 (2.68 to 2.79)		15 278	4.95 (4.87 to 5.02)
Difference		0.40 (0.28 to 0.53)			0.38 (0.22 to 0.55)			0.16 (−0.05 to 0.38)			−0.58 (−0.86 to −0.29)
Ventricular arrhythmia*											
Antipsychotic user	10	-		16	-		30	0.11 (0.08 to 0.15)		40	0.16 (0.11 to 0.21)
Matched comparators	181	-		321	-		549	0.14 (0.13 to 0.15)		886	0.25 (0.23 to 0.26)
Difference		-			-			−0.03 (−0.06 to 0.02)			−0.09 (−0.14 to −0.03)
**Fracture**											
Antipsychotic user	367	1.88 (1.70 to 2.07)		626	3.34 (3.09 to 3.60)		1016	5.80 (5.46 to 6.15)		1574	9.99 (9.52 to 10.47)
Matched comparators	3777	1.42 (1.37 to 1.46)		7088	2.75 (2.69 to 2.81)		12 694	5.22 (5.13 to 5.31)		20 764	9.50 (9.37 to 9.62)
Difference		0.46 (0.28 to 0.66)			0.59 (0.33 to 0.86)			0.58 (0.23 to 0.94)			0.49 (0.01 to 0.99)
**Pneumonia**											
Antipsychotic user	1283	4.48 (4.26 to 4.71)		1849	6.72 (6.44 to 7.01)		2695	10.41 (10.05 to 10.78)		3807	16.23 (15.76 to 16.71)
Matched comparators	5945	1.49 (1.45 to 1.53)		11 160	2.89 (2.84 to 2.94)		20 395	5.63 (5.55 to 5.70)		34 209	10.59 (10.49 to 10.70)
Difference		2.99 (2.77 to 3.22)			3.83 (3.54 to 4.12)			4.78 (4.41 to 5.16)			5.64 (5.15 to 6.13)
**Acute kidney injury**											
Antipsychotic user	420	1.46 (1.33 to 1.60)		657	2.34 (2.17 to 2.52)		933	3.52 (3.31 to 3.75)		1300	5.38 (5.10 to 5.67)
Matched comparators	3020	0.74 (0.71 to 0.76)		5706	1.44 (1.41 to 1.48)		10 505	2.83 (2.78 to 2.88)		17 848	5.40 (5.32 to 5.48)
Difference		0.73 (0.60 to 0.87)			0.90 (0.73 to 1.08)			0.70 (0.47 to 0.93)			−0.02 (−0.31 to 0.28)
Negative control outcome*†											
Antipsychotic user	14	-		19	-		34	0.13 (0.10 to 0.19)		53	0.23 (0.17 to 0.30)
Matched comparators	184	-		344	-		615	0.17 (0.15 to 0.18)		1026	0.31 (0.29 to 0.33)
Difference		-			-			−0.03 (−0.07 to 0.02)			−0.08 (−0.14 to −0.01)

CI=confidence interval.

* Not estimated for 90 days or 180 days owing to small number.

† Appendicitis and cholecystitis.

### Sensitivity analyses

Similar results were found in sensitivity analysis using two other definitions of antipsychotic use (see supplementary figures S4 and S5). Of the 544 203 antipsychotic prescriptions issued, 1.3% were for levomepromazine (see supplementary table S1). Results remained similar when patients were censored at the time of their first levomepromazine prescription (see supplementary figure S6). Results of the Fine-Gray models accounting for the competing risks of death also showed broadly similar patterns of hazards to those from the Cox models (see supplementary table S12 and figure S7). Sex specific analyses showed that male patients had higher incidence rates of all adverse outcomes than female patients, except for fracture and venous thromboembolism where incidence was higher for female patients than for male patients (see supplementary table S13). Compared with female antipsychotic users, male users had increased hazards for pneumonia and acute kidney injury (male to female hazard ratio 1.16, 95% CI 1.08 to 1.25 for pneumonia and 1.22, 1.08 to 1.37 for acute kidney injury), but lower hazards for stroke (0.81, 0.73 to 0.91). No significant differences were found by sex in the hazards for venous thromboembolism, myocardial infarction, heart failure, ventricular arrhythmia, or fracture (see supplementary table S14).

## Discussion

In this population based cohort study of adults (≥50 years) with dementia, use of antipsychotics compared with non-use was associated with increased risks for stroke, venous thromboembolism, myocardial infarction, heart failure, fracture, pneumonia, and acute kidney injury. Increased risks were observed among current and recent users and were highest in the first week after initiation of treatment. In the 90 days after a prescription, relative hazards were highest for pneumonia, acute kidney injury, stroke, and venous thromboembolism, with increased risks ranging from 1.5-fold (for venous thromboembolism) to twofold (for pneumonia) compared with non-use. No increased risk was found for ventricular arrhythmia or the negative control outcome (appendicitis and cholecystitis). Absolute risk differences between antipsychotic users and their matched comparators were substantial for most adverse events, and largest for pneumonia. In the 90 days after a prescription, risks of stroke, heart failure, fracture, pneumonia, and acute kidney injury were higher for typical antipsychotics versus atypical antipsychotics, whereas no significant differences between these two drug classes were found for risks of venous thromboembolism or myocardial infarction. Haloperidol was associated with higher risks for fracture, pneumonia, and acute kidney injury than risperidone, but no significant differences between the two drugs were found for the other outcomes. Risks of almost all adverse outcomes were higher for haloperidol than for quetiapine. No significant differences were found between risperidone and quetiapine for risks of myocardial infarction, heart failure, or fracture, but risks for stroke, venous thromboembolism, pneumonia, and acute kidney injury were lower for quetiapine versus risperidone.

### Comparison with other studies

A population based study in Wales reported no increased risks for non-fatal acute cardiac events associated with antipsychotic use in patients with all cause dementia, although those with Alzheimer’s disease showed increased risks.^37^ Systematic reviews and meta-analyses of studies not limited to patients with dementia have also reported inconsistent evidence for myocardial infarction, or lack of robustness of these data.^33^ ^34^ ^71^ Our findings for myocardial infarction were similar to those in a study that first documented a modest and time limited increase in risk of this outcome associated with antipsychotic use among patients with dementia.^56^ In a study of nursing home residents in the US, users of typical, but not atypical, antipsychotics were more likely than non-users to be admitted to hospital for ventricular arrhythmia or cardiac arrest,^35^ and a study not limited to older people reported increased risks for ventricular arrhythmia or sudden cardiac death associated with both typical and atypical antipsychotics.^36^ We did not find any association with ventricular arrhythmia, but the number of events was low and we did not examine cardiac arrest or sudden death.

Increased risks of venous thromboembolism associated with antipsychotic use have been reported in the general population,^38^ but meta-analyses found increased risks of venous thromboembolism only among younger users.^39^ ^40^ Our findings are consistent with those of the Welsh study, which reported increased risks of venous thromboembolism in the 12 months after drug initiation (prior event rate ratio 1.95, 95% CI 1.83 to 2.0).^37^ In absolute terms, however, these risks were relatively low compared with other outcomes examined in this study.

We found that both the relative and the absolute risks for pneumonia were highest among all outcomes examined. Current users of antipsychotics had a twofold increased risk compared with non-users (fig 1), and although this magnitude of increased risk was comparable to previous reports,^14^ ^31^ ^32^ we additionally observed that risks were greater in the first week after drug initiation. One study also reported a particularly high risk for patients with hospital diagnosed pneumonia in the first week, but the magnitude of increase (odds ratio 4.5, 95% CI 2.8 to 7.3) was much lower than our observation.^30^ The mechanisms linking antipsychotic use and development of pneumonia is not well understood, and substantial heterogeneity exists among the drug substances, but antipsychotic induced extrapyramidal symptoms, sedation, xerostomia (dry mouth), and dyskinesia or impaired swallowing are commonly considered as potential risk factors.^72^ In addition, because elderly people with pneumonia may be less likely than younger patients to present with respiratory symptoms but more likely to show signs of delirium,^73^ it is possible that reverse causality might have contributed to the high risks observed in the early days after drug initiation, as delirium from the onset of pneumonia might have been treated with antipsychotics before pneumonia was diagnosed.^30^ However, although causality cannot be inferred, the particularly high increased risks observed for a range of outcomes and not only for pneumonia in the early days after drug initiation are consistent with other studies.^28^ This could be partly explained by further prescriptions being given only to patients who tolerated the first days of drug use.

The use of atypical antipsychotics in older adults (≥65 years) has been shown to be associated with increased risk of acute kidney injury.^44^ ^45^ ^46^ Two studies reported significantly increased risks in users compared with non-users in the 90 days after initiation of atypical antipsychotics.^44^ ^45^ In contrast, another study observed no increased risks from use of the broad category of atypical antipsychotics, although a significantly increased risk was found with olanzapine.^46^ In our study, we found increased risks of acute kidney injury with both typical and atypical antipsychotics, with risks being higher for haloperidol than for risperidone and quetiapine.

In a meta-analysis of observational studies, antipsychotic use was associated with increased risks of hip fracture among people with dementia.^41^ A self-controlled case series study of older adult patients (≥65 years) also reported increased risks of falls and fracture after initiation of antipsychotics, but incidence was found to be even higher in the 14 days before treatment started.^43^ Similar findings were also reported in another study, suggesting that the risks observed during the treatment periods might not be attributable to the antipsychotics alone.^42^ Although we cannot eliminate confounding in our study, we minimised this risk by adjusting for a large number of both clinical and non-clinical characteristics that might have influenced treatment assignment. We also found no increased risks associated with current or recent antipsychotic use for the negative control outcome (appendicitis and cholecystitis).

Our study found that the risks of stroke and heart failure were higher for typical antipsychotics than for atypical antipsychotics, but risks of venous thromboembolism and myocardial infarction were similar between the two drug classes. We also found no significant differences between haloperidol and risperidone in risks of these four outcomes, but significantly increased risks for stroke, venous thromboembolism, and heart failure for haloperidol versus quetiapine. Previous studies of elderly patients have reported similar risks for cardiovascular or cerebrovascular events associated with use of typical and atypical antipsychotics,^17^ ^74^ ^75^ ^76^ but risks of these outcomes and of all cause mortality were increased with haloperidol versus risperidone.^21^ ^76^ For fracture and pneumonia, we found that risks were higher in association with typical antipsychotics than atypical antipsychotics and for haloperidol versus risperidone or quetiapine. The findings from previous studies comparing these risks by antipsychotic types have been inconsistent.^30^ ^31^ ^32^ ^74^ ^75^

### Strengths and limitations of this study

A key strength of this study was the investigation of a wide range of adverse events in a large population based cohort, and the reporting of both relative and absolute risk differences over multiple periods. Previous studies commonly focused on a single outcome or type of outcome, such as cerebrovascular events, and on the reporting of relative risks. By examining the same cohort at risk, we were able to directly compare the hazards of multiple outcomes without differential biases between the cohorts. In addition, we only included patients with a clinician recorded diagnosis of dementia, and we adjusted for many variables that might have influenced the probability of antipsychotic initiation, seeking to minimise confounding by indication. CPRD is one of the largest primary care databases in the world, and it is broadly representative of the UK population.^47^ ^48^ ^49^ The database includes all prescriptions issued in participating primary care practices in the UK, and it is recognised as a high quality resource to support international pharmacovigilance.^77^ The longitudinal nature of CPRD, with linked data from secondary care and mortality records, enabled us to capture the study outcomes from multiple sources, as well as information on prescribing and comorbidities.^78^ ^79^ Our findings were also robust to different classifications of usage periods and we found no associations between current and recent antipsychotic use with the development of the negative control outcome (appendicitis and cholecystitis). However, a significant association with past use was observed that we are unable to explain.

As with all observational studies, residual confounding cannot be excluded. For example, polypharmacy is common among elderly people, which could lead to drug-drug interactions and potentially confound our findings.^80^ ^81^ We also did not have information on indications for antipsychotics treatment. We minimised the risk of confounding using propensity score methods to control for imbalances in measurable patient characteristics between antipsychotic users and their matched comparators. However, unlike randomised control trials, which, if properly conducted, could account for both observed and unobserved differences between treated and untreated groups, the propensity score method can only adjust for the observed differences between two groups. Additionally, our choice of covariates was based on the literature and discussions with clinical experts and was not formally structured using, for example, a directed acyclic graph. Although the strong associations with pneumonia in the first seven days of antipsychotic initiation may partially be attributed to reverse causality, however, it is less likely to explain associations over longer periods. We also found no increased risk for appendicitis and cholecystitis during current and recent use—our negative control outcome that was included to detect potential unmeasured confounding.^53^ Another limitation of our study is that although prescriptions issued in primary care are reliable in CPRD, information on dosage is not well recorded and information on drug adherence or prescriptions issued while patients are in hospital is not available.^48^ Misclassification of drug use is therefore a potential problem. As with other electronic health data that are routinely collected for administrative rather than research purposes, potential issues exist with coding errors, missing or incomplete information, and variations in data quality between practices and healthcare settings. Although the data undergo quality checks before being released and our use of the linked data would have helped to deal with such problems, we were restricted to data coded in patients’ electronic health records. In addition, despite the representativeness of the CPRD data, care should be taken in making inferences beyond the population studied. Our sex specific investigations were also conducted as post hoc analyses. By using existing matched sets but restricting the comparators to those of the same sex as the antipsychotic user to whom they were matched, the number of comparators was greatly reduced. Although we found some evidence of differences in hazards for stroke, pneumonia, and acute kidney injury between male and female antipsychotic users, further research is needed to validate these findings.

### Policy implications

The mechanisms underlying the links between antipsychotics and the outcomes in our study are not fully understood. In the UK, US, and Europe, current regulatory warnings for using antipsychotics to treat behavioural and psychological symptoms of dementia were mostly based on evidence of increased risks for stroke and mortality.^8^ ^11^ ^22^ ^23^ ^24^ ^25^ ^26^ We found a considerably wider range of harms associated with antipsychotic use in people with dementia, and the risks of harm were highest soon after initiation. Our findings must be seen in the context of trial evidence of at best modest benefit on behavioural and psychological symptoms of dementia. The efficacy of antipsychotics in the management of behavioural and psychological symptoms of dementia remains inconclusive.^82^ ^83^ ^84^ ^85^ Atypical antipsychotics, including risperidone, which is one of two antipsychotics licensed in the UK for the treatment of behavioural and psychological symptoms of dementia, have the strongest evidence base, but the benefits are only modest.^82^ ^85^

Any potential benefits of antipsychotic treatment therefore need to be weighed against the risk of serious harm across multiple outcomes. Although there may be times when an antipsychotic prescription is the least bad option, clinicians should actively consider the risks, considering patients’ pre-existing comorbidities and living support. The NNH reported in this study can help to inform clinical judgements on the appropriateness of treatments, taking account of the modest potential benefits reported in clinical trials. When prescriptions of such drugs are needed, treatment plans should be reviewed regularly with patients and their carers to reassess the need for continuing treatment.^9^ In addition, given the higher risks of adverse events in the early days after drug initiation, clinical examinations should be taken before, and clinical reviews conducted shortly after, the start of treatment. Our study reaffirms that these drugs should only be prescribed for the shortest period possible.^9^ Although regulators have made efforts to limit the use of these drugs to people with the most severe behavioural and psychological symptoms of dementia,^8^ ^82^ ^86^ antipsychotic prescribing in dementia remains common and has even increased in recent years.^4^ ^5^ ^87^ ^88^ If such trends continue, further communication on the associated risks could be considered by guideline developers or regulators after a review of the totality of evidence. Greater accountability and monitoring in the use of these drugs may be called for, and additional legal reforms may be required to regulate adherence.^89^ In recent years, other psychotropic drugs such as antidepressants, benzodiazepines, mood stabilisers, and anticonvulsants have been prescribed instead of antipsychotics for the treatment of behavioural and psychological symptoms of dementia.^28^ ^90^ ^91^ These drugs, however, also pose their own risks. Further research is needed into safer drug treatment of behavioural and psychological symptoms of dementia and more efficacious, easy to deliver, initial non-drug treatments.

### Conclusions

Antipsychotic use is associated with a wide range of serious adverse outcomes in people with dementia, with relatively large absolute risks of harm for some outcomes. These risks should be considered in future regulatory decisions, alongside cerebrovascular events and mortality. Any potential benefits of antipsychotic treatment need to be weighed against risk of serious harm, and treatment plans should be reviewed regularly. The effect of antipsychotics on behavioural and psychological symptoms of dementia is modest at best, but the proportion of people with dementia prescribed antipsychotics has increased in recent years. Our finding that antipsychotics are associated with a wider range of risks than previously known is therefore of direct relevance to guideline developers, regulators, and clinicians considering the appropriateness of antipsychotic prescribing for behavioural and psychological symptoms of dementia.

**What is already known on this topic**

Despite safety concerns, antipsychotics continue to be frequently prescribed for the management of behavioural and psychological symptoms of dementia
Current regulatory warnings for the treatment of behavioural and psychological symptoms of dementia using antipsychotics are based on evidence of increased risks of stroke and death
Evidence for other adverse outcomes is less conclusive or is more limited among people with dementia, and comparisons of risks for multiple adverse events are also difficult owing to different study designs and populations

**What this study adds**

Antipsychotic use in people with dementia was associated with increased risks of stroke, venous thromboembolism, myocardial infarction, heart failure, fracture, pneumonia, and acute kidney injury, compared with non-use, but not ventricular arrhythmia
Relative hazards were highest for pneumonia, acute kidney injury, stroke, and venous thromboembolism, and absolute risk and risk difference between antipsychotic users and their matched comparators was largest for pneumonia
Risks of these wide ranging adverse outcomes need to be considered before prescribing antipsychotic drugs to people with dementia

## Data Availability

Electronic health records are, by definition, considered sensitive data in the UK by the Data Protection Act and cannot be shared via public deposition because of information governance restriction in place to protect patient confidentiality. Access to Clinical Practice Research Datalink (CPRD) data is subject to protocol approval via CPRD’s research data governance process. For more information see https://cprd.com/data-access. Linked secondary care data from Hospital Episodes Statistics, mortality data from the Office for National Statistics, and index of multiple deprivation data can also be requested from CPRD.

## Web extra

Extra material supplied by authors

Supplementary material: Clinical code lists

## Notes

Contributors: All authors conceived and designed the study and acquired, analysed, or interpreted the data. BG, DRM, TvS, AJA, and DMA reviewed the clinical codes. PLHM conducted the statistical analyses and wrote the first draft of the manuscript. All authors critically revised the manuscript for important intellectual content and approved the final version. AJA, DMA, RAE, BG, DRM, AS, and TvS obtained the funding. PLHM is the guarantor. The corresponding author (PLHM) attests that all listed authors meet authorship criteria and that no others meeting the criteria have been omitted.

Transparency: The lead author (PLHM: the manuscript’s guarantor) affirms that the manuscript is an honest, accurate, and transparent account of the study being reported; that no important aspects of the study have been omitted; and that any discrepancies from the study as planned (and, if relevant, registered) have been explained.

Dissemination to participants and related patient and public communities: This study used anonymised electronic health records from the CPRD and it is therefore not possible to disseminate the findings directly to individuals whose data we used. This study is part of a National Institute for Health and Care Research (NIHR) funded programme (RP-PG-1214-20012): Avoiding patient harm through the application of prescribing safety indicators in English general practices (PRoTeCT). We have experienced patient and public involvement members aligned to the programme who we will consult in the results dissemination. In addition, senior author DMA is director of NIHR Greater Manchester Patient Safety Research Collaboration (GMPSRC), and co-authors MJC and AJA are affiliated with it. The Patient Safety Research Collaboration has a community of public contributors including patients, carers, and people accessing health and social care services. The authors will work with this network to disseminate findings.

Provenance and peer review: Not commissioned; externally peer reviewed.
