# Supplementary material for: Multiple adverse outcomes associated with antipsychotic use in people with dementia: population based matched cohort study
Source: BMJ. 2024 Apr 17;385:e076268. doi: 10.1136/bmj-2023-076268 (PMC11022137; doi:10.1136/bmj-2023-076268)
Supplement: Supplementary file 1 — Supplementary material: Clinical code lists [file mokp076268.ww1.pdf]

Appendix - Clinical codes lists

Codes list for dementia – Aurum and GOLD 2
Code list for antipsychotics – Aurum 9
Code list for antipsychotics – GOLD 18
Code list for stroke – ICD10 26
Code list for stroke – Aurum and GOLD 27
Code list for venous thromboembolism (VTE) – ICD10 32
Code list for venous thromboembolism (VTE) – Aurum and GOLD 33
Code list for myocardial infarction – ICD10 39
Code list for myocardial infarction – Aurum and GOLD 40
Code list for heart failure – ICD10 43
Code list for heart failure – Aurum and GOLD 44
Code list for ventricular arrhythmia – ICD10 46
Code list for ventricular arrhythmia – Aurum and GOLD 47
Code list for fracture – ICD10 48
Code list for fracture – Aurum and GOLD 55
Code list for pneumonia – ICD10 148
Code list for acute kidney injury (AKI) – ICD10 149
Code list for appendicitis and cholecystitis (negative control outcome) – ICD10 150
Code list for appendicitis and cholecystitis (negative control outcome) – Aurum and GOLD 151

Codes list for dementia – Aurum and GOLD

Source	SNOMED_CT_Concept_id	SNOMED_CT_Description_id	Medcode_Aurum	Medcode_GOLD	Readcode	Term
Aurum only	45864009	882941000006113	882941000006113		F112.99	senile brain degeneration
Aurum only	191449005	882201000006116	882201000006116		E000.99	senile dementia - simple type
Aurum only	191461002	882211000006118	882211000006118		E003.99	senile dementia-acute confused
Aurum only	268612007	882171000006115	882171000006115		E00..97	dementia
Aurum only	268612007	882181000006117	882181000006117		E00..98	other senile/presenile dement.
Aurum only	268612007	882191000006119	882191000006119		E00..99	senile and presenile dementias
Aurum only	905791000006104	905791000006115	905791000006115			[rfc] alzheimer's disease
Aurum only	914921000006101	914921000006117	914921000006117			[d] vascular dementia
Aurum only	914931000006103	914931000006119	914931000006119			[d] dementia with lewy bodies
Aurum only	914941000006108	914941000006112	914941000006112			[d] dementia
Aurum only	914951000006105	914951000006114	914951000006114			[d] dementia in alzheimer's disease
Aurum only	939491000006102	939491000006118	939491000006118			[rfc] dementia
Aurum only	1576281000006103	1576281000006119	1576281000006119			cause of death- alzheimer's disease
Aurum only	1823871000006108	1823871000006112	1823871000006112			dementia confirmed
Aurum only	1949621000006102	1949621000006118	1949621000006118			dementia stage at diagnosis
Aurum only	1949631000006104	1949631000006115	1949631000006115			dementia stage at diagnosis - early (mild)
Aurum only	1949641000006109	1949641000006113	1949641000006113			dementia stage at diagnosis - mid (moderate)
Aurum only	1949651000006106	1949651000006110	1949651000006110			dementia stage at diagnosis - late (severe)
Aurum only	1949661000006108	1949661000006112	1949661000006112			dementia stage at diagnosis - undetermined
Aurum only	1949671000006101	1949671000006117	1949671000006117			dementia stage at diagnosis - not known
Aurum only	1971401000006107	1971401000006111	1971401000006111			dementia in alzheimer's disease with early onset, without additional symptoms
Aurum only	1971541000006105	1971541000006114	1971541000006114			dementia in alzheimer's disease with early onset, other symptoms, predominantly delusional
Aurum only	1971771000006108	1971771000006112	1971771000006112			dementia in alzheimer's disease with early onset, other symptoms, predominantly hallucinatory

Aurum only	1972021000006103	1972021000006119	1972021000006119			unspecified dementia, without additional symptoms
Aurum only	1972041000006105	1972041000006114	1972041000006114			unspecified dementia, other symptoms, predominantly delusional
Aurum only	1972061000006109	1972061000006113	1972061000006113			unspecified dementia, other symptoms, predominantly hallucinatory
Aurum only	1972071000006102	1972071000006118	1972071000006118			unspecified dementia, other symptoms, predominantly depressive
Aurum only	1972081000006104	1972081000006115	1972081000006115			unspecified dementia, other mixed symptoms
Aurum only	1972131000006104	1972131000006115	1972131000006115			dementia in alzheimer's disease with early onset, other symptoms, predominantly depressive
Aurum only	1972141000006109	1972141000006113	1972141000006113			dementia in alzheimer's disease with early onset, other mixed symptoms
Aurum only	1972171000006101	1972171000006117	1972171000006117			dementia in alzheimer's disease with late onset, without additional symptoms
Aurum only	1972181000006103	1972181000006119	1972181000006119			dementia in alzheimer's disease with late onset, other symptoms, predominantly delusional
Aurum only	1972191000006100	1972191000006116	1972191000006116			dementia in alzheimer's disease with late onset, other symptoms, predominantly hallucinatory
Aurum only	1972201000006102	1972201000006118	1972201000006118			dementia in alzheimer's disease with late onset, other symptoms, predominantly depressive
Aurum only	1972211000006104	1972211000006115	1972211000006115			dementia in alzheimer's disease with late onset, other mixed symptoms
Aurum only	1972231000006105	1972231000006114	1972231000006114			dementia in alzheimer's dis, atypical or mixed type, without additional symptoms
Aurum only	1972251000006103	1972251000006119	1972251000006119			dementia in alzheimer's dis, atypical or mixed type, other symptoms, predominantly delusional
Aurum only	1972291000006109	1972291000006113	1972291000006113			dementia in alzheimer's dis, atypical or mixed type, other symptoms, predominantly hallucinatory
Aurum only	1972311000006108	1972311000006112	1972311000006112			dementia in alzheimer's dis, atypical or mixed type, other symptoms, predominantly depressive

Aurum only	1972341000006107	1972341000006111	1972341000006111			dementia in alzheimer's dis, atypical or mixed type, other mixed symptoms
Aurum only	1972371000006104	1972371000006115	1972371000006115			dementia in alzheimer's disease, unspecified, without additional symptoms
Aurum only	1972401000006101	1972401000006117	1972401000006117			dementia in alzheimer's disease, unspecified, other symptoms, predominantly delusional
Aurum only	1972421000006106	1972421000006110	1972421000006110			dementia in alzheimer's disease, unspecified, other symptoms, predominantly hallucinatory
Aurum only	1972451000006102	1972451000006118	1972451000006118			dementia in alzheimer's disease, unspecified, other symptoms, predominantly depressive
Aurum only	1972471000006107	1972471000006111	1972471000006111			dementia in alzheimer's disease, unspecified, other mixed symptoms
Aurum only	1972481000006105	1972481000006114	1972481000006114			vascular dementia of acute onset, without additional symptoms
Aurum only	1972501000006100	1972501000006116	1972501000006116			vascular dementia of acute onset, other symptoms, predominantly delusional
Aurum only	1972521000006105	1972521000006114	1972521000006114			vascular dementia of acute onset, other symptoms, predominantly hallucinatory
Aurum only	1972541000006103	1972541000006119	1972541000006119			vascular dementia of acute onset, other symptoms, predominantly depressive
Aurum only	1972571000006106	1972571000006110	1972571000006110			vascular dementia of acute onset, other mixed symptoms
Aurum only	1972601000006104	1972601000006115	1972601000006115			multi-infarct dementia, without additional symptoms
Aurum only	1972621000006109	1972621000006113	1972621000006113			multi-infarct dementia, other symptoms, predominantly delusional
Aurum only	1972641000006102	1972641000006118	1972641000006118			multi-infarct dementia, other symptoms, predominantly hallucinatory
Aurum only	1972661000006103	1972661000006119	1972661000006119			multi-infarct dementia, other symptoms, predominantly depressive
Aurum only	1972681000006108	1972681000006112	1972681000006112			multi-infarct dementia, other mixed symptoms

Aurum only	1972711000006109	1972711000006113	1972711000006113			subcortical vascular dementia, without additional symptoms
Aurum only	1972731000006103	1972731000006119	1972731000006119			subcortical vascular dementia, other symptoms, predominantly delusional
Aurum only	1972751000006105	1972751000006114	1972751000006114			subcortical vascular dementia, other symptoms, predominantly hallucinatory
Aurum only	1972771000006100	1972771000006116	1972771000006116			subcortical vascular dementia, other symptoms, predominantly depressive
Aurum only	1972791000006104	1972791000006115	1972791000006115			subcortical vascular dementia, other mixed symptoms
Aurum only	1972821000006108	1972821000006112	1972821000006112			mixed cortical and subcortical vascular dementia, without additional symptoms
Aurum only	1972831000006106	1972831000006110	1972831000006110			mixed cortical and subcortical vascular dementia, other symptoms, predominantly delusional
Aurum only	1972871000006109	1972871000006113	1972871000006113			mixed cortical and subcortical vascular dementia, other symptoms, predominantly hallucinatory
Aurum only	1972911000006107	1972911000006111	1972911000006111			mixed cortical and subcortical vascular dementia, other symptoms, predominantly depressive
Aurum only	1972931000006101	1972931000006117	1972931000006117			mixed cortical and subcortical vascular dementia, other mixed symptoms
Aurum only	1973221000006102	1973221000006118	1973221000006118			other vascular dementia, without additional symptoms
Aurum only	1973271000006101	1973271000006117	1973271000006117			other vascular dementia, other symptoms, predominantly delusional
Aurum only	1973341000006102	1973341000006118	1973341000006118			other vascular dementia, other symptoms, predominantly hallucinatory
Aurum only	1973381000006108	1973381000006112	1973381000006112			other vascular dementia, other symptoms, predominantly depressive
Aurum only	1973401000006108	1973401000006112	1973401000006112			other vascular dementia, other mixed symptoms
Aurum only	1973461000006109	1973461000006113	1973461000006113			vascular dementia, unspecified, without additional symptoms

Aurum only	1973501000006109	1973501000006113	1973501000006113			vascular dementia, unspecified, other symptoms, predominantly delusional
Aurum only	1973531000006101	1973531000006117	1973531000006117			vascular dementia, unspecified, other symptoms, predominantly hallucinatory
Aurum only	1973551000006108	1973551000006112	1973551000006112			vascular dementia, unspecified, other symptoms, predominantly depressive
Aurum only	1976831000006107	1976831000006111	1976831000006111			vascular dementia, unspecified, other mixed symptoms
Aurum and GOLD	281004	1225776018	346929012	27342	E012.11	alcoholic dementia nos
Aurum and GOLD	281004	1225776018	401760017	54505	E012.00	other alcoholic dementia
Aurum and GOLD	281004	1225776018	362941000006113	26323	Eu10711	[x]alcoholic dementia nos
Aurum and GOLD	2776000	5694012	295714013	53446	Eu04100	[x]delirium superimposed on dementia
Aurum and GOLD	12348006	21256010	21256010	15165	E001.00	presenile dementia
Aurum and GOLD	12348006	21256010	294643015	38438	E001z00	presenile dementia nos
Aurum and GOLD	12348006	21256010	359081000006118	48501	Eu02z11	[x] presenile dementia nos
Aurum and GOLD	13092008	22408016	22408016	11136	F111.00	pick's disease
Aurum and GOLD	15662003	26545010	26545010	1916	E00..11	senile dementia
Aurum and GOLD	15662003	26545010	359141000006111	4357	Eu02z14	[x] senile dementia nos
Aurum and GOLD	26929004	45046017	45046017	1917	F110.00	alzheimer's disease
Aurum and GOLD	26929004	45046017	295668011	7664	Eu00.00	[x]dementia in alzheimer's disease
Aurum and GOLD	26929004	45046017	295671015	30706	Eu00200	[x]dementia in alzheimer's dis, atypical or mixed type
Aurum and GOLD	26929004	45046017	295672010	29386	Eu00z00	[x]dementia in alzheimer's disease, unspecified
Aurum and GOLD	26929004	45046017	299325013	59122	Fyu3000	[x]other alzheimer's disease
Aurum and GOLD	26929004	45046017	363021000006113	8195	Eu00z11	[x]alzheimer's dementia unspec
Aurum and GOLD	45864009	76484016	76484016	29512	F112.00	senile degeneration of brain
Aurum and GOLD	52448006	87274019	148381000006115	1350	E00..12	senile/presenile dementia
Aurum and GOLD	52448006	87274019	359241000006119	4693	Eu02z00	[x] unspecified dementia
Aurum and GOLD	56267009	93568017	399031000006111	11175	Eu01100	[x]multi-infarct dementia
Aurum and GOLD	56267009	93568017	423221000006117	55838	Eu01111	[x]predominantly cortical dementia
Aurum and GOLD	56267009	93568017	696161000006115	8634	E004.11	multi infarct dementia
Aurum and GOLD	56267009	497559016	294656010	42279	E004z00	arteriosclerotic dementia nos

Aurum and GOLD	56267009	497559016	497559016	19477	E004.00	arteriosclerotic dementia
Aurum and GOLD	56267009	497559016	363791000006112	9565	Eu01.11	[x]arteriosclerotic dementia
Aurum and GOLD	80098002	132893017	299641000000112	26270	Eu02500	[x]lewy body dementia
Aurum and GOLD	80098002	132893017	745381000006119	7572	F116.00	lewy body disease
Aurum and GOLD	90099008	149347010	149347010	5095	F21y200	binswanger's disease
Aurum and GOLD	90099008	1235534016	1235534016	68194	F21y211	binswanger's encephalopathy
Aurum and GOLD	161465002	251625013	251625013	5931	1461.00	h/o: dementia
Aurum and GOLD	191449005	294635013	294635013	7323	E000.00	uncomplicated senile dementia
Aurum and GOLD	191451009	294637017	294637017	42602	E001000	uncomplicated presenile dementia
Aurum and GOLD	191452002	294638010	294638010	49513	E001100	presenile dementia with delirium
Aurum and GOLD	191454001	294641018	294641018	30032	E001200	presenile dementia with paranoia
Aurum and GOLD	191455000	294642013	294642013	27677	E001300	presenile dementia with depression
Aurum and GOLD	191457008	294644014	294644014	44674	E002.00	senile dementia with depressive or paranoid features
Aurum and GOLD	191457008	294644014	294647019	41089	E002z00	senile dementia with depressive or paranoid features nos
Aurum and GOLD	191457008	294644014	359151000006113	27759	Eu02z16	[x] senile dementia, depressed or paranoid type
Aurum and GOLD	191458003	294645010	294645010	18386	E002000	senile dementia with paranoia
Aurum and GOLD	191459006	294646011	294646011	21887	E002100	senile dementia with depression
Aurum and GOLD	191461002	294648012	294648012	37015	E003.00	senile dementia with delirium
Aurum and GOLD	191463004	294652012	294652012	43089	E004000	uncomplicated arteriosclerotic dementia
Aurum and GOLD	191464005	294653019	294653019	56912	E004100	arteriosclerotic dementia with delirium
Aurum and GOLD	191465006	294654013	294654013	55467	E004200	arteriosclerotic dementia with paranoia
Aurum and GOLD	191466007	294655014	294655014	43292	E004300	arteriosclerotic dementia with depression
Aurum and GOLD	191519005	294718018	294718018	25386	E041.00	dementia in conditions ec
Aurum and GOLD	191519005	2547722017	295684012	12621	Eu02.00	[x]dementia in other diseases classified elsewhere
Aurum and GOLD	191519005	2547722017	295690011	64267	Eu02y00	[x]dementia in other specified diseases classif elsewhere
Aurum and GOLD	230285003	345110014	431691000006119	46488	Eu01000	[x]vascular dementia of acute onset
Aurum and GOLD	230286002	345111013	428201000006119	8934	Eu01200	[x]subcortical vascular dementia

Aurum and GOLD	230287006	345112018	398571000006112	31016	Eu01300	[x]mixed cortical and subcortical vascular dementia
Aurum and GOLD	268612007	401757012	401757012	33707	E00..00	senile and presenile organic psychotic conditions
Aurum and GOLD	279982005	417465012	359101000006114	34944	Eu02z13	[x] primary degenerative dementia nos
Aurum and GOLD	416780008	2957119019	363041000006118	61528	Eu00013	[x]alzheimer's disease type 2
Aurum and GOLD	416780008	2957119019	376531000006119	49263	Eu00000	[x]dementia in alzheimer's disease with early onset
Aurum and GOLD	416780008	2957119019	423391000006114	60059	Eu00012	[x]primary degen dementia, alzheimer's type, presenile onset
Aurum and GOLD	416780008	2957138011	423351000006115	25704	Eu00011	[x]presenile dementia,alzheimer's type
Aurum and GOLD	416780008	2957141019	499946014	16797	F110000	alzheimer's disease with early onset
Aurum and GOLD	416975007	2957124016	500317011	32057	F110100	alzheimer's disease with late onset
Aurum and GOLD	416975007	2957124016	425901000006116	11379	Eu00112	[x]senile dementia,alzheimer's type
Aurum and GOLD	416975007	2957137018	363031000006111	46762	Eu00111	[x]alzheimer's disease type 1
Aurum and GOLD	416975007	2957137018	376541000006112	38678	Eu00100	[x]dementia in alzheimer's disease with late onset
Aurum and GOLD	416975007	2957137018	423381000006111	43346	Eu00113	[x]primary degen dementia of alzheimer's type, senile onset
Aurum and GOLD	421529006	2622767013	376571000006116	41185	Eu02400	[x]dementia in human immunodef virus [hiv] disease
Aurum and GOLD	425390006	2921040013	295688010	9509	Eu02300	[x]dementia in parkinson's disease
Aurum and GOLD	429458009	2695885013	295686014	54106	Eu02100	[x]dementia in creutzfeldt-jakob disease
Aurum and GOLD	429998004	2770951017	295680015	55313	Eu01y00	[x]other vascular dementia
Aurum and GOLD	429998004	2770951017	295681016	19393	Eu01z00	[x]vascular dementia, unspecified
Aurum and GOLD	429998004	2770951017	431681000006117	6578	Eu01.00	[x]vascular dementia
Aurum and GOLD	442344002	2820374011	295687017	37014	Eu02200	[x]dementia in huntington's disease
Aurum and GOLD	700214004	2989656010	2290431000000110	109047	8BPa.00	antipsychotic drug therapy for dementia
Aurum and GOLD	21921000119103	2968126015	295685013	28402	Eu02000	[x]dementia in pick's disease

Code list for antipsychotics – Aurum

prodcodeid	term	drug substance	class
145141000033119	Benperidol 250microgram tablets	Benperidol	FirstGeneration
69041000033115	Anquil 250microgram tablets	Benperidol	FirstGeneration
11876741000033116	Chlorpromazine 25mg/5ml oral solution sugar free	Chlorpromazine	FirstGeneration
1719341000033115	Chlorpromazine 100mg/5ml oral solution	Chlorpromazine	FirstGeneration
240041000033110	Chlorpromazine 25mg/5ml oral solution	Chlorpromazine	FirstGeneration
241341000033116	Chlorpromazine 50mg/2ml solution	Chlorpromazine	FirstGeneration
242341000033113	Chlorpromazine Hydrochloride Injection 25 mg/ml	Chlorpromazine	FirstGeneration
246641000033118	Chlorpromazine 100mg/5ml oral suspension sugar free	Chlorpromazine	FirstGeneration
249041000033117	Chlorpromazine 10mg tablets	Chlorpromazine	FirstGeneration
249141000033118	Chlorpromazine 100mg tablets	Chlorpromazine	FirstGeneration
249241000033113	Chlorpromazine 25mg tablets	Chlorpromazine	FirstGeneration
249341000033115	Chlorpromazine 50mg tablets	Chlorpromazine	FirstGeneration
251141000033117	Chloractil Tablets 100 mg	Chlorpromazine	FirstGeneration
251341000033119	Chloractil Tablets 50 mg	Chlorpromazine	FirstGeneration
3849441000033112	Chlorpromazine 50mg/5ml oral solution	Chlorpromazine	FirstGeneration
4148441000033110	Chlorpromazine Capsules 10 mg	Chlorpromazine	FirstGeneration
811041000033116	Largactil 50mg/2ml solution	Chlorpromazine	FirstGeneration
813741000033119	Largactil 25mg/5ml syrup	Chlorpromazine	FirstGeneration
815741000033115	Largactil 10mg tablets	Chlorpromazine	FirstGeneration
815841000033113	Largactil 100mg tablets	Chlorpromazine	FirstGeneration
815941000033117	Largactil 25mg tablets	Chlorpromazine	FirstGeneration
816041000033110	Largactil 50mg tablets	Chlorpromazine	FirstGeneration
483541000033115	Droperidol Liquid 1mg/ml	Droperidol	FirstGeneration
485041000033112	Droperidol Tablets 10 mg	Droperidol	FirstGeneration
3090541000033111	Flupentixol 50mg/0.5ml solution	Flupentixol	FirstGeneration
3090641000033112	Flupentixol 100mg/1ml solution	Flupentixol	FirstGeneration
3090741000033115	Flupentixol 200mg/1ml solution	Flupentixol	FirstGeneration
3090841000033113	Flupentixol 20mg/1ml solution	Flupentixol	FirstGeneration

3090941000033117	Flupentixol 40mg/2ml solution	Flupentixol	FirstGeneration
3091041000033110	Flupentixol 1mg tablets	Flupentixol	FirstGeneration
3091141000033114	Flupentixol 500microgram tablets	Flupentixol	FirstGeneration
3091241000033119	Flupentixol 3mg tablets	Flupentixol	FirstGeneration
419941000033113	Depixol Low Volume 200mg/1ml solution	Flupentixol	FirstGeneration
420041000033111	Depixol Injection 200 mg/10 ml	Flupentixol	FirstGeneration
420141000033110	Depixol 40mg/2ml solution	Flupentixol	FirstGeneration
420241000033115	Depixol Injection	Flupentixol	FirstGeneration
420441000033119	Depixol Conc 50mg/0.5ml solution	Flupentixol	FirstGeneration
422941000033113	Depixol 20mg/1ml solution	Flupentixol	FirstGeneration
423041000033115	Depixol Conc 100mg/1ml solution	Flupentixol	FirstGeneration
431741000033118	Depixol 3mg tablets	Flupentixol	FirstGeneration
593441000033111	Fluanxol 1mg tablets	Flupentixol	FirstGeneration
593541000033112	Fluanxol 500microgram tablets	Flupentixol	FirstGeneration
3179941000033111	Nortriptyline 10mg / Fluphenazine 500microgram tablets	Fluphenazine	FirstGeneration
584641000033111	Fluphenazine decanoate 25mg/1ml solution	Fluphenazine	FirstGeneration
586641000033119	Fluphenazine decanoate 12.5mg/0.5ml solution	Fluphenazine	FirstGeneration
587741000033113	Fluphenazine decanoate 100mg/1ml solution	Fluphenazine	FirstGeneration
595441000033112	Fluphenazine 1mg tablets	Fluphenazine	FirstGeneration
595641000033114	Fluphenazine 2.5mg tablets	Fluphenazine	FirstGeneration
925841000033113	Modecate 12.5mg/0.5ml solution	Fluphenazine	FirstGeneration
925941000033117	Modecate Concentrate 100mg/1ml solution	Fluphenazine	FirstGeneration
934841000033111	Modecate 25mg/1ml solution	Fluphenazine	FirstGeneration
935041000033115	Moditen 1mg tablets	Fluphenazine	FirstGeneration
938841000033118	Motipress tablets	Fluphenazine	FirstGeneration
938941000033114	Motival 10mg/500microgram tablets	Fluphenazine	FirstGeneration
10493341000033115	Haloperidol 500micrograms/5ml oral solution	Haloperidol	FirstGeneration
1268341000033116	Serenace 500microgram capsules	Haloperidol	FirstGeneration
1269641000033115	Serenace Elixir 2 mg/5 ml	Haloperidol	FirstGeneration
1269741000033112	Serenace 2mg/ml liquid	Haloperidol	FirstGeneration
1271641000033113	Serenace 5mg/1ml solution	Haloperidol	FirstGeneration
1275941000033115	Serenace 1.5mg tablets	Haloperidol	FirstGeneration

1276041000033113	Serenace 10mg tablets	Haloperidol	FirstGeneration
1276241000033117	Serenace 5mg tablets	Haloperidol	FirstGeneration
2758141000033116	Haloperidol Oral Solution, Sugar Free 1 mg/5 ml	Haloperidol	FirstGeneration
2929741000033118	Haloperidol 5mg/1ml solution	Haloperidol	FirstGeneration
2929841000033111	Haldol 5mg/1ml solution	Haloperidol	FirstGeneration
4424541000033119	Haloperidol 5mg/5ml oral solution	Haloperidol	FirstGeneration
476541000033119	Dozic 5mg/5ml oral solution	Haloperidol	FirstGeneration
5054641000033118	Haloperidol 1mg/5ml oral solution	Haloperidol	FirstGeneration
5890741000033111	Haloperidol 1mg/5ml oral suspension	Haloperidol	FirstGeneration
5969641000033115	Haloperidol 250micrograms/5ml oral solution	Haloperidol	FirstGeneration
5969741000033112	Haloperidol 250micrograms/5ml oral suspension	Haloperidol	FirstGeneration
658341000033110	Haloperidol 500microgram capsules	Haloperidol	FirstGeneration
659241000033113	Haloperidol Elixir 2 mg/5 ml	Haloperidol	FirstGeneration
659441000033114	Haldol decanoate 100mg/1ml solution	Haloperidol	FirstGeneration
659541000033110	Haldol decanoate 50mg/1ml solution	Haloperidol	FirstGeneration
659641000033111	Haloperidol 20mg/2ml solution	Haloperidol	FirstGeneration
659741000033119	Haloperidol decanoate 100mg/1ml solution	Haloperidol	FirstGeneration
659841000033112	Haloperidol decanoate 50mg/1ml solution	Haloperidol	FirstGeneration
659941000033116	Haloperidol Injection 5 mg/ml	Haloperidol	FirstGeneration
660041000033118	Haldol Injection 5 mg/ml	Haloperidol	FirstGeneration
660341000033116	Haloperidol 5mg/5ml oral solution sugar free	Haloperidol	FirstGeneration
660441000033110	Haloperidol Liquid 2mg/ml	Haloperidol	FirstGeneration
661341000033112	Haloperidol 10mg/5ml oral solution sugar free	Haloperidol	FirstGeneration
661541000033117	Haldol 2mg/ml oral solution	Haloperidol	FirstGeneration
662341000033115	Haloperidol 1.5mg tablets	Haloperidol	FirstGeneration
662441000033114	Haloperidol 20mg tablets	Haloperidol	FirstGeneration
662541000033110	Haloperidol 5mg tablets	Haloperidol	FirstGeneration
663241000033118	Haloperidol 10mg tablets	Haloperidol	FirstGeneration
663341000033111	Haloperidol 500microgram tablets	Haloperidol	FirstGeneration
663541000033116	Haldol 5mg tablets	Haloperidol	FirstGeneration
1512741000033113	Nozinan 25mg tablets	Levomepromazine	FirstGeneration
3063441000033119	Levomepromazine 25mg/1ml solution	Levomepromazine	FirstGeneration

3063541000033118	Levomepromazine 25mg tablets	Levomepromazine	FirstGeneration
3063641000033117	Levomepromazine 6mg tablets	Levomepromazine	FirstGeneration
5814341000033116	Levomepromazine 2.5mg/5ml oral suspension	Levomepromazine	FirstGeneration
6445641000033110	Levomepromazine 6mg/5ml oral suspension	Levomepromazine	FirstGeneration
981641000033117	Nozinan 25mg/1ml solution	Levomepromazine	FirstGeneration
1026941000033117	Oxypertine 40mg tablets	Oxypertine	FirstGeneration
1063541000033113	Pericyazine 10mg/5ml oral solution	Pericyazine	FirstGeneration
1066741000033111	Pericyazine 10mg tablets	Pericyazine	FirstGeneration
1067041000033110	Pericyazine 2.5mg tablets	Pericyazine	FirstGeneration
965041000033112	Neulactil Forte syrup	Pericyazine	FirstGeneration
965541000033119	Neulactil 10mg tablets	Pericyazine	FirstGeneration
965641000033118	Neulactil 2.5mg tablets	Pericyazine	FirstGeneration
1065141000033117	Perphenazine 2mg tablets	Perphenazine	FirstGeneration
1065241000033112	Perphenazine 4mg tablets	Perphenazine	FirstGeneration
1473341000033117	Triptafen tablets	Perphenazine	FirstGeneration
1473441000033111	Triptafen-M tablets	Perphenazine	FirstGeneration
3180441000033118	Amitriptyline 25mg / Perphenazine 2mg tablets	Perphenazine	FirstGeneration
3180541000033117	Amitriptyline 10mg / Perphenazine 2mg tablets	Perphenazine	FirstGeneration
570941000033117	Fentazin 2mg tablets	Perphenazine	FirstGeneration
571041000033110	Fentazin 4mg tablets	Perphenazine	FirstGeneration
1016441000033117	Orap 2mg tablets	Pimozide	FirstGeneration
1016541000033116	Orap 4mg tablets	Pimozide	FirstGeneration
1090841000033118	Pimozide Tablets 10 mg	Pimozide	FirstGeneration
1090941000033114	Pimozide 2mg tablets	Pimozide	FirstGeneration
1091041000033116	Pimozide 4mg tablets	Pimozide	FirstGeneration
1086741000033114	Piportil Depot 50mg/1ml solution	Pipotiazine	FirstGeneration
3103841000033110	Pipotiazine 100mg/2ml solution	Pipotiazine	FirstGeneration
3104041000033117	Pipotiazine 50mg/1ml solution	Pipotiazine	FirstGeneration
1117541000033112	Promazine Hydrochloride Injection 50 mg/ml	Promazine	FirstGeneration
1129141000033118	Promazine Hydrochloride Suspension 50 mg/5 ml	Promazine	FirstGeneration
1129641000033111	Promazine 25mg/5ml oral solution	Promazine	FirstGeneration
1134241000033114	Promazine Hydrochloride Tablets 100 mg	Promazine	FirstGeneration

1134341000033116	Promazine 25mg tablets	Promazine	FirstGeneration
1134441000033110	Promazine 50mg tablets	Promazine	FirstGeneration
1370441000033112	Sparine Injection 50 mg/ml	Promazine	FirstGeneration
1372741000033116	Sparine Suspension 50 mg/5 ml	Promazine	FirstGeneration
2640141000033112	Promazine 50mg/5ml oral solution	Promazine	FirstGeneration
2730641000033111	Promazine Hydrochloride Syrup 50 mg/5 ml	Promazine	FirstGeneration
1396441000033117	Sulpiride 200mg tablets	Sulpiride	FirstGeneration
1397641000033117	Sulpiride 400mg tablets	Sulpiride	FirstGeneration
1698441000033110	Sulpiride 200mg/5ml oral solution sugar free	Sulpiride	FirstGeneration
2658941000033110	Sulpor 200mg/5ml oral solution	Sulpiride	FirstGeneration
480941000033116	Dolmatil 200mg tablets	Sulpiride	FirstGeneration
1432341000033111	Thioridazine 100mg/5ml oral suspension	Thioridazine	FirstGeneration
1432441000033117	Thioridazine 25mg/5ml oral suspension	Thioridazine	FirstGeneration
1432541000033116	Thioridazine 25mg/5ml oral solution	Thioridazine	FirstGeneration
1432741000033112	Thioridazine Syrup 12.5mg/5 ml	Thioridazine	FirstGeneration
1433641000033111	Thioridazine 10mg tablets	Thioridazine	FirstGeneration
1433741000033119	Thioridazine 100mg tablets	Thioridazine	FirstGeneration
1433841000033112	Thioridazine 25mg tablets	Thioridazine	FirstGeneration
1433941000033116	Thioridazine 50mg tablets	Thioridazine	FirstGeneration
892241000033119	Melleril 25mg/5ml oral suspension	Thioridazine	FirstGeneration
893741000033115	Melleril 25mg/5ml syrup	Thioridazine	FirstGeneration
895841000033114	Melleril 10mg tablets	Thioridazine	FirstGeneration
895941000033118	Melleril 100mg tablets	Thioridazine	FirstGeneration
896041000033111	Melleril 25mg tablets	Thioridazine	FirstGeneration
896141000033110	Melleril 50mg tablets	Thioridazine	FirstGeneration
1385341000033116	Stelazine 10mg Spansules	Trifluoperazine	FirstGeneration
1385441000033110	Stelazine 15mg Spansules	Trifluoperazine	FirstGeneration
1385541000033111	Stelazine 2mg Spansules	Trifluoperazine	FirstGeneration
1385941000033117	Stelazine 1mg/5ml syrup	Trifluoperazine	FirstGeneration
1386341000033112	Stelazine 1mg tablets	Trifluoperazine	FirstGeneration
1386441000033118	Stelazine 5mg tablets	Trifluoperazine	FirstGeneration
1464141000033113	Trifluoperazine 5mg/5ml oral solution sugar free	Trifluoperazine	FirstGeneration

1466941000033117	Trifluoperazine 10mg modified-release capsules	Trifluoperazine	FirstGeneration
1467041000033116	Trifluoperazine 15mg modified-release capsules	Trifluoperazine	FirstGeneration
1467141000033117	Trifluoperazine 2mg modified-release capsules	Trifluoperazine	FirstGeneration
1467641000033110	Trifluoperazine 1mg/5ml oral solution sugar free	Trifluoperazine	FirstGeneration
1470641000033116	Trifluoperazine 1mg tablets	Trifluoperazine	FirstGeneration
1470741000033113	Trifluoperazine 5mg tablets	Trifluoperazine	FirstGeneration
1474541000033114	Trifluperidol Tablets 500 mcg	Trifluperidol	FirstGeneration
1563441000033114	Zuclopenthixol acetate 100mg/2ml solution	Zuclopenthixol	FirstGeneration
1563541000033110	Zuclopenthixol acetate 50mg/1ml solution	Zuclopenthixol	FirstGeneration
1563641000033111	Zuclopenthixol decanoate 200mg/1ml solution	Zuclopenthixol	FirstGeneration
1563741000033119	Zuclopenthixol decanoate 500mg/1ml solution	Zuclopenthixol	FirstGeneration
1564041000033119	Zuclopenthixol 10mg tablets	Zuclopenthixol	FirstGeneration
1564141000033115	Zuclopenthixol 2mg tablets	Zuclopenthixol	FirstGeneration
276041000033113	Clopixol 200mg/1ml solution	Zuclopenthixol	FirstGeneration
276141000033112	Clopixol Conc 500mg/1ml solution	Zuclopenthixol	FirstGeneration
286941000033115	Clopixol 10mg tablets	Zuclopenthixol	FirstGeneration
287041000033119	Clopixol 2mg tablets	Zuclopenthixol	FirstGeneration
1366741000033110	Solian 50 tablets	Amisulpride	SecondGeneration
2216641000033113	Amisulpride 400mg tablets	Amisulpride	SecondGeneration
2644841000033114	Amisulpride 100mg/ml oral solution sugar free	Amisulpride	SecondGeneration
2644941000033118	Solian 100mg/ml oral solution	Amisulpride	SecondGeneration
2835141000033114	Amisulpride 100mg tablets	Amisulpride	SecondGeneration
4940141000033119	Amisulpride 25mg/5ml oral solution	Amisulpride	SecondGeneration
5890941000033114	Amisulpride 25mg/5ml oral suspension	Amisulpride	SecondGeneration
58941000033117	Amisulpride 200mg tablets	Amisulpride	SecondGeneration
59041000033114	Amisulpride 50mg tablets	Amisulpride	SecondGeneration
3137541000033113	Aripiprazole 10mg tablets	Aripiprazole	SecondGeneration
3137641000033114	Aripiprazole 15mg tablets	Aripiprazole	SecondGeneration
3137741000033117	Aripiprazole 30mg tablets	Aripiprazole	SecondGeneration
3137941000033119	Abilify 15mg tablets	Aripiprazole	SecondGeneration
3225241000033118	Aripiprazole 5mg tablets	Aripiprazole	SecondGeneration
3225341000033111	Abilify 5mg tablets	Aripiprazole	SecondGeneration

3950341000033115	Aripiprazole 10mg orodispersible tablets sugar free	Aripiprazole	SecondGeneration
3950441000033114	Aripiprazole 15mg orodispersible tablets sugar free	Aripiprazole	SecondGeneration
3950541000033110	Aripiprazole 1mg/ml oral solution	Aripiprazole	SecondGeneration
3950841000033112	Abilify 1mg/ml oral solution	Aripiprazole	SecondGeneration
284941000033113	Clozaril 25mg tablets	Clozapine	SecondGeneration
285041000033113	Clozaril 100mg tablets	Clozapine	SecondGeneration
287541000033112	Clozapine 100mg tablets	Clozapine	SecondGeneration
287641000033113	Clozapine 25mg tablets	Clozapine	SecondGeneration
5078041000033117	Clozapine 50mg tablets	Clozapine	SecondGeneration
1003641000033113	Olanzapine 10mg tablets	Olanzapine	SecondGeneration
1003741000033116	Olanzapine 5mg tablets	Olanzapine	SecondGeneration
1003841000033114	Olanzapine 7.5mg tablets	Olanzapine	SecondGeneration
1565141000033119	Zyprexa 10mg tablets	Olanzapine	SecondGeneration
1565241000033114	Zyprexa 5mg tablets	Olanzapine	SecondGeneration
1565341000033116	Zyprexa 7.5mg tablets	Olanzapine	SecondGeneration
1661141000033114	Olanzapine 2.5mg tablets	Olanzapine	SecondGeneration
1713941000033118	Zyprexa 2.5mg tablets	Olanzapine	SecondGeneration
2077741000033115	Olanzapine 5mg oral lyophilisates sugar free	Olanzapine	SecondGeneration
2077841000033113	Olanzapine 10mg oral lyophilisates sugar free	Olanzapine	SecondGeneration
2077941000033117	Zyprexa 5mg Velotabs	Olanzapine	SecondGeneration
2759641000033117	Olanzapine 15mg tablets	Olanzapine	SecondGeneration
2759741000033114	Olanzapine 15mg oral lyophilisates sugar free	Olanzapine	SecondGeneration
2759941000033112	Zyprexa 15mg Velotabs	Olanzapine	SecondGeneration
3866541000033110	Olanzapine 20mg oral lyophilisates sugar free	Olanzapine	SecondGeneration
3892541000033114	Olanzapine 2.5mg/5ml oral suspension	Olanzapine	SecondGeneration
3996241000033117	Olanzapine 20mg tablets	Olanzapine	SecondGeneration
5997341000033118	Olanzapine 2.5mg/5ml oral solution	Olanzapine	SecondGeneration
6465941000033113	Olanzapine 5mg orodispersible tablets sugar free	Olanzapine	SecondGeneration
6466041000033115	Olanzapine 10mg orodispersible tablets sugar free	Olanzapine	SecondGeneration
6466141000033116	Olanzapine 15mg orodispersible tablets sugar free	Olanzapine	SecondGeneration
7681641000033116	Olanzapine 10mg orodispersible tablets	Olanzapine	SecondGeneration
7681941000033111	Olanzapine 5mg orodispersible tablets	Olanzapine	SecondGeneration

6359441000033116	Paliperidone 50mg/0.5ml suspension	Paliperidone	SecondGeneration
6359741000033111	Paliperidone 150mg/1.5ml suspension	Paliperidone	SecondGeneration
10041141000033118	Biquelle XL 50mg tablets	Quetiapine	SecondGeneration
10041241000033113	Biquelle XL 150mg tablets	Quetiapine	SecondGeneration
10041541000033110	Biquelle XL 400mg tablets	Quetiapine	SecondGeneration
10041741000033119	Zaluron XL 50mg tablets	Quetiapine	SecondGeneration
10041941000033116	Zaluron XL 200mg tablets	Quetiapine	SecondGeneration
1150041000033111	Quetiapine 100mg tablets	Quetiapine	SecondGeneration
1150141000033110	Quetiapine 200mg tablets	Quetiapine	SecondGeneration
1150241000033115	Quetiapine 25mg tablets	Quetiapine	SecondGeneration
1150341000033113	Quetiapine Fumarate Tablets	Quetiapine	SecondGeneration
11753641000033118	Quetiapine 20mg/ml oral suspension sugar free	Quetiapine	SecondGeneration
1277641000033116	Seroquel 100mg tablets	Quetiapine	SecondGeneration
1277841000033115	Seroquel 25mg tablets	Quetiapine	SecondGeneration
1832841000033113	Quetiapine Fumarate Tablets	Quetiapine	SecondGeneration
1844641000033118	Quetiapine 150mg tablets	Quetiapine	SecondGeneration
2975841000033113	Quetiapine 300mg tablets	Quetiapine	SecondGeneration
4133841000033118	Quetiapine Oral Suspension Sugar Free 12.5 mg/5 ml	Quetiapine	SecondGeneration
4422541000033110	Quetiapine 100mg/5ml oral suspension	Quetiapine	SecondGeneration
4591741000033114	Quetiapine 50mg modified-release tablets	Quetiapine	SecondGeneration
4591841000033116	Quetiapine 200mg modified-release tablets	Quetiapine	SecondGeneration
4591941000033112	Quetiapine 300mg modified-release tablets	Quetiapine	SecondGeneration
4592041000033118	Quetiapine 400mg modified-release tablets	Quetiapine	SecondGeneration
4592141000033119	Seroquel XL 50mg tablets	Quetiapine	SecondGeneration
5889741000033118	Quetiapine 12.5mg/5ml oral solution	Quetiapine	SecondGeneration
5889841000033111	Quetiapine 12.5mg/5ml oral suspension	Quetiapine	SecondGeneration
5891141000033117	Quetiapine 100mg/5ml oral solution	Quetiapine	SecondGeneration
5909341000033112	Quetiapine 150mg modified-release tablets	Quetiapine	SecondGeneration
5909441000033118	Seroquel XL 150mg tablets	Quetiapine	SecondGeneration
5974841000033112	Quetiapine 25mg/5ml oral solution	Quetiapine	SecondGeneration
5974941000033116	Quetiapine 25mg/5ml oral suspension	Quetiapine	SecondGeneration
5975041000033116	Quetiapine 50mg/5ml oral solution	Quetiapine	SecondGeneration

7687841000033114	Sondate XL 50mg tablets	Quetiapine	SecondGeneration
9604441000033112	Ebesque XL 50mg tablets	Quetiapine	SecondGeneration
1173841000033113	Risperdal 1mg/ml oral solution	Risperidone	SecondGeneration
1173941000033117	Risperidone 1mg/ml oral solution sugar free	Risperidone	SecondGeneration
1176241000033115	Risperdal 1mg tablets	Risperidone	SecondGeneration
1176341000033113	Risperdal 2mg tablets	Risperidone	SecondGeneration
1176441000033119	Risperdal 3mg tablets	Risperidone	SecondGeneration
1176641000033117	Risperidone 1mg tablets	Risperidone	SecondGeneration
1176741000033114	Risperidone 2mg tablets	Risperidone	SecondGeneration
1176841000033116	Risperidone 3mg tablets	Risperidone	SecondGeneration
1176941000033112	Risperidone 4mg tablets	Risperidone	SecondGeneration
1177741000033111	Risperidone 6mg tablets	Risperidone	SecondGeneration
2188141000033115	Risperdal 500microgram tablets	Risperidone	SecondGeneration
2188241000033110	Risperidone 500microgram tablets	Risperidone	SecondGeneration
2779841000033118	Risperdal Consta 25mg powder and solvent	Risperidone	SecondGeneration
2779941000033114	Risperdal Consta 37.5mg powder and solvent	Risperidone	SecondGeneration
2780041000033113	Risperdal Consta 50mg powder and solvent	Risperidone	SecondGeneration
2780241000033117	Risperidone 37.5mg powder and solvent	Risperidone	SecondGeneration
2780341000033110	Risperidone 50mg powder and solvent	Risperidone	SecondGeneration
2868841000033116	Risperidone 1mg orodispersible tablets sugar free	Risperidone	SecondGeneration
2868941000033112	Risperidone 2mg orodispersible tablets sugar free	Risperidone	SecondGeneration
2869041000033115	Risperdal Quicklet 1mg orodispersible tablets	Risperidone	SecondGeneration
2869141000033116	Risperdal Quicklet 2mg orodispersible tablets	Risperidone	SecondGeneration
3246041000033119	Risperidone 500microgram orodispersible tablets sugar free	Risperidone	SecondGeneration
3246141000033115	Risperdal Quicklet 500microgram orodispersible tablets	Risperidone	SecondGeneration
4012041000033116	Risperidone 3mg orodispersible tablets sugar free	Risperidone	SecondGeneration
1766141000033110	Zotepine 25mg tablets	Zotepine	SecondGeneration

Code list for antipsychotics – GOLD

prodcode	productname	drug substance	class
2540	Benperidol 250microgram tablets	Benperidol	FirstGeneration
10434	Largactil 25mg/5ml Oral solution (Hawgreen Ltd)	Chlorpromazine	FirstGeneration
12356	LARGACTIL 50 MG INJ	Chlorpromazine	FirstGeneration
2154	Chlorpromazine 100mg tablets	Chlorpromazine	FirstGeneration
2474	Chlorpromazine 10mg tablets	Chlorpromazine	FirstGeneration
2814	Largactil 25mg Tablet (Hawgreen Ltd)	Chlorpromazine	FirstGeneration
31171	Chlorpromazine 50mg tablets (A A H Pharmaceuticals Ltd)	Chlorpromazine	FirstGeneration
3348	Chlorpromazine 50mg tablets	Chlorpromazine	FirstGeneration
34630	Chlorpromazine 50mg tablets (Thornton & Ross Ltd)	Chlorpromazine	FirstGeneration
3772	Largactil 50mg Tablet (Hawgreen Ltd)	Chlorpromazine	FirstGeneration
3952	Chlorpromazine 25mg/5ml oral solution	Chlorpromazine	FirstGeneration
4434	Chlorpromazine 50mg/5ml oral solution	Chlorpromazine	FirstGeneration
588	Chlorpromazine 25mg tablets	Chlorpromazine	FirstGeneration
7514	Largactil 50mg/2ml solution for injection ampoules (Sanofi)	Chlorpromazine	FirstGeneration
8045	CHLORPROMAZINE 200 MG TAB	Chlorpromazine	FirstGeneration
8311	Chlorpromazine 25mg/ml injection	Chlorpromazine	FirstGeneration
8771	Largactil 10mg Tablet (Hawgreen Ltd)	Chlorpromazine	FirstGeneration
9190	Chlorpromazine 25mg/5ml oral solution sugar free	Chlorpromazine	FirstGeneration
13341	DROPERIDOL 5 MG/5ML ELI	Droperidol	FirstGeneration
15128	Droperidol 1mg/ml liquid	Droperidol	FirstGeneration
3773	Droperidol 10mg tablets	Droperidol	FirstGeneration
14839	Flupentixol 200mg/1ml solution for injection ampoules	Flupentixol	FirstGeneration
14889	Depixol Conc 100mg/1ml solution for injection ampoules (Lundbeck Ltd)	Flupentixol	FirstGeneration
14966	Flupentixol 20mg/1ml solution for injection ampoules	Flupentixol	FirstGeneration
1733	Flupentixol decanoate 20mg/ml Injection	Flupentixol	FirstGeneration
18175	Flupentixol 100mg/1ml solution for injection ampoules	Flupentixol	FirstGeneration
19283	Depixol 20mg/1ml solution for injection ampoules (Lundbeck Ltd)	Flupentixol	FirstGeneration
2136	Depixol 20mg/ml Injection (Lundbeck Ltd)	Flupentixol	FirstGeneration

2156	Depixol 40mg/2ml solution for injection ampoules (Lundbeck Ltd)	Flupentixol	FirstGeneration
2275	Flupentixol 500microgram tablets	Flupentixol	FirstGeneration
2276	Flupentixol 40mg/2ml solution for injection ampoules	Flupentixol	FirstGeneration
3951	Fluanxol 1mg tablets (Lundbeck Ltd)	Flupentixol	FirstGeneration
3953	Fluanxol 500microgram tablets (Lundbeck Ltd)	Flupentixol	FirstGeneration
5707	Flupentixol 3mg tablets	Flupentixol	FirstGeneration
5712	Depixol 3mg tablets (Lundbeck Ltd)	Flupentixol	FirstGeneration
600	Flupentixol 1mg tablets	Flupentixol	FirstGeneration
8712	Flupentixol decanoate 100mg/ml Injection	Flupentixol	FirstGeneration
12128	Modecate concentrate 100mg/ml Injection (Sanofi-Synthelabo Ltd)	Fluphenazine	FirstGeneration
14578	Nortriptyline 30mg / Fluphenazine 1.5mg tablets	Fluphenazine	FirstGeneration
2936	Motival 10mg/500microgram tablets (Sanofi)	Fluphenazine	FirstGeneration
35065	Fluphenazine decanoate 25mg/1ml solution for injection ampoules	Fluphenazine	FirstGeneration
3926	Modecate 25mg/ml Injection (Sanofi-Synthelabo Ltd)	Fluphenazine	FirstGeneration
5212	Fluphenazine 1mg tablets	Fluphenazine	FirstGeneration
7780	Nortriptyline 10mg / Fluphenazine 500microgram tablets	Fluphenazine	FirstGeneration
8043	MODECATE 12.5 MG INJ	Fluphenazine	FirstGeneration
8377	Fluphenazine 2.5mg tablets	Fluphenazine	FirstGeneration
8493	Motipress tablets (Sanofi-Synthelabo Ltd)	Fluphenazine	FirstGeneration
9022	Fluphenazine decanoate 25mg/ml Injection	Fluphenazine	FirstGeneration
10565	Haloperidol decanoate 50mg/1ml solution for injection ampoules	Haloperidol	FirstGeneration
11213	Haloperidol 2mg/5ml sugar free Oral solution	Haloperidol	FirstGeneration
12921	Haldol 2mg/ml oral solution (Janssen-Cilag Ltd)	Haloperidol	FirstGeneration
13105	Haloperidol 2mg/ml Oral solution	Haloperidol	FirstGeneration
2094	Haldol Decanoate 50mg/1ml solution for injection ampoules (Janssen-Cilag Ltd)	Haloperidol	FirstGeneration
22660	Haldol 5mg tablets (Janssen-Cilag Ltd)	Haloperidol	FirstGeneration
2419	Haloperidol 500microgram capsules	Haloperidol	FirstGeneration
2620	Haloperidol 1mg/ml Oral solution	Haloperidol	FirstGeneration
2621	Haloperidol 5mg tablets	Haloperidol	FirstGeneration
3233	Haloperidol 2mg/ml sugar free Liquid	Haloperidol	FirstGeneration
329	Haloperidol 1.5mg tablets	Haloperidol	FirstGeneration
34039	Haloperidol 1mg/ml Liquid (Rosemont Pharmaceuticals Ltd)	Haloperidol	FirstGeneration

3671	Haloperidol 500microgram tablets	Haloperidol	FirstGeneration
36771	Haloperidol 250micrograms/5ml oral suspension	Haloperidol	FirstGeneration
38262	Haloperidol 5mg/1ml solution for injection ampoules	Haloperidol	FirstGeneration
4234	Haloperidol 5mg/ml Injection	Haloperidol	FirstGeneration
45810	Haloperidol 10mg/5ml oral solution sugar free	Haloperidol	FirstGeneration
45880	Haloperidol 5mg/5ml oral solution sugar free	Haloperidol	FirstGeneration
47149	Haloperidol 1mg/5ml oral solution	Haloperidol	FirstGeneration
475	Haloperidol 10mg tablets	Haloperidol	FirstGeneration
5192	Haloperidol 1mg/5ml sugar free Oral solution	Haloperidol	FirstGeneration
5545	Serenace 500microgram capsules (Teva UK Ltd)	Haloperidol	FirstGeneration
8136	HALOPERIDOL 5 MG LIQ	Haloperidol	FirstGeneration
8153	Serenace 2mg/ml liquid (Teva UK Ltd)	Haloperidol	FirstGeneration
8979	Serenace 1.5mg tablets (Teva UK Ltd)	Haloperidol	FirstGeneration
9975	Haloperidol 1mg/ml sugar free Oral solution	Haloperidol	FirstGeneration
40782	Levomepromazine 6mg Tablet	Levomepromazine	FirstGeneration
4232	Nozinan 25mg tablets (Sanofi)	Levomepromazine	FirstGeneration
4442	Nozinan 25mg/1ml solution for injection ampoules (Sanofi)	Levomepromazine	FirstGeneration
5014	Levomepromazine 25mg tablets	Levomepromazine	FirstGeneration
6064	Levomepromazine 25mg/1ml solution for injection ampoules	Levomepromazine	FirstGeneration
7390	Levomepromazine 6mg tablets	Levomepromazine	FirstGeneration
12616	Loxapine 10mg capsules	Loxapine	FirstGeneration
8921	Integrin 10mg Capsule (Sanofi-Synthelabo Ltd)	Oxypertine	FirstGeneration
8922	Oxypertine 10mg capsules	Oxypertine	FirstGeneration
12195	Pericyazine 10mg/5ml oral solution	Pericyazine	FirstGeneration
13902	Neulactil Forte syrup (Sanofi)	Pericyazine	FirstGeneration
7833	Neulactil 2.5mg Tablet (JHC Healthcare Ltd)	Pericyazine	FirstGeneration
7834	Pericyazine 2.5mg tablets	Pericyazine	FirstGeneration
8032	Pericyazine 10mg tablets	Pericyazine	FirstGeneration
1208	Triptafen tablets (AMCo)	Perphenazine	FirstGeneration
1453	Triptafen m 2mg+10mg Tablet (Goldshield Pharmaceuticals Ltd)	Perphenazine	FirstGeneration
16323	Perphenazine 2mg with Amitriptyline 10mg tablet	Perphenazine	FirstGeneration
2157	Perphenazine 4mg tablets	Perphenazine	FirstGeneration

3490	Amitriptyline 10mg / Perphenazine 2mg tablets	Perphenazine	FirstGeneration
595	Amitriptyline 25mg / Perphenazine 2mg tablets	Perphenazine	FirstGeneration
609	Perphenazine 2mg tablets	Perphenazine	FirstGeneration
6894	Perphenazine 2mg with Amitriptyline 25mg tablet	Perphenazine	FirstGeneration
7919	Fentazin 4mg tablets (AMCo)	Perphenazine	FirstGeneration
840	Fentazin 2mg tablets (AMCo)	Perphenazine	FirstGeneration
2489	Pimozide 2mg tablets	Pimozide	FirstGeneration
4524	Orap 2mg tablets (Janssen-Cilag Ltd)	Pimozide	FirstGeneration
8637	Pimozide 10mg tablet	Pimozide	FirstGeneration
35235	Piportil Depot 50mg/1ml solution for injection ampoules (Sanofi)	Pipotiazine	FirstGeneration
10780	Promazine 50mg/5ml oral solution	Promazine	FirstGeneration
13311	Sparine 50mg/ml Injection (Wyeth Pharmaceuticals)	Promazine	FirstGeneration
14610	Promazine 50mg/5ml oral solution sugar free	Promazine	FirstGeneration
15395	Promazine 12.5mg/5ml oral solution	Promazine	FirstGeneration
17634	Promazine 50mg/5ml oral solution	Promazine	FirstGeneration
2972	Promazine 25mg tablets	Promazine	FirstGeneration
3197	Promazine 100mg tablet	Promazine	FirstGeneration
3227	Sparine 50mg/5ml Liquid (Wyeth Pharmaceuticals)	Promazine	FirstGeneration
3228	Promazine 50mg tablets	Promazine	FirstGeneration
40390	Promazine 25mg/5ml syrup (Rosemont Pharmaceuticals Ltd)	Promazine	FirstGeneration
46945	Promazine 25mg Tablet (Biorex Laboratories Ltd)	Promazine	FirstGeneration
6443	Promazine 25mg/5ml oral solution	Promazine	FirstGeneration
8988	PROMAZINE HCl 25 MG TAB	Promazine	FirstGeneration
10666	Dolmatil 200mg tablets (Sanofi)	Sulpiride	FirstGeneration
18181	Sulpor 200mg/5ml oral solution (Rosemont Pharmaceuticals Ltd)	Sulpiride	FirstGeneration
2135	Sulpiride 200mg tablets	Sulpiride	FirstGeneration
8903	Sulpiride 200mg/5ml oral solution sugar free	Sulpiride	FirstGeneration
9247	Sulpiride 400mg tablets	Sulpiride	FirstGeneration
10405	Thioridazine 25mg/5ml sugar free Oral solution	Thioridazine	FirstGeneration
10675	Thioridazine 100mg/5ml oral suspension	Thioridazine	FirstGeneration
10870	THIORIDAZINE S/F 50 MG/5ML SYR	Thioridazine	FirstGeneration
1162	Melleril 25mg tablets (Novartis Pharmaceuticals UK Ltd)	Thioridazine	FirstGeneration

1192	Thioridazine 10mg tablets	Thioridazine	FirstGeneration
1218	Thioridazine 25mg tablets	Thioridazine	FirstGeneration
1314	Thioridazine 50mg tablets	Thioridazine	FirstGeneration
15157	Melleril 100mg/5ml oral suspension (Novartis Pharmaceuticals UK Ltd)	Thioridazine	FirstGeneration
2502	Melleril 50mg tablets (Novartis Pharmaceuticals UK Ltd)	Thioridazine	FirstGeneration
2801	Thioridazine 10mg/5ml Oral solution	Thioridazine	FirstGeneration
284	Melleril 10mg tablets (Novartis Pharmaceuticals UK Ltd)	Thioridazine	FirstGeneration
3021	Thioridazine 100mg tablets	Thioridazine	FirstGeneration
3579	Melleril 25mg/5ml oral suspension (Novartis Pharmaceuticals UK Ltd)	Thioridazine	FirstGeneration
3605	Thioridazine 25mg/5ml oral solution	Thioridazine	FirstGeneration
4673	Melleril 25mg/5ml syrup (Novartis Pharmaceuticals UK Ltd)	Thioridazine	FirstGeneration
8774	Melleril 100mg tablets (Novartis Pharmaceuticals UK Ltd)	Thioridazine	FirstGeneration
9387	Thioridazine 25mg/5ml oral suspension	Thioridazine	FirstGeneration
10535	TRIFLUOPERAZINE 5.00MG/ML 5 MG SYR	Trifluoperazine	FirstGeneration
11531	Trifluoperazine 5mg/5ml oral solution sugar free	Trifluoperazine	FirstGeneration
1159	Trifluoperazine 2mg modified-release capsules	Trifluoperazine	FirstGeneration
1245	Trifluoperazine 5mg tablets	Trifluoperazine	FirstGeneration
13145	Trifluoperazine 1mg/5ml oral solution sugar free	Trifluoperazine	FirstGeneration
1316	Stelazine 5mg tablets (Mercury Pharma Group Ltd)	Trifluoperazine	FirstGeneration
1318	Stelazine 1mg tablets (Mercury Pharma Group Ltd)	Trifluoperazine	FirstGeneration
1735	Stelazine 10mg Spansules (Mercury Pharma Group Ltd)	Trifluoperazine	FirstGeneration
1857	Trifluoperazine 1mg tablets	Trifluoperazine	FirstGeneration
18668	Trifluoperazine 10mg/ml concentrate	Trifluoperazine	FirstGeneration
2713	Stelazine 2mg Spansules (Mercury Pharma Group Ltd)	Trifluoperazine	FirstGeneration
2714	Trifluoperazine 10mg modified-release capsules	Trifluoperazine	FirstGeneration
3356	Parstelin Tablet (GlaxoSmithKline Consumer Healthcare)	Trifluoperazine	FirstGeneration
3955	Tranylcypromine with trifluoperazine Tablet	Trifluoperazine	FirstGeneration
8445	Stelabid Tablet (GlaxoSmithKline Consumer Healthcare)	Trifluoperazine	FirstGeneration
8985	Stelazine 1mg/5ml syrup (Mercury Pharma Group Ltd)	Trifluoperazine	FirstGeneration
12224	Zuclopenthixol decanoate 500mg/1ml solution for injection ampoules	Zuclopenthixol	FirstGeneration
12707	Zuclopenthixol 2mg tablets	Zuclopenthixol	FirstGeneration
1319	Clopixol 10mg tablets (Lundbeck Ltd)	Zuclopenthixol	FirstGeneration

13368	Clopixol 2mg tablets (Lundbeck Ltd)	Zuclopenthixol	FirstGeneration
3774	Clopixol 200mg/ml Oily injection (Lundbeck Ltd)	Zuclopenthixol	FirstGeneration
3775	Zuclopenthixol decanoate 200mg/ml oily injection	Zuclopenthixol	FirstGeneration
9686	Zuclopenthixol 10mg tablets	Zuclopenthixol	FirstGeneration
11938	Amisulpride 25mg/5ml oral suspension	Amisulpride	SecondGeneration
16768	Solian 50 tablets (Sanofi)	Amisulpride	SecondGeneration
41714	Amisulpride 50mg tablets (Zentiva)	Amisulpride	SecondGeneration
46889	Amisulpride 25mg/5ml oral solution	Amisulpride	SecondGeneration
4876	Amisulpride 50mg tablets	Amisulpride	SecondGeneration
5071	Amisulpride 200mg tablets	Amisulpride	SecondGeneration
5927	Amisulpride 400mg tablets	Amisulpride	SecondGeneration
6482	Amisulpride 100mg/ml oral solution sugar free	Amisulpride	SecondGeneration
6524	Amisulpride 100mg tablets	Amisulpride	SecondGeneration
14344	Aripiprazole 5mg tablets	Aripiprazole	SecondGeneration
14858	Abilify 15mg tablets (Otsuka Pharmaceuticals (U.K.) Ltd)	Aripiprazole	SecondGeneration
16575	Aripiprazole 1mg/ml oral solution	Aripiprazole	SecondGeneration
18132	Abilify 5mg tablets (Otsuka Pharmaceuticals (U.K.) Ltd)	Aripiprazole	SecondGeneration
24358	Abilify 10mg tablets (Otsuka Pharmaceuticals (U.K.) Ltd)	Aripiprazole	SecondGeneration
32076	Aripiprazole 10mg orodispersible tablets sugar free	Aripiprazole	SecondGeneration
6561	Aripiprazole 10mg tablets	Aripiprazole	SecondGeneration
6573	Aripiprazole 15mg tablets	Aripiprazole	SecondGeneration
8046	Clozapine 25mg tablets	Clozapine	SecondGeneration
8047	Clozapine 100mg tablets	Clozapine	SecondGeneration
1249	Olanzapine 10mg tablets	Olanzapine	SecondGeneration
14717	Zyprexa 5mg Velotabs (Eli Lilly and Company Ltd)	Olanzapine	SecondGeneration
18024	Zyprexa 5mg tablets (Eli Lilly and Company Ltd)	Olanzapine	SecondGeneration
18453	Zyprexa 2.5mg tablets (Eli Lilly and Company Ltd)	Olanzapine	SecondGeneration
2656	Olanzapine 2.5mg tablets	Olanzapine	SecondGeneration
29540	Olanzapine 20mg tablets	Olanzapine	SecondGeneration
3281	Olanzapine 5mg tablets	Olanzapine	SecondGeneration
47055	Olanzapine 5mg orodispersible tablets	Olanzapine	SecondGeneration
47063	Olanzapine 10mg orodispersible tablets sugar free	Olanzapine	SecondGeneration

47098	Olanzapine 5mg orodispersible tablets sugar free	Olanzapine	SecondGeneration
50214	Olanzapine 5mg orodispersible tablets	Olanzapine	SecondGeneration
55622	Olanzapine 10mg orodispersible tablets	Olanzapine	SecondGeneration
5653	Olanzapine 7.5mg tablets	Olanzapine	SecondGeneration
57160	Olanzapine 5mg oral lyophilisates sugar free	Olanzapine	SecondGeneration
6023	Olanzapine 10mg Orodispersible tablet	Olanzapine	SecondGeneration
6412	Olanzapine 5mg Orodispersible tablet	Olanzapine	SecondGeneration
6838	Olanzapine 15mg Orodispersible tablet	Olanzapine	SecondGeneration
6850	Olanzapine 15mg tablets	Olanzapine	SecondGeneration
10107	Quetiapine Starter Pack	Quetiapine	SecondGeneration
14859	Seroquel 25mg tablets (AstraZeneca UK Ltd)	Quetiapine	SecondGeneration
18013	Seroquel 100mg tablets (AstraZeneca UK Ltd)	Quetiapine	SecondGeneration
18023	Seroquel tablets starter pack (AstraZeneca UK Ltd)	Quetiapine	SecondGeneration
38885	Quetiapine 50mg modified-release tablets	Quetiapine	SecondGeneration
38906	Quetiapine 300mg modified-release tablets	Quetiapine	SecondGeneration
38913	Seroquel XL 50mg tablets (AstraZeneca UK Ltd)	Quetiapine	SecondGeneration
38914	Seroquel XL 200mg tablets (AstraZeneca UK Ltd)	Quetiapine	SecondGeneration
40779	Quetiapine 100mg/5ml oral solution	Quetiapine	SecondGeneration
40932	Quetiapine 100mg/5ml oral suspension	Quetiapine	SecondGeneration
44024	Quetiapine 150mg modified-release tablets	Quetiapine	SecondGeneration
45839	Quetiapine 25mg/5ml oral suspension	Quetiapine	SecondGeneration
46764	Quetiapine 12.5mg/5ml oral solution	Quetiapine	SecondGeneration
46871	Quetiapine 12.5mg/5ml oral suspension	Quetiapine	SecondGeneration
5039	Quetiapine 100mg tablets	Quetiapine	SecondGeneration
5040	Quetiapine 150mg tablets	Quetiapine	SecondGeneration
5283	Quetiapine 25mg tablets	Quetiapine	SecondGeneration
61575	Ebesque XL 50mg tablets (Ethypharm UK Ltd)	Quetiapine	SecondGeneration
63049	Biquelle XL 50mg tablets (Aspire Pharma Ltd)	Quetiapine	SecondGeneration
63389	Zaluron XL 50mg tablets (Fontus Health Ltd)	Quetiapine	SecondGeneration
70192	Quetiapine 20mg/ml oral suspension sugar free	Quetiapine	SecondGeneration
7039	Quetiapine 300mg tablets	Quetiapine	SecondGeneration
9794	Quetiapine 200mg tablets	Quetiapine	SecondGeneration

12445	Remoxipride 300mg capsule	Remoxipride	SecondGeneration
11821	Risperdal Quicklet 500microgram orodispersible tablets (Janssen-Cilag Ltd)	Risperidone	SecondGeneration
1320	Risperidone 1mg tablets	Risperidone	SecondGeneration
1321	Risperidone 2mg tablets	Risperidone	SecondGeneration
16986	Risperdal Quicklet 1mg orodispersible tablets (Janssen-Cilag Ltd)	Risperidone	SecondGeneration
2787	Risperidone 4mg tablets	Risperidone	SecondGeneration
302	Risperidone 1mg/ml oral solution sugar free	Risperidone	SecondGeneration
35141	Risperidone 3mg orodispersible tablets sugar free	Risperidone	SecondGeneration
4820	Risperdal 1mg tablets (Janssen-Cilag Ltd)	Risperidone	SecondGeneration
5219	Risperidone 3mg tablets	Risperidone	SecondGeneration
5262	Risperdal 1mg/ml oral solution (Janssen-Cilag Ltd)	Risperidone	SecondGeneration
631	Risperdal 500microgram tablets (Janssen-Cilag Ltd)	Risperidone	SecondGeneration
6373	Risperidone 1mg orodispersible tablets sugar free	Risperidone	SecondGeneration
64957	Risperidone 1mg/ml oral solution sugar free (Rosemont Pharmaceuticals Ltd)	Risperidone	SecondGeneration
667	Risperidone 500microgram tablets	Risperidone	SecondGeneration
7382	Risperidone 500microgram orodispersible tablets sugar free	Risperidone	SecondGeneration
9659	Risperdal 2mg tablets (Janssen-Cilag Ltd)	Risperidone	SecondGeneration

Code list for stroke – ICD10

ICD10	Description
G45	Transient cerebral ischaemic attacks and related sydnromes
G45.0	Vertebro-basilar artery syndrome
G45.1	Carotif artery syndrome (hemispheric)
G45.2	Multiple and bilateral precerebral artery syndromes
G45.3	Amaurosis fugax
G45.8	Other transient cerebral ischaemic attacks and related syndromes
G45.9	Transient cerebral ischaemic attack, unspecified
G46	Vascular syndromes of brain in cerebrovascular diseases
G46.0	Middle cerebral artery syndrome
G46.1	Anterior cerebral artery syndrome
G46.2	Posterior cerebral artery syndrome
G46.3	Brain stem stroke syndrome
G46.4	Cerebellar stroke syndrome
G46.5	Pure motor lacunar syndrome
G46.6	Pure sensory lacunar syndrome
G46.7	Other lacunar syndromes
G46.8	Other vascular syndromes of brain in cerebrovascular disease
I61	Intracerebral haemorrhage
I61.0	Intracerebral haemorrhage in hemisphere, subcortical
I61.1	Intracerebral haemorrhage in hemisphere, cortical
I61.2	Intracerebral haemorrhage in hemisphere, unspecified
I61.3	Intracerebral haemorrhage in brain stem
I61.4	Intracerebral haemorrhage in cerebellum
I61.5	Intracerebral haemorrhage, intraventricular
I61.6	Intracerebral haemorrhage, multiple localized
I61.8	Other intracerebral haemorrhage
I61.9	Intracerebral haemorrhage, unspecified
I63	Cerebral infarction
I63.0	Cerebral infarction due to thrombosis of precerebral arteries
I63.1	Cerebral infarction due to embolism of precerebral arteries
I63.2	Cerebral infarction due to unspecified occlusion or stenosis of precerebral arteries
I63.3	Cerebral infarction due to thrombosis of cerebral arteries
I63.4	Cerebral infarction due to embolism of cerebral arteries
I63.5	Cerebral infarction due to unspecified occlusion or stenosis of cerebral arteries
I63.8	Other cerebral infarction
I63.9	Cerebral infarction, unspecified
I64	Stroke, not specified as haemorrhage or infarction

Code list for stroke – Aurum and GOLD

Source	SNOMED_CT_Concept_id	SNOMED_CT_Description_id	Medcode_Aurum	Medcode_GOLD	Readcode	Term	History_only
Aurum and GOLD	1386000	475553012	300298011	20284	G62z.00	intracranial haemorrhage nos	
Aurum and GOLD	7713009	503469016	503469016	7912	G614.00	pontine haemorrhage	
Aurum and GOLD	20059004	33759015	300943012	92036	Gyu6600	[x]occlusion and stenosis of other cerebral arteries	
Aurum and GOLD	20059004	33759015	370701000006118	91627	Gyu6300	[x]cerebrl infarctn due/unspcf occlusn or sten/cerebrl artrs	
Aurum and GOLD	20059004	33759015	543141000006110	33543	G6X..00	cerebrl infarctn due/unspcf occlusn or sten/cerebrl artrs	
Aurum and GOLD	20059004	1222398015	1222398015	8837	G64..00	cerebral arterial occlusion	
Aurum and GOLD	21454007	36011016	428181000006115	108630	Gyu6E00	[x]subarachnoid haemorrh from intracranial artery, unspecif	
Aurum and GOLD	29322000	49074018	300380014	70536	G671000	acute cerebrovascular insufficiency nos	
Aurum and GOLD	49422009	495394013	495394013	31595	G610.00	cortical haemorrhage	
Aurum and GOLD	52201006	496232015	496232015	40338	G611.00	internal capsule haemorrhage	
Aurum and GOLD	62914000	104563015	104563015	2418	G6...00	cerebrovascular disease	
Aurum and GOLD	62914000	104563015	300290016	31805	G62..00	other and unspecified intracranial haemorrhage	
Aurum and GOLD	62914000	104563015	300374018	13577	G67..00	other cerebrovascular disease	
Aurum and GOLD	62914000	104563015	300401011	34117	G67y.00	other cerebrovascular disease os	
Aurum and GOLD	62914000	104563015	300402016	37493	G67z.00	other cerebrovascular disease nos	
Aurum and GOLD	62914000	104563015	300418010	51311	G6y..00	other specified cerebrovascular disease	
Aurum and GOLD	62914000	104563015	300419019	10062	G6z..00	cerebrovascular disease nos	
Aurum and GOLD	62914000	104563015	300944018	111096	Gyu6700	[x]other specified cerebrovascular diseases	
Aurum and GOLD	62914000	104563015	370711000006115	73901	Gyu6.00	[x]cerebrovascular diseases	
Aurum and GOLD	62914000	104563015	407291000006117	99367	Gyu6A00	[x]other cerebrovascular disorders in diseases ce	
Aurum and GOLD	64009001	499739014	499739014	5268	G650.11	insufficiency - basilar artery	
Aurum and GOLD	71444005	118689010	118689010	16517	G640.00	cerebral thrombosis	

Aurum and GOLD	75038005	502878012	502878012	13564	G613.00	cerebellar haemorrhage	
Aurum and GOLD	75543006	125470015	125470015	15019	G641.00	cerebral embolism	
Aurum and GOLD	75543006	125470015	542831000006116	34758	G641.11	cerebral embolus	
Aurum and GOLD	78569004	130374019	57341000006119	47642	G64z100	wallenberg syndrome	
Aurum and GOLD	78569004	130375018	130375018	5185	G64z111	lateral medullary syndrome	
Aurum and GOLD	86003009	142588012	100771000006112	4152	G631.12	thrombosis, carotid artery	
Aurum and GOLD	95457000	158113017	345650013	15252	G64z.11	brainstem infarction nos	
Aurum and GOLD	95457000	158113017	524541000006117	25615	G64z000	brainstem infarction	
Aurum and GOLD	95460007	158118014	158118014	5602	G64z.12	cerebellar infarction	
Aurum and GOLD	111298007	360778011	360778011	24385	G671100	chronic cerebral ischaemia	
Aurum and GOLD	175399007	271573013	271573013	104517	7A24400	open embolectomy of cerebral artery	
Aurum and GOLD	175401001	271576017	271576017	48118	7A24600	open embolisation of cerebral artery	
Aurum and GOLD	175406006	271585017	271585017	34171	7A25000	percutaneous transluminal embolisation of cerebral artery	
Aurum and GOLD	195165005	300272017	503791000006114	46316	G612.00	basal nucleus haemorrhage	
Aurum and GOLD	195167002	300276019	300276019	30045	G616.00	external capsule haemorrhage	
Aurum and GOLD	195168007	300277011	300277011	30202	G617.00	intracerebral haemorrhage, intraventricular	
Aurum and GOLD	195169004	300280012	746571000006116	57315	G618.00	intracerebral haemorrhage, multiple localized	
Aurum and GOLD	195180004	300303013	300303013	32447	G630.00	basilar artery occlusion	
Aurum and GOLD	195185009	300312010	300312010	23671	G63y000	cerebral infarct due to thrombosis of precerebral arteries	
Aurum and GOLD	195186005	300313017	300313017	24446	G63y100	cerebral infarction due to embolism of precerebral arteries	
Aurum and GOLD	195189003	300321011	300321011	36717	G640000	cerebral infarction due to thrombosis of cerebral arteries	
Aurum and GOLD	195190007	300322016	300322016	27975	G641000	cerebral infarction due to embolism of cerebral arteries	
Aurum and GOLD	195200006	300344014	300344014	44765	G653.00	carotid artery syndrome hemispheric	
Aurum and GOLD	195206000	300353019	300353019	16507	G65z100	intermittent cerebral ischaemia	
Aurum and GOLD	195209007	300362017	300362017	18689	G660.00	middle cerebral artery syndrome	
Aurum and GOLD	195210002	300363010	300363010	19280	G661.00	anterior cerebral artery syndrome	
Aurum and GOLD	195211003	300364016	300364016	19260	G662.00	posterior cerebral artery syndrome	
Aurum and GOLD	195212005	300365015	524511000006116	8443	G663.00	brain stem stroke syndrome	

Aurum and GOLD	195213000	300366019	300366019	17322	G664.00	cerebellar stroke syndrome	
Aurum and GOLD	195216008	300370010	300370010	7780	G667.00	left sided cva	
Aurum and GOLD	195217004	300371014	300371014	12833	G668.00	right sided cva	
Aurum and GOLD	195232006	300395011	300395011	51759	G677000	occlusion and stenosis of middle cerebral artery	
Aurum and GOLD	195233001	300396012	300396012	57527	G677100	occlusion and stenosis of anterior cerebral artery	
Aurum and GOLD	195234007	300398013	300398013	65770	G677200	occlusion and stenosis of posterior cerebral artery	
Aurum and GOLD	195235008	300399017	300399017	55602	G677300	occlusion and stenosis of cerebellar arteries	
Aurum and GOLD	195236009	300400012	267311000006118	71274	G677400	occlusion+stenosis of multiple and bilat cerebral arteries	
Aurum and GOLD	195239002	300403014	149571000006118	6228	G68X.00	sequelae of stroke,not specfd as h'morrhage or infarction	History
Aurum and GOLD	195239002	300403014	149701000006118	51138	G68W.00	sequelae/other + unspecified cerebrovascular diseases	History
Aurum and GOLD	195239002	300403014	426321000006116	110337	Gyu6C00	[x]sequelae of stroke,not specfd as h'morrhage or infarction	History
Aurum and GOLD	195241001	300407010	300407010	48149	G681.00	sequelae of intracerebral haemorrhage	History
Aurum and GOLD	195243003	300411016	300411016	39403	G683.00	sequelae of cerebral infarction	History
Aurum and GOLD	230690007	345635016	605491000006113	6116	G66..13	cva - cerebrovascular accident unspecified	
Aurum and GOLD	230690007	345637012	405339016	1469	G66..00	stroke and cerebrovascular accident unspecified	
Aurum and GOLD	230690007	345637012	122401000006115	6253	G66..12	stroke unspecified	
Aurum and GOLD	230690007	345637012	605501000006117	1298	G66..11	cva unspecified	
Aurum and GOLD	230691006	345638019	122361000006113	6155	G64..13	stroke due to cerebral arterial occlusion	
Aurum and GOLD	230691006	345638019	605461000006117	5363	G64..11	cva - cerebral artery occlusion	
Aurum and GOLD	230692004	345639010	345639010	57495	G63..11	infarction - precerebral	
Aurum and GOLD	230698000	345651012	299342019	93459	Fyu5600	[x]other lacunar syndromes	
Aurum and GOLD	230699008	345655015	345655015	33499	G665.00	pure motor lacunar syndrome	
Aurum and GOLD	230700009	345658018	345658018	51767	G666.00	pure sensory lacunar syndrome	
Aurum and GOLD	230716006	345684012	345684012	105738	G657.00	carotid territory transient ischaemic attack	
Aurum and GOLD	233317001	349598015	271590019	26094	7A25200	embolisation of cerebral artery nec	
Aurum and GOLD	266253001	395777014	395777014	45781	G63..00	precerebral arterial occlusion	
Aurum and GOLD	266254007	395778016	395778016	4240	G631.00	carotid artery occlusion	

Aurum and GOLD	266257000	395783012	395783012	1433	G65..12	transient ischaemic attack	
Aurum and GOLD	266257000	395788015	300348012	19354	G65y.00	other transient cerebral ischaemia	
Aurum and GOLD	266257000	395788015	300349016	15788	G65zz00	transient cerebral ischaemia nos	
Aurum and GOLD	266257000	395788015	395788015	504	G65..00	transient cerebral ischaemia	
Aurum and GOLD	266257000	395788015	95931000006111	1895	G65z.00	transient cerebral ischaemia nos	
Aurum and GOLD	266257000	395788015	416991000006112	63746	Fyu5500	[x]other transnt cerebral ischaemic attacks+related syndroms	
Aurum and GOLD	274100004	409859018	122371000006118	18604	G61..12	stroke due to intracerebral haemorrhage	
Aurum and GOLD	274100004	409859018	605471000006112	6960	G61..11	cva - cerebrovascular accid due to intracerebral haemorrhage	
Aurum and GOLD	274100004	409860011	300939013	53810	Gyu6200	[x]other intracerebral haemorrhage	
Aurum and GOLD	274100004	409860011	300956017	96630	Gyu6F00	[x]intracerebral haemorrhage in hemisphere, unspecified	
Aurum and GOLD	274100004	409860011	744921000006116	31060	G61X.00	intracerebral haemorrhage in hemisphere, unspecified	
Aurum and GOLD	274100004	409860011	748941000006115	28314	G61X000	left sided intracerebral haemorrhage, unspecified	
Aurum and GOLD	274100004	2819959010	300287010	3535	G61z.00	intracerebral haemorrhage nos	
Aurum and GOLD	274100004	2819959010	744901000006114	5051	G61..00	intracerebral haemorrhage	
Aurum and GOLD	307766002	451133011	451133011	9985	G64z200	left sided cerebral infarction	
Aurum and GOLD	307767006	451134017	451134017	10504	G64z300	right sided cerebral infarction	
Aurum and GOLD	308128006	451441015	163261000006119	19201	G61X100	right sided intracerebral haemorrhage, unspecified	
Aurum and GOLD	363302008	482447013	149551000006111	43451	G682.00	sequelae of other nontraumatic intracranial haemorrhage	History
Aurum and GOLD	390936003	1477210016	841051000006113	9943	G678.00	cereb autosom dominant arteriop subcort infarcts leukoenceph	
Aurum and GOLD	413102000	2474651019	2474651019	26424	G64z400	infarction of basal ganglia	
Aurum and GOLD	432504007	2770034014	300941014	53745	Gyu6400	[x]other cerebral infarction	
Aurum and GOLD	432504007	2770034014	395780010	3149	G64z.00	cerebral infarction nos	
Aurum and GOLD	432504007	2770034014	218511000000117	569	G64..12	infarction - cerebral	
Aurum and GOLD	443929000	2842187014	1130181000000113	98188	G679.00	small vessel cerebrovascular disease	
Aurum and GOLD	732923001	3467314012	483988011	62342	G615.00	bulbar haemorrhage	
Aurum and GOLD	125081000119106	3042974014	370661000006114	94482	Gyu6G00	[x]cereb infarct due unsp occlus/stenos precerebr arteries	
Aurum and GOLD	125081000119106	3042974014	542261000006114	40758	G6W..00	cereb infarct due unsp occlus/stenos precerebr arteries	

Aurum and GOLD	914991000000106	2350961000000113	1900651000006110	108569	2Ba2200	scpe class predom patt c.3 infarct of middle cerebral artery	
Aurum and GOLD	.	.	251692018	13567	14AB	H/O TIA	History
Aurum and GOLD	.	.	411518010	6305	14A7.11	H/O: CVA	History
Aurum and GOLD	.	.	413750011	100639	1M4.00	Central post-stroke pain	History
Aurum and GOLD	.	.	1780304018	18686	662e.00	Stroke/CVA annual review	History
Aurum and GOLD	.	.	2476091017	5871	14A7.12	H/O stroke	History
Aurum and GOLD	.	.	809421000006116	34135	14A7.00	H/O: CVA/stroke	History
Aurum only	195180004	884461000006113	884461000006113	.	G630.99	basilar artery occluded	
Aurum only	195239002	300403014	426481000006114	.	Gyu6D00	[x]sequelae/other + unspecified cerebrovascular diseases	History
Aurum only	266254007	884471000006118	884471000006118	.	G631.99	carotid artery occluded	
Aurum only	266257000	988951000006117	988951000006117	.	G65..99	transient ischaemic attacks	
Aurum only	274100004	409860011	989201000006117	.	G61..99	cerebral haemorrhage	
Aurum only	274100004	884421000006119	884421000006119	.	G6...99	cerebral haemorrhage	
Aurum only	274100004	989211000006119	989211000006119	.	G61..98	cerebral haemorrhage nos	
Aurum only	363302008	482447013	426231000006114	.	Gyu6B00	[x]sequelae of other nontraumatic intracranial haemorrhage	History
Aurum only	373606000	1212072018	1212072018	.		occlusive stroke	
Aurum only	584181000000100	884511000006111	884511000006111	.	G65z.99	transient ischaemic attacks	
Aurum only	682621000000105	884501000006113	884501000006113	.	G64z.99	cerebral a. occlusion nos	
Aurum only	685631000000102	884521000006115	884521000006115	.	G66..98	stroke/cva - undefined	
Aurum only	685631000000102	884531000006117	884531000006117	.	G66..99	stroke	
Aurum only	700251000000105	884451000006111	884451000006111	.	G62z.99	cerebral haemorrhage nos	
Aurum only	907581000006103	907581000006119	907581000006119	.		[rfc] stroke/cva	
Aurum only	907591000006100	907591000006116	907591000006116	.		[rfc] stroke	
Aurum only	908801000006105	908801000006114	908801000006114	.		[rfc] stroke	
Aurum only	909171000006104	909171000006115	909171000006115	.		[rfc] cva	
Aurum only	1573101000006108	1573101000006112	1573101000006112	.		cerebral infarction with haemorrhagic transformation	
Aurum only	1576261000006108	1576261000006112	1576261000006112	.		cause of death- cerebral infarct	

Code list for venous thromboembolism (VTE) – ICD10

ICD10	Description
I26	Pulmonary Embolism
I26.0	Pulmonary Embolism with mention of acute cor pulmonale
I26.9	Pulmonary Embolism without mention of acute cor pulmonale
I80.1	Phlebitis and thrombophlebitis of femoral vein
I80.2	Phlebitis and thrombophlebitis of other deep vessels of lower extremities
I80.3	Phlebitis and thrombophlebitis of lower extremities, unspecified
I82	Other venous embolism and thrombosis

Code list for venous thromboembolism (VTE) – Aurum and GOLD

Source	SNOMED_CT_Concept_id	SNOMED_CT_Description_id	Medcode_Aurum	Medcode_GOLD	Readcode	Term	History_only
Aurum and GOLD	128053003	194647015	2162149016	824	G801.11	Deep vein thrombosis	
Aurum and GOLD	128053003	2162149016	2162148012	3392	G801.13	DVT - Deep vein thrombosis	
Aurum and GOLD	134399007	216205019	216205019	42506	G801C00	Deep vein thrombosis of leg related to air travel	
Aurum and GOLD	14246007	1221072019	1221072019	36568	F050.00	Embolism of central nervous system venous sinus	
Aurum and GOLD	161508001	251689017	251689017	5614	14A8100	H/O: Deep Vein Thrombosis	History
Aurum and GOLD	161512007	251693011	251693011	10280	14AC.00	H/O: pulmonary embolus	History
Aurum and GOLD	161512007	251693011	451479013	16976	ZV12900	[V] Personal history of pulmonary embolism	History
Aurum and GOLD	175262009	271392011	271392011	34687	7A09311	Trendelenburg pulmonary embolectomy	
Aurum and GOLD	175262009	271393018	261021000006119	45740	7A09300	Open embolectomy of pulmonary artery	
Aurum and GOLD	175263004	271394012	271394012	35472	7A09400	Open embolisation of pulmonary artery	
Aurum and GOLD	175843001	272243017	272243017	5696	7A6B.00	Open removal of thrombus from vein	
Aurum and GOLD	175843001	272243017	272247016	83512	7A6By00	Other specified open removal of thrombus from vein	
Aurum and GOLD	175843001	272243017	272248014	15798	7A6Bz00	Open removal of thrombus from vein NOS	
Aurum and GOLD	175843001	272244011	272244011	18144	7A6B.11	Open vein thrombectomy	
Aurum and GOLD	175844007	272245012	272245012	70634	7A6B000	Open thrombectomy of vein of upper limb	
Aurum and GOLD	175845008	272246013	272246013	33600	7A6B100	Open thrombectomy of vein of lower limb	
Aurum and GOLD	194883006	299847015	216591000006115	18121	G401000	Post operative pulmonary embolus	
Aurum and GOLD	195394007	300649013	420621000006111	97853	Gyu8000	[X]Phlebitis+thrombophlebitis/oth deep vessls/low extremites	
Aurum and GOLD	195404000	300666010	300666010	30743	G801000	Phlebitis of the femoral vein	
Aurum and GOLD	195405004	300667018	300667018	54663	G801100	Phlebitis of the popliteal vein	
Aurum and GOLD	195410000	300672010	300672010	15382	G801600	Thrombophlebitis of the femoral vein	
Aurum and GOLD	195411001	300673017	300673017	55661	G801700	Thrombophlebitis of the popliteal vein	

Aurum and GOLD	195425000	300691010	300691010	58166	G80y400	Thrombophlebitis of the common iliac vein	
Aurum and GOLD	195426004	300692015	300692015	94496	G80y500	Thrombophlebitis of the internal iliac vein	
Aurum and GOLD	195427008	300695018	300695018	58023	G80y600	Thrombophlebitis of the external iliac vein	
Aurum and GOLD	195437003	300710016	300710016	43555	G822.00	Embolism and thrombosis of the vena cava	
Aurum and GOLD	195438008	300711017	300711017	5503	G823.00	Embolism and thrombosis of the renal vein	
Aurum and GOLD	197001004	302981012	100821000006116	54205	J420200	Thrombus of the superior mesenteric veins	
Aurum and GOLD	200232006	307740016	307740016	94405	L413100	Antenatal deep vein thrombosis - delivered	
Aurum and GOLD	200233001	307741017	307741017	100502	L413200	Antenatal deep vein thrombosis with antenatal complication	
Aurum and GOLD	200237000	307748011	211691000006114	112358	L414100	Postnatal deep vein thrombosis - delivered with p/n comp	
Aurum and GOLD	200238005	307749015	307749015	23588	L414200	Postnatal deep vein thrombosis with postnatal complication	
Aurum and GOLD	200284000	307815016	268641000006113	73569	L43z.00	Obstetric pulmonary embolism NOS	
Aurum and GOLD	200284000	307815016	307815016	7174	L43..00	Obstetric pulmonary embolism	
Aurum and GOLD	200284000	307815016	307866014	109337	L43yz00	Other obstetric pulmonary embolism NOS	
Aurum and GOLD	200284000	307815016	307867017	98639	L43zz00	Obstetric pulmonary embolism NOS	
Aurum and GOLD	200284000	307815016	307868010	101944	L43z000	Obstetric pulmonary embolism NOS, unspecified	
Aurum and GOLD	200284000	307815016	307869019	97367	L43z100	Obstetric pulmonary embolism NOS - delivered	
Aurum and GOLD	200284000	307815016	307872014	44404	L43z400	Obstetric pulmonary embolism NOS with postnatal complication	
Aurum and GOLD	200284000	307816015	307816015	68438	L43..11	Obstetric pulmonary embolus	
Aurum and GOLD	200299000	307832017	307832017	49269	L432.00	Obstetric blood-clot pulmonary embolism	
Aurum and GOLD	200299000	307832017	307838018	112710	L432z00	Obstetric blood-clot pulmonary embolism NOS	
Aurum and GOLD	200303005	307836019	269431000006114	112578	L432300	Obstetric blood-clot pulmonary embolism + a/n complication	

Aurum and GOLD	213220000	325066018	216571000006116	1224	SP12200	Post operative deep vein thrombosis	
Aurum and GOLD	213309003	325205013	325205013	38372	SP32100	Thromboembolism after infusion	
Aurum and GOLD	213310008	325206014	325206014	25161	SP32200	Thrombophlebitis after infusion	
Aurum and GOLD	233947005	350517010	350517010	102444	G41y100	Thromboembolic pulmonary hypertension	History
Aurum and GOLD	233972005	350563016	350563016	23477	G740.14	Saddle embolus	
Aurum and GOLD	266267005	395801015	218551000000118	25478	G801.12	Deep vein thrombosis, leg	
Aurum and GOLD	266267005	395801015	300681016	27284	G801z00	Deep vein phlebitis and thrombophlebitis of the leg NOS	
Aurum and GOLD	266267005	395801015	395801015	3576	G801.00	Deep vein phlebitis and thrombophlebitis of the leg	
Aurum and GOLD	276494008	412645018	300697014	70467	G80y800	Phlebitis and thrombophlebitis of the iliac vein NOS	
Aurum and GOLD	276494008	412645018	412645018	38099	G80y.11	Phlebitis and/or thrombophlebitis of iliac vein	
Aurum and GOLD	276977004	413381016	1549601000006119	89444	7A6Q.00	Percutaneous removal of thrombus from vein	
Aurum and GOLD	276977004	413381016	359571000000118	102038	7A6Qz00	Percutaneous removal of thrombus from vein NOS	
Aurum and GOLD	276977004	413381016	359771000000114	111872	7A6Qy00	Other specified percutaneous removal of thrombus from vein	
Aurum and GOLD	276985008	413389019	358041000000114	84367	7A6S300	Percutaneous transluminal venous thrombolysis NEC	
Aurum and GOLD	281595001	419640012	419640012	97808	G822000	Thrombosis of inferior vena cava	
Aurum and GOLD	297156001	437622013	437622013	100103	G824.00	Axillary vein thrombosis	
Aurum and GOLD	309735004	453210011	453210011	9255	G802000	Thrombosis of vein of leg	
Aurum and GOLD	312588002	456335013	300671015	42158	G801500	Deep vein phlebitis of the leg unspecified	
Aurum and GOLD	312588002	456335013	300690011	54152	G80y300	Phlebitis of the iliac vein unspecified	
Aurum and GOLD	397035004	1776763013	1776763013	104358	7A6Ey11	Fogarty embolectomy of vein	
Aurum and GOLD	40198004	63532015	300677016	32002	G801B00	Deep vein thrombophlebitis of the leg unspecified	
Aurum and GOLD	404223003	2162422011	2162422011	22038	G801D00	Deep vein thrombosis of lower limb	
Aurum and GOLD	413956008	2534187010	2534187010	48920	G801E00	Deep vein thrombosis of leg related to intravenous drug use	
Aurum and GOLD	426937008	2675885015	2675885015	98333	7A6B200	Open thrombectomy of renal vein	
Aurum and GOLD	42861008	71531019	300696017	91282	G80y700	Thrombophlebitis of the iliac vein unspecified	

Aurum and GOLD	429098002	2692478010	300704016	26650	G82..00	Other venous embolism and thrombosis	
Aurum and GOLD	429166006	2695366017	2695366017	49423	780D200	Transjugular intrahepatic thrombolysis of portal vein	
Aurum and GOLD	429311000000105	877481000000112	451478017	19562	ZV12800	[V] Personal history deep vein thrombosis	History
Aurum and GOLD	429311000000105	877491000000114	1227369013	17847	ZV12811	[V] Personal history DVT- deep vein thrombosis	History
Aurum and GOLD	438647008	2795114019	2795114019	105403	G825.00	Thrombosis of subclavian vein	
Aurum and GOLD	438773007	2793990014	632111000000118	96209	G401100	Recurrent pulmonary embolism	
Aurum and GOLD	443210003	1664901000000117	1119161000000115	98526	G801F00	Deep vein thrombosis of peroneal vein	
Aurum and GOLD	492161000000109	1152491000000116	1059641000000112	101655	9kg..00	Deep vein thrombosis - enhanced services administration	History
Aurum and GOLD	49956009	495551019	632731000006118	26873	L413.11	DVT - deep venous thrombosis, antenatal	
Aurum and GOLD	49956009	495552014	495552014	23667	L413.00	Antenatal deep vein thrombosis	
Aurum and GOLD	49956009	83208016	307739018	65725	L413000	Antenatal deep vein thrombosis unspecified	
Aurum and GOLD	49956009	83208016	307742012	61203	L413z00	Antenatal deep vein thrombosis NOS	
Aurum and GOLD	503421000000105	1152451000000112	1715671000006119	102776	9kg0.00	DVT stage 1 service level - enhanced services administration	History
Aurum and GOLD	503421000000105	1152451000000112	1741651000006115	104290	9kg0.11	DVT enhanced services administration stage 1 service level	History
Aurum and GOLD	503451000000100	1152471000000115	1716271000006111	102777	9kg1.00	DVT stage 2 service level - enhanced services administration	History
Aurum and GOLD	503481000000106	1152431000000117	1715681000006116	107023	9kg2.00	DVT stage 3 service level - enhanced services administration	History
Aurum and GOLD	56272000	497563011	497563011	4607	L414.00	Postnatal deep vein thrombosis	
Aurum and GOLD	56272000	497564017	497564017	18830	L414.11	DVT - deep venous thrombosis, postnatal	
Aurum and GOLD	56272000	93577012	307747018	69921	L414000	Postnatal deep vein thrombosis unspecified	
Aurum and GOLD	56272000	93577012	307750015	61204	L414z00	Postnatal deep vein thrombosis NOS	
Aurum and GOLD	59282003	98484016	193601000006115	9701	G401.12	Pulmonary embolus	
Aurum and GOLD	59282003	98484016	98484016	1266	G401.00	Pulmonary embolism	
Aurum and GOLD	609480009	2966889016	305585018	67006	L096400	Pulmonary embolism following abortive pregnancy	
Aurum and GOLD	64662007	107459015	728841000006111	24444	G401.11	Infarction - pulmonary	

Aurum and GOLD	64662007	499917019	499917019	4717	G402.00	Pulmonary infarct	
Aurum and GOLD	710167004	3043650017	2144741000000111	104342	G801G00	Recurrent deep vein thrombosis	
Aurum and GOLD	82385007	136654014	527221000006115	20676	G820.00	Budd - Chiari syndrome (hepatic vein thrombosis)	
Aurum and GOLD	82385007	136654014	64466019	57100	G820.11	Hepatic vein thrombosis	
Aurum and GOLD	864191000000104	2234371000000119	2234371000000119	106850	G826.00	Thrombosis of internal jugular vein	
Aurum and GOLD	864211000000100	2234411000000115	2234411000000115	107851	G827.00	Thrombosis of external jugular vein	
Aurum and GOLD	869611000000104	2247661000000111	2247661000000111	106566	8CMWA00	On deep vein thrombosis care pathway	History
Aurum and GOLD	978421000000101	2488341000000112	2488341000000112	110542	G801H00	Unprovoked deep vein thrombosis	
Aurum and GOLD	978441000000108	2488381000000116	2488381000000116	110449	G801J00	Provoked deep vein thrombosis	
Aurum only	1749291000006109	1749291000006113	1749291000006113			Thromboembolic pulmonary hypertension	History
Aurum only	1786921000006100	1786921000006116	1786921000006116			Recurrent deep vein thrombosis	
Aurum only	200284000	307815016	24951000006110		L43y200	Other obstetric pulmonary embolism - delivered + p/n comp	
Aurum only	200284000	307815016	24981000006119		L43y300	Other obstetric pulmonary embolism with antenatal comp	
Aurum only	200284000	307815016	24991000006116		L43y400	Other obstetric pulmonary embolism with postnatal comp	
Aurum only	200284000	307815016	268671000006117		L43z200	Obstetric pulmonary embolism NOS - delivered with p/n comp	
Aurum only	200284000	307815016	307861016		L43y000	Other obstetric pulmonary embolism unspecified	
Aurum only	200284000	307815016	307862011		L43y100	Other obstetric pulmonary embolism - delivered	
Aurum only	200284000	307815016	307871019		L43z300	Obstetric pulmonary embolism NOS with antenatal complication	
Aurum only	200284000	307815016	398450017		L43y.00	Other obstetric pulmonary embolism	
Aurum only	200284000	888731000006119	888731000006119		L43..99	Obstetrical pulmonary embolism	
Aurum only	200299000	307832017	307833010		L432000	Obstetric blood-clot pulmonary embolism unspecified	
Aurum only	200301007	307834016	307834016		L432100	Obstetric blood-clot pulmonary embolism - delivered	
Aurum only	200302000	307835015	269411000006115		L432200	Obstetric blood-clot pulm embolism - delivered with p/n comp	

Aurum only	200304004	307837011	269441000006116		L432400	Obstetric blood-clot pulmonary embolism + p/n complication	
Aurum only	223501000000102	356141000000111	1548051000006118		7A0C200	Perc translum embolis major systemic pulmonary collater art	
Aurum only	266267005	884731000006110	884731000006110		G801.99	Deep venous thrombosis - leg	
Aurum only	404223003	989221000006110	989221000006110		G801D99	Deep venous thrombosis - leg	
Aurum only	429788001	2691870012	2691870012		780D100	Transjugular intrahepatic thrombectomy of portal vein	
Aurum only	49956009	888681000006112	888681000006112		L413.99	Antepartum DVT	
Aurum only	503451000000100	1152471000000115	1741661000006118		9kg1.11	DVT enhanced services administration stage 2 service level	History
Aurum only	503481000000106	1152431000000117	1741671000006113		9kg2.11	DVT enhanced services administration stage 3 service level	History
Aurum only	56272000	888691000006110	888691000006110		L414.99	Postpartum DVT	
Aurum only	587851000000108	884741000006117	884741000006117		G82z099	Venous embolism NOS	
Aurum only	851241000006104	851241000006115	851241000006115			Axillary vein thrombosis	
Aurum only	905451000006102	905451000006118	905451000006118			[RFC] Pulmonary embolism/pulmonary hypertension	
Aurum only	905491000006108	905491000006112	905491000006112			[RFC] Venous thrombosis	
Aurum only	909471000006105	909471000006114	909471000006114			[RFC] Deep vein thrombosis	

Code list for myocardial infarction – ICD10

ICD10	Description
I21	Acute myocardial infarction
I21.0	Acute transmural myocardial infarction of anterior wall
I21.1	Acute transmural myocardial infarction of inferior wall
I21.2	Acute transmural myocardial infarction of other sites
I21.3	Acute transmural myocardial infarction of unspecified sites
I21.4	Acute subendocardial myocardial infarction
I21.9	Acute myocardial infarction, unspecified
I22	Subsequent myocardial infarction
I22.0	Subsequent myocardial infarction of anterior wall
I22.1	Subsequent myocardial infarction of inferior wall
I22.8	Subsequent myocardial infarction of other sites
I22.9	Subsequent myocardial infarction of unspecified site

Code list for myocardial infarction – Aurum and GOLD

Source	SNOMED_CT_Concept_id	SNOMED_CT_Description_id	Medcode_Aurum	Medcode_GOLD	Readcode	Term	History_only
Aurum only	129574000	208365015	212081000006112	.	G382.00	postoperative transmural myocardial infarction other sites	
Aurum only	1576271000006101	1576271000006117	1576271000006117	.		cause of death- myocardial infarction	
Aurum only	1576301000006104	1576301000006115	1576301000006115	.		cause of death- acute myocardial infarction	
Aurum only	194823009	931961000006117	931961000006117	.	G31y099	acute coronary syndrome	
Aurum only	57054005	884141000006116	884141000006116	.	G30..98	coronary thrombosis	
Aurum only	57054005	884151000006119	884151000006119	.	G30..99	myocardial infarction	
Aurum only	905351000006109	905351000006113	905351000006113	.		[rfc] myocardial infarction (mi)	
Aurum only	932081000006102	932081000006118	932081000006118	.		first myocardial infarction	
Aurum and GOLD	.	.	299719012	28736	G30y000	Acute atrial infarction	
Aurum and GOLD	.	.	350376014	17689	G30..17	Silent myocardial infarction	
Aurum and GOLD	.	.	1218860015	62626	G30y100	Acute papillary muscle infarction	
Aurum and GOLD	.	.	299712015	63467	G306.00	True posterior myocardial infarction	
Aurum and GOLD	.	.	158601000006116	59189	G363.00	Ruptur cardiac wall w'out haemopericard/cur comp fol ac MI	
Aurum and GOLD	.	.	219521000000119	13566	G30..11	Attack-heart	
Aurum and GOLD	.	.	37443015	1204	G30..14	Heart attack	
Aurum and GOLD	.	.	159001000006119	69474	G365.00	Rupture papillary muscle/curr comp fol acute myocard infarct	
Aurum and GOLD	.	.	1229885017	68357	G31y100	microinfarction of heart	
Aurum and GOLD	129574000	208365015	455423016	68748	G38z.00	postoperative myocardial infarction, unspecified	
Aurum and GOLD	129574000	208365015	212091000006110	106812	G383.00	postoperative transmural myocardial infarction unspec site	
Aurum and GOLD	129574000	208365015	208365015	32272	G38..00	postoperative myocardial infarction	
Aurum and GOLD	15990001	27071012	967931000006114	32854	G30B.00	acute posterolateral myocardial infarction	
Aurum and GOLD	164865005	256452010	256460011	59032	323Z.00	ecg: myocardial infarct nos	History
Aurum and GOLD	164865005	256452010	256452010	7783	323..00	ecg: myocardial infarction	History
Aurum and GOLD	194821006	2619484018	2619484018	55137	G311011	mi - myocardial infarction aborted	

Aurum and GOLD	194821006	2619484018	682481000006118	61072	G311000	myocardial infarction aborted	
Aurum and GOLD	194856005	299808017	118831000006118	46166	G35X.00	subsequent myocardial infarction of unspecified site	
Aurum and GOLD	194856005	299808017	299808017	18842	G35..00	subsequent myocardial infarction	
Aurum and GOLD	194856005	299808017	299813018	72562	G353.00	subsequent myocardial infarction of other sites	
Aurum and GOLD	194856005	299808017	300882013	99991	Gyu3600	[x]subsequent myocardial infarction of unspecified site	
Aurum and GOLD	194856005	299808017	300881018	109035	Gyu3500	[x]subsequent myocardial infarction of other sites	
Aurum and GOLD	194857001	299811016	299811016	45809	G350.00	subsequent myocardial infarction of anterior wall	
Aurum and GOLD	194858006	299812011	299812011	38609	G351.00	subsequent myocardial infarction of inferior wall	
Aurum and GOLD	194861007	299815013	543291000006110	36423	G36..00	certain current complication follow acute myocardial infarct	
Aurum and GOLD	194862000	299816014	813961000006116	24126	G360.00	haemopericardium/current comp folow acut myocard infarct	History
Aurum and GOLD	194866002	299822017	158611000006118	59940	G364.00	ruptur chordae tendinae/curr comp fol acute myocard infarct	
Aurum and GOLD	233838001	350371016	299710011	23892	G304.00	posterior myocardial infarction nos	
Aurum and GOLD	233846000	350381017	67081000006119	37657	G362.00	ventric septal defect/curr comp fol acut myocardal infarctn	History
Aurum and GOLD	233847009	350383019	537751000006115	30421	G30..13	cardiac rupture following myocardial infarction (mi)	
Aurum and GOLD	302049001	1208730013	408571000006116	113358	Gyu3100	[x]other current complicatns following acute myocard infarct	
Aurum and GOLD	304914007	447324018	447324018	30330	G309.00	acute q-wave infarct	
Aurum and GOLD	307140009	450322013	450322013	9507	G307000	acute non-q wave infarction	
Aurum and GOLD	311792005	455418016	212061000006119	46112	G380.00	postoperative transmural myocardial infarction anterior wall	
Aurum and GOLD	311793000	455419012	212071000006114	46276	G381.00	postoperative transmural myocardial infarction inferior wall	
Aurum and GOLD	311796008	455422014	455422014	41835	G384.00	postoperative subendocardial myocardial infarction	
Aurum and GOLD	315287002	459791015	616081000006113	61670	889A.00	diab mellit insulin-glucose infus acute myocardial infarct	
Aurum and GOLD	394659003	1488382011	1488382011	11983	G311500	acute coronary syndrome	
Aurum and GOLD	398274000	1786197015	1786197015	2491	G30..12	coronary thrombosis	
Aurum and GOLD	398274000	1786198013	1786198013	13571	G30..16	thrombosis - coronary	

Aurum and GOLD	401303003	1780491019	1780491019	12229	G30X000	acute st segment elevation myocardial infarction	
Aurum and GOLD	401314000	1780501013	1780501013	10562	G307100	acute non-st segment elevation myocardial infarction	
Aurum and GOLD	52035003	3038718019	299708014	40429	G301000	acute anteroapical infarction	
Aurum and GOLD	54329005	1231324017	299709018	14897	G301z00	anterior myocardial infarction nos	
Aurum and GOLD	54329005	1231324017	299707016	5387	G301.00	other specified anterior myocardial infarction	
Aurum and GOLD	57054005	94884017	299718016	34803	G30y.00	other acute myocardial infarction	
Aurum and GOLD	57054005	94884017	460681000006116	29758	G30X.00	acute transmural myocardial infarction of unspecif site	
Aurum and GOLD	57054005	94884017	362461000006119	96838	Gyu3400	[x]acute transmural myocardial infarction of unspecif site	
Aurum and GOLD	57054005	94884017	94884017	241	G30..00	acute myocardial infarction	
Aurum and GOLD	57054005	94884017	299721019	14658	G30z.00	acute myocardial infarction nos	
Aurum and GOLD	57054005	94884017	299720018	46017	G30yz00	other acute myocardial infarction nos	
Aurum and GOLD	57054005	94884017	219531000000117	1677	G30..15	mi - acute myocardial infarction	
Aurum and GOLD	58612006	1231860015	299711010	14898	G305.00	lateral myocardial infarction nos	
Aurum and GOLD	62695002	104192010	455651000006114	17872	G301100	acute anteroseptal infarction	
Aurum and GOLD	65547006	1232697013	457531000006110	8935	G302.00	acute inferolateral infarction	
Aurum and GOLD	70211005	1233238016	455641000006112	12139	G300.00	acute anterolateral infarction	
Aurum and GOLD	70422006	116992017	116992017	3704	G307.00	acute subendocardial infarction	
Aurum and GOLD	73795002	122557015	299714019	1678	G308.00	inferior myocardial infarction nos	
Aurum and GOLD	76593002	1234005010	1234005010	29643	G303.00	acute inferoposterior infarction	
Aurum and GOLD	79009004	1234306015	1234306015	41221	G30y200	acute septal infarction	

Code list for heart failure – ICD10

ICD10	Description
I11.0	Hypertensive heart disease with (congestive) heart failure
I13.0	Hypertensive heart and renal disease with (congestive) heart failure
I13.2	Hypertensive heart and renal disease with both (congestive) heart failure and renal failure
I50	Heart Failure
I50.0	Congestive Heart Failure
I50.1	Left Ventricular Failure
I50.9	Heart failure, unspecified

Code list for heart failure – Aurum and GOLD

Source	SNOMED_CT_Concept_id	SNOMED_CT_Description_id	Medcode_Aurum	Medcode_GOLD	Readcode	Term
Aurum and GOLD	10633002	18472010	18472010	23707	G580000	acute congestive heart failure
Aurum and GOLD	128404006	206703015	206703015	10079	G580.12	right heart failure
Aurum and GOLD	194779001	299672017	741701000006114	21837	G232.00	hypertensive heart&renal dis wth (congestive) heart failure
Aurum and GOLD	194781004	299674016	789941000006117	57987	G234.00	hyperten heart&renal dis+both(congestv)heart and renal fail
Aurum and GOLD	195111005	300179017	300179017	27884	G580200	decompensated cardiac failure
Aurum and GOLD	195112003	300180019	300180019	11424	G580300	compensated cardiac failure
Aurum and GOLD	195114002	300190010	300190010	5255	G581000	acute left ventricular failure
Aurum and GOLD	233924009	350484012	350484012	66306	SP11111	heart failure as a complication of care
Aurum and GOLD	275514001	411506018	411506018	5942	G581.13	impaired left ventricular function
Aurum and GOLD	367363000	490972013	1816101000006113	104275	G584.00	right ventricular failure
Aurum and GOLD	367363000	490972013	490972013	10154	G580.13	right ventricular failure
Aurum and GOLD	394887005	1488591011	1488591011	21235	1J60.00	suspected heart failure
Aurum and GOLD	395105005	1488804017	1488804017	9913	1O1..00	heart failure confirmed
Aurum and GOLD	416683003	2549208013	2549208013	32898	8H2S.00	admit heart failure emergency
Aurum and GOLD	42343007	70653017	493287011	2906	G580.11	congestive cardiac failure
Aurum and GOLD	42343007	70653017	70653017	398	G580.00	congestive heart failure
Aurum and GOLD	426611007	2675255018	2675255018	94870	G580400	congestive heart failure due to valvular disease
Aurum and GOLD	43736008	72934016	72934016	22262	G1yz100	rheumatic left ventricular failure
Aurum and GOLD	446221000	1713091000000115	1661371000000112	101137	G583.11	hfnef - heart failure with normal ejection fraction
Aurum and GOLD	446221000	2227501000000110	2227501000000110	106897	G583.12	heart failure with preserved ejection fraction
Aurum and GOLD	446221000	2883808011	1647701000000118	101138	G583.00	heart failure with normal ejection fraction
Aurum and GOLD	56675007	94251011	94251011	27964	G582.00	acute heart failure
Aurum and GOLD	84114007	139475013	139475013	2062	G58..00	heart failure
Aurum and GOLD	84114007	139475013	395772015	4024	G58z.00	heart failure nos
Aurum and GOLD	84114007	139482012	139482012	1223	G58..11	cardiac failure
Aurum and GOLD	84114007	139482012	223981000000118	17278	G58z.12	cardiac failure nos

Aurum and GOLD	85232009	141306010	141306010	884	G581.00	left ventricular failure
Aurum and GOLD	88805009	147247018	147247018	32671	G580100	chronic congestive heart failure
Aurum and GOLD	92506005	153058012	510016018	9524	G580.14	biventricular failure
Aurum only	1576321000006109	1576321000006113	1576321000006113			cause of death- congestive cardiac failure
Aurum only	1861731000006105	1861731000006114	1861731000006114			auras-af - consider the patient to have heart failure
Aurum only	905391000006103	905391000006119	905391000006119			[rfc] cardiac failure

Code list for ventricular arrhythmia – ICD10

ICD10	Description
I47.0	Re-entry ventricular arrhythmia
I47.2	Ventricular tachycardia
I49.0	Ventricular fibrillation and flutter
I49.01	Ventricular fibrillation
I49.02	Ventricular flutter
I49.3	Ventricular premature depolarization

Code list for ventricular arrhythmia – Aurum and GOLD

Source	SNOMED_CT_Concept_id	SNOMED_CT_Description_id	Medcode_Aurum	Medcode_GOLD	Readcode	Term	History_only
Aurum and GOLD	111288001	178509016	178509016	5484	G574100	Ventricular flutter	
Aurum and GOLD	161513002	251698019	251698019	22874	14AD.00	H/O ventricular fibrillation	History
Aurum and GOLD	164893009	256482016	256482016	19707	328..00	ECG: ventricular arrhythmia	
Aurum and GOLD	164893009	256482016	256486018	29371	328Z.00	ECG: ventricular arrhythmia NOS	
Aurum and GOLD	164895002	256484015	256484015	4924	3282.00	ECG: ventricular tachycardia	
Aurum and GOLD	164896001	256485019	256485019	31286	3283.00	ECG: ventricular fibrillation	
Aurum and GOLD	17338001	2619422017	2619422017	4802	G576200	Ventricular ectopic beats	
Aurum and GOLD	195083004	300133010	300133010	4374	G574.00	Ventricular fibrillation and flutter	
Aurum and GOLD	195083004	300133010	300134016	41916	G574z00	Ventricular fibrillation and flutter NOS	
Aurum and GOLD	195105007	300168010	300168010	31690	G57yA00	Re-entry ventricular arrhythmia	
Aurum and GOLD	200061000000108	303101000000110	303101000000110	30712	14AP.00	History of ventricular tachycardia	History
Aurum and GOLD	251175005	374330017	374331018	31809	G576500	Ventricular premature depolarization	
Aurum and GOLD	25569003	42864016	42864016	7794	G571.11	Ventricular tachycardia	
Aurum and GOLD	66657009	110733013	110733013	3418	G571.00	Paroxysmal ventricular tachycardia	
Aurum and GOLD	71908006	119481012	119481012	4827	G574000	Ventricular fibrillation	
Aurum and GOLD	71908006	1233433010	537191000006113	25583	G574011	Cardiac arrest-ventricular fibrillation	
Aurum only	66657009	884371000006114	884371000006114		G571.99	Paroxysmal ventric. tachyc.	

Code list for fracture – ICD10

ICD10	Description	History_only
M48.4	Fatigue fracture of vertebra	
M80	Osteoporosis with pathological fracture	
M80.1	Postoophorectomy osteoporosis with pathological fracture	
M80.2	Osteoporosis of disuse with pathological fracture	
M80.3	Postsurgical malabsorption osteoporosis with pathological fracture	
M80.4	Drug-induced osteoporosis with pathological fracture	
M80.5	Idiopathic osteoporosis with pathological fracture	
M80.8	Other osteoporosis with pathological fracture	
M80.9	Unspecified osteoporosis with pathological fracture	
S02	fracture of skull and facial bones	
S02.0	fracture of vault of skull	
S02.00	fracture of vault of skull	
S02.01	fracture of vault of skull	
S02.1	fracture of base of skull	
S02.10	fracture of base of skull	
S02.11	fracture of base of skull	
S02.2	fracture of nasal bones	
S02.20	fracture of nasal bones	
S02.21	fracture of nasal bones	
S02.3	fracture of orbital floor	
S02.30	fracture of orbital floor	
S02.31	fracture of orbital floor	
S02.4	fracture of malar and maxillary bones	
S02.40	fracture of malar and maxillary bones	
S02.41	fracture of malar and maxillary bones	
S02.5	fracture of tooth	
S02.50	fracture of tooth	
S02.51	fracture of tooth	
S02.6	fracture of mandible	
S02.60	fracture of mandible	
S02.61	fracture of mandible	
S02.7	multiple fractures involving skull and facial bones	
S02.70	multiple fractures involving skull and facial bones	
S02.71	multiple fractures involving skull and facial bones	
S02.8	fractures of other skull and facial bones	
S02.80	fractures of other skull and facial bones	
S02.81	fractures of other skull and facial bones	
S02.9	fracture of skull and facial bones, part unspecified	
S02.90	fracture of skull and facial bones, part unspecified	
S02.91	fracture of skull and facial bones, part unspecified	
S12	fracture of neck	
S12.0	fracture of first cervical vertebra	
S12.00	fracture of first cervical vertebra	
S12.01	fracture of first cervical vertebra	

S12.1	fracture of second cervical vertebra	
S12.10	fracture of second cervical vertebra	
S12.11	fracture of second cervical vertebra	
S12.2	fracture of other specified cervical vertebra	
S12.20	fracture of other specified cervical vertebra	
S12.21	fracture of other specified cervical vertebra	
S12.7	multiple fractures of cervical spine	
S12.70	multiple fractures of cervical spine	
S12.71	multiple fractures of cervical spine	
S12.8	fracture of other parts of neck	
S12.80	fracture of other parts of neck	
S12.81	fracture of other parts of neck	
S12.9	fracture of neck, part unspecified	
S12.90	fracture of neck, part unspecified	
S12.91	fracture of neck, part unspecified	
S22	fracture of rib(s), sternum and thoracic spine	
S22.0	fracture of thoracic vertebra	
S22.00	fracture of thoracic vertebra	
S22.01	fracture of thoracic vertebra	
S22.1	multiple fractures of thoracic spine	
S22.10	multiple fractures of thoracic spine	
S22.11	multiple fractures of thoracic spine	
S22.2	fracture of sternum	
S22.20	fracture of sternum	
S22.21	fracture of sternum	
S22.3	fracture of rib	
S22.30	fracture of rib	
S22.31	fracture of rib	
S22.4	multiple fractures of ribs	
S22.40	multiple fractures of ribs	
S22.41	multiple fractures of ribs	
S22.5	flail chest	
S22.50	flail chest	
S22.51	flail chest	
S22.8	fracture of other parts of bony thorax	
S22.80	fracture of other parts of bony thorax	
S22.81	fracture of other parts of bony thorax	
S22.9	fracture of bony thorax, part unspecified	
S22.90	fracture of bony thorax, part unspecified	
S22.91	fracture of bony thorax, part unspecified	
S32	fracture of lumbar spine and pelvis	
S32.0	fracture of lumbar vertebra	
S32.00	fracture of lumbar vertebra	
S32.01	fracture of lumbar vertebra	
S32.1	fracture of sacrum	
S32.10	fracture of sacrum	
S32.11	fracture of sacrum	

S32.2	fracture of coccyx	
S32.20	fracture of coccyx	
S32.21	fracture of coccyx	
S32.3	fracture of ilium	
S32.30	fracture of ilium	
S32.31	fracture of ilium	
S32.4	fracture of acetabulum	
S32.40	fracture of acetabulum	
S32.41	fracture of acetabulum	
S32.5	fracture of pubis	
S32.50	fracture of pubis	
S32.51	fracture of pubis	
S32.7	multiple fractures of lumbar spine and pelvis	
S32.70	multiple fractures of lumbar spine and pelvis	
S32.71	multiple fractures of lumbar spine and pelvis	
S32.8	fracture of other and unspecified parts of lumbar spine and pelvis	
S32.80	fracture of other and unspecified parts of lumbar spine and pelvis	
S32.81	fracture of other and unspecified parts of lumbar spine and pelvis	
S42	fracture of shoulder and upper arm	
S42.0	fracture of clavicle	
S42.00	fracture of clavicle	
S42.01	fracture of clavicle	
S42.1	fracture of scapula	
S42.10	fracture of scapula	
S42.11	fracture of scapula	
S42.2	fracture of upper end of humerus	
S42.20	fracture of upper end of humerus	
S42.21	fracture of upper end of humerus	
S42.3	fracture of shaft of humerus	
S42.30	fracture of shaft of humerus	
S42.31	fracture of shaft of humerus	
S42.4	fracture of lower end of humerus	
S42.40	fracture of lower end of humerus	
S42.41	fracture of lower end of humerus	
S42.7	multiple fractures of clavicle, scapula and humerus	
S42.70	multiple fractures of clavicle, scapula and humerus	
S42.71	multiple fractures of clavicle, scapula and humerus	
S42.8	fracture of other parts of shoulder and upper arm	
S42.80	fracture of other parts of shoulder and upper arm	
S42.81	fracture of other parts of shoulder and upper arm	
S42.9	fracture of shoulder girdle, part unspecified	
S42.90	fracture of shoulder girdle, part unspecified	
S42.91	fracture of shoulder girdle, part unspecified	
S52	fracture of forearm	
S52.0	fracture of upper end of ulna	
S52.00	fracture of upper end of ulna	
S52.01	fracture of upper end of ulna	

S52.1	fracture of upper end of radius	
S52.10	fracture of upper end of radius	
S52.11	fracture of upper end of radius	
S52.2	fracture of shaft of ulna	
S52.20	fracture of shaft of ulna	
S52.21	fracture of shaft of ulna	
S52.3	fracture of shaft of radius	
S52.30	fracture of shaft of radius	
S52.31	fracture of shaft of radius	
S52.4	fracture of shafts of both ulna and radius	
S52.40	fracture of shafts of both ulna and radius	
S52.41	fracture of shafts of both ulna and radius	
S52.5	fracture of lower end of radius	
S52.50	fracture of lower end of radius	
S52.51	fracture of lower end of radius	
S52.6	fracture of lower end of both ulna and radius	
S52.60	fracture of lower end of both ulna and radius	
S52.61	fracture of lower end of both ulna and radius	
S52.7	multiple fractures of forearm	
S52.70	multiple fractures of forearm	
S52.71	multiple fractures of forearm	
S52.8	fracture of other parts of forearm	
S52.80	fracture of other parts of forearm	
S52.81	fracture of other parts of forearm	
S52.9	fracture of forearm, part unspecified	
S52.90	fracture of forearm, part unspecified	
S52.91	fracture of forearm, part unspecified	
S62	fracture at wrist and hand level	
S62.0	fracture of navicular [scaphoid] bone of hand	
S62.00	fracture of navicular [scaphoid] bone of hand	
S62.01	fracture of navicular [scaphoid] bone of hand	
S62.1	fracture of other carpal bone(s)	
S62.10	fracture of other carpal bone(s)	
S62.11	fracture of other carpal bone(s)	
S62.2	fracture of first metacarpal bone	
S62.20	fracture of first metacarpal bone	
S62.21	fracture of first metacarpal bone	
S62.3	fracture of other metacarpal bone	
S62.30	fracture of other metacarpal bone	
S62.31	fracture of other metacarpal bone	
S62.4	multiple fractures of metacarpal bones	
S62.40	multiple fractures of metacarpal bones	
S62.41	multiple fractures of metacarpal bones	
S62.5	fracture of thumb	
S62.50	fracture of thumb	
S62.51	fracture of thumb	
S62.6	fracture of other finger	

S62.60	fracture of other finger	
S62.61	fracture of other finger	
S62.7	multiple fractures of fingers	
S62.70	multiple fractures of fingers	
S62.71	multiple fractures of fingers	
S62.8	fracture of other and unspecified parts of wrist and hand	
S62.80	fracture of other and unspecified parts of wrist and hand	
S62.81	fracture of other and unspecified parts of wrist and hand	
S72	fracture of femur	
S72.0	fracture of neck of femur	
S72.00	fracture of neck of femur	
S72.01	fracture of neck of femur	
S72.1	pertrochanteric fracture	
S72.10	pertrochanteric fracture	
S72.11	pertrochanteric fracture	
S72.2	subtrochanteric fracture	
S72.20	subtrochanteric fracture	
S72.21	subtrochanteric fracture	
S72.3	fracture of shaft of femur	
S72.30	fracture of shaft of femur	
S72.31	fracture of shaft of femur	
S72.4	fracture of lower end of femur	
S72.40	fracture of lower end of femur	
S72.41	fracture of lower end of femur	
S72.7	multiple fractures of femur	
S72.70	multiple fractures of femur	
S72.71	multiple fractures of femur	
S72.8	fractures of other parts of femur	
S72.80	fractures of other parts of femur	
S72.81	fractures of other parts of femur	
S72.9	fracture of femur, part unspecified	
S72.90	fracture of femur, part unspecified	
S72.91	fracture of femur, part unspecified	
S82	fracture of lower leg, including ankle	
S82.0	fracture of patella	
S82.00	fracture of patella	
S82.01	fracture of patella	
S82.1	fracture of upper end of tibia	
S82.10	fracture of upper end of tibia	
S82.11	fracture of upper end of tibia	
S82.2	fracture of shaft of tibia	
S82.20	fracture of shaft of tibia	
S82.21	fracture of shaft of tibia	
S82.3	fracture of lower end of tibia	
S82.30	fracture of lower end of tibia	
S82.31	fracture of lower end of tibia	
S82.4	fracture of fibula alone	

S82.40	fracture of fibula alone	
S82.41	fracture of fibula alone	
S82.5	fracture of medial malleolus	
S82.50	fracture of medial malleolus	
S82.51	fracture of medial malleolus	
S82.6	fracture of lateral malleolus	
S82.60	fracture of lateral malleolus	
S82.61	fracture of lateral malleolus	
S82.7	multiple fractures of lower leg	
S82.70	multiple fractures of lower leg	
S82.71	multiple fractures of lower leg	
S82.8	fractures of other parts of lower leg	
S82.80	fractures of other parts of lower leg	
S82.81	fractures of other parts of lower leg	
S82.9	fracture of lower leg, part unspecified	
S82.90	fracture of lower leg, part unspecified	
S82.91	fracture of lower leg, part unspecified	
S92	fracture of foot, except ankle	
S92.0	fracture of calcaneus	
S92.00	fracture of calcaneus	
S92.01	fracture of calcaneus	
S92.1	fracture of talus	
S92.10	fracture of talus	
S92.11	fracture of talus	
S92.2	fracture of other tarsal bone(s)	
S92.20	fracture of other tarsal bone(s)	
S92.21	fracture of other tarsal bone(s)	
S92.3	fracture of metatarsal bone	
S92.30	fracture of metatarsal bone	
S92.31	fracture of metatarsal bone	
S92.4	fracture of great toe	
S92.40	fracture of great toe	
S92.41	fracture of great toe	
S92.5	fracture of other toe	
S92.50	fracture of other toe	
S92.51	fracture of other toe	
S92.7	multiple fractures of foot	
S92.70	multiple fractures of foot	
S92.71	multiple fractures of foot	
S92.9	fracture of foot, unspecified	
S92.90	fracture of foot, unspecified	
S92.91	fracture of foot, unspecified	
T02	Fractures involving multiple body regions	
T02.0	Fractures involving head with neck	
T02.1	Fractures involving thorax with lower back and pelvis	
T02.2	Fractures involving multiple regions of one upper limb	
T02.3	Fractures involving multiple regions of one lower limb	

T02.4	Fractures involving multiple regions of both upper limbs	
T02.5	Fractures involving multiple regions of both lower limbs	
T02.6	Fractures involving multiple regions of upper limb(s) with lower limb(s)	
T02.7	Fractures involving thorax with lower back and pelvis with limb(s)	
T02.8	Fractures involving other combinations of body regions	
T02.9	Multiple fractures, unspecified	
T08	Fracture of spine, level unspecified	
T10	Fracture of upper limb, level unspecified	
T12	Fracture of lower limb, level unspecified	
T14.2	Fracture of unspecified body region	
T90.2	Sequelae of fracture of skull and facial bones	History
T91.1	Sequelae of fracture of spine	History
T91.2	Sequelae of other fracture of thorax and pelvis	History
T92.1	Sequelae of fracture of arm	History
T92.2	Sequelae of fracture at wrist and hand level	History
T93.1	Sequelae of fracture of femur	History
T93.2	Sequelae of other fractures of lower limb	History

Code list for fracture – Aurum and GOLD

Source	SNOMED_CT_Concept_id	SNOMED_CT_Description_id	Medcode_Aurum	Medcode_GOLD	Readcode	Term	History_only
Aurum only	10568008	18370015	18370015		S121800	open fracture of eight or more ribs	
Aurum only	111609001	178856011	178856011			open fracture of facial bones	
Aurum only	11782000	20339011	391330014		S100.12	closed fracture of axis without spinal cord lesion	
Aurum only	125605004	895241000006111	895241000006111		S02z.99	#facial bones nos	
Aurum only	14457000	1221098013	1221098013		S126000	open fracture larynx	
Aurum only	15385006	989581000006112	989581000006112		S340.99	#ankle - medial malleolus	
Aurum only	16114001	895811000006111	895811000006111		S34..94	#ankle - trimalleolar	
Aurum only	16114001	895821000006115	895821000006115		S34..95	#ankle - bimalleolar - potts #	
Aurum only	16114001	895831000006117	895831000006117		S34..96	#ankle - lateral malleolus	
Aurum only	16114001	895841000006110	895841000006110		S34..97	#ankle - medial malleolus	
Aurum only	16114001	895851000006112	895851000006112		S34..98	#ankle	
Aurum only	16114001	895861000006114	895861000006114		S34..99	fracture left ankle	
Aurum only	16114001	991041000006113	991041000006113		S34..93	#ankle - nos	
Aurum only	1726531000006101	1726531000006117	1726531000006117			closed fracture of tibia and fibula, distal	
Aurum only	1726541000006106	1726541000006110	1726541000006110			open fracture of tibia and fibula, distal	
Aurum only	1727761000006100	1727761000006116	1727761000006116			fracture of tibial plateau	
Aurum only	1727771000006107	1727771000006111	1727771000006111			open fracture of tibial plateau	
Aurum only	176635002	875651000006115	875651000006115		7K1J.99	closed # reduct+internal fixat	
Aurum only	178397008	874751000006112	874751000006112		7J03298	open reduction - zygomatic #	
Aurum only	178397008	874761000006114	874761000006114		7J03299	closed reduction - zygomatic #	
Aurum only	178436001	988351000006116	988351000006116		7J12099	open reduction - alveolar #	
Aurum only	178442002	874931000006115	874931000006115		7J13099	open reduction - alveolar #	
Aurum only	178445000	874961000006112	874961000006112		7J13399	open reduction - orbital #	
Aurum only	178496003	875011000006114	875011000006114		7J17799	traction for # jaw	
Aurum only	178690000	276491016	276491016		7J42800	primary skull traction stabilisation of spinal fracture	

Aurum only	178692008	276495013	276495013		7J42A00	primary external fixation stabilisation of spinal fracture	
Aurum only	178696006	276502015	276502015		7J42E00	revision to skull traction stabilisation of spinal fracture	History
Aurum only	178697002	276505018	276505018		7J42F00	revision to cast stabilisation of spinal fracture	History
Aurum only	178701004	276512010	210131000006113		7J42K00	primary cls reduction spinal fracture+collar stabilisation	
Aurum only	178704007	276517016	210121000006110		7J42N00	primary cls reduction spinal fracture+cast stabilisation	
Aurum only	178705008	276519018	210141000006115		7J42P00	primary cls reduction spinal fracture+external fixation	
Aurum only	178708005	276524015	171491000006118		7J42S00	revision cls reduction spinal fracture+collar stabilisation	History
Aurum only	178709002	276525019	171401000006113		7J42T00	revision cls reduc spinal fracture+bedrest stabilisation	History
Aurum only	178710007	276528017	171441000006110		7J42U00	revision cls reduc spinal fracture+skull traction stabilistn	History
Aurum only	178711006	276529013	171411000006111		7J42V00	revision cls reduc spinal fracture+cast stabilisation	History
Aurum only	178712004	276531016	171421000006115		7J42W00	revision cls reduc spinal fracture+external fixation	History
Aurum only	178715002	276536014	205491000006111		7J42Z00	primary opn reduction spinal fracture+collar stabilisation	
Aurum only	178716001	276537017	205471000006110		7J42a00	primary opn reduction spinal fracture+bedrest stabilisation	
Aurum only	178717005	276540017	205451000006117		7J42b00	primary opn reduc spinal fracture+skull traction stabilisatn	
Aurum only	178718000	276541018	205481000006113		7J42c00	primary opn reduction spinal fracture+cast stabilisation	
Aurum only	178719008	276543015	276543015		7J42d00	primary open reduction spinal fracture and external fixation	
Aurum only	178722005	276548012	159391000006112		7J42g00	rvsn open reduc spinal fracture+collar stabilisation	History
Aurum only	178723000	276549016	159371000006111		7J42h00	rvsn open reduc spinal fracture+bedrest stabilisation	History

Aurum only	178724006	276552012	159471000006115		7J42j00	rvsn open reduc spinal fracture+skull traction stabilisation	History
Aurum only	178725007	276553019	159381000006114		7J42k00	rvsn open reduc spinal fracture+cast stabilisation	History
Aurum only	178726008	276555014	159401000006114		7J42l00	rvsn open reduc spinal fracture+external fix	History
Aurum only	178741008	276576016	159441000006111		7J43800	rvsn open reduc spinal fracture+internal fix+wire	History
Aurum only	178744000	276579011	159361000006116		7J43B00	rvsn open reduc spinal #+internal fix+segmental wire system	History
Aurum only	178926002	875411000006118	875411000006118		7K14.99	refracture of bone-osteoclasis	
Aurum only	179058004	277069012	151851000006112		7K1H300	secondary open reduct fracture bone & skeletal traction hfq	
Aurum only	179085006	277101010	166881000006119		7K1HR00	revision to open red #+int fxn+multiple implant types	History
Aurum only	179139006	277159015	166801000006111		7K1K600	revision to closed reduction+ext fxn proximal femoral #	History
Aurum only	179146002	277168018	277168018		7K1KD00	primary closed reduction of fracture and functional bracing	
Aurum only	179148001	277170010	166671000006116		7K1KF00	revision to closed reduction of # and functional bracing	History
Aurum only	179161001	875681000006111	875681000006111		7K1L799	closed reduction-#tibia/fibula	
Aurum only	179163003	875691000006114	875691000006114		7K1L999	closed reduction-# metatarsus	
Aurum only	179171004	875721000006116	875721000006116		7K1LK99	closed reduction- # metacarpus	
Aurum only	179172006	875731000006118	875731000006118		7K1LL99	closed reduction-# radius/ulna	
Aurum only	179182007	277212015	277212015		7K1LY00	revision to closed reduction of fracture and skin traction	History
Aurum only	179213000	277260017	277260017		7K1NA00	revision to skeletal traction of fracture	History
Aurum only	179615002	277904015	205111000006113		7K6FH00	primary open reduction of # dislocation+skin traction	
Aurum only	179647007	277943016	209801000006116		7K6GZ00	primary closed reduction of # dislocation+functional bracing	
Aurum only	179648002	277944010	209811000006118		7K6Ga00	primary closed reduction of # dislocation+skin traction	

Aurum only	179680000	277985017	166691000006115		7K6HQ00	revision to closed reduction of # dislocation+cast immobil	History
Aurum only	179681001	277987013	166541000006111		7K6HR00	revision to closed reduction # dislocation+function bracing	History
Aurum only	179682008	277988015	166711000006117		7K6HS00	revision to closed reduction of # dislocation+skin traction	History
Aurum only	179683003	277989011	166561000006110		7K6HT00	revision to closed reduction # dislocation+skeletal traction	History
Aurum only	179684009	277990019	166531000006118		7K6HU00	revision to closed reduction # dislocation+fixation+wire(s)	History
Aurum only	179685005	277991015	166701000006115		7K6HV00	revision to closed reduction of # dislocation+ext fixation	History
Aurum only	179688007	277996013	167131000006113		7K6HY00	revision to open reduction of # dislocation+cast immobil	History
Aurum only	179689004	277997016	167031000006112		7K6HZ00	revision to open reduction # dislocation+functional bracing	History
Aurum only	179690008	277998014	167151000006118		7K6Ha00	revision to open reduction of # dislocation+skin traction	History
Aurum only	179692000	278000012	167001000006116		7K6Hc00	revision to open reduction # dislocation+external fixation	History
Aurum only	179695003	278004015	167011000006118		7K6Hf00	revision to open reduction # dislocation+fixation+plate(s)	History
Aurum only	1856821000006103	1856821000006119	1856821000006119			non fragility fracture	
Aurum only	19259001	895531000006115	895531000006115		S230.99	#radius/ulna - upper end	
Aurum only	1954391000006105	1954391000006114	1954391000006114			fracture/ dislocation (primary group)	
Aurum only	1954591000006101	1954591000006117	1954591000006117			closed fracture (secondary group)	
Aurum only	1954601000006109	1954601000006113	1954601000006113			open fracture (secondary group)	
Aurum only	20013001	874711000006111	874711000006111		7J03.97	facial # - open reduction nos	
Aurum only	20013001	874721000006115	874721000006115		7J03.98	facial # -closed reduction nos	
Aurum only	20013001	874731000006117	874731000006117		7J03.99	reduction of facial #	
Aurum only	20013001	991591000006110	991591000006110		7J03.96	facial # - reduction nos	

Aurum only	203449003	312161016	387551000006116		NyuCE00	[x]fracture of bone in neoplastic diseases ce	
Aurum only	207693007	318123013	560271000006112		S001500	closed #skull vlt + intracranial inj, >24hr loc not restored	
Aurum only	207696004	318126017	263821000006111		S002200	open #skull vlt no intracranial injury, <1hr loss of consc	
Aurum only	207696004	318126017	263801000006118		S002500	open #skull vlt no intracranial inj, >24hr loc not restored	
Aurum only	207696004	318126017	263831000006114		S002400	open #skull vlt no intracranial injury, >24hr loc+recovery	
Aurum only	207696004	318126017	263811000006115		S002z00	open #skull vlt no intracranial injury + concussion unspec	
Aurum only	207696004	318126017	263841000006116		S002300	open #skull vlt no intracranial injury, 1-24hr loss of consc	
Aurum only	207696004	318126017	263851000006119		S002600	open #skull vlt no intracranial injury, loc unspec duration	
Aurum only	207708000	318138014	264431000006110		S003200	open #skull vlt + intracranial injury, <1hr loss of consc	
Aurum only	207709008	318139018	52941000006118		S003300	open #skull vlt + intracranial injury, 1-24hr loss of consc	
Aurum only	207711004	318141017	264421000006112		S003500	open #skull vlt + intracranial inj, >24hr loc not restored	
Aurum only	207731000	318175016	559951000006113		S011500	closed #skull bse + intracranial inj, >24hr loc not restored	
Aurum only	207745006	318189014	264161000006114		S013100	open #skull bse + intracranial injury, no loss of consc	
Aurum only	207747003	318191018	264141000006110		S013300	open #skull bse + intracranial injury, 1-24hr loss of consc	
Aurum only	207749000	318193015	265451000006116		S013500	open #skull bse + intracranial inj, >24hr loc not restored	
Aurum only	207753003	895201000006114	895201000006114		S022.99	#mandible	
Aurum only	207902006	318382014	318382014		S100F00	closed fracture axis, tricolumnar	
Aurum only	207909002	318389017	318389017		S100N00	closed fracture cervical vertebra, tricolumnar	

Aurum only	207921003	318418017	318418017		S101800	open fracture atlas, isolated arch or articular process	
Aurum only	207924006	318421015	318421015		S101B00	open fracture axis, spondylolysis	
Aurum only	207925007	318422010	318422010		S101C00	open fracture axis, spinous process	
Aurum only	207927004	318424011	318424011		S101E00	open fracture axis, posterior arch	
Aurum only	207928009	318425012	318425012		S101F00	open fracture axis, tricolumnar	
Aurum only	207929001	318426013	318426013		S101G00	open fracture cervical vertebra, burst	
Aurum only	207930006	318427016	318427016		S101H00	open fracture cervical vertebra, wedge	
Aurum only	207931005	318428014	318428014		S101J00	open fracture cervical vertebra, spondylolysis	
Aurum only	207932003	318429018	318429018		S101K00	open fracture cervical vertebra, spinous process	
Aurum only	207933008	318430011	318430011		S101L00	open fracture cervical vertebra, transverse process	
Aurum only	207934002	318431010	318431010		S101M00	open fracture cervical vertebra, posterior arch	
Aurum only	207935001	318432015	318432015		S101N00	open fracture cervical vertebra, tricolumnar	
Aurum only	207938004	895281000006117	895281000006117		S102.99	#thoracic spine-no cord lesion	
Aurum only	207945004	318442018	318442018		S102600	closed fracture thoracic vertebra, tricolumnar	
Aurum only	207950005	318447012	318447012		S103000	open fracture thoracic vertebra, burst	
Aurum only	207952002	318449010	318449010		S103200	open fracture thoracic vertebra, spondylolysis	
Aurum only	207953007	318450010	318450010		S103300	open fracture thoracic vertebra, spinous process	
Aurum only	207954001	318451014	318451014		S103400	open fracture thoracic vertebra, transverse process	
Aurum only	207956004	318453012	318453012		S103600	open fracture thoracic vertebra, tricolumnar	
Aurum only	207957008	895291000006119	895291000006119		S104.99	#lumbar spine - no cord lesion	
Aurum only	207968008	318465011	318465011		S105200	open fracture lumbar vertebra, spondylolysis	
Aurum only	207969000	318466012	318466012		S105300	open fracture lumbar vertebra, spinous process	
Aurum only	207971000	318468013	318468013		S105500	open fracture lumbar vertebra, posterior arch	
Aurum only	207972007	318469017	318469017		S105600	open fracture lumbar vertebra, tricolumnar	
Aurum only	207974008	895301000006118	895301000006118		S106.99	#sacrum/coccyx-no cord lesion	

Aurum only	207998001	895321000006111	895321000006111		S110.99	#cervical spine + cord lesion	
Aurum only	208001004	318503015	570671000006117		S110200	cls spinal fracture with anterior cervcl cord lesion, c1-4	
Aurum only	208002006	318504014	570701000006116		S110300	cls spinal fracture with central cervical cord lesion, c1-4	
Aurum only	208003001	318505010	570771000006110		S110400	cls spinal fracture with posterior cervcl cord lesion, c1-4	
Aurum only	208008005	318510014	570711000006118		S110900	cls spinal fracture with central cervical cord lesion, c5-7	
Aurum only	208009002	318511013	570781000006113		S110A00	cls spinal fracture with posterior cervcl cord lesion, c5-7	
Aurum only	208012004	318514017	318527018		S111z00	open fracture of cervical spine with spinal cord lesion nos	
Aurum only	208014003	318516015	44451000006118		S111100	opn spinal fracture with complete cervcl cord lesion, c1-4	
Aurum only	208015002	318517012	44411000006119		S111200	opn spinal fracture with anterior cervcl cord lesion, c1-4	
Aurum only	208016001	318518019	318518019		S111300	open spinal fracture with central cervical cord lesion, c1-4	
Aurum only	208017005	318519010	43161000006114		S111400	opn spinal fracture with posterior cervcl cord lesion, c1-4	
Aurum only	208020002	318522012	44461000006116		S111700	opn spinal fracture with complete cervcl cord lesion, c5-7	
Aurum only	208021003	318523019	44421000006110		S111800	opn spinal fracture with anterior cervcl cord lesion, c5-7	
Aurum only	208022005	318524013	318524013		S111900	open spinal fracture with central cervical cord lesion, c5-7	
Aurum only	208023000	318525014	43171000006119		S111A00	opn spinal fracture with posterior cervcl cord lesion, c5-7	
Aurum only	208026008	895331000006114	895331000006114		S112.99	#thoracic spine + cord lesion	
Aurum only	208029001	318531012	570851000006111		S112200	cls spinal fracture wth anterior thoracic cord lesion,t1-6	

Aurum only	208030006	318532017	570721000006114		S112300	cls spinal fracture with central thoracic cord lesion, t1-6	
Aurum only	208031005	318533010	570791000006111		S112400	cls spinal fracture with posterior thorac cord lesion, t1-6	
Aurum only	208035001	318537011	570691000006116		S112800	cls spinal fracture with anterior thorac cord lesion, t7-12	
Aurum only	208040009	318542015	318555019		S113z00	open fracture of thoracic spine with spinal cord lesion nos	
Aurum only	208042001	318544019	43141000006110		S113100	opn spinal fracture with complete thorac cord lesion, t1-6	
Aurum only	208043006	318545018	44431000006113		S113200	opn spinal fracture with anterior thorac cord lesion, t1-6	
Aurum only	208044000	318546017	318546017		S113300	open spinal fracture with central thoracic cord lesion, t1-6	
Aurum only	208045004	318547014	43181000006116		S113400	opn spinal fracture with posterior thorac cord lesion, t1-6	
Aurum only	208048002	318550012	43151000006112		S113700	opn spinal fracture with complete thorac cord lesion, t7-12	
Aurum only	208049005	318551011	44441000006115		S113800	opn spinal fracture with anterior thorac cord lesion, t7-12	
Aurum only	208050005	318552016	265391000006117		S113900	op spinal fracture with central thoracic cord lesion, t7-12	
Aurum only	208054001	895341000006116	895341000006116		S114.99	#lumbar spine + cord lesion	
Aurum only	208057008	318559013	318559013		S114200	closed spinal fracture with anterior lumbar cord lesion	
Aurum only	208058003	318560015	318560015		S114300	closed spinal fracture with central lumbar cord lesion	
Aurum only	208059006	318561016	318561016		S114400	closed spinal fracture with posterior lumbar cord lesion	
Aurum only	208063004	318565013	318565013		S115100	open spinal fracture with complete lumbar cord lesion	
Aurum only	208064005	318566014	318566014		S115200	open spinal fracture with anterior lumbar cord lesion	

Aurum only	208065006	318567017	318567017		S115300	open spinal fracture with central lumbar cord lesion	
Aurum only	208066007	318568010	318568010		S115400	open spinal fracture with posterior lumbar cord lesion	
Aurum only	208067003	318569019	318569019		S115500	open spinal fracture with cauda equina lesion	
Aurum only	208069000	318571019	318574010		S116200	closed fracture of sacrum with other cauda equina injury	
Aurum only	208069000	318571019	318575011		S116300	closed fracture of sacrum with other spinal cord injury	
Aurum only	208069000	318571019	563691000006115		S116000	closed fracture of sacrum with unspec spinal cord lesion	
Aurum only	208069000	895351000006119	895351000006119		S116.99	#sacrum/coccyx + cord lesion	
Aurum only	208071000	318573016	318573016		S116100	closed fracture of sacrum with complete cauda equina lesion	
Aurum only	208076005	318581015	318582010		S117000	open fracture of sacrum with unspecified spinal cord lesion	
Aurum only	208076005	318581015	318586013		S117z00	open fracture of sacrum with spinal cord lesion nos	
Aurum only	208076005	318581015	318584011		S117200	open fracture of sacrum with other cauda equina injury	
Aurum only	208078006	318583017	318583017		S117100	open fracture of sacrum with complete cauda equina lesion	
Aurum only	208082008	318587016	318591014		S118300	closed fracture of coccyx with other spinal cord injury	
Aurum only	208082008	318587016	562841000006118		S118000	closed fracture of coccyx with unspec spinal cord lesion	
Aurum only	208084009	318589018	318589018		S118100	closed fracture of coccyx with complete cauda equina lesion	
Aurum only	208088007	318593012	318596016		S119200	open fracture of coccyx with other cauda equina injury	
Aurum only	208088007	318593012	318594018		S119000	open fracture of coccyx with unspecified spinal cord lesion	
Aurum only	208088007	318593012	318593012		S119.00	open fracture of coccyx with spinal cord lesion	

Aurum only	208088007	318593012	318597013		S119300	open fracture of coccyx with other spinal cord injury	
Aurum only	208088007	318593012	318598015		S119z00	open fracture of coccyx with spinal cord lesion nos	
Aurum only	208090008	318595017	318595017		S119100	open fracture of coccyx with complete cauda equina lesion	
Aurum only	208122008	1489806012	318632014		S125z00	closed fracture of larynx and trachea nos	
Aurum only	208122008	895401000006110	895401000006110		S125.99	#larynx/trachea	
Aurum only	208128007	318633016	318633016		S126.00	open fracture larynx and trachea	
Aurum only	208128007	318633016	318638013		S126z00	open fracture of larynx and trachea nos	
Aurum only	208151007	318660011	318660011		S130500	closed fracture acetabulum, double column transverse	
Aurum only	208155003	318664019	318664019		S131000	open fracture acetabulum, anterior lip alone	
Aurum only	208156002	318665018	318665018		S131100	open fracture acetabulum, posterior lip alone	
Aurum only	208157006	318666017	318666017		S131200	open fracture acetabulum, anterior column	
Aurum only	208158001	318667014	318667014		S131300	open fracture acetabulum, posterior column	
Aurum only	208159009	318668016	318668016		S131400	open fracture acetabulum, floor	
Aurum only	208160004	318669012	318669012		S131500	open fracture acetabulum, double column transverse	
Aurum only	208164008	895431000006119	895431000006119		S132.99	#pubis of pelvis	
Aurum only	208197009	318707016	318707016		S135700	open vertical fracture of ilium	
Aurum only	208235007	318746019	318746019		S211500	open fracture scapula, spine	
Aurum only	208342009	318883012	49861000006117		S235912	open volar barton fracture-subluxation	
Aurum only	208342009	318884018	49851000006119		S235911	open volar barton fracture-dislocation	
Aurum only	208343004	318885017	261541000006119		S235A11	open dorsal barton's fracture-dislocation	
Aurum only	208343004	318886016	261551000006117		S235A12	open dorsal barton's fracture-subluxation	
Aurum only	208385000	318933018	318933018		S241F00	open fracture carpal bones, multiple	
Aurum only	208393000	895621000006119	895621000006119		S25..99	#metacarpal bones	
Aurum only	208393000	989511000006117	989511000006117		S25..98	#metacarpal bone nos	
Aurum only	208396008	895631000006116	895631000006116		S250199	#bennett's fracture	
Aurum only	208430000	895661000006113	895661000006113		S260.98	#phalanx of thumb	

Aurum only	208430000	895671000006118	895671000006118		S260.99	#phalanx of finger	
Aurum only	208590001	319175012	319175012		S313600	open fracture distal femur, bicondylar (t-y fracture)	
Aurum only	208605007	319192015	319192015		S321300	open fracture patella, vertical	
Aurum only	208629000	989571000006114	989571000006114		S332299	#tibia/fibula - shaft	
Aurum only	208679002	319285015	319285015		S351000	open fractures calcaneus, extra-articular	
Aurum only	209248000	319945018	255601000006111		S4A3000	open fracture-subluxation shoulder joint	
Aurum only	209261000	319958017	256261000006119		S4B3000	open fracture-subluxation elbow joint	
Aurum only	209262007	319959013	255631000006115		S4B3100	open fracture-subluxation superior radio-ulnar joint	
Aurum only	209273000	319970018	255971000006112		S4C1y00	open fracture-dislocation other carpal	
Aurum only	209277004	319975011	256741000006114		S4C1200	open fracture-dislocation mid carpal	
Aurum only	209279001	319977015	256731000006116		S4C1400	open fracture-dislocation lunate (volar)	
Aurum only	209280003	319978013	255981000006110		S4C1500	open fracture-dislocation peri-lunate (dorsal)	
Aurum only	209289002	319987016	565691000006116		S4C2500	closed fracture-subluxation peri-lunate (dorsal)	
Aurum only	209295001	319993012	256291000006110		S4C3200	open fracture-subluxation mid carpal	
Aurum only	209298004	319996016	255561000006111		S4C3500	open fracture-subluxation peri-lunate (dorsal)	
Aurum only	209326009	320024017	256241000006118		S4D3000	open fracture-subluxation digit, unspecified	
Aurum only	209338000	320036011	255691000006116		S4E3.00	open fracture-subluxation, hip joint	
Aurum only	209380001	320081010	255791000006111		S4H3000	open fracture-subluxation, subtalar joint	
Aurum only	209381002	320082015	255761000006115		S4H3100	open fracture-subluxation, midtarsal joint	
Aurum only	209385006	320086017	265441000006118		S4H3500	open #-subluxation, metatarsophalangeal joint, multiple	
Aurum only	209386007	320087014	255711000006118		S4H3600	open fracture-subluxation, ipj, multiple toes	
Aurum only	209402006	320103012	559921000006116		S4J2300	closed #-subluxation sterno-clavicular joint, posterior	
Aurum only	209406009	320107013	255611000006114		S4J3200	open fracture-subluxation sterno-clavicular joint, anterior	
Aurum only	209407000	320108015	255621000006118		S4J3300	open fracture-subluxation sterno-clavicular joint, posterior	
Aurum only	21351003	895901000006119	895901000006119		S36..99	#phalanges of foot - toe	

Aurum only	21698002	36405012	36405012			open fracture of phalanx of finger	
Aurum only	22000002	36906016	36906016		S121300	open fracture of three ribs	
Aurum only	239548003	358967010	276508016		7J42H00	revision to other external stabilisation of spinal fracture	History
Aurum only	24063002	40395011	264221000006113		S012400	open #skull bse no intracranial injury, >24hr loc+recovery	
Aurum only	24063002	40395011	264251000006116		S012100	open #skull bse no intracranial injury, no loss of consc	
Aurum only	24063002	40395011	264241000006118		S012600	open #skull bse no intracranial injury, loc unspec duration	
Aurum only	24063002	40395011	264191000006118		S012000	open #skull bse no intracranial inj, unspec state of consc	
Aurum only	24063002	40395011	264231000006111		S012300	open #skull bse no intracranial injury, 1-24hr loss of consc	
Aurum only	24063002	40395011	264181000006116		S012500	open #skull bse no intracranial inj, >24hr loc not restored	
Aurum only	24063002	40395011	264201000006115		S012z00	open #skull bse no intracranial injury + concussion unspec	
Aurum only	24063002	895171000006110	895171000006110		S01..99	#base of skull	
Aurum only	24424003	40965012	40965012			closed fracture of phalanx of finger	
Aurum only	25060006	875711000006112	875711000006112		7K1LD99	closed reduction- # nasal bone	
Aurum only	262525000	390470012	325341011		Syu2700	[x]fracture of other parts of bony thorax	
Aurum only	262525000	390470012	455024016		S12y100	open fracture of other parts of bony thorax	
Aurum only	262525000	390470012	455021012		S12X100	open fracture of bony thorax part unspecified	
Aurum only	263102004	391219013	256351000006110		S4C3y00	open fracture-subluxation other carpal	
Aurum only	263105002	391222010	256271000006114		S4D3200	open fracture-subluxation ipj, unspecified	
Aurum only	263151001	895211000006112	895211000006112		S024099	#maxilla	
Aurum only	263188003	391337012	391337012		S101.11	open fracture of atlas without spinal cord lesion	
Aurum only	263189006	391340012	391340012		S101.12	open fracture of axis without spinal cord lesion	
Aurum only	263231005	2536030018	2536030018		S301300	open fracture proximal femur, basicervical	

Aurum only	263232003	989551000006116	989551000006116		S315.99	#lower end of femur	
Aurum only	263244000	989601000006119	989601000006119		S344.99	#ankle - bimalleolar - potts #	
Aurum only	265132005	393775016	208751000006118		7K1D01C	prim open reduct # neck femur & op fix - thornton nail plate	
Aurum only	265138009	393781012	208841000006110		7K1E611	prim open reduction # elbow & fixatn c zuezler fixatn plate	
Aurum only	265721002	394587017	602751000006117		7J43212	crutchfield skull traction for fracture of spine	
Aurum only	265721002	394587017	159461000006110		7J43D00	rvsn open reduc spinal fracture+other internal fix	History
Aurum only	268029009	892401000006119	892401000006119		N331.91	path.fracture - nos	
Aurum only	268029009	892481000006111	892481000006111		N331.99	path.fracture - multiple	
Aurum only	269061001	990941000006118	990941000006118		S10..99	#spine nos - no cord lesion	
Aurum only	269062008	895271000006115	895271000006115		S100.99	#cervical spine-no cord lesion	
Aurum only	269070003	402841017	318434019		S101z00	open fracture of cervical spine not otherwise specified	
Aurum only	269073001	402844013	402844013		S101300	open fracture of third cervical vertebra	
Aurum only	269074007	402845014	402845014		S101400	open fracture of fourth cervical vertebra	
Aurum only	269078005	402850015	318600014		S11y.00	open fracture of spine with spinal cord lesion unspecified	
Aurum only	269080004	895501000006111	895501000006111		S224.99	#humerus - lower end	
Aurum only	269083002	895551000006110	895551000006110		S234198	#radius/ulna-lower end-colles	
Aurum only	269083002	895561000006112	895561000006112		S234199	colles fracture	
Aurum only	269100000	991051000006110	991051000006110		S35..99	#tarsal/metatarsal bone nos	
Aurum only	274156000	409924017	44371000006115		S111500	opn spinal # with incomplete cervical cord lesion, c1-4 nos	
Aurum only	274156000	409924017	44381000006117		S111B00	opn spinal # with incomplete cervical cord lesion, c5-7 nos	
Aurum only	274156000	409924017	43211000006117		S111600	opn spinal fracture with unspec cervical cord lesion, c5-7	
Aurum only	274156000	409924017	43201000006115		S111000	opn spinal fracture with unspec cervical cord lesion, c1-4	

Aurum only	274157009	409925016	43231000006111		S113600	opn spinal fracture with unspec thoracic cord lesion, t7-12	
Aurum only	274157009	409925016	44391000006119		S113500	opn spinal # with incomplete thoracic cord lesion, t1-6 nos	
Aurum only	274157009	409925016	44401000006117		S113B00	opn spinal # with incomplete thoracic cord lesion, t7-12 nos	
Aurum only	274158004	409926015	51781000006118		S115z00	open spinal fracture with incomplete lumbar cord lesion nos	
Aurum only	274158004	409926015	318564012		S115000	open spinal fracture with unspecified lumbar cord lesion	
Aurum only	274160002	989531000006111	989531000006111		S262.99	#phalanx of thumb	
Aurum only	27477003	895181000006113	895181000006113		S02..99	#facial bones	
Aurum only	27477003	990931000006111	990931000006111		S02..98	#facial bones nos	
Aurum only	27644009	46274013	560041000006114		S010500	closed #skull bse no intracranial inj,>24hr loc not restored	
Aurum only	27644009	46274013	560081000006115		S010300	closed #skull bse no intracranial injury, 1-24hr loss consc	
Aurum only	28078000	895541000006113	895541000006113		S232.99	#radius/ulna -shaft	
Aurum only	281973008	420142011	420142011			weber type c fracture	
Aurum only	281974002	420143018	420143018			weber type b fracture	
Aurum only	281975001	420144012	420144012			weber type a fracture	
Aurum only	287064006	892471000006113	892471000006113		N331.98	path.fracture - shoulder	
Aurum only	287065007	892461000006118	892461000006118		N331.97	path.fracture - upper arm	
Aurum only	287066008	892451000006115	892451000006115		N331.96	path.fracture - fore arm	
Aurum only	287067004	892441000006117	892441000006117		N331.95	path.fracture - hand	
Aurum only	287068009	892431000006110	892431000006110		N331.94	path.fracture - pelvis/thigh	
Aurum only	287069001	892421000006112	892421000006112		N331.93	path.fracture - lower leg	
Aurum only	287070000	892411000006116	892411000006116		N331.92	path.fracture - ankle/foot	
Aurum only	29014003	48573010	318934012		S241y00	open fracture of other carpal bone	
Aurum only	301052006	442282011	167041000006119		7K6Hd00	revision to open reduction # dislocation+oth externl immobil	History

Aurum only	301053001	442283018	205011000006114		7K6FL00	primary open reduction # dislocation+other external immobil	
Aurum only	311409007	454978014	454978014		S124100	open flail chest	
Aurum only	311428000	454999012	1702421000006111		7K1Y200	remanip frac of long bone and flexible internal fixation hfq	History
Aurum only	311488002	455069010	210061000006115		7J42Q00	primary cls reduct spinal fracture+oth external stabilisatn	
Aurum only	311489005	455070011	171431000006117		7J42X00	revision cls reduc spinal fracture+other external stabilistn	History
Aurum only	311489005	455070011	276522016		7J42R00	revision to closed reduction spinal fracture alone	History
Aurum only	311490001	455071010	205441000006119		7J42e00	primary opn reduc spinal fracture+other external stabilisatn	
Aurum only	311490001	455071010	276534012		7J42Y00	primary open reduction spinal fracture alone	
Aurum only	311491002	455072015	276546011		7J42f00	revision to open reduction spinal fracture alone	History
Aurum only	311491002	455072015	159451000006113		7J42m00	rvsn open reduc spinal fracture+other external stabilisation	History
Aurum only	311820001	455450014	265381000006115		S294100	op fractures involving multiple regions of both upper limbs	
Aurum only	311848001	455486010	455486010		SR10100	open fractures involving head with neck	
Aurum only	311850009	455488011	455488011		SR12100	open fractures involving multiple regions of one upper limb	
Aurum only	311852001	455491011	559801000006115		SR14000	closd fractures involving multiple regions of both low limbs	
Aurum only	311853006	455492016	255841000006114		SR14100	open fractures involving multiple regions both lower limbs	
Aurum only	311859005	455498017	259491000006116		SR15100	open fracture involv multiple regions upper with lower lmb	
Aurum only	311864009	455503019	259481000006119		SR16100	open fracture inv thorax wth low back and pelvis and limbs	
Aurum only	316893008	461557016	318551000006118		Zw02G00	[q] open fracture grade 3a	
Aurum only	316894002	461558014	318561000006116		Zw02H00	[q] open fracture grade 3b	

Aurum only	316895001	461560011	461560011		Zw02J00	[q] open fracture grade 3c	
Aurum only	33118001	895421000006117	895421000006117		S130.99	#acetabulum of pelvis	
Aurum only	3381004	6700013	6700013		S353111	open fracture of astragalus	
Aurum only	342070009	470290012	470290012			closed fracture of foot	
Aurum only	34268009	989591000006110	989591000006110		S342.99	#ankle - lateral malleolus	
Aurum only	367527001	492641015	492641015			open fracture of foot	
Aurum only	371161001	1209863015	52921000006113		S033400	open #skull nos + intracranial inj, >24hrs loc + recovery	
Aurum only	371161001	1209863015	264281000006112		S033500	open #skull nos + intracranial inj, >24hrs loc not restored	
Aurum only	371161001	1209863015	264301000006111		S033600	open #skull nos + intracranial inj, loc unspec duration	
Aurum only	371161001	1209863015	264291000006110		S033300	open #skull nos + intracranial inj, 1-24hrs loss of consc	
Aurum only	371161001	1209863015	264271000006114		S033200	open #skull nos + intracranial inj, <1hr loss of consc	
Aurum only	371161001	1209863015	264361000006112		S032500	open #skull nos no intracranial inj, >24hrs loc not restored	
Aurum only	371161001	1209863015	264381000006119		S032600	open #skull nos no intracranial inj, loc unspec duration	
Aurum only	371161001	1209863015	264371000006117		S032300	open #skull nos no intracranial inj, 1-24hrs loss of consc	
Aurum only	371161001	1209863015	264321000006118		S033000	open #skull nos + intracranial inj, unspec state of consc	
Aurum only	371161001	1209863015	264311000006114		S033100	open #skull nos + intracranial inj, no loss of consc	
Aurum only	371161001	1209863015	264351000006110		S032400	open #skull nos no intracranial inj, >24hrs loc + recovery	
Aurum only	371161001	1209863015	264391000006116		S032100	open #skull nos no intracranial inj, no loss of consc	
Aurum only	371161001	1209863015	264401000006119		S032000	open #skull nos no intracranial inj, unspec state of consc	
Aurum only	371161001	1209863015	264341000006113		S032200	open #skull nos no intracranial inj, <1hr loss of consc	
Aurum only	371161001	1209863015	264261000006119		S033z00	open #skull nos + intracranial inj + concussion unspec	

Aurum only	371162008	1209864014	560141000006113		S031400	closed #skull nos + intracranial inj, >24hrs loc + recovery	
Aurum only	371162008	1209864014	560171000006117		S031100	closed #skull nos + intracranial inj, no loss of consc	
Aurum only	371162008	1209864014	560261000006117		S030500	closed #skull nos no intracranial inj,>24hr loc not restored	
Aurum only	371162008	1209864014	560181000006119		S031000	closed #skull nos + intracranial inj, unspec state of consc	
Aurum only	371162008	1209864014	1209864014			closed fracture of skull	
Aurum only	371162008	1209864014	560131000006115		S031500	closed #skull nos + intracranial inj, >24hr loc not restored	
Aurum only	371162008	1209864014	560231000006114		S030600	closed #skull nos no intracranial inj, loc unspec duration	
Aurum only	371162008	1209864014	560211000006115		S030400	closed #skull nos no intracranial inj, >24hrs loc + recovery	
Aurum only	371162008	1209864014	560111000006114		S031z00	closed #skull nos + intracranial inj + concussion unspec	
Aurum only	371566003	1210202016	209761000006113		7K1KH00	primary closed reduction of # + oth external immobilisation	
Aurum only	379161000000101	746911000000110	1702431000006114		7K1Y300	remanipulation fracture small bone and fixation using screw	History
Aurum only	405817008	895651000006111	895651000006111		S26..99	#phalanx of finger/thumb	
Aurum only	405817008	991691000006114	991691000006114		S26..98	#phalanx of finger/thumb nos	
Aurum only	413876003	2533503012	2533503012			closed fracture of lower leg	
Aurum only	414293001	895751000006118	895751000006118		S33..95	#tibia/fibula - shaft	
Aurum only	414293001	895761000006116	895761000006116		S33..96	#tibia/fibula - upper end	
Aurum only	414293001	895771000006111	895771000006111		S33..97	#fibula alone	
Aurum only	414293001	895781000006114	895781000006114		S33..98	#tibia alone	
Aurum only	414293001	895791000006112	895791000006112		S33..99	#tibia/fibula	
Aurum only	414293001	991701000006114	991701000006114		S33..94	#tibia/fibula nos	
Aurum only	414942001	2533507013	2533507013			open fracture of lower leg	
Aurum only	42636007	895481000006118	895481000006118		S220.99	#humerus - upper end	
Aurum only	428019004	2694503015	2694503015			open fracture of bone of knee joint	

Aurum only	428151000	2693040014	2693040014			closed fracture of bone of knee joint	
Aurum only	42818005	895601000006112	895601000006112		S240199	#scaphoid	
Aurum only	430984009	2770946011	2770946011			closed fracture of facial bone	
Aurum only	44039000	73439011	73439011		S121500	open fracture of five ribs	
Aurum only	441546003	2816901016	1764531000006116			periprosthetic fracture	
Aurum only	46866001	895911000006116	895911000006116		S3y..99	multiple fractures	
Aurum only	4788002	895221000006116	895221000006116		S024199	#zygoma	
Aurum only	48466003	895151000006117	895151000006117		S00..98	#parietal bone	
Aurum only	48466003	895161000006115	895161000006115		S00..99	#frontal bone	
Aurum only	49128003	895261000006110	895261000006110		S04..99	#skull - multiple	
Aurum only	49346003	895231000006118	895231000006118		S026.99	#orbital floor (blow-out)	
Aurum only	50531009	84170010	695611000006115		S3y1.00	multiple open #both legs, leg + arm, leg + rib + sternum	
Aurum only	51037009	895741000006115	895741000006115		S32..99	#patella	
Aurum only	54441004	989541000006118	989541000006118		S314.99	#shaft of femur	
Aurum only	544741000000101	895251000006113	895251000006113		S03z.99	#skull nos	
Aurum only	545441000000107	932521000006112	932521000006112		7J1y.99	open reduction - alveolar #	
Aurum only	5468008	10127011	263921000006119		S043200	open #skull/face, mult + intracranial inj, <1hr loc	
Aurum only	5468008	10127011	263911000006110		S043600	open #skull/face, mult + intracran inj, loc unspec duration	
Aurum only	5468008	10127011	264001000006114		S043500	open #skull/face,mult + intracran inj, >24hr loc no restored	
Aurum only	5468008	10127011	560471000006111		S041400	closed #skull/face, mult+intracran inj, >24hr loc+recovery	
Aurum only	5468008	10127011	263961000006113		S042200	open #skull/face, mult, no intracranial inj, <1hr loc	
Aurum only	5468008	10127011	560431000006113		S041200	closed #skull/face, mult + intracranial inj, <1hr loc	
Aurum only	5468008	10127011	263951000006111		S042400	open #skull/face, mult, no intracran inj, >24hr loc+recovery	
Aurum only	5468008	10127011	263981000006115		S042100	open #skull/face, mult, no intracranial inj, no loss consc	

Aurum only	5468008	10127011	263991000006117		S042000	open #skull/face, mult, no intracranial inj, unspec consc	
Aurum only	5468008	10127011	560521000006114		S040400	closed #skull/face, mult,no intracran inj,>24hr loc+recovery	
Aurum only	5468008	10127011	263971000006118		S042300	open #skull/face, mult, no intracranial inj, 1-24hrs loc	
Aurum only	5468008	10127011	52911000006117		S043300	open #skull/face, mult + intracranial inj, 1-24hrs loc	
Aurum only	5468008	10127011	264021000006116		S042600	open #skull/face,mult,no intracran inj, loc unspec duration	
Aurum only	5468008	10127011	263891000006113		S043z00	open #skull/face, mult + intracran inj + concussion, unspec	
Aurum only	5468008	10127011	560491000006112		S040300	closed #skull/face, mult, no intracranial inj, 1-24hrs loc	
Aurum only	5468008	10127011	560451000006118		S041100	closed #skull/face, mult + intracranial inj, no loss consc	
Aurum only	5468008	10127011	264011000006112		S042z00	open #skull/face,mult,no intracran inj, concussion unspec	
Aurum only	5468008	10127011	264031000006118		S042500	open #skull/face,mult,no intracran inj,>24hr loc no restored	
Aurum only	5468008	10127011	560571000006110		S040500	closed #skull/face,mult,no intracran inj,>24hr loc-restored	
Aurum only	5468008	10127011	560581000006113		S040600	closed #skull/face,mult,no intracran inj,loc unspec duration	
Aurum only	5468008	10127011	263931000006116		S043100	open #skull/face, mult + intracranial inj, no loss consc	
Aurum only	5468008	10127011	560531000006112		S041500	closed #skull/face, multi+intracran inj, >24hr loc-restored	
Aurum only	550131000000103	874741000006110	874741000006110		7J03199	#nasal bone - open reduction	
Aurum only	550151000000105	874771000006119	874771000006119		7J03z99	facial # - reduction nos	
Aurum only	552301000000105	875611000006116	875611000006116		7K1Dy99	open # reduction+intern.fixat.	
Aurum only	559241000000108	877711000006118	877711000006118		7L1G099	splinting of fracture	
Aurum only	57168000	875341000006110	875341000006110		7K1..99	surgical # reduction	
Aurum only	58150001	895451000006114	895451000006114		S20..99	#clavicle	

Aurum only	58154005	96634011	96634011		S121400	open fracture of four ribs	
Aurum only	5913000	895691000006117	895691000006117		S30..99	#neck of femur	
Aurum only	592821000000104	895731000006113	895731000006113		S31z.99	#femur nos	
Aurum only	592951000000104	895801000006113	895801000006113		S33z.99	#tibia/fibula nos	
Aurum only	592981000000105	895871000006119	895871000006119		S34z.99	#ankle - nos	
Aurum only	593041000000109	895891000006118	895891000006118		S35z.99	#tarsal/metatarsal bone nos	
Aurum only	605161000000102	895701000006117	895701000006117		S31..97	#lower end of femur	
Aurum only	605161000000102	895711000006119	895711000006119		S31..98	#shaft of femur	
Aurum only	605161000000102	895721000006110	895721000006110		S31..99	#femur	
Aurum only	605161000000102	991061000006112	991061000006112		S31..96	#femur nos	
Aurum only	606041000000107	895511000006114	895511000006114		S22z.99	#humerus nos	
Aurum only	60667009	895371000006112	895371000006112		S120.99	#rib	
Aurum only	614461000000105	895921000006112	895921000006112		S3z..99	fractures	
Aurum only	623711000000109	895491000006115	895491000006115		S222.99	#humerus - shaft	
Aurum only	628991000000108	875761000006110	875761000006110		7K1Lz99	other closed # reduction nos	
Aurum only	634841000000102	874911000006114	874911000006114		7J12199	open reduction -mandibular #	
Aurum only	634851000000104	874921000006118	874921000006118		7J12299	closed reduction-mandibular #	
Aurum only	634871000000108	874941000006113	874941000006113		7J13199	open reduction - maxillary #	
Aurum only	634881000000105	874951000006110	874951000006110		7J13299	closed reduction - maxillary #	
Aurum only	63563000	875701000006114	875701000006114		7K1LC99	lower limb #-closed reduct.nos	
Aurum only	64665009	895881000006116	895881000006116		S350.99	#calcaneus - heel	
Aurum only	648581000000104	895311000006115	895311000006115		S10z.99	#spine nos - no cord lesion	
Aurum only	649391000000102	989621000006112	989621000006112		SR1z.99	multiple fractures	
Aurum only	655981000000101	875621000006112	875621000006112		7K1G.98	operation for multiple #	
Aurum only	655981000000101	875631000006110	875631000006110		7K1G.99	open # reduction	
Aurum only	656001000000100	875641000006117	875641000006117		7K1Gz99	surgical # reduction nos	
Aurum only	656001000000100	932541000006117	932541000006117		7K1Gz98	open # reduction+intern.fixat.	
Aurum only	66112004	895381000006110	895381000006110		S122.99	#sternum	
Aurum only	66308002	895471000006116	895471000006116		S22..99	#humerus	
Aurum only	66308002	990971000006114	990971000006114		S22..98	#humerus nos	
Aurum only	66691008	110787019	318722014		S141.00	open fracture of ill-defined bone of trunk	
Aurum only	6698000	989611000006116	989611000006116		S346.99	#ankle - trimalleolar	

Aurum only	674461000000100	895571000006117	895571000006117		S234599	fracture of distal end of ulna	
Aurum only	674611000000104	895581000006119	895581000006119		S23z.99	#radius/ulna nos	
Aurum only	687331000000100	895361000006117	895361000006117		S11z.99	#spine nos + cord lesion	
Aurum only	69446009	875811000006115	875811000006115		7K1N.95	traction for closed #	
Aurum only	695661000000108	989521000006113	989521000006113		S263.99	#phalanx of finger	
Aurum only	695671000000101	895681000006115	895681000006115		S26z.99	#phalanx of finger/thumb nos	
Aurum only	697111000000107	895611000006110	895611000006110		S24z.99	#carpal bones nos	
Aurum only	6990005	989561000006119	989561000006119		S337.99	#tibia alone	
Aurum only	700861000000109	895641000006114	895641000006114		S250z99	#metacarpal bone nos	
Aurum only	704213001	3011218018	3011218018			closed fracture of phalanx of thumb	
Aurum only	704236005	3012276014	3012276014			open fracture of phalanx of thumb	
Aurum only	71642004	895141000006119	895141000006119		S0...99	#skull	
Aurum only	71642004	990921000006113	990921000006113		S0...98	#skull nos	
Aurum only	75857000	895521000006118	895521000006118		S23..99	#radius/ulna	
Aurum only	75857000	991021000006118	991021000006118		S23..98	#radius/ulna nos	
Aurum only	77493009	895411000006113	895411000006113		S13..99	#pelvis	
Aurum only	77493009	895441000006112	895441000006112		S13y.99	#pelvis nos	
Aurum only	77493009	990951000006116	990951000006116		S13..98	#pelvis nos	
Aurum only	77546004	128724013	128724013		S121600	open fracture of six ribs	
Aurum only	77598002	128798015	318670013		S131600	open fracture acetabulum, double column unspecified	
Aurum only	78011002	895391000006113	895391000006113		S124.99	#flail chest	
Aurum only	81639003	895191000006111	895191000006111		S020.99	#nasal bones	
Aurum only	82065001	895591000006116	895591000006116		S24..99	#carpal bones	
Aurum only	82065001	991031000006115	991031000006115		S24..98	#carpal bones nos	
Aurum only	84385008	875741000006111	875741000006111		7K1LN99	upper limb #-closed reduct.nos	
Aurum only	851421000006105	851421000006114	851421000006114			torus fracture of radius	
Aurum only	857661000006106	857661000006110	857661000006110			wedge compression # lumbar spine	
Aurum only	857671000006104	857671000006115	857671000006115			wedge compression # of dorsal spine	
Aurum only	90235006	990961000006119	990961000006119		S222199	#humerus - shaft	
Aurum only	907011000006101	907011000006117	907011000006117			[rfc] jaw fracture	
Aurum only	909211000006102	909211000006118	909211000006118			[rfc] fracture/ trauma	

Aurum only	91296001	151267013	151267013			open fracture of forearm	
Aurum only	91419009	151450016	151450016			closed fracture of forearm	
Aurum only	9682006	895461000006111	895461000006111		S21..99	#scapula	
Aurum only	981341000006105	981341000006114	981341000006114			fractures	
GOLD only				30377	Z6G1900	fracture - traction	
Aurum and GOLD	10859002	18859019	649231000006118	30707	S01..12	ethmoid sinus fracture	
Aurum and GOLD	111603000	178853015	561651000006113	63679	S011.00	closed fracture base of skull with intracranial injury	
Aurum and GOLD	111603000	178853015	559961000006110	99019	S011000	closed #skull bse + intracranial inj, unspec state of consc	
Aurum and GOLD	111603000	178853015	559971000006115	111559	S011z00	closed #skull bse + intracranial injury + concussion unspec	
Aurum and GOLD	111603000	178853015	560011000006110	45956	S011600	closed #skull bse + intracranial injury, loc unspec duration	
Aurum and GOLD	111607004	178855010	264151000006112	99282	S013600	open #skull bse + intracranial injury, loc unspec duration	
Aurum and GOLD	111607004	178855010	264451000006115	102718	S013z00	open #skull bse + intracranial injury + concussion unspec	
Aurum and GOLD	111607004	178855010	264171000006119	113378	S013000	open #skull bse + intracranial injury, unspec state of consc	
Aurum and GOLD	111607004	178855010	259601000006113	97064	S013.00	open fracture base of skull with intracranial injury	
Aurum and GOLD	111609001	178856011	318248017	59233	S02y.00	open fracture other facial bone	
Aurum and GOLD	111622009	178861013	531621000006112	52699	S100311	c3 vertebra closed fracture without spinal cord lesion	
Aurum and GOLD	111636004	178862018	178862018	94108	S126300	open fracture of trachea	
Aurum and GOLD	111637008	178863011	318733012	94460	S201z00	open fracture of clavicle nos	
Aurum and GOLD	111637008	178863011	178863011	61812	S201.00	open fracture of clavicle	
Aurum and GOLD	111637008	178863011	318732019	70864	S201000	open fracture of clavicle, unspecified part	
Aurum and GOLD	111638003	1219672018	1219672018	100040	S201100	open fracture clavicle, medial end	
Aurum and GOLD	111639006	1219673011	1219673011	16389	S210100	closed fracture scapula, acromion	
Aurum and GOLD	111640008	178867012	402863014	17952	S23x100	closed fracture of radius (alone), unspecified	
Aurum and GOLD	111641007	1219674017	1219674017	34737	S231700	open fracture radial neck	

Aurum and GOLD	111643005	178870011	257771000006115	28273	S321.00	open fracture of the patella	
Aurum and GOLD	111644004	364017010	564011000006117	44830	S330.00	closed fracture of tibia and fibula, proximal	
Aurum and GOLD	111644004	364017010	364017010	54280	S330200	closed fracture of tibia and fibula, proximal	
Aurum and GOLD	111645003	2969178012	257801000006118	33706	S331000	open fracture of the proximal tibia	
Aurum and GOLD	111646002	1219675016	1219675016	51938	S333100	open fracture shaft of fibula	
Aurum and GOLD	112777008	183865012	183865012	18962	7K1L500	closed reduction of fracture of femur	
Aurum and GOLD	11782000	20339011	531601000006119	33967	S100211	c2 vertebra closed fracture without spinal cord lesion	
Aurum and GOLD	11964008	20626019	210921000006113	35004	7K1J500	primary int fxn(no red) prox fem #+screw/nail device alone	
Aurum and GOLD	12204004	1220597016	1220597016	11004	S120900	closed fracture multiple ribs	
Aurum and GOLD	125605004	194093016	402879010	2470	S3z..00	fracture of unspecified bones	
Aurum and GOLD	125605004	194093016	319353015	953	S3z1.00	open fracture of bones, unspecified	
Aurum and GOLD	125605004	194093016	325508014	109469	SyuBB00	[x]fracture of unspecified body region	
Aurum and GOLD	125605004	194093016	319355010	29162	S3zz.00	fracture of bones nos	
Aurum and GOLD	125605004	194093016	329379013	16769	TC7..00	fracture, cause unspecified	
Aurum and GOLD	125605004	473534016	403161016	358	S3z..11	fracture nos	
Aurum and GOLD	125606003	1220722015	1220722015	3288	S10A.00	fracture of neck	
Aurum and GOLD	125606003	194094010	325323015	94638	Syu1500	[x]fracture of other specified cervical vertebra	
Aurum and GOLD	125606003	194094010	325324014	99936	Syu1600	[x]fracture of other parts of neck	
Aurum and GOLD	125607007	1220723013	1220723013	5381	S15..00	fracture of thoracic vertebra	
Aurum and GOLD	125608002	1220724019	1220724019	10990	S10B000	fracture of lumbar vertebra	
Aurum and GOLD	125871005	194543011	194543011	835	S10B200	fracture of coccyx	
Aurum and GOLD	125872003	194542018	194542018	3675	S10B100	fracture of sacrum	
Aurum and GOLD	1261007	1220743019	1220743019	10696	S127000	multiple fractures of ribs	
Aurum and GOLD	1261007	3226012	695161000006110	17107	S29..12	multiple rib fractures	
Aurum and GOLD	12676007	21770014	391347010	137	S23x111	fracture of radius nos	
Aurum and GOLD	12676007	21770014	318912010	34367	S23y100	open fracture of radius (alone), unspecified	
Aurum and GOLD	127278005	566013	325381016	52457	Syu4300	[x]fracture of other parts of shoulder and upper arm	
Aurum and GOLD	127286005	574014	574014	2101	S226.00	fracture of upper end of humerus	

Aurum and GOLD	12746007	21881015	402871013	68668	S301y00	open fracture proximal femur, other transcervical	
Aurum and GOLD	12746007	21881015	257561000006119	112712	S301z00	open fracture proximal femur, transcervical, nos	
Aurum and GOLD	12746007	3043023014	319100018	73981	S301.00	open fracture proximal femur, transcervical	
Aurum and GOLD	134291007	216055019	455736015	50270	SR1z.00	multiple fractures, unspecified	
Aurum and GOLD	134291007	216055019	455508011	73336	SR1z000	[x]closed multiple fractures unspecified	
Aurum and GOLD	134291007	216055019	455509015	96460	SR1z100	[x]open multiple fractures unspecified	
Aurum and GOLD	135895003	216665012	216665012	53007	Zw01.00	[q] fractures involving the epiphyseal plate	
Aurum and GOLD	1370007	1221007014	1221007014	29111	S251.00	open fracture of metacarpal bone(s)	
Aurum and GOLD	1370007	3391014	318978015	47847	S251z00	open fracture of metacarpal bone(s) nos	
Aurum and GOLD	1370007	3391014	318977013	94393	S251x00	open fractures of multiple sites of unspecified metacarpus	
Aurum and GOLD	1370007	3391014	318964016	62853	S251000	open fracture of metacarpal bone(s), site unspecified	
Aurum and GOLD	150617003	235046017	499625015	55077	7K1LC00	closed reduction of fracture of lower limb	
Aurum and GOLD	150617003	235046017	499624016	31902	7K1LC11	closed reduction # leg	
Aurum and GOLD	15385006	1221187014	1221187014	6286	S340.00	closed fracture ankle, medial malleolus	
Aurum and GOLD	15741007	1221230012	1221230012	55531	S02x200	fracture of palate, closed	
Aurum and GOLD	16114001	27272011	27272011	325	S34..00	fracture of ankle	
Aurum and GOLD	16114001	27273018	319273011	9212	S34z.00	fracture of ankle, nos	
Aurum and GOLD	16250001000004108	3047594015	325382011	53866	Syu4400	[x]fracture of shoulder and upper arm, unspecified	
Aurum and GOLD	1658003	1221377017	1221377017	29899	S200300	closed fracture clavicle, lateral end	
Aurum and GOLD	1705000	3954014	565211000006111	51861	S300311	closed fracture, base of neck of femur	
Aurum and GOLD	1705000	3954014	564391000006113	52194	S300300	closed fracture proximal femur, basicervical	
Aurum and GOLD	172182006	266614018	266614018	20445	7206400	open reduction of fracture of orbit and internal fixation	
Aurum and GOLD	172185008	266618015	266618015	39071	7206700	packing of maxilla to correct blow-out fracture of orbit	
Aurum and GOLD	17222009	29115016	318862018	28293	S234E00	closed fracture distal radius, intra-articular, other type	
Aurum and GOLD	17222009	29115016	632631000006110	23987	S234211	dupuytren's fracture, radius - closed	

Aurum and GOLD	17222009	29115016	402860012	1742	S234200	closed fracture of the distal radius, unspecified	
Aurum and GOLD	1739001	1221530011	268071000006113	9736	S01..15	occiput bone fracture	
Aurum and GOLD	17633000	29800015	29800015	55424	S120500	closed fracture of five ribs	
Aurum and GOLD	178395000	276102014	276102014	25173	7J03000	reduction of fracture of nasoethmoid complex of bones	
Aurum and GOLD	178397008	276104010	183781000006117	4465	7J03200	reduction of fracture of zygomatic bones	
Aurum and GOLD	178398003	276106012	276106012	43730	7J03300	reduction of closed fracture of orbit bone	
Aurum and GOLD	178436001	276158018	276158018	60254	7J12000	reduction of fracture of alveolus of mandible	
Aurum and GOLD	178441009	276163019	276163019	22780	7J13.00	reduction of fracture of maxilla	
Aurum and GOLD	178441009	276163019	276178014	69319	7J13z00	reduction of fracture of maxilla nos	
Aurum and GOLD	178441009	276163019	276177016	88784	7J13y00	other specified reduction of fracture of maxilla	
Aurum and GOLD	178442002	276164013	276164013	30288	7J13000	reduction of fracture of alveolus of maxilla	
Aurum and GOLD	178445000	276167018	183641000006117	25312	7J13300	reduction of blowout fracture of orbital floor	
Aurum and GOLD	178446004	276168011	276168011	4350	7J13400	reduction of le fort 1 fracture of maxilla	
Aurum and GOLD	178447008	276173017	276173017	51147	7J13500	reduction of le fort 2 fracture of maxilla	
Aurum and GOLD	178448003	276174011	276174011	60412	7J13600	reduction of le fort 3 fracture of maxilla	
Aurum and GOLD	178496003	276240016	276240016	17138	7J17700	traction for fracture of jaw	
Aurum and GOLD	178674000	276467013	276474015	45410	7J41z00	decompression of fracture of spine nos	
Aurum and GOLD	178674000	276467013	276467013	31933	7J41.00	decompression of fracture of spine	
Aurum and GOLD	178674000	276467013	276471011	65606	7J41y00	other specified decompression of fracture of spine	
Aurum and GOLD	178675004	276468015	276468015	106504	7J41000	complex decompression of fracture of spine	
Aurum and GOLD	178676003	276469011	276469011	68811	7J41100	anterior decompression of fracture of spine	
Aurum and GOLD	178677007	276470012	375761000000118	91658	7J41400	posterior decompression of fracture of spine nec	
Aurum and GOLD	178677007	276470012	276470012	98016	7J41200	posterior decompression of fracture of spine	
Aurum and GOLD	178682000	276480011	43041000006111	49564	7J42000	open reduction of fracture of spine & excis facet of spine	
Aurum and GOLD	178684004	276482015	698931000006114	49529	7J42200	manipulative reduction of fracture of spine	

Aurum and GOLD	178685003	276483013	276483013	63452	7J42300	spinal extension traction for fracture of spine	
Aurum and GOLD	178686002	276484019	276484019	43600	7J42400	halo skull traction for fracture of spine	
Aurum and GOLD	178688001	276487014	276487014	51521	7J42600	primary bedrest stabilisation of spinal fracture	
Aurum and GOLD	178689009	276488016	276488016	46171	7J42700	primary collar stabilisation of spinal fracture	
Aurum and GOLD	178691001	276492011	276492011	71006	7J42900	primary cast stabilisation of spinal fracture	
Aurum and GOLD	178694009	276498010	276498010	100161	7J42C00	revision to bedrest stabilisation of spinal fracture	History
Aurum and GOLD	178695005	276501010	276501010	58589	7J42D00	revision to collar stabilisation of spinal fracture	History
Aurum and GOLD	178698007	276506017	166841000006113	94374	7J42G00	revision to external fixation stabilisation spinal fracture	History
Aurum and GOLD	178702006	276513017	210111000006119	104803	7J42L00	primary cls reduction spinal fracture+bedrest stabilisation	
Aurum and GOLD	178703001	276516013	210051000006117	61389	7J42M00	primary cls reduc spinal fracture+skull traction stabilisatn	
Aurum and GOLD	178733008	276565015	204961000006112	28966	7J43000	primary open reduc spinal fracture+internal fix+plate	
Aurum and GOLD	178734002	276566019	276566019	34850	7J43100	fixation of fracture of spine using harrington rod	
Aurum and GOLD	178736000	276571014	204981000006119	64388	7J43300	primary open reduc spinal fracture+internal fix+wire	
Aurum and GOLD	178737009	276572019	204971000006117	61491	7J43400	primary open reduc spinal fracture+internal fix+rod system	
Aurum and GOLD	178738004	276573012	204941000006113	108040	7J43500	primary open reduc spinal #+intern fix+segmental wire system	
Aurum and GOLD	178739007	276574018	204951000006110	64709	7J43600	primary open reduc spinal #+internal fix+internal fixator	
Aurum and GOLD	178742001	276577013	159421000006116	67910	7J43900	rvsn open reduc spinal fracture+internal fix+plate	History
Aurum and GOLD	178743006	276578015	159431000006118	98056	7J43A00	rvsn open reduc spinal fracture+internal fix+rod system	History
Aurum and GOLD	178745004	276580014	159411000006112	63218	7J43C00	rvsn open reduc spinal fracture+internal fix+internl fixator	History

Aurum and GOLD	178926002	276851010	276863017	32178	7K14y00	other specified open surgical fracture of bone	
Aurum and GOLD	178926002	276851010	276864011	41563	7K14z00	open surgical fracture of bone nos	
Aurum and GOLD	178926002	276852015	276852015	6734	7K14.00	open surgical fracture of bone	
Aurum and GOLD	179010008	276977015	208831000006117	4115	7K1D100	prim open reduct fract long bone & fixation rigid nail nec	
Aurum and GOLD	179010008	276977015	205041000006113	7672	7K1D.00	primary open reduction fracture bone & intramedull fixation	
Aurum and GOLD	179010008	276977015	208871000006119	36698	7K1Dz00	prim open reduction fracture bone & intramedull fixation nos	
Aurum and GOLD	179010008	276977015	208881000006116	34277	7K1Dy00	prim open reduction fracture bone & intramedullary fixatn os	
Aurum and GOLD	179010008	276978013	207721000006114	28667	7K1DD00	prmy open reduction of #+intramedullary nail fixation	
Aurum and GOLD	179014004	277003011	208851000006112	29858	7K1D300	prim open reduction fract small bone & fixation using screw	
Aurum and GOLD	179018001	277012013	207591000006115	34764	7K1D700	prmy open red+int fxn prox fem #+screw/nail+intramed device	
Aurum and GOLD	179019009	277013015	207661000006112	23971	7K1D800	prmy open reduction #+locked reamed intramedullary nail fxtn	
Aurum and GOLD	179020003	277014014	207571000006116	37761	7K1D900	prmy open red #+locked unreamed intramedullary nail fixation	
Aurum and GOLD	179021004	277015010	207581000006118	65176	7K1DA00	prmy open red #+unlocked reamed intramedullary nail fixation	
Aurum and GOLD	179022006	277016011	207681000006119	56608	7K1DB00	prmy open reduction #+unlocked unreamed intramedullary nail	
Aurum and GOLD	179023001	277018012	207691000006116	6894	7K1DC00	prmy open reduction of #+internal fixation with k-wire	
Aurum and GOLD	179027000	277022019	207671000006117	27445	7K1Ey00	prmy open reduction #+other int(extramedullary) fixation	
Aurum and GOLD	179027000	277022019	205031000006115	8630	7K1E.00	primary open reduction fract bone & extramedullary fixation	

Aurum and GOLD	179027000	277022019	208861000006114	36449	7K1Ez00	prim open reduction fracture bone & extramedull fixation nos	
Aurum and GOLD	179027000	277022019	208651000006111	55286	7K1E300	prim open reduc fract long bone & cmplx extramedul fixat nec	
Aurum and GOLD	179029002	277028015	208661000006113	72378	7K1E112	prim open reduct # long bone & fix - parham circlage band	
Aurum and GOLD	179029002	277030018	207641000006113	10429	7K1E100	prmy open reduction #+internal fixation with cerclage wiring	
Aurum and GOLD	179030007	277031019	208781000006114	54327	7K1E200	prim open reduct fract long bone & extramedull fixatn suture	
Aurum and GOLD	179034003	277037015	207651000006110	10782	7K1E700	prmy open reduction #+internal fxn with tension band wiring	
Aurum and GOLD	179035002	277038013	207711000006118	33711	7K1E800	prmy open reduction of #+internal fixation with screw(s)	
Aurum and GOLD	179036001	277039017	207631000006115	62541	7K1E900	prmy open reduction #+int fxtn with multiple implant types	
Aurum and GOLD	179039008	277042011	204611000006115	45394	7K1F300	primary intraarticular fixation intraartic fracture bone nec	
Aurum and GOLD	179039008	277042011	205311000006111	30429	7K1F.00	primary open reduction of intraarticular fracture of bone	
Aurum and GOLD	179039008	277042011	205301000006113	53348	7K1Fy00	primary open reduction of intraarticular fracture bone os	
Aurum and GOLD	179039008	277042011	208571000006117	97111	7K1F400	prim extraarticular reduction intraartic fracture bone nec	
Aurum and GOLD	179039008	277042011	205291000006112	50460	7K1Fz00	primary open reduction of intraarticular fracture bone nos	
Aurum and GOLD	179040005	277043018	205931000006116	42223	7K1F000	primary reduction intraarticular fract bone using arthrotomy	
Aurum and GOLD	179041009	277044012	210391000006114	55995	7K1F100	primary excis intraarticular fragment intraartic fract bone	
Aurum and GOLD	179049006	277054011	277054011	49870	7K1G000	prmy open reduction of fracture and skeletal traction	

Aurum and GOLD	179050006	277055012	205141000006112	21942	7K1Gy11	primary open reduction of bone fracture & external fixation	
Aurum and GOLD	179050006	277055012	207731000006112	34396	7K1G100	prmy open reduction of fracture and external fixation	
Aurum and GOLD	179051005	277056013	205371000006119	41888	7K1G200	primary open reduction+external fixation of femoral fracture	
Aurum and GOLD	179053008	277059018	277059018	33602	7K1G400	primary open reduction of fracture and cast immobilisation	
Aurum and GOLD	179054002	277060011	277060011	63085	7K1G500	primary open reduction of fracture and functional bracing	
Aurum and GOLD	179055001	277061010	277061010	91919	7K1G600	primary open reduction of fracture and skin traction	
Aurum and GOLD	179058004	277069012	166951000006118	39646	7K1HF00	revision to open red #+other external immobilisation	History
Aurum and GOLD	179058004	277069012	277069012	9727	7K1H.00	secondary open reduction of fracture of bone	
Aurum and GOLD	179058004	277070013	277070013	54959	7K1H.11	revision to open reduction of fracture of bone	History
Aurum and GOLD	179061003	277073010	151911000006116	55447	7K1H200	secondary open reduction of intraarticular fracture of bone	
Aurum and GOLD	179064006	277077011	166991000006112	67028	7K1H500	revision to open red+ext fxtn of proximal femoral #	History
Aurum and GOLD	179066008	277079014	159521000006117	50450	7K1H700	rvsn to opn red+int fxtn prox fem #+ scrw/nl+intramed device	History
Aurum and GOLD	179067004	277080012	159511000006113	63189	7K1H800	rvsn to opn red+int fxtn prox fem #+ scrw/nail+plate device	History
Aurum and GOLD	179069001	277083014	167111000006119	101629	7K1HA00	revision to open reduction of # and cast immobilisation	History
Aurum and GOLD	179070000	277084015	167121000006110	98292	7K1HB00	revision to open reduction of # and functional bracing	History
Aurum and GOLD	179071001	277085019	277085019	113408	7K1HC00	revision to open reduction of fracture and skin traction	History
Aurum and GOLD	179073003	277088017	277088017	108001	7K1HD00	revision to open reduction of fracture and skeletal traction	History
Aurum and GOLD	179074009	277089013	277089013	59716	7K1HE00	revision to open reduction of fracture and external fixation	History

Aurum and GOLD	179076006	277092012	166931000006113	67198	7K1HG00	revision to open red #+locked reamed intramed nail fxn	History
Aurum and GOLD	179077002	277093019	166941000006115	51763	7K1HH00	revision to open red #+locked unreamed intramed nail fxn	History
Aurum and GOLD	179078007	277094013	166971000006111	55638	7K1HJ00	revision to open red #+unlocked reamed intramed nail fxn	History
Aurum and GOLD	179079004	277095014	166981000006114	98418	7K1HK00	revision to open red #+unlocked unreamed intramed nail fxn	History
Aurum and GOLD	179080001	277096010	166871000006117	28988	7K1HL00	revision to open red #+int fxn+k-wire	History
Aurum and GOLD	179081002	277097018	166911000006119	39647	7K1HM00	revision to open red #+int fxn+tension band wiring	History
Aurum and GOLD	179082009	277098011	166861000006112	93711	7K1HN00	revision to open red #+int fxn+cerclage wiring	History
Aurum and GOLD	179083004	277099015	166901000006117	22167	7K1HP00	revision to open red #+int fxn+screw(s)	History
Aurum and GOLD	179084005	277100011	166891000006116	39352	7K1HQ00	revision to open red #+int fxn+plate	History
Aurum and GOLD	179087003	277103013	166921000006110	52910	7K1HT00	revision to open red #+intramedullary nail fxn	History
Aurum and GOLD	179094000	277114011	566971000006116	12823	7K1J300	closed reduction fracture small bone & fixation using screw	
Aurum and GOLD	179095004	277115012	570601000006111	34988	7K1J400	cls red fragment bone+fxn using screw	
Aurum and GOLD	179097007	277117016	210941000006118	44594	7K1J600	primary int fxn(no red) prox fem #+scrw/nail+intramed device	
Aurum and GOLD	179098002	277118014	210931000006111	38856	7K1J700	primary int fxn(no red) prox fem #+screw/nail+plate device	
Aurum and GOLD	179100002	277120012	159501000006110	48973	7K1J900	rvsn to int fxn(no red) prox fem #+screw/nail+intramed dev	History
Aurum and GOLD	179102005	277122016	167981000006112	37998	7K1JA00	revisn to int fxn(no red) prox fem #+screw/nail+plate device	History
Aurum and GOLD	179102005	277122016	167971000006114	43403	7K1J800	revisn to int fxn(no red) prox fem #+screw/nail device alone	History
Aurum and GOLD	179104006	277124015	208561000006112	54819	7K1JC00	prim cls rd+int fxn prox fem #+screw/nail+intramdulry device	
Aurum and GOLD	179105007	277125019	210041000006119	46959	7K1JD00	primary cls red+int fxn prox fem #+screw/nail+plate device	

Aurum and GOLD	179106008	277126018	159481000006117	63554	7K1JE00	rvsn to cls red+int fxn prox fem #+screw/nail device alone	History
Aurum and GOLD	179106008	277126018	1702401000006118	107358	7K1Y000	remanip intracap fract neck fem and fix using nail or screw	History
Aurum and GOLD	179107004	277127010	159351000006118	69857	7K1JF00	rvsn cls red+int fxn prox fem #+screw/nail+intramed device	History
Aurum and GOLD	179108009	277128017	159491000006119	34846	7K1JG00	rvsn to cls red+int fxn prox fem #+screw/nail+plate device	History
Aurum and GOLD	179109001	277129013	277129013	22393	7K1JH00	primary wire fixation of fracture	
Aurum and GOLD	179110006	277130015	277130015	30748	7K1JJ00	revision to wire fixation of fracture	History
Aurum and GOLD	179111005	277131016	277131016	34355	7K1JK00	primary closed reduction of fracture and wire fixation	
Aurum and GOLD	179111005	277131016	209701000006112	50532	7K1JR00	primary closed reduction #+internal fixation with wire	
Aurum and GOLD	179112003	277132011	277132011	44899	7K1JL00	revision to closed reduction of fracture and wire fixation	History
Aurum and GOLD	179112003	277132011	166511000006112	39010	7K1JY00	revision to closed reduction # + internal fixation with wire	History
Aurum and GOLD	179113008	277133018	210071000006110	68151	7K1JM00	primary cls reduction #+locked reamed intramed nail fxn	
Aurum and GOLD	179114002	277134012	210081000006113	64873	7K1JN00	primary cls reduction #+locked unreamed intramed nail fxn	
Aurum and GOLD	179115001	277135013	210091000006111	59169	7K1JP00	primary cls reduction #+unlocked reamed intramed nail fxn	
Aurum and GOLD	179116000	277136014	210101000006117	95501	7K1JQ00	primary cls reduction #+unlocked unreamed intramed nail fxn	
Aurum and GOLD	179118004	277138010	209691000006112	29953	7K1JS00	primary closed reduction #+internal fixation with screw(s)	
Aurum and GOLD	179120001	277140017	171451000006112	104627	7K1JU00	revision cls reduction #+locked reamed intramed nail fxn	History
Aurum and GOLD	179121002	277141018	171461000006114	110049	7K1JV00	revision cls reduction #+locked unreamed intramed nail fxn	History

Aurum and GOLD	179122009	277142013	171471000006119	110998	7K1JW00	revision cls reduction #+unlocked reamed intramed nail fxn	History
Aurum and GOLD	179123004	277143015	171481000006116	99152	7K1JX00	revision cls reduction #+unlocked unreamed intramed nail fxn	History
Aurum and GOLD	179125006	277145010	166571000006115	33559	7K1JZ00	revision to closed reduction #+internal fixation with screws	History
Aurum and GOLD	179127003	277147019	209711000006110	8685	7K1Jb00	primary closed reduction #+intramed nail fixation	
Aurum and GOLD	179128008	277148012	166581000006117	36816	7K1Jc00	revision to closed reduction #+intramed nail fixation	History
Aurum and GOLD	179134001	277154013	566891000006111	59570	7K1K100	closed reduct fract bone and fixat functional bracing system	
Aurum and GOLD	179136004	277156010	210531000006116	70018	7K1K300	primary external fixation(without reduction) prox femoral #	
Aurum and GOLD	179137008	277157018	166821000006118	102104	7K1K400	revision to ext fxn(without reduction) proximal femoral #	History
Aurum and GOLD	179138003	277158011	210151000006118	102313	7K1K500	primary cls reduction+external fixation proximal femoral #	
Aurum and GOLD	179140008	277160013	277160013	69702	7K1K700	primary functional bracing of fracture	
Aurum and GOLD	179141007	277161012	277161012	11333	7K1K800	primary external fixation of fracture	
Aurum and GOLD	179143005	277164016	277164016	96903	7K1KA00	revision to functional bracing of fracture	History
Aurum and GOLD	179144004	277165015	277165015	12165	7K1KB00	revision to external fixation of fracture	History
Aurum and GOLD	179147006	277169014	277169014	29103	7K1KE00	primary closed reduction of fracture and external fixation	
Aurum and GOLD	179149009	277171014	166661000006111	38988	7K1KG00	revision to closed reduction of # and external fixation	History
Aurum and GOLD	179159005	277185016	277185016	6660	7K1L400	closed reduction of fracture of hip	
Aurum and GOLD	179160000	277186015	277186015	8800	7K1L600	closed reduction of fracture of knee	
Aurum and GOLD	179161001	277187012	277187012	5886	7K1L700	closed reduction of fracture of tibia and or fibula	
Aurum and GOLD	179162008	277188019	277188019	6106	7K1L800	closed reduction of fracture of ankle	
Aurum and GOLD	179163003	277189010	277189010	38433	7K1L900	closed reduction of fracture of metatarsus	
Aurum and GOLD	179165005	277191019	277191019	11453	7K1LB00	closed reduction of fracture of hallux	
Aurum and GOLD	179166006	277192014	277192014	7034	7K1LE00	closed reduction of fracture of elbow	

Aurum and GOLD	179167002	277193016	277193016	7428	7K1LG00	closed reduction of fracture of shoulder	
Aurum and GOLD	179168007	277194010	277194010	6069	7K1LH00	closed reduction of fracture of finger	
Aurum and GOLD	179169004	277195011	277195011	8885	7K1LJ00	closed reduction of fracture of thumb	
Aurum and GOLD	179171004	277198013	277198013	35530	7K1LK00	closed reduction of fracture of metacarpus	
Aurum and GOLD	179172006	277199017	277199017	6942	7K1LL00	closed reduction of fracture of radius and or ulna	
Aurum and GOLD	179173001	277200019	277200019	5951	7K1LM00	closed reduction of fracture of wrist	
Aurum and GOLD	179177000	277206013	277206013	57893	7K1LT00	primary closed reduction of fracture and cast immobilisation	
Aurum and GOLD	179178005	277207016	166651000006114	61994	7K1LU00	revision to closed reduction of # and cast immobilisation	History
Aurum and GOLD	179180004	277210011	277210011	36893	7K1LW00	primary closed reduction of fracture and skin traction	
Aurum and GOLD	179183002	277213013	277213013	48219	7K1LZ00	primary skin traction of fracture	
Aurum and GOLD	179184008	277214019	277214019	24809	7K1La00	revision to skin traction of fracture	History
Aurum and GOLD	179185009	277216017	277216017	41760	7K1Lb00	primary cast immobilisation of fracture	
Aurum and GOLD	179186005	277217014	277217014	46862	7K1Lc00	revision to cast immobilisation of fracture	History
Aurum and GOLD	179186005	277217014	277166019	110066	7K1KC00	other revision to external immobilisation of fracture	History
Aurum and GOLD	179187001	277219012	277219012	38472	7K1Ld00	primary arthroscopic reduction of fracture	
Aurum and GOLD	179188006	277220018	277220018	32012	7K1Le00	primary arthroscopic reduction and fixation of fracture	
Aurum and GOLD	179189003	277221019	277221019	35742	7K1Lf00	revision to arthroscopic reduction of fracture	History
Aurum and GOLD	179190007	277222014	277222014	34743	7K1Lg00	revision to arthroscopic reduction and fixation of fracture	History
Aurum and GOLD	179212005	277259010	277259010	73812	7K1N900	primary skeletal traction of fracture	
Aurum and GOLD	179597004	277882015	1554021000006116	59690	7K6FQ00	primary open reduction # dislocation joint internal fix nec	
Aurum and GOLD	179598009	277883013	206771000006112	35905	7K6F000	primry opn redctn of # dislocation of jt + skeletal traction	
Aurum and GOLD	179613009	277902016	205061000006112	66539	7K6FF00	primary open reduction of # dislocation+cast immobilisation	

Aurum and GOLD	179614003	277903014	205101000006110	111333	7K6FG00	primary open reduction of # dislocation+functional bracing	
Aurum and GOLD	179616001	277905019	205121000006117	40403	7K6FJ00	primary open reduction of # dislocation+wire fixation	
Aurum and GOLD	179617005	277906018	205071000006117	38426	7K6FK00	primary open reduction of # dislocation+external fixation	
Aurum and GOLD	179619008	277909013	205091000006116	42960	7K6FM00	primary open reduction of # dislocation+fixation+screw(s)	
Aurum and GOLD	179620002	277910015	205081000006119	65614	7K6FN00	primary open reduction of # dislocation+fixation+plate(s)	
Aurum and GOLD	179625007	277916014	209661000006116	28149	7K6G000	primary closed reduction # dislocation jnt+skeletal traction	
Aurum and GOLD	179636000	277929014	566961000006111	22144	7K6GN00	closed reduction fracture disloc joint & internal fixation	
Aurum and GOLD	179646003	277942014	209781000006115	98158	7K6GY00	primary closed reduction of # dislocation+cast immobil	
Aurum and GOLD	179649005	277945011	209671000006111	48332	7K6Gb00	primary closed reduction # dislocation+fixation by wire(s)	
Aurum and GOLD	179650005	277946012	209791000006117	101993	7K6Gc00	primary closed reduction of # dislocation+external fixation	
Aurum and GOLD	179655000	277952013	167051000006117	50413	7K6H000	revision to open reduction # dislocation+skeletal traction	History
Aurum and GOLD	179663004	277963011	151871000006119	36783	7K6H700	secondary open reduction fracture disloc joint & fixation	
Aurum and GOLD	179663004	277963011	1589031000006110	85656	7K6Hh00	sec open red fracture dislocat joint and intern fixation nec	
Aurum and GOLD	179691007	277999018	167141000006115	96575	7K6Hb00	revision to open reduction of # dislocation+fixation+wire(s)	History
Aurum and GOLD	179694004	278003014	167021000006114	110164	7K6He00	revision to open reduction # dislocation+fixation+screw(s)	History
Aurum and GOLD	18171007	30690013	319052019	6299	S263.00	fracture of other finger	
Aurum and GOLD	18171007	478502010	478502010	5260	S26..11	finger fracture	
Aurum and GOLD	182704006	282456019	282456019	101973	8752.00	plugging of flail chest	

Aurum and GOLD	19259001	1494944016	1494944016	44538	S230A00	closed fracture radius and ulna, proximal	
Aurum and GOLD	19259001	32476011	318820013	42957	S230z00	closed fracture of proximal forearm not otherwise specified	
Aurum and GOLD	19259001	479288015	479288015	43570	S230.00	closed fracture of proximal radius and ulna	
Aurum and GOLD	19652000	33084011	319171015	49595	S313100	open fracture of femoral condyle, unspecified	
Aurum and GOLD	19688005	33145016	276481010	34634	7J42100	open reduction of fracture of spine nec	
Aurum and GOLD	198741000000106	299771000000110	299771000000110	45779	S300A00	closed fracture of femur, upper epiphysis	
Aurum and GOLD	198751000000109	299791000000114	299791000000114	96518	S301A00	open fracture of femur, upper epiphysis	
Aurum and GOLD	20013001	33686012	276107015	63064	7J03y00	other specified reduction of fracture of facial bone	
Aurum and GOLD	20013001	33686012	276108013	28621	7J03z00	reduction of fracture of facial bone nos	
Aurum and GOLD	20013001	33686012	33686012	15800	7J03.00	reduction of fracture of facial bone	
Aurum and GOLD	20100009	33823015	570541000006112	39984	S300000	cls # prox femur, intracapsular section, unspecified	
Aurum and GOLD	20121009	33856019	33856019	90494	S120700	closed fracture of seven ribs	
Aurum and GOLD	202831001	311305016	311305016	16895	N1y1.00	fatigue fracture of vertebra	
Aurum and GOLD	203444008	312156018	312156018	39334	N331200	postoophorectomy osteoporosis with pathological fracture	
Aurum and GOLD	203445009	312157010	312157010	33526	N331300	osteoporosis of disuse with pathological fracture	
Aurum and GOLD	203446005	312158017	212441000006112	68019	N331400	postsurgical malabsorption osteoporosis with path fracture	
Aurum and GOLD	203447001	312159013	312159013	46894	N331500	drug-induced osteoporosis with pathological fracture	
Aurum and GOLD	203448006	312160015	312160015	27597	N331600	idiopathic osteoporosis with pathological fracture	
Aurum and GOLD	203449003	312161016	312161016	54834	N331700	fracture of bone in neoplastic disease	
Aurum and GOLD	203450003	312162011	40881000006116	17377	N331800	osteoporosis + pathological fracture lumbar vertebrae	
Aurum and GOLD	203451004	312163018	40891000006118	12673	N331900	osteoporosis + pathological fracture thoracic vertebrae	
Aurum and GOLD	203452006	312164012	40871000006119	48772	N331A00	osteoporosis + pathological fracture cervical vertebrae	

Aurum and GOLD	203453001	312165013	312165013	38395	N331B00	postmenopausal osteoporosis with pathological fracture	
Aurum and GOLD	20433007	34376013	34376013	6917	S336.00	fracture of upper end of tibia	
Aurum and GOLD	20511007	480728016	480728016	12516	S2B..00	fracture of bone of hand	
Aurum and GOLD	20701002	34832014	157231000006111	56080	7K1H100	second open reduct fract bone & extramedullary fixation hfq	
Aurum and GOLD	20701002	34832014	157241000006118	28533	7K1H000	second open reduct fract bone & intramedullary fixation hfq	
Aurum and GOLD	20701002	34832014	277104019	28081	7K1Hy00	other specified secondary open reduction of fracture of bone	
Aurum and GOLD	20701002	34832014	277105018	62029	7K1Hz00	secondary open reduction of fracture of bone nos	
Aurum and GOLD	207687004	318117015	560311000006112	62841	S001600	closed #skull vlt + intracranial injury, loc unspec duration	
Aurum and GOLD	207687004	318117015	318117015	27657	S001.00	closed fracture vault of skull with intracranial injury	
Aurum and GOLD	207687004	318117015	560421000006110	59959	S001z00	closed #skull vlt with intracranial injury+concussion unspec	
Aurum and GOLD	207687004	318117015	560331000006118	17958	S001000	closed #skull vlt + intracranial injury, unspec state consc	
Aurum and GOLD	207689001	318119017	560321000006116	99018	S001100	closed #skull vlt + intracranial injury, no loss of consc	
Aurum and GOLD	207690005	318120011	560281000006110	98776	S001200	closed #skull vlt + intracranial injury, <1hr loss of consc	
Aurum and GOLD	207691009	318121010	560301000006114	93851	S001300	closed #skull vlt + intracranial injury, 1-24hr loss consc	
Aurum and GOLD	207692002	318122015	560291000006113	72412	S001400	closed #skull vlt + intracranial injury, >24hr loc+recovery	
Aurum and GOLD	207696004	318126017	263861000006117	66312	S002100	open #skull vlt no intracranial injury, no loss of consc	
Aurum and GOLD	207696004	318126017	318126017	57246	S002.00	open fracture vault of skull without intracranial injury	
Aurum and GOLD	207696004	318126017	263871000006112	66114	S002000	open #skull vlt no intracranial injury, unspec state consc	
Aurum and GOLD	207705002	318135012	263771000006115	61357	S003600	open #skull vlt + intracranial injury, loc unspec duration	

Aurum and GOLD	207705002	318135012	263791000006119	64167	S003000	open #skull vlt + intracranial injury, unspec state of consc	
Aurum and GOLD	207705002	318135012	318135012	51299	S003.00	open fracture vault of skull with intracranial injury	
Aurum and GOLD	207705002	318135012	263881000006110	62743	S003z00	open #skull vlt with intracranial injury + concussion unspec	
Aurum and GOLD	207707005	318137016	263781000006117	73441	S003100	open #skull vlt + intracranial injury, no loss of consc	
Aurum and GOLD	207710003	318140016	52931000006111	104726	S003400	open #skull vlt + intracranial injury, >24hr loc + recovery	
Aurum and GOLD	207727006	318171013	560021000006119	112568	S011100	closed #skull bse + intracranial injury, no loss of consc	
Aurum and GOLD	207728001	318172018	559981000006117	112563	S011200	closed #skull bse + intracranial injury, <1hr loss of consc	
Aurum and GOLD	207729009	318173011	560001000006112	105137	S011300	closed #skull bse + intracranial injury, 1-24hr loss consc	
Aurum and GOLD	207730004	318174017	559991000006119	71725	S011400	closed #skull bse + intracranial injury, >24hr loc+recovery	
Aurum and GOLD	207746007	318190017	264121000006115	99505	S013200	open #skull bse + intracranial injury, <1hr loss of consc	
Aurum and GOLD	207748008	318192013	264131000006117	73451	S013400	open #skull bse + intracranial injury, >24hr loc + recovery	
Aurum and GOLD	207753003	318197019	318197019	2642	S022.00	fracture of mandible, closed	
Aurum and GOLD	207753003	318197019	792811000006115	62716	S022.11	fracture of inferior maxilla, closed	
Aurum and GOLD	207753003	318197019	318209017	36268	S022z00	fracture of mandible, closed, nos	
Aurum and GOLD	207753003	318197019	318203016	28913	S022400	closed fracture of mandible, ramus, unspecified	
Aurum and GOLD	207753003	318197019	318199016	29091	S022000	closed fracture mandible (site unspecified)	
Aurum and GOLD	207753003	318197019	318207015	59006	S022800	closed fracture of mandible, body, other and unspecified	
Aurum and GOLD	207753003	318198012	318198012	16890	S022.12	fracture of lower jaw, closed	
Aurum and GOLD	207755005	318200018	318200018	12179	S022100	closed fracture of mandible, condylar process	
Aurum and GOLD	207757002	318202014	318202014	59341	S022300	closed fracture of mandible, coronoid process	

Aurum and GOLD	207759004	318204010	318204010	41707	S022500	closed fracture of mandible, angle of jaw	
Aurum and GOLD	207760009	318205011	318205011	71583	S022600	closed fracture of mandible, symphysis of body	
Aurum and GOLD	207763006	318208013	318208013	37904	S022x00	closed fracture of mandible, multiple sites	
Aurum and GOLD	207767007	318213012	318213012	60633	S023100	open fracture of mandible, condylar process	
Aurum and GOLD	207768002	318214018	318214018	99549	S023200	open fracture of mandible, subcondylar	
Aurum and GOLD	207769005	318215017	318215017	112595	S023300	open fracture of mandible, coronoid process	
Aurum and GOLD	207771005	318217013	318217013	55955	S023500	open fracture of mandible, angle of jaw	
Aurum and GOLD	207776000	318223015	318223015	54553	S023x00	open fracture of mandible, multiple sites	
Aurum and GOLD	207782002	318233011	318233011	32011	S025000	open fracture maxilla	
Aurum and GOLD	207785000	318236015	325294016	109772	Syu0300	[x]fractures of other skull and facial bones	
Aurum and GOLD	207785000	318236015	325295015	94692	Syu0400	[x]fracture of skull and facial bones, part unspecified	
Aurum and GOLD	207785000	318236015	318236015	30203	S028.00	fracture of skull and facial bones	
Aurum and GOLD	207787008	318238019	318238019	20515	S028100	fracture of orbital floor	
Aurum and GOLD	207788003	318239010	318239010	30028	S028200	fracture of malar and maxillary bones	
Aurum and GOLD	207794006	318245019	318245019	27287	S02x000	fracture of alveolus, closed	
Aurum and GOLD	207798009	318249013	318249013	49840	S02y000	fracture of alveolus, open	
Aurum and GOLD	207800002	318251012	318251012	104738	S02y200	fracture of palate, open	
Aurum and GOLD	207881000	318341014	318341014	33515	S044.00	multiple fractures involving skull and facial bones	
Aurum and GOLD	207884008	318344018	318344018	10252	S1...00	fracture of neck and trunk	
Aurum and GOLD	207884008	318344018	318729017	25284	S1z..00	fracture of neck and trunk nos	
Aurum and GOLD	207895002	318375010	318375010	98393	S100800	closed fracture atlas, isolated arch or articular process	
Aurum and GOLD	207896001	318376011	318376011	69974	S100900	closed fracture atlas, comminuted	
Aurum and GOLD	207897005	318377019	318377019	39887	S100A00	closed fracture axis, odontoid process	
Aurum and GOLD	207898000	318378012	318378012	105702	S100B00	closed fracture axis, spondylolysis	
Aurum and GOLD	207899008	318379016	318379016	42149	S100C00	closed fracture axis, spinous process	
Aurum and GOLD	207900003	318380018	318380018	94292	S100D00	closed fracture axis, transverse process	
Aurum and GOLD	207901004	318381019	318381019	95006	S100E00	closed fracture axis, posterior arch	
Aurum and GOLD	207903001	318383016	318383016	67973	S100G00	closed fracture cervical vertebra, burst	

Aurum and GOLD	207904007	318384010	318384010	53337	S100H00	closed fracture cervical vertebra, wedge	
Aurum and GOLD	207905008	318385011	318385011	95620	S100J00	closed fracture cervical vertebra, spondylolysis	
Aurum and GOLD	207906009	318386012	318386012	54299	S100K00	closed fracture cervical vertebra, spinous process	
Aurum and GOLD	207907000	318387015	318387015	64297	S100L00	closed fracture cervical vertebra, transverse process	
Aurum and GOLD	207908005	318388013	318388013	95513	S100M00	closed fracture cervical vertebra, posterior arch	
Aurum and GOLD	207910007	318390014	318390014	55346	S100x00	multiple closed fractures of cervical vertebrae	
Aurum and GOLD	207922005	318419013	318419013	110732	S101900	open fracture atlas, comminuted	
Aurum and GOLD	207923000	318420019	318420019	94844	S101A00	open fracture axis, odontoid process	
Aurum and GOLD	207926008	318423017	318423017	112905	S101D00	open fracture axis, transverse process	
Aurum and GOLD	207936000	318433013	318433013	72617	S101x00	multiple open fractures of cervical vertebrae	
Aurum and GOLD	207938004	318435018	318435018	27404	S102.00	closed fracture thoracic vertebra	
Aurum and GOLD	207938004	318435018	318443011	11770	S102y00	other specified closed fracture thoracic vertebra	
Aurum and GOLD	207938004	318435018	318445016	41138	S102z00	closed fracture thoracic vertebra not otherwise specified	
Aurum and GOLD	207939007	318436017	318436017	39816	S102000	closed fracture thoracic vertebra, burst	
Aurum and GOLD	207940009	318437014	318437014	28524	S102100	closed fracture thoracic vertebra, wedge	
Aurum and GOLD	207941008	318438016	318438016	96659	S102200	closed fracture thoracic vertebra, spondylolysis	
Aurum and GOLD	207942001	318439012	318439012	64872	S102300	closed fracture thoracic vertebra, spinous process	
Aurum and GOLD	207943006	318440014	318440014	48886	S102400	closed fracture thoracic vertebra, transverse process	
Aurum and GOLD	207944000	318441013	318441013	99516	S102500	closed fracture thoracic vertebra, posterior arch	
Aurum and GOLD	207949005	318446015	318446015	63253	S103.00	open fracture thoracic vertebra	
Aurum and GOLD	207951009	318448019	318448019	62047	S103100	open fracture thoracic vertebra, wedge	
Aurum and GOLD	207955000	318452019	318452019	101318	S103500	open fracture thoracic vertebra, posterior arch	
Aurum and GOLD	207957008	318454018	318454018	3888	S104.00	closed fracture lumbar vertebra	
Aurum and GOLD	207958003	318455017	318455017	42968	S104000	closed fracture lumbar vertebra, burst	
Aurum and GOLD	207959006	318456016	318456016	8266	S104100	closed fracture lumbar vertebra, wedge	

Aurum and GOLD	207960001	318457013	318457013	61150	S104200	closed fracture lumbar vertebra, spondylolysis	
Aurum and GOLD	207961002	318458015	318458015	35096	S104300	closed fracture lumbar vertebra, spinous process	
Aurum and GOLD	207962009	318459011	318459011	29089	S104400	closed fracture lumbar vertebra, transverse process	
Aurum and GOLD	207963004	318460018	318460018	95842	S104500	closed fracture lumbar vertebra, posterior arch	
Aurum and GOLD	207964005	318461019	318461019	95585	S104600	closed fracture lumbar vertebra, tricolumnar	
Aurum and GOLD	207965006	318462014	318462014	42780	S105.00	open fracture lumbar vertebra	
Aurum and GOLD	207966007	318463016	318463016	73601	S105000	open fracture lumbar vertebra, burst	
Aurum and GOLD	207967003	318464010	318464010	65302	S105100	open fracture lumbar vertebra, wedge	
Aurum and GOLD	207970004	318467015	318467015	105695	S105400	open fracture lumbar vertebra, transverse process	
Aurum and GOLD	207974008	318473019	318473019	15877	S106.00	closed fracture sacrum	
Aurum and GOLD	207975009	318474013	318474013	72404	S106000	closed compression fracture sacrum	
Aurum and GOLD	207976005	318475014	318475014	72600	S106100	closed vertical fracture of sacrum	
Aurum and GOLD	207977001	318476010	318476010	66434	S107.00	open fracture sacrum	
Aurum and GOLD	207978006	318477018	318477018	105935	S107000	open compression fracture sacrum	
Aurum and GOLD	207979003	318478011	318478011	97354	S107100	open vertical fracture of sacrum	
Aurum and GOLD	207980000	318479015	318479015	14834	S108.00	closed fracture pelvis, coccyx	
Aurum and GOLD	207981001	318480017	318480017	55280	S109.00	open fracture pelvis, coccyx	
Aurum and GOLD	207983003	318482013	318482013	28133	S10A000	fracture of first cervical vertebra	
Aurum and GOLD	207984009	318483015	318483015	34403	S10A100	fracture of second cervical vertebra	
Aurum and GOLD	207985005	318484014	318484014	19189	S10A200	multiple fractures of cervical spine	
Aurum and GOLD	207986006	318485010	318485010	12406	S10B.00	fracture of lumbar spine and pelvis	
Aurum and GOLD	207993005	318492017	318492017	8613	S10B600	multiple fractures of lumbar spine and pelvis	
Aurum and GOLD	207998001	318500017	318500017	52300	S110.00	closed fracture of cervical spine with cord lesion	
Aurum and GOLD	207998001	318500017	562751000006110	73416	S110z00	closed fracture of cervical spine with cord lesion nos	
Aurum and GOLD	208000003	318502013	570741000006119	109377	S110100	cls spinal fracture with complete cervcl cord lesion, c1-4	
Aurum and GOLD	208006009	318508012	570751000006117	102735	S110700	cls spinal fracture with complete cervcl cord lesion, c5-7	

Aurum and GOLD	208007000	318509016	570681000006119	96514	S110800	cls spinal fracture with anterior cervcl cord lesion, c5-7	
Aurum and GOLD	208012004	318514017	318514017	69432	S111.00	open fracture of cervical spine with spinal cord lesion	
Aurum and GOLD	208026008	318528011	563961000006116	73611	S112z00	closed fracture of thoracic spine with cord lesion nos	
Aurum and GOLD	208026008	318528011	318528011	35849	S112.00	closed fracture of thoracic spine with spinal cord lesion	
Aurum and GOLD	208028009	318530013	570861000006113	48958	S112100	cls spinal fracture wth complete thoracic cord lesion,t1-6	
Aurum and GOLD	208034002	318536019	570761000006115	31545	S112700	cls spinal fracture with complete thorac cord lesion, t7-12	
Aurum and GOLD	208036000	318538018	570731000006112	113268	S112900	cls spinal fracture with central thoracid cord lesion, t7-12	
Aurum and GOLD	208037009	318539014	570801000006112	102043	S112A00	cls spinal fracture with posterior thorac cord lesion, t7-12	
Aurum and GOLD	208040009	318542015	318542015	60615	S113.00	open fracture of thoracic spine with spinal cord lesion	
Aurum and GOLD	208051009	318553014	43191000006118	104755	S113A00	opn spinal fracture with posterior thorac cord lesion, t7-12	
Aurum and GOLD	208054001	318556018	318556018	44059	S114.00	closed fracture of lumbar spine with spinal cord lesion	
Aurum and GOLD	208056004	318558017	318558017	95529	S114100	closed spinal fracture with complete lumbar cord lesion	
Aurum and GOLD	208060001	318562011	318562011	73788	S114500	closed spinal fracture with cauda equina lesion	
Aurum and GOLD	208061002	318563018	318563018	94189	S115.00	open fracture of lumbar spine with spinal cord lesion	
Aurum and GOLD	208069000	318571019	318580019	99376	S116z00	closed fracture of sacrum with spinal cord lesion nos	
Aurum and GOLD	208069000	318571019	318571019	57444	S116.00	closed fracture of sacrum with spinal cord lesion	
Aurum and GOLD	208076005	318581015	318581015	96473	S117.00	open fracture of sacrum with spinal cord lesion	
Aurum and GOLD	208076005	318581015	318585012	94584	S117300	open fracture of sacrum with other spinal cord injury	
Aurum and GOLD	208082008	318587016	318590010	113065	S118200	closed fracture of coccyx with other cauda equina injury	

Aurum and GOLD	208082008	318587016	318587016	51018	S118.00	closed fracture of coccyx with spinal cord lesion	
Aurum and GOLD	208082008	318587016	318592019	101517	S118z00	closed fracture of coccyx with spinal cord lesion nos	
Aurum and GOLD	208122008	318627013	318627013	71567	S125.00	closed fracture larynx and trachea	
Aurum and GOLD	208130009	318635011	318635011	96643	S126100	open fracture of hyoid bone	
Aurum and GOLD	208136003	318641016	318641016	28538	S127100	cough fracture of ribs	
Aurum and GOLD	208136003	318642011	318642011	8968	S120A00	cough fracture	
Aurum and GOLD	208145002	318654011	318654011	738	S13..00	fracture or disruption of pelvis	
Aurum and GOLD	208146001	318655012	318655012	72525	S130000	closed fracture acetabulum, anterior lip alone	
Aurum and GOLD	208147005	318656013	318656013	94649	S130100	closed fracture acetabulum, posterior lip alone	
Aurum and GOLD	208148000	318657016	318657016	73479	S130200	closed fracture acetabulum, anterior column	
Aurum and GOLD	208149008	318658014	318658014	57923	S130300	closed fracture acetabulum, posterior column	
Aurum and GOLD	208150008	318659018	318659018	69418	S130400	closed fracture acetabulum, floor	
Aurum and GOLD	208164008	318673010	318679014	38895	S132y00	other specified closed fracture pubis	
Aurum and GOLD	208164008	318673010	318673010	5302	S132.00	closed fracture pubis	
Aurum and GOLD	208164008	318673010	318680012	28702	S132z00	closed fracture pubis nos	
Aurum and GOLD	208165009	318674016	318674016	7004	S132000	closed fracture pelvis, single pubic ramus	
Aurum and GOLD	208166005	318675015	318675015	6667	S132100	closed fracture pelvis, multiple pubic rami - stable	
Aurum and GOLD	208168006	318678018	318678018	46592	S132200	closed fracture pelvis, multiple pubic rami - unstable	
Aurum and GOLD	208172005	318682016	318682016	34685	S133000	open fracture pelvis, single pubic ramus	
Aurum and GOLD	208173000	318683014	318683014	51038	S133100	open fracture pelvis, multiple pubic rami - stable	
Aurum and GOLD	208174006	318684015	318684015	101447	S133200	open fracture pelvis, multiple pubic rami - unstable	
Aurum and GOLD	208181004	318691017	318691017	41698	S134300	closed fracture pelvis, ischial tuberosity	
Aurum and GOLD	208182006	318692012	318692012	28234	S134400	closed fracture pelvis, anterior superior iliac spine	
Aurum and GOLD	208183001	318693019	318693019	40587	S134500	closed fracture pelvis, anterior inferior iliac spine	
Aurum and GOLD	208184007	318694013	318694013	27854	S134600	closed fracture pelvis, iliac wing	
Aurum and GOLD	208185008	318695014	318695014	52470	S134700	closed vertical fracture of ilium	

Aurum and GOLD	208186009	318696010	561821000006117	34195	S134800	closed fracture dislocation of sacro-iliac joint	
Aurum and GOLD	208192003	318702010	318702010	68763	S135300	open fracture pelvis, ischial tuberosity	
Aurum and GOLD	208193008	318703017	318703017	43448	S135400	open fracture pelvis, anterior superior iliac spine	
Aurum and GOLD	208194002	318704011	318704011	112187	S135500	open fracture pelvis, anterior inferior iliac spine	
Aurum and GOLD	208195001	318705012	318705012	67669	S135600	open fracture pelvis, iliac wing	
Aurum and GOLD	208198004	318708014	259761000006117	94655	S135800	open fracture dislocation of sacro-iliac joint	
Aurum and GOLD	208215006	318725011	318725011	11277	S150.00	multiple fractures of thoracic spine	
Aurum and GOLD	208225001	318736016	318736016	34907	S210200	closed fracture scapula, coracoid	
Aurum and GOLD	208226000	318737013	318737013	4029	S210300	closed fracture scapula, glenoid	
Aurum and GOLD	208227009	318738015	318738015	5344	S210400	closed fracture scapula, blade	
Aurum and GOLD	208228004	318739011	318739011	48859	S210500	closed fracture scapula, spine	
Aurum and GOLD	208229007	318740013	318740013	36332	S210600	closed fracture scapula, neck	
Aurum and GOLD	208232005	318743010	318743010	64021	S211200	open fracture scapula, coracoid	
Aurum and GOLD	208233000	318744016	318744016	60108	S211300	open fracture scapula, glenoid	
Aurum and GOLD	208234006	318745015	318745015	73109	S211400	open fracture scapula, blade	
Aurum and GOLD	208236008	318747011	318747011	71953	S211600	open fracture scapula, neck	
Aurum and GOLD	208240004	318751013	318751013	11313	S220100	closed fracture proximal humerus, neck	
Aurum and GOLD	208241000	318752018	318752018	33489	S220200	closed fracture of proximal humerus, anatomical neck	
Aurum and GOLD	208242007	318753011	318753011	11044	S220300	closed fracture proximal humerus, greater tuberosity	
Aurum and GOLD	208244008	318755016	318755016	52406	S220500	closed fracture of humerus, upper epiphysis	
Aurum and GOLD	208245009	318756015	318756015	40330	S220600	closed fracture proximal humerus, three part	
Aurum and GOLD	208246005	318757012	318757012	29137	S220700	closed fracture proximal humerus, four part	
Aurum and GOLD	208250003	318763015	318763015	53622	S221100	open fracture proximal humerus, neck	
Aurum and GOLD	208251004	318764014	318764014	71207	S221200	open fracture of proximal humerus, anatomical neck	
Aurum and GOLD	208253001	318767019	318767019	48239	S221300	open fracture proximal humerus, greater tuberosity	
Aurum and GOLD	208254007	318768012	318768012	70486	S221400	open fracture proximal humerus, head	

Aurum and GOLD	208255008	318769016	318769016	59943	S221500	open fracture of humerus, upper epiphysis	
Aurum and GOLD	208256009	318770015	318770015	70604	S221600	open fracture proximal humerus, three part	
Aurum and GOLD	208257000	318771016	318771016	70653	S221700	open fracture proximal humerus, four part	
Aurum and GOLD	208267005	318784013	318784013	18394	S224200	closed fracture distal humerus, lateral condyle	
Aurum and GOLD	208270009	318787018	318787018	52083	S224500	closed fracture of distal humerus, trochlea	
Aurum and GOLD	208271008	318788011	318788011	8661	S224600	closed fracture distal humerus, lateral epicondyle	
Aurum and GOLD	208272001	318789015	318789015	28393	S224800	closed fracture distal humerus, capitellum	
Aurum and GOLD	208273006	318790012	318790012	53677	S224900	closed fracture distal humerus, bicondylar (t-y fracture)	
Aurum and GOLD	208274000	318791011	318791011	62833	S224x00	closed fracture of distal humerus, multiple	
Aurum and GOLD	208281007	318800012	318800012	63899	S225500	open fracture of distal humerus, trochlea	
Aurum and GOLD	208283005	318802016	318802016	48217	S225600	open fracture distal humerus, lateral epicondyle	
Aurum and GOLD	208283005	318802016	259861000006110	60163	S225200	open fracture distal humerus, lateral condyle	
Aurum and GOLD	208284004	318803014	318803014	32646	S225800	open fracture distal humerus, capitellum	
Aurum and GOLD	208285003	318804015	318804015	97820	S225900	open fracture distal humerus, bicondylar (t-y fracture)	
Aurum and GOLD	208286002	318805019	318805019	99325	S225x00	open fracture of distal humerus, multiple	
Aurum and GOLD	208294009	318815013	318815013	9538	S230100	closed fracture olecranon, extra-articular	
Aurum and GOLD	208295005	318816014	318816014	34371	S230400	closed fracture of proximal ulna, comminuted	
Aurum and GOLD	208296006	318817017	318817017	34370	S230800	closed fracture proximal radius, comminuted	
Aurum and GOLD	208298007	318819019	318819019	12063	S230B00	closed fracture olecranon, intra-articular	
Aurum and GOLD	208301006	318822017	318822017	29152	S231100	open fracture olecranon, extra-articular	
Aurum and GOLD	208303009	318824016	318824016	112944	S231400	open fracture proximal ulna, comminuted	
Aurum and GOLD	208304003	318825015	318825015	72408	S231800	open fracture proximal radius, comminuted	
Aurum and GOLD	208306001	318827011	318827011	8410	S231B00	open fracture olecranon, intra-articular	
Aurum and GOLD	208309008	318830016	318830016	35031	S232300	closed fracture radius and ulna, middle	

Aurum and GOLD	208312006	318833019	318833019	48245	S233300	open fracture radius and ulna, middle	
Aurum and GOLD	208318005	318845011	318845011	42076	S234400	closed fracture of ulna, lower epiphysis	
Aurum and GOLD	208321007	318848013	318848013	52389	S234111	smith's fracture - closed	
Aurum and GOLD	208321007	318848013	318849017	2862	S234700	closed smith's fracture	
Aurum and GOLD	208322000	318850017	318850017	40268	S234800	closed galeazzi fracture	
Aurum and GOLD	208323005	318851018	318851018	11066	S234900	closed volar barton's fracture	
Aurum and GOLD	208323005	318852013	569501000006112	65636	S234912	closed volar barton fracture-subluxation	
Aurum and GOLD	208323005	318853015	569521000006119	53689	S234911	closed volar barton's fracture-dislocation	
Aurum and GOLD	208324004	318854014	561311000006117	107741	S234A12	closed dorsal barton fracture-subluxation	
Aurum and GOLD	208324004	318855010	561321000006113	57736	S234A11	closed dorsal barton's fracture-dislocation	
Aurum and GOLD	208324004	318856011	559791000006116	50053	S234A00	closd dorsal barton's fracture	
Aurum and GOLD	208325003	318857019	318857019	6915	S234B00	closed fracture radial styloid	
Aurum and GOLD	208326002	318858012	318858012	44844	S234C00	closed fracture distal radius, intra-articular, die-punch	
Aurum and GOLD	208336005	318875018	318875018	11262	S235300	open fracture of ulna, styloid process	
Aurum and GOLD	208337001	318876017	318876017	96691	S235400	open fracture of ulna, lower epiphysis	
Aurum and GOLD	208340001	318880010	318880010	98681	S235111	smith's fracture - open	
Aurum and GOLD	208340001	318880010	52291000006115	34730	S235700	open smith's fracture	
Aurum and GOLD	208341002	318881014	318881014	30418	S235800	open galeazzi fracture	
Aurum and GOLD	208342009	318882019	318882019	61675	S235900	open volar barton's fracture	
Aurum and GOLD	208343004	318887013	318887013	111858	S235A00	open dorsal barton's fracture	
Aurum and GOLD	208344005	318888015	318888015	6380	S235B00	open fracture radial styloid	
Aurum and GOLD	208345006	318889011	318889011	104070	S235C00	open fracture distal radius, intra-articular, die-punch	
Aurum and GOLD	208369005	318917016	318917016	39458	S240900	closed fracture hamate, hook	
Aurum and GOLD	208370006	318918014	318918014	16985	S240A00	closed fracture scaphoid, proximal pole	
Aurum and GOLD	208371005	318919018	318919018	19403	S240B00	closed fracture scaphoid, waist, transverse	
Aurum and GOLD	208372003	318920012	318920012	47837	S240C00	closed fracture scaphoid, waist, oblique	
Aurum and GOLD	208373008	318921011	318921011	44712	S240D00	closed fracture scaphoid, waist, comminuted	
Aurum and GOLD	208374002	318922016	318922016	28425	S240E00	closed fracture scaphoid, tuberosity	
Aurum and GOLD	208375001	318923014	318923014	73165	S240F00	closed fracture carpal bones, multiple	

Aurum and GOLD	208379007	318927010	318927010	110906	S241900	open fracture hamate, hook	
Aurum and GOLD	208380005	318928017	318928017	55814	S241A00	open fracture scaphoid, proximal pole	
Aurum and GOLD	208381009	318929013	318929013	73824	S241B00	open fracture scaphoid, waist, transverse	
Aurum and GOLD	208382002	318930015	318930015	59985	S241C00	open fracture scaphoid, waist, oblique	
Aurum and GOLD	208383007	318931016	318931016	64575	S241D00	open fracture scaphoid, waist, comminuted	
Aurum and GOLD	208384001	318932011	318932011	49588	S241E00	open fracture scaphoid, tuberosity	
Aurum and GOLD	208388003	1489807015	387591000006110	53068	Syu6500	[x]fracture of other & unspecified parts of wrist and hand	
Aurum and GOLD	208388003	318936014	318936014	8056	S242.00	fracture at wrist and hand level	
Aurum and GOLD	208390002	318938010	318938010	7564	S242100	fracture of first metacarpal bone	
Aurum and GOLD	208393000	318941018	318939019	993	S242200	fracture of other metacarpal bone	
Aurum and GOLD	208393000	318941018	318941018	2888	S25..00	fracture of metacarpal bone	
Aurum and GOLD	208393000	318941018	325418019	71739	Syu6400	[x]fracture of other metacarpal bone	
Aurum and GOLD	208393000	318943015	318943015	2643	S25..11	hand fracture - metacarpal bone	
Aurum and GOLD	208394006	2837251018	318945010	31525	S250000	closed fracture of metacarpal bone (s), site unspecified	
Aurum and GOLD	208394006	2837251018	318961012	21175	S250x00	closed fractures of multiple sites of unspecified metacarpus	
Aurum and GOLD	208394006	2837251018	318962017	6881	S250z00	closed fracture of metacarpal bone(s) nos	
Aurum and GOLD	208394006	318944014	318944014	6392	S250.00	closed fracture of metacarpal bone(s)	
Aurum and GOLD	208396008	318946011	570581000006118	10428	S250100	cls # thumb metacarpal base, intra-articular, bennett	
Aurum and GOLD	208397004	318947019	318947019	33905	S250200	closed fracture finger metacarpal base	
Aurum and GOLD	208399001	318949016	318949016	12546	S250400	closed fracture finger metacarpal neck	
Aurum and GOLD	208400008	318950016	318950016	24598	S250500	closed fracture finger metacarpal head	
Aurum and GOLD	208401007	318951017	318951017	27881	S250600	closed fracture finger metacarpal	
Aurum and GOLD	208402000	318952012	318952012	52895	S250700	closed fracture finger metacarpal, multiple	
Aurum and GOLD	208403005	318953019	318953019	25811	S250800	closed fracture of thumb metacarpal	
Aurum and GOLD	208404004	318954013	570591000006115	34054	S250900	cls # thumb metacarpal base, intra-articular, rolando	
Aurum and GOLD	208405003	318955014	318955014	50634	S250A00	closed fracture thumb metacarpal shaft	

Aurum and GOLD	208406002	318956010	318956010	49598	S250B00	closed fracture thumb metacarpal neck	
Aurum and GOLD	208408001	318959015	318959015	53650	S250C00	closed fracture thumb metacarpal head	
Aurum and GOLD	208413002	318965015	44351000006113	23983	S251100	opn # thumb metacarpal base, intra-articular, bennett	
Aurum and GOLD	208414008	318966019	318966019	73150	S251200	open fracture finger metacarpal base	
Aurum and GOLD	208416005	318968018	318968018	52588	S251400	open fracture finger metacarpal neck	
Aurum and GOLD	208417001	318969014	318969014	53693	S251500	open fracture finger metacarpal head	
Aurum and GOLD	208418006	318970010	318970010	40361	S251600	open fracture finger metacarpal	
Aurum and GOLD	208419003	318971014	318971014	72407	S251700	open fracture finger metacarpal, multiple	
Aurum and GOLD	208420009	318972019	318972019	60765	S251800	open fracture of thumb metacarpal	
Aurum and GOLD	208421008	318973012	44361000006110	56906	S251900	opn # thumb metacarpal base, intra-articular, rolando	
Aurum and GOLD	208422001	318974018	318974018	98867	S251A00	open fracture thumb metacarpal shaft	
Aurum and GOLD	208423006	318975017	318975017	112925	S251B00	open fracture thumb metacarpal neck	
Aurum and GOLD	208424000	318976016	318976016	102225	S251C00	open fracture thumb metacarpal head	
Aurum and GOLD	208427007	318979011	318979011	7531	S252.00	closed fracture sesamoid bone of hand	
Aurum and GOLD	208428002	318980014	318980014	52067	S253.00	open fracture sesamoid bone of hand	
Aurum and GOLD	208430000	318985016	570521000006117	25620	S260200	cls # distal phalanx or phalanges, unspecified part	
Aurum and GOLD	208430000	318985016	319017010	40535	S260z00	closed fracture of one or more phalanges of hand nos	
Aurum and GOLD	208430000	318985016	318985016	8302	S260.00	closed fracture of one or more phalanges of hand	
Aurum and GOLD	208430000	318985016	318986015	34058	S260000	closed fracture of phalanx or phalanges, unspecified	
Aurum and GOLD	208434009	318989010	318989010	33582	S260300	closed fracture thumb proximal phalanx	
Aurum and GOLD	208435005	318990018	318990018	33678	S260400	closed fracture thumb proximal phalanx, base	
Aurum and GOLD	208436006	318991019	318991019	43681	S260500	closed fracture thumb proximal phalanx, shaft	
Aurum and GOLD	208437002	318992014	318992014	64027	S260600	closed fracture thumb proximal phalanx, neck	
Aurum and GOLD	208438007	318993016	318993016	33651	S260700	closed fracture thumb proximal phalanx, head	
Aurum and GOLD	208439004	318994010	318994010	28249	S260800	closed fracture thumb distal phalanx	
Aurum and GOLD	208440002	318995011	318995011	34080	S260900	closed fracture thumb distal phalanx, base	

Aurum and GOLD	208441003	318996012	318996012	62808	S260A00	closed fracture thumb distal phalanx, shaft	
Aurum and GOLD	208442005	318997015	318997015	42139	S260B00	closed fracture thumb distal phalanx, tuft	
Aurum and GOLD	208443000	318998013	318998013	61181	S260C00	closed fracture thumb distal phalanx, mallet	
Aurum and GOLD	208444006	318999017	318999017	24516	S260D00	closed fracture finger proximal phalanx	
Aurum and GOLD	208445007	319000015	319000015	27699	S260E00	closed fracture finger proximal phalanx, base	
Aurum and GOLD	208446008	319001016	319001016	18338	S260F00	closed fracture finger proximal phalanx, shaft	
Aurum and GOLD	208447004	319002011	319002011	33679	S260G00	closed fracture finger proximal phalanx, neck	
Aurum and GOLD	208448009	319003018	319003018	44943	S260H00	closed fracture finger proximal phalanx, head	
Aurum and GOLD	208449001	319004012	319004012	44737	S260J00	closed fracture finger proximal phalanx, multiple	
Aurum and GOLD	208450001	319005013	319005013	33616	S260K00	closed fracture finger middle phalanx	
Aurum and GOLD	208451002	319006014	319006014	33598	S260L00	closed fracture finger middle phalanx, base	
Aurum and GOLD	208452009	319007017	319007017	33757	S260M00	closed fracture finger middle phalanx, shaft	
Aurum and GOLD	208453004	319008010	319008010	34356	S260N00	closed fracture finger middle phalanx, neck	
Aurum and GOLD	208454005	319009019	319009019	34307	S260P00	closed fracture finger middle phalanx, head	
Aurum and GOLD	208455006	319010012	319010012	67097	S260Q00	closed fracture finger middle phalanx, multiple	
Aurum and GOLD	208456007	319011011	319011011	27643	S260S00	closed fracture finger distal phalanx, base	
Aurum and GOLD	208457003	319012016	319012016	33695	S260T00	closed fracture finger distal phalanx, shaft	
Aurum and GOLD	208458008	319013014	319013014	33845	S260U00	closed fracture finger distal phalanx, tuft	
Aurum and GOLD	208459000	319014015	319014015	52398	S260V00	closed fracture finger distal phalanx, mallet	
Aurum and GOLD	208460005	319015019	319015019	45094	S260W00	closed fracture finger distal phalanx, multiple	
Aurum and GOLD	208461009	319016018	319016018	45374	S260x00	closed fractures of phalanx or phalanges, multiple sites	
Aurum and GOLD	208463007	319018017	319050010	50781	S261z00	open fracture of one or more phalanges of hand nos	
Aurum and GOLD	208463007	319018017	44321000006116	34099	S261200	opn # distal phalanx or phalanges, unspecified part	

Aurum and GOLD	208463007	319018017	319018017	18336	S261.00	open fracture of one or more phalanges of hand	
Aurum and GOLD	208463007	319018017	319019013	36556	S261000	open fracture of phalanx or phalanges, unspecified	
Aurum and GOLD	208463007	319018017	44331000006118	63961	S261100	opn # mid/prox phalanx or phalanges, unspecified part	
Aurum and GOLD	208467008	319022010	319022010	61279	S261300	open fracture thumb proximal phalanx	
Aurum and GOLD	208468003	319023017	319023017	94031	S261400	open fracture thumb proximal phalanx, base	
Aurum and GOLD	208469006	319024011	319024011	94416	S261500	open fracture thumb proximal phalanx, shaft	
Aurum and GOLD	208470007	319025012	319025012	99397	S261600	open fracture thumb proximal phalanx, neck	
Aurum and GOLD	208471006	319026013	319026013	101316	S261700	open fracture thumb proximal phalanx, head	
Aurum and GOLD	208472004	319027016	319027016	37986	S261800	open fracture thumb distal phalanx	
Aurum and GOLD	208473009	319028014	319028014	65731	S261900	open fracture thumb distal phalanx, base	
Aurum and GOLD	208474003	319029018	319029018	65715	S261A00	open fracture thumb distal phalanx, shaft	
Aurum and GOLD	208475002	319030011	319030011	53593	S261B00	open fracture thumb distal phalanx, tuft	
Aurum and GOLD	208476001	319031010	319031010	103416	S261C00	open fracture thumb distal phalanx, mallet	
Aurum and GOLD	208477005	319032015	319032015	33684	S261D00	open fracture finger proximal phalanx	
Aurum and GOLD	208478000	319033013	319033013	44700	S261E00	open fracture finger proximal phalanx, base	
Aurum and GOLD	208479008	319034019	319034019	62334	S261F00	open fracture finger proximal phalanx, shaft	
Aurum and GOLD	208480006	319035018	319035018	64113	S261G00	open fracture finger proximal phalanx, neck	
Aurum and GOLD	208481005	319036017	319036017	61529	S261H00	open fracture finger proximal phalanx, head	
Aurum and GOLD	208482003	319037014	319037014	69729	S261J00	open fracture finger proximal phalanx, multiple	
Aurum and GOLD	208483008	319038016	319038016	40304	S261K00	open fracture finger middle phalanx	
Aurum and GOLD	208484002	319039012	319039012	67718	S261L00	open fracture finger middle phalanx, base	
Aurum and GOLD	208485001	319040014	319040014	33866	S261M00	open fracture finger middle phalanx, shaft	
Aurum and GOLD	208486000	319041013	319041013	69363	S261N00	open fracture finger middle phalanx, neck	

Aurum and GOLD	208487009	319042018	319042018	99459	S261P00	open fracture finger middle phalanx, head	
Aurum and GOLD	208488004	319043011	319043011	112876	S261Q00	open fracture finger middle phalanx, multiple	
Aurum and GOLD	208489007	319044017	319044017	48837	S261S00	open fracture finger distal phalanx, base	
Aurum and GOLD	208490003	319045016	319045016	52333	S261T00	open fracture finger distal phalanx, shaft	
Aurum and GOLD	208491004	319046015	319046015	51700	S261U00	open fracture finger distal phalanx, tuft	
Aurum and GOLD	208492006	319047012	319047012	102046	S261V00	open fracture finger distal phalanx, mallet	
Aurum and GOLD	208493001	319048019	319048019	54123	S261W00	open fracture finger distal phalanx, multiple	
Aurum and GOLD	208494007	319049010	319049010	67011	S261x00	open fracture of phalanx or phalanges, multiple sites	
Aurum and GOLD	208499002	319054018	319054018	44431	S270.00	closed multiple fractures of hand bones	
Aurum and GOLD	208500006	319055017	319055017	33990	S271.00	open multiple fractures of hand bones	
Aurum and GOLD	208502003	319057013	319057013	49267	S28..00	ill-defined fractures of upper limb	
Aurum and GOLD	208502003	319057013	319063016	2660	S28z.00	ill-defined fractures of upper limb nos	
Aurum and GOLD	208502003	319058015	319058015	1179	S28..11	ill-defined fracture of arm	
Aurum and GOLD	208503008	319059011	319059011	47478	S280.00	closed ill-defined fractures of upper limb	
Aurum and GOLD	208505001	319062014	319062014	66853	S281.00	open ill-defined fractures of upper limb	
Aurum and GOLD	208508004	319069017	566791000006117	65674	S290.00	closed multiple #upper limbs & upper limb with rib + sternum	
Aurum and GOLD	208509007	319070016	254521000006118	111584	S291.00	open multiple #upper limbs & upper limb with rib + sternum	
Aurum and GOLD	208510002	319071017	399091000006110	97352	Syu4200	[x]multiple fractures of clavicle, scapula and humerus	
Aurum and GOLD	208510002	319071017	319071017	16944	S292.00	multiple fractures of clavicle, scapula and humerus	
Aurum and GOLD	208513000	319074013	319074013	8915	S293.00	multiple fractures of forearm	
Aurum and GOLD	208514006	319075014	319075014	27605	S294.00	fractures involving multiple regions of both upper limbs	
Aurum and GOLD	208521006	319084014	564491000006115	62966	S300z00	closed fracture proximal femur, transcervical, nos	
Aurum and GOLD	208521006	319084014	402869013	49209	S300y00	closed fracture proximal femur, other transcervical	
Aurum and GOLD	208521006	319084014	319084014	38489	S300.00	closed fracture proximal femur, transcervical	

Aurum and GOLD	208523009	319086011	319086011	69919	S300100	closed fracture proximal femur, transepiphyseal	
Aurum and GOLD	208524003	319087019	319087019	65690	S300200	closed fracture proximal femur, midcervical section	
Aurum and GOLD	208526001	319090013	319090013	36391	S300400	closed fracture head of femur	
Aurum and GOLD	208528000	319092017	319092017	34351	S300600	closed fracture proximal femur, subcapital, garden grade i	
Aurum and GOLD	208529008	319093010	319093010	33957	S300700	closed fracture proximal femur, subcapital, garden grade ii	
Aurum and GOLD	208530003	319094016	319094016	36599	S300800	closed fracture proximal femur, subcapital, garden grade iii	
Aurum and GOLD	208531004	319095015	319095015	34078	S300900	closed fracture proximal femur, subcapital, garden grade iv	
Aurum and GOLD	208536009	319102014	319102014	72138	S301100	open fracture proximal femur, transepiphyseal	
Aurum and GOLD	208537000	319103016	319103016	112836	S301200	open fracture proximal femur, midcervical section	
Aurum and GOLD	208540000	319108013	319108013	38878	S301500	open fracture proximal femur,subcapital, garden grade unspec	
Aurum and GOLD	208541001	319109017	319109017	60885	S301600	open fracture proximal femur,subcapital, garden grade i	
Aurum and GOLD	208542008	319110010	319110010	67394	S301700	open fracture proximal femur,subcapital, garden grade ii	
Aurum and GOLD	208543003	319111014	319111014	23803	S301800	open fracture proximal femur,subcapital, garden grade iii	
Aurum and GOLD	208544009	319112019	319112019	51999	S301900	open fracture proximal femur,subcapital, garden grade iv	
Aurum and GOLD	208548007	319118015	570571000006116	19117	S302000	cls # proximal femur, trochanteric section, unspecified	
Aurum and GOLD	208548007	319118015	319118015	5301	S302.00	closed fracture of proximal femur, pertrochanteric	
Aurum and GOLD	208548007	319118015	570531000006119	44735	S302z00	cls # of proximal femur, pertrochanteric section, nos	
Aurum and GOLD	208550004	319123015	319123015	45141	S302100	closed fracture proximal femur, intertrochanteric, two part	

Aurum and GOLD	208551000	319124014	319124014	29145	S302200	closed fracture proximal femur, subtrochanteric	
Aurum and GOLD	208552007	319125010	570561000006111	51216	S302300	cls # proximal femur, intertrochanteric, comminuted	
Aurum and GOLD	208555009	319128012	258191000006119	70479	S303z00	open fracture of proximal femur, pertrochanteric, nos	
Aurum and GOLD	208555009	319128012	319128012	61733	S303.00	open fracture of proximal femur, pertrochanteric	
Aurum and GOLD	208557001	319133011	319133011	101567	S303100	open fracture proximal femur, intertrochanteric, two part	
Aurum and GOLD	208558006	319134017	319134017	71282	S303200	open fracture proximal femur, subtrochanteric	
Aurum and GOLD	208559003	319135016	319135016	97971	S303300	open fracture proximal femur, intertrochanteric, comminuted	
Aurum and GOLD	208562000	319138019	319138019	28965	S304.00	pertrochanteric fracture	
Aurum and GOLD	208578003	319161017	319161017	21922	S312200	closed fracture of femur, lower epiphysis	
Aurum and GOLD	208579006	319162012	319162012	45562	S312400	closed fracture distal femur, medial condyle	
Aurum and GOLD	208580009	319163019	319163019	38355	S312500	closed fracture distal femur, lateral condyle	
Aurum and GOLD	208581008	319164013	319164013	54242	S312600	closed fracture distal femur, bicondylar (t-y fracture)	
Aurum and GOLD	208582001	319165014	319165014	55327	S312x00	closed fracture distal femur, comminuted/intra-articular	
Aurum and GOLD	208587007	319172010	319172010	48142	S313200	open fracture of femur, lower epiphysis	
Aurum and GOLD	208588002	319173017	319173017	67294	S313400	open fracture distal femur, medial condyle	
Aurum and GOLD	208589005	319174011	319174011	34738	S313500	open fracture distal femur, lateral condyle	
Aurum and GOLD	208591002	319176013	319176013	73208	S313x00	open fracture distal femur, comminuted/intra-articular	
Aurum and GOLD	208596007	319181016	319181016	49526	S320000	closed fracture patella, transverse	
Aurum and GOLD	208597003	319182011	319182011	50549	S320100	closed fracture patella, proximal pole	
Aurum and GOLD	208598008	319183018	319183018	44329	S320200	closed fracture patella, distal pole	
Aurum and GOLD	208600002	319186014	319186014	54660	S320300	closed fracture patella, vertical	
Aurum and GOLD	208601003	319188010	319188010	41287	S320400	closed fracture patella, comminuted (stellate)	
Aurum and GOLD	208602005	319189019	319189019	50227	S321000	open fracture patella, transverse	

Aurum and GOLD	208603000	319190011	319190011	100159	S321100	open fracture patella, proximal pole	
Aurum and GOLD	208604006	319191010	319191010	33475	S321200	open fracture patella, distal pole	
Aurum and GOLD	208606008	319193013	319193013	50254	S321400	open fracture patella, comminuted (stellate)	
Aurum and GOLD	208610006	319200010	319200010	18840	S330300	closed fracture proximal tibia, medial condyle (plateau)	
Aurum and GOLD	208611005	319201014	319201014	22370	S330400	closed fracture proximal tibia, lateral condyle (plateau)	
Aurum and GOLD	208612003	319202019	319202019	40164	S330500	closed fracture proximal tibia, bicondylar	
Aurum and GOLD	208613008	319203012	319203012	33768	S330600	closed fracture spine, tibia	
Aurum and GOLD	208614002	319204018	319204018	38733	S330700	closed fracture tubercle, tibia	
Aurum and GOLD	208615001	319205017	319205017	52322	S330900	closed fracture fibula, neck	
Aurum and GOLD	208619007	319212014	319212014	44276	S331300	open fracture proximal tibia, medial condyle (plateau)	
Aurum and GOLD	208620001	319213016	319213016	44786	S331400	open fracture proximal tibia, lateral condyle (plateau)	
Aurum and GOLD	208621002	319214010	319214010	112873	S331500	open fracture proximal tibia, bicondylar	
Aurum and GOLD	208622009	319215011	319215011	63633	S331600	open fracture spine, tibia	
Aurum and GOLD	208623004	319216012	319216012	49798	S331700	open fracture tubercle, tibia	
Aurum and GOLD	208624005	319217015	319217015	99027	S331800	open fracture fibula, head	
Aurum and GOLD	208625006	319218013	319218013	99161	S331900	open fracture fibula, neck	
Aurum and GOLD	208627003	319220011	564101000006116	29121	S332.00	closed fracture of tibia/fibula, shaft	
Aurum and GOLD	208629000	319222015	319222015	33520	S332200	closed fracture of tibia and fibula, shaft	
Aurum and GOLD	208629000	319222015	319223013	55464	S332z00	closed fracture of tibia and fibula, shaft, nos	
Aurum and GOLD	208631009	319224019	257211000006119	28068	S333.00	open fracture of tibia/fibula, shaft	
Aurum and GOLD	208632002	319225018	319225018	20678	S333200	open fracture of tibia and fibula, shaft	
Aurum and GOLD	208632002	319225018	319226017	28198	S333z00	open fracture of tibia and fibula, shaft, nos	
Aurum and GOLD	208634001	319227014	319227014	27719	S334.00	closed fracture distal tibia	
Aurum and GOLD	208635000	319228016	319228016	34151	S334000	closed fracture distal tibia, extra-articular	
Aurum and GOLD	208636004	319229012	319229012	8465	S334100	closed fracture distal tibia, intra-articular	
Aurum and GOLD	208637008	319230019	319230019	27992	S335.00	open fracture distal tibia	
Aurum and GOLD	208638003	319231015	319231015	27721	S335000	open fracture distal tibia, extra-articular	

Aurum and GOLD	208639006	319232010	319232010	65228	S335100	open fracture distal tibia, intra-articular	
Aurum and GOLD	208657007	319252011	319252011	7135	S342000	closed fracture ankle, lateral malleolus, low	
Aurum and GOLD	208658002	319253018	319253018	35620	S342100	closed fracture ankle, lateral malleolus, high	
Aurum and GOLD	208659005	319254012	319254012	43566	S343000	open fracture ankle, lateral malleolus, low	
Aurum and GOLD	208660000	319255013	319255013	73105	S343100	open fracture ankle, lateral malleolus, high	
Aurum and GOLD	208662008	319261011	319261011	42969	S344000	closed fracture ankle, bimalleolar, low fibular fracture	
Aurum and GOLD	208663003	319262016	319262016	52371	S344100	closed fracture ankle, bimalleolar, high fibular fracture	
Aurum and GOLD	208664009	319263014	319263014	66808	S345000	open fracture ankle, bimalleolar, low fibular fracture	
Aurum and GOLD	208665005	319264015	319264015	105816	S345100	open fracture ankle, bimalleolar, high fibular fracture	
Aurum and GOLD	208666006	319265019	319265019	56525	S346000	closed fracture ankle, trimalleolar, low fibular fracture	
Aurum and GOLD	208667002	319266018	319266018	52346	S346100	closed fracture ankle, trimalleolar, high fibular fracture	
Aurum and GOLD	208668007	319267010	319267010	47828	S347000	open fracture ankle, trimalleolar, low fibular fracture	
Aurum and GOLD	208669004	319268017	319268017	105819	S347100	open fracture ankle, trimalleolar, high fibular fracture	
Aurum and GOLD	208677000	319283010	319283010	57924	S350000	closed fracture calcaneus, extra-articular	
Aurum and GOLD	208678005	319284016	319284016	34723	S350100	closed fracture calcaneus, intra-articular	
Aurum and GOLD	208680004	319286019	319286019	70226	S351100	open fractures calcaneus, intra-articular	
Aurum and GOLD	208684008	319293015	319293015	37450	S352800	closed fracture talus, head	
Aurum and GOLD	208685009	319294014	319294014	46955	S352900	closed fracture talus, neck	
Aurum and GOLD	208686005	319295010	319295010	45664	S352A00	closed fracture talus, body	
Aurum and GOLD	208687001	319296011	319296011	24620	S352B00	closed fracture metatarsal base	
Aurum and GOLD	208688006	319297019	319297019	27567	S352C00	closed fracture metatarsal shaft	
Aurum and GOLD	208689003	319298012	319298012	29748	S352D00	closed fracture metatarsal neck	
Aurum and GOLD	208690007	319299016	319299016	35077	S352E00	closed fracture metatarsal head	
Aurum and GOLD	208691006	319300012	319300012	28371	S352F00	closed fracture metatarsal, multiple	
Aurum and GOLD	208692004	319301011	319301011	64378	S352G00	closed tarsal fractures, multiple	

Aurum and GOLD	208696001	319305019	319305019	109115	S353800	open fracture talus, head	
Aurum and GOLD	208697005	319306018	319306018	93536	S353900	open fracture talus, neck	
Aurum and GOLD	208698000	319307010	319307010	97386	S353A00	open fracture talus, body	
Aurum and GOLD	208699008	319308017	319308017	72586	S353B00	open fracture metatarsal base	
Aurum and GOLD	208700009	319309013	319309013	64545	S353C00	open fracture metatarsal shaft	
Aurum and GOLD	208701008	319310015	319310015	97803	S353D00	open fracture metatarsal neck	
Aurum and GOLD	208702001	319311016	319311016	105612	S353E00	open fracture metatarsal head	
Aurum and GOLD	208703006	319312011	319312011	66099	S353F00	open fracture metatarsal, multiple	
Aurum and GOLD	208704000	319313018	319313018	113126	S353G00	open tarsal fractures, multiple	
Aurum and GOLD	208710000	319319019	319319019	25073	S360000	closed fracture proximal phalanx, toe	
Aurum and GOLD	208711001	319320013	319320013	29804	S360100	closed fracture middle phalanx, toe	
Aurum and GOLD	208712008	319321012	319321012	28251	S360200	closed fracture distal phalanx, toe	
Aurum and GOLD	208713003	319322017	319322017	53905	S360300	closed fracture multiple phalanges, toe	
Aurum and GOLD	208715005	319324016	319324016	50517	S361000	open fracture proximal phalanx, toe	
Aurum and GOLD	208716006	319325015	319325015	69728	S361100	open fracture middle phalanx, toe	
Aurum and GOLD	208717002	319326019	319326019	51213	S361200	open fracture distal phalanx, toe	
Aurum and GOLD	208718007	319327011	319327011	61556	S361300	open fracture multiple phalanges, toe	
Aurum and GOLD	208719004	319328018	319328018	2176	S362.00	fracture of great toe	
Aurum and GOLD	208731002	319340018	319340018	21773	S3x2.00	multiple fractures of femur	
Aurum and GOLD	208732009	319341019	319341019	9348	S3x3.00	multiple fractures of lower leg	
Aurum and GOLD	208733004	319342014	319342014	9174	S3x4.00	multiple fractures of foot	
Aurum and GOLD	209236003	319932017	793931000006111	5345	S4A..00	fracture-dislocation or subluxation shoulder	
Aurum and GOLD	209238002	319934016	565391000006112	8348	S4A0.00	closed fracture-dislocation shoulder	
Aurum and GOLD	209238002	319934016	565401000006114	33704	S4A0000	closed fracture-dislocation shoulder joint	
Aurum and GOLD	209239005	319935015	565221000006115	27718	S4A0100	closed fracture-dislocation acromio-clavicular joint	
Aurum and GOLD	209242004	319939014	256011000006112	64229	S4A1.00	open fracture-dislocation shoulder	
Aurum and GOLD	209242004	319939014	256021000006116	102965	S4A1000	open fracture-dislocation shoulder joint	
Aurum and GOLD	209243009	319940011	256661000006116	50573	S4A1100	open fracture-dislocation acromio-clavicular joint	
Aurum and GOLD	209244003	319941010	565721000006114	35789	S4A2.00	closed fracture-subluxation shoulder	
Aurum and GOLD	209245002	319942015	565731000006112	60580	S4A2000	closed fracture-subluxation shoulder joint	

Aurum and GOLD	209246001	319943013	565561000006111	24534	S4A2100	closed fracture-subluxation acromio-clavicular joint	
Aurum and GOLD	209248000	319945018	255591000006115	111060	S4A3.00	open fracture-subluxation shoulder	
Aurum and GOLD	209249008	319946017	256221000006113	60828	S4A3100	open fracture-subluxation acromio-clavicular joint	
Aurum and GOLD	209250008	319947014	793881000006117	10228	S4B..00	fracture-dislocation or subluxation elbow	
Aurum and GOLD	209252000	319949012	565271000006119	9468	S4B0000	closed fracture-dislocation elbow joint	
Aurum and GOLD	209252000	319949012	565261000006114	33687	S4B0.00	closed fracture-dislocation elbow	
Aurum and GOLD	209253005	319950012	565411000006112	62960	S4B0100	closed fracture-dislocation superior radio-ulnar joint	
Aurum and GOLD	209255003	2475701014	256701000006112	43972	S4B1.00	open fracture-dislocation elbow	
Aurum and GOLD	209255003	319952016	256711000006110	54149	S4B1000	open fracture-dislocation elbow joint	
Aurum and GOLD	209256002	319953014	256051000006113	104355	S4B1100	open fracture-dislocation superior radio-ulnar joint	
Aurum and GOLD	209257006	319954015	565591000006115	58752	S4B2.00	closed fracture-subluxation elbow	
Aurum and GOLD	209258001	319955019	565601000006111	67036	S4B2000	closed fracture-subluxation elbow joint	
Aurum and GOLD	209259009	319956018	565741000006119	104356	S4B2100	closed fracture-subluxation superior radio-ulnar joint	
Aurum and GOLD	209261000	3034703012	256251000006116	85491	S4B3.00	open fracture-subluxation elbow	
Aurum and GOLD	209263002	319960015	793921000006113	18614	S4C..00	fracture-dislocation or subluxation of wrist	
Aurum and GOLD	209264008	319961016	565351000006118	112915	S4C0y00	closed fracture-dislocation other carpal	
Aurum and GOLD	209264008	319961016	319961016	34429	S4C0.00	closed fracture dislocation of wrist	
Aurum and GOLD	209265009	319962011	565251000006112	17922	S4C0000	closed fracture-dislocation distal radio-ulnar joint	
Aurum and GOLD	209266005	319963018	565381000006114	38408	S4C0100	closed fracture-dislocation radiocarpal joint	
Aurum and GOLD	209267001	319964012	565311000006119	67584	S4C0200	closed fracture-dislocation mid carpal	
Aurum and GOLD	209268006	319965013	565431000006118	27783	S4C0300	closed fracture-dislocation, carpometacarpal joint	
Aurum and GOLD	209269003	319966014	565301000006117	64435	S4C0400	closed fracture-dislocation lunate (volar)	
Aurum and GOLD	209270002	319967017	565361000006116	96136	S4C0500	closed fracture-dislocation peri-lunate (dorsal)	
Aurum and GOLD	209271003	319968010	565371000006111	55212	S4C0600	closed fracture-dislocation peri-lunate trans-scaphoid	
Aurum and GOLD	209273000	319970018	319970018	42844	S4C1.00	open fracture dislocation wrist	
Aurum and GOLD	209274006	319971019	256081000006117	9261	S4C1000	open fracture-dislocation, distal radio-ulnar joint	

Aurum and GOLD	209276008	319974010	256001000006114	49256	S4C1100	open fracture-dislocation radiocarpal joint	
Aurum and GOLD	209278009	319976012	256671000006111	97476	S4C1300	open fracture-dislocation carpometacarpal joint	
Aurum and GOLD	209281004	319979017	255991000006113	63292	S4C1600	open fracture-dislocation peri-lunate trans-scaphoid	
Aurum and GOLD	209283001	319981015	565671000006117	17921	S4C2.00	closed fracture-subluxation of the wrist	
Aurum and GOLD	209284007	319982010	565781000006113	44652	S4C2000	closed fracture-subluxation, distal radio-ulnar jt	
Aurum and GOLD	209285008	319983017	565711000006118	50148	S4C2100	closed fracture-subluxation radiocarpal joint	
Aurum and GOLD	209286009	319984011	565631000006115	103524	S4C2200	closed fracture-subluxation mid carpal	
Aurum and GOLD	209287000	319985012	565761000006115	68595	S4C2300	closed fracture-subluxation, carpometacarpal joint	
Aurum and GOLD	209288005	319986013	565621000006118	55412	S4C2400	closed fracture-subluxation lunate (volar)	
Aurum and GOLD	209290006	319988014	565701000006116	63712	S4C2600	closed fracture-subluxation peri-lunate trans-scaphoid	
Aurum and GOLD	209292003	319990010	256341000006113	59219	S4C3.00	open fracture-subluxation of the wrist	
Aurum and GOLD	209293008	319991014	255671000006117	60343	S4C3000	open fracture-subluxation, distal radio-ulnar joint	
Aurum and GOLD	209294002	319992019	255581000006118	46798	S4C3100	open fracture-subluxation radiocarpal joint	
Aurum and GOLD	209296000	319994018	255651000006110	94661	S4C3300	open fracture-subluxation, carpometacarpal joint	
Aurum and GOLD	209297009	319995017	256281000006112	113363	S4C3400	open fracture-subluxation lunate (volar)	
Aurum and GOLD	209299007	319997013	255571000006116	68262	S4C3600	open fracture-subluxation peri-lunate trans-scaphoid	
Aurum and GOLD	209301000	319999011	793941000006118	10250	S4D..00	fracture-dislocation/subluxation finger/thumb	
Aurum and GOLD	209302007	320000013	565291000006118	39708	S4D0200	closed fracture-dislocation ipj, unspecified	
Aurum and GOLD	209302007	320000013	565231000006117	35198	S4D0.00	closed fracture-dislocation digit	
Aurum and GOLD	209302007	320000013	565241000006110	42990	S4D0000	closed fracture-dislocation digit, unspecified	
Aurum and GOLD	209304008	320002017	565501000006110	25445	S4D0100	closed fracture-dislocation, metacarpophalangeal joint	
Aurum and GOLD	209306005	320004016	565441000006111	33985	S4D0300	closed fracture-dislocation, distal interphalangeal joint	

Aurum and GOLD	209307001	320005015	565531000006119	19375	S4D0400	closed fracture-dislocation, proximal interphalangeal joint	
Aurum and GOLD	209308006	320006019	565461000006110	10462	S4D0500	closed fracture-dislocation, interphalangeal joint thumb	
Aurum and GOLD	209309003	320007011	565321000006110	63049	S4D0600	closed fracture-dislocation multiple digits	
Aurum and GOLD	209310008	320008018	256681000006114	18841	S4D1.00	open fracture-dislocation digit	
Aurum and GOLD	209310008	320008018	256721000006119	94265	S4D1200	open fracture-dislocation ipj, unspecified	
Aurum and GOLD	209310008	320008018	256691000006112	65848	S4D1000	open fracture-dislocation digit, unspecified	
Aurum and GOLD	209312000	320010016	256151000006112	61858	S4D1100	open fracture-dislocation, metacarpophalangeal joint	
Aurum and GOLD	209314004	320012012	256071000006115	43423	S4D1300	open fracture-dislocation, distal interphalangeal joint	
Aurum and GOLD	209315003	320013019	256191000006118	37582	S4D1400	open fracture-dislocation, proximal interphalangeal joint	
Aurum and GOLD	209316002	320014013	256111000006111	45690	S4D1500	open fracture-dislocation, interphalangeal joint thumb	
Aurum and GOLD	209317006	320015014	256751000006111	96998	S4D1600	open fracture-dislocation multiple digits	
Aurum and GOLD	209318001	320016010	565571000006116	48874	S4D2.00	closed fracture-subluxation digit	
Aurum and GOLD	209318001	320016010	565581000006118	41558	S4D2000	closed fracture-subluxation digit, unspecified	
Aurum and GOLD	209320003	320018011	565851000006111	36527	S4D2100	closed fracture-subluxation, metacarpophalangeal joint	
Aurum and GOLD	209322006	320020014	565771000006110	55356	S4D2300	closed fracture-subluxation, distal interphalangeal joint	
Aurum and GOLD	209323001	320021013	565881000006115	40817	S4D2400	closed fracture-subluxation, proximal interphalangeal joint	
Aurum and GOLD	209324007	320022018	565811000006110	63071	S4D2500	closed fracture-subluxation, interphalangeal joint thumb	
Aurum and GOLD	209325008	320023011	565641000006113	104015	S4D2600	closed fracture-subluxation multiple digits	
Aurum and GOLD	209326009	320024017	256231000006111	73986	S4D3.00	open fracture-subluxation digit	
Aurum and GOLD	209328005	320026015	255741000006119	66544	S4D3100	open fracture-subluxation, metacarpophalangeal joint	

Aurum and GOLD	209330007	320028019	255661000006112	51946	S4D3300	open fracture-subluxation, distal interphalangeal joint	
Aurum and GOLD	209331006	320029010	255781000006113	65494	S4D3400	open fracture-subluxation, proximal interphalangeal joint	
Aurum and GOLD	209332004	320030017	255701000006116	92349	S4D3500	open fracture-subluxation, interphalangeal joint thumb	
Aurum and GOLD	209333009	320031018	256301000006111	96438	S4D3600	open fracture-subluxation multiple digits	
Aurum and GOLD	209334003	320032013	793901000006115	24587	S4E..00	fracture-dislocation or subluxation hip	
Aurum and GOLD	209335002	320033015	565451000006113	40267	S4E0.00	closed fracture-dislocation, hip joint	
Aurum and GOLD	209336001	320034014	256101000006113	58720	S4E1.00	open fracture-dislocation, hip joint	
Aurum and GOLD	209337005	320035010	565801000006112	93374	S4E2.00	closed fracture-subluxation, hip joint	
Aurum and GOLD	209339008	320037019	793911000006117	29911	S4F..00	fracture-dislocation or subluxation knee	
Aurum and GOLD	209340005	320038012	565491000006119	40653	S4F0.00	closed fracture-dislocation, knee joint	
Aurum and GOLD	209341009	320039016	256141000006110	28731	S4F1.00	open fracture-dislocation, knee joint	
Aurum and GOLD	209342002	320040019	565841000006114	33666	S4F2.00	closed fracture-subluxation, knee joint	
Aurum and GOLD	209343007	320041015	255731000006112	57196	S4F3.00	open fracture-subluxation, knee joint	
Aurum and GOLD	209344001	320042010	565521000006117	29981	S4F4.00	closed fracture-dislocation, patello-femoral joint	
Aurum and GOLD	209345000	320043017	256181000006116	60669	S4F5.00	open fracture-dislocation, patello-femoral joint	
Aurum and GOLD	209346004	320044011	565871000006118	40650	S4F6.00	closed fracture-subluxation, patello-femoral joint	
Aurum and GOLD	209347008	320045012	255771000006110	38943	S4F7.00	open fracture-subluxation, patello-femoral joint	
Aurum and GOLD	209348003	320046013	793871000006115	15491	S4G..00	fracture-dislocation or subluxation ankle	
Aurum and GOLD	209349006	320047016	565421000006116	34302	S4G0.00	closed fracture-dislocation, ankle joint	
Aurum and GOLD	209350006	320048014	256061000006110	56927	S4G1.00	open fracture-dislocation, ankle joint	
Aurum and GOLD	209351005	320049018	565751000006117	59411	S4G2.00	closed fracture-subluxation, ankle joint	
Aurum and GOLD	209352003	320050018	255641000006113	28070	S4G3.00	open fracture-subluxation, ankle joint	
Aurum and GOLD	209353008	320051019	793891000006119	17249	S4H..00	fracture-dislocation or subluxation foot	
Aurum and GOLD	209354002	320052014	565281000006116	44628	S4H0.00	closed fracture-dislocation foot	
Aurum and GOLD	209355001	320053016	565541000006112	56599	S4H0000	closed fracture-dislocation, subtalar joint	
Aurum and GOLD	209356000	320054010	565511000006113	71132	S4H0100	closed fracture-dislocation, midtarsal joint	

Aurum and GOLD	209357009	320055011	565551000006114	67849	S4H0200	closed fracture-dislocation, tarsometatarsal joint	
Aurum and GOLD	209358004	320056012	559901000006114	42200	S4H0300	closed #-dislocation, metatarsophalangeal joint, single	
Aurum and GOLD	209359007	320057015	565481000006117	28800	S4H0400	closed fracture-dislocation, ipj, single toe	
Aurum and GOLD	209360002	320058013	559891000006110	49894	S4H0500	closed #-dislocation, metatarsophalangeal joint, multiple	
Aurum and GOLD	209361003	320059017	565471000006115	92268	S4H0600	closed fracture-dislocation, ipj, multiple toes	
Aurum and GOLD	209362005	320060010	256091000006119	52340	S4H1.00	open fracture-dislocation, foot	
Aurum and GOLD	209363000	320061014	256201000006115	40445	S4H1000	open fracture-dislocation, subtalar joint	
Aurum and GOLD	209364006	320062019	256171000006119	108596	S4H1100	open fracture-dislocation, midtarsal joint	
Aurum and GOLD	209365007	320063012	256211000006117	92043	S4H1200	open fracture-dislocation, tarsometatarsal joint	
Aurum and GOLD	209366008	320064018	256161000006114	39893	S4H1300	open fracture-dislocation, metatarsophalangeal joint, single	
Aurum and GOLD	209367004	320065017	256131000006117	42902	S4H1400	open fracture-dislocation, ipj, single toe	
Aurum and GOLD	209368009	320066016	265431000006111	65688	S4H1500	open #-dislocation, metatarsophalangeal joint, multiple	
Aurum and GOLD	209369001	320067013	256121000006115	106875	S4H1600	open fracture-dislocation, ipj, multiple toes	
Aurum and GOLD	209370000	320068015	565791000006111	44673	S4H2.00	closed fracture-subluxation, foot	
Aurum and GOLD	209371001	320069011	565891000006117	72071	S4H2000	closed fracture-subluxation, subtalar joint	
Aurum and GOLD	209372008	320070012	565861000006113	65028	S4H2100	closed fracture-subluxation, midtarsal joint	
Aurum and GOLD	209373003	320071011	565901000006118	73703	S4H2200	closed fracture-subluxation, tarsometatarsal joint	
Aurum and GOLD	209374009	320072016	559941000006111	17611	S4H2300	closed #-subluxation, metatarsophalangeal joint, single	
Aurum and GOLD	209375005	320073014	565831000006116	95728	S4H2400	closed fracture-subluxation, ipj, single toe	
Aurum and GOLD	209377002	320076018	559931000006118	57907	S4H2500	closed #-subluxation, metatarsophalangeal joint, multiple	
Aurum and GOLD	209378007	320079013	565821000006119	72822	S4H2600	closed fracture-subluxation, ipj, multiple toes	
Aurum and GOLD	209379004	320080011	255681000006119	93650	S4H3.00	open fracture-subluxation, foot	

Aurum and GOLD	209382009	320083013	255801000006112	112577	S4H3200	open fracture-subluxation, tarsometatarsal joint	
Aurum and GOLD	209383004	320084019	255751000006117	65895	S4H3300	open fracture-subluxation, metatarsophalangeal joint, single	
Aurum and GOLD	209384005	320085018	255721000006114	68514	S4H3400	open fracture-subluxation, ipj, single toe	
Aurum and GOLD	209389000	320090015	565341000006115	65155	S4J0000	closed fracture-dislocation of sternum	
Aurum and GOLD	209390009	320091016	565331000006113	34910	S4J0100	closed fracture-dislocation of pelvis	
Aurum and GOLD	209391008	320092011	559871000006114	50309	S4J0200	closed #-dislocation sterno-clavicular joint, anterior	
Aurum and GOLD	209392001	320093018	559881000006112	93679	S4J0300	closed #-dislocation sterno-clavicular joint, posterior	
Aurum and GOLD	209394000	320095013	255961000006117	94236	S4J1000	open fracture-dislocation of sternum	
Aurum and GOLD	209395004	320096014	256761000006113	45011	S4J1100	open fracture-dislocation of pelvis	
Aurum and GOLD	209396003	320097017	256031000006118	24615	S4J1200	open fracture-dislocation sterno-clavicular joint, anterior	
Aurum and GOLD	209397007	320098010	256041000006111	112863	S4J1300	open fracture-dislocation sterno-clavicular joint, posterior	
Aurum and GOLD	209399005	320100010	565661000006112	62631	S4J2000	closed fracture-subluxation of sternum	
Aurum and GOLD	209400003	320101014	565651000006110	34212	S4J2100	closed fracture-subluxation of pelvis	
Aurum and GOLD	209401004	320102019	559911000006112	62484	S4J2200	closed #-subluxation sterno-clavicular joint, anterior	
Aurum and GOLD	209404007	320105017	256321000006118	94593	S4J3000	open fracture-subluxation of sternum	
Aurum and GOLD	209405008	320106016	256311000006114	65297	S4J3100	open fracture-subluxation of pelvis	
Aurum and GOLD	213085001	324880017	324880017	62649	SP04C00	fracture of bone allograft	
Aurum and GOLD	213345000	325242018	325485012	110205	SyuA200	[x]fractures involving other combinations of body regions	
Aurum and GOLD	213346004	325243011	325243011	19206	SR1..00	fractures involving multiple body regions	
Aurum and GOLD	213347008	325244017	325244017	32009	SR10.00	fractures involving head with neck	
Aurum and GOLD	213350006	325247012	325247012	45934	SR11.00	fractures involving thorax with lower back and pelvis	
Aurum and GOLD	213350006	325247012	387541000006118	53802	Syu3400	[x]fract of other and unspec parts of lumbar spine & pelvis	
Aurum and GOLD	213351005	325248019	325248019	53423	SR12.00	fractures involving multiple regions of one upper limb	
Aurum and GOLD	213354002	325251014	325251014	69824	SR13.00	fractures involving multiple regions of one lower limb	

Aurum and GOLD	213355001	325252019	325252019	99303	SR14.00	fractures involving multiple regions of both lower limbs	
Aurum and GOLD	213358004	325255017	792401000006116	56858	SR15.00	fract invol multiple regions of up limb(s) with low limb(s)	
Aurum and GOLD	213361003	325258015	792411000006118	28110	SR16.00	fract invol thorax with lower back and pelvis with limb(s)	
Aurum and GOLD	21351003	1222629012	1222629012	1873	S36..11	toe fracture	
Aurum and GOLD	21351003	2956777012	793141000006115	2672	S36..00	fracture of one or more phalanges of foot	
Aurum and GOLD	21351003	35860018	319332012	34424	S36z.00	fracture of one or more phalanges of foot nos	
Aurum and GOLD	21351003	35863016	319331017	7159	S363.00	fracture of other toe	
Aurum and GOLD	21419000	35953013	1222634011	28724	S224700	closed fracture distal humerus, medial epicondyle	
Aurum and GOLD	21419000	35953013	318785014	40367	S224300	closed fracture distal humerus, medial condyle	
Aurum and GOLD	21573009	36188017	531701000006114	38053	S100711	c7 vertebra closed fracture without spinal cord lesion	
Aurum and GOLD	22225000	37308015	319339015	69917	S3x1.00	other, multiple and ill-defined open fractures of lower limb	
Aurum and GOLD	22234005	1222720019	1222720019	28118	S333000	open fracture shaft of tibia	
Aurum and GOLD	22709009	481406012	481406012	70086	7433300	reduction of laryngeal fracture	
Aurum and GOLD	22713002	38124014	570931000006114	34931	S260100	clsd # mid/prox phalanx/phalanges, unspecified part	
Aurum and GOLD	2295008	4947010	318814012	33933	S230000	closed fracture of proximal forearm, unspecified part	
Aurum and GOLD	23020007	1223016015	1223016015	97211	S353500	open fracture intermediate cuneiform	
Aurum and GOLD	230411000000106	370001000000111	370001000000111	88269	7J41300	vertebroplasty of fracture of spine	
Aurum and GOLD	23382007	1223079017	1223079017	69786	Zw02400	[q] stress fracture	
Aurum and GOLD	23382007	39246018	39246018	3025	S3z2.00	stress fracture	
Aurum and GOLD	23406007	1223089018	1223089018	5929	S2...11	arm fracture	
Aurum and GOLD	23406007	39287017	319079015	33882	S2A..00	fracture of upper limb, level unspecified	
Aurum and GOLD	23406007	39287017	39287017	6195	S2...00	fracture of upper limb	
Aurum and GOLD	23406007	39287017	319080017	30076	S2z..00	fracture of upper limb nos	
Aurum and GOLD	23611004	1495084019	1495084019	36772	S023.11	fracture of lower jaw, open	
Aurum and GOLD	23611004	1495085018	1495085018	38050	S023.00	fracture of mandible, open	

Aurum and GOLD	23611004	39648010	318212019	70673	S023000	open fracture mandible (site unspecified)	
Aurum and GOLD	23611004	39648010	318222013	60260	S023800	open fracture of mandible, body, other and unspecified	
Aurum and GOLD	23611004	39648010	318224014	60239	S023z00	fracture of mandible, open, nos	
Aurum and GOLD	23611004	39648010	318216016	68660	S023400	open fracture of mandible, ramus, unspecified	
Aurum and GOLD	23741005	1223146015	1223146015	40358	S223100	open fracture of humerus, shaft	
Aurum and GOLD	23813004	1223154018	1223154018	63948	S231900	open fracture of the proximal radius	
Aurum and GOLD	23897002	40139014	40139014	10736	7206100	open reduction of fracture of orbit	
Aurum and GOLD	23900009	40149012	563891000006116	28550	S330000	closed fracture of the proximal tibia	
Aurum and GOLD	239278008	358592018	276092017	53575	7J02300	repair of fracture of cranium nec	
Aurum and GOLD	239281003	358595016	208591000006116	97337	7K1D013	prim op red # nck femur & op fix - deyerle multiple hip pin	
Aurum and GOLD	239284006	358598019	208601000006112	52395	7K1D012	prim op red # nck femur & op fix-charnley compression screw	
Aurum and GOLD	239286008	358601012	208731000006113	24493	7K1D01A	prim open reduct # neck femur & op fix - richards screw	
Aurum and GOLD	239288009	358603010	570611000006114	57514	7K1J013	cls red+int fxn prox femoral #+richard's cannulat hip screw	
Aurum and GOLD	239290005	358606019	557971000006111	40999	7K1J012	cl red intracaps fract neck femur fix - smith-petersen nail	
Aurum and GOLD	239291009	358607011	208681000006115	94714	7K1D014	prim open reduct # neck femur & op fix - holt nail	
Aurum and GOLD	239292002	358608018	208741000006115	57884	7K1D01B	prim open reduct # neck femur & op fix - ross brown nail	
Aurum and GOLD	239294001	358614013	180831000006119	38868	7K1K200	remanipulation of fracture of bone and external fixation hfq	History
Aurum and GOLD	239294001	358614013	151841000006110	68992	7K1H400	secondary open reduct fracture bone & external fixation hfq	
Aurum and GOLD	239295000	358615014	205131000006119	29455	7K1Gy00	primary open reduction of #+other ext immobilisation	
Aurum and GOLD	239548003	358967010	276559015	93752	7J42y00	other specified other reduction of fracture of spine	
Aurum and GOLD	239548003	358967010	276496014	98165	7J42B00	primary other external stabilisation of spinal fracture	

Aurum and GOLD	239548003	358967010	411017011	48159	7J42.11	other reduction of fracture of spine and stabilisation	
Aurum and GOLD	239548003	358967010	394586014	20744	7J42.00	other reduction of fracture of spine	
Aurum and GOLD	239548003	358967010	276560013	92356	7J42z00	other reduction of fracture of spine nos	
Aurum and GOLD	239550006	358969013	276485018	15622	7J42500	spinal traction for fracture of spine nec	
Aurum and GOLD	240198002	2920899019	455536018	4013	N331L00	collapse of vertebra due to osteoporosis nos	
Aurum and GOLD	240198002	359833013	359833013	44386	N331.14	osteoporotic vertebral collapse	
Aurum and GOLD	24063002	40395011	318196011	33593	S01z.00	fracture of base of skull nos	
Aurum and GOLD	24063002	40395011	560101000006111	58688	S010000	closed #skull bse no intracranial injury, unspec state consc	
Aurum and GOLD	24063002	40395011	259611000006111	69737	S012.00	open fracture base skull without mention intracranial injury	
Aurum and GOLD	24063002	40395011	264211000006117	100796	S012200	open #skull bse no intracranial injury, <1hr loss of consc	
Aurum and GOLD	24063002	481804018	40395011	2461	S01..00	fracture of base of skull	
Aurum and GOLD	24212007	1223219019	1223219019	58065	S352500	closed fracture intermediate cuneiform	
Aurum and GOLD	24832002	41612014	276160016	11342	7J12200	closed reduction of fracture of mandible nec	
Aurum and GOLD	24948002	41818011	41818011	43378	S351.00	open fracture of calcaneus	
Aurum and GOLD	25060006	41986016	567361000006114	26130	7K1LD00	closed reduction of fracture of nasal bone	
Aurum and GOLD	25415003	42617018	319140012	24276	S30w.00	closed fracture of unspecified proximal femur	
Aurum and GOLD	25415003	42617018	402875016	37662	S310000	closed fracture of femur, unspecified part	
Aurum and GOLD	25424007	42633015	794881000006119	3809	S00..11	frontal bone fracture	
Aurum and GOLD	25529004	42804019	402862016	44924	S235200	open fracture of the distal radius, unspecified	
Aurum and GOLD	25529004	42804019	632641000006117	105278	S235211	dupuytren's fracture, radius - open	
Aurum and GOLD	25899002	43394011	632621000006112	14826	S344.11	dupuytren's fracture, fibula	
Aurum and GOLD	25899002	43394011	561441000006114	7317	S344.00	closed fracture ankle, bimalleolar	
Aurum and GOLD	262525000	390470012	455022017	99895	S12y.00	fracture of other parts of bony thorax	
Aurum and GOLD	262525000	390470012	455023010	95839	S12y000	closed fracture of other parts of bony thorax	
Aurum and GOLD	262525000	390470012	455020013	58190	S12X000	closed fracture of bony thorax part unspecified	

Aurum and GOLD	263004001	391111016	391111016	868	S4...13	fracture dislocations and fracture subluxations	
Aurum and GOLD	263063009	391178016	29481000006111	40752	S4J..00	other fracture-dislocation or subluxation	
Aurum and GOLD	263063009	391178016	25171000006116	38590	S4J1.00	other open fracture-dislocation	
Aurum and GOLD	263063009	391178016	34791000006116	34993	S4J0.00	other closed fracture-dislocation	
Aurum and GOLD	263094009	391211011	34801000006115	71622	S4J2.00	other closed fracture-subluxation	
Aurum and GOLD	263094009	391211011	25181000006118	111125	S4J3.00	other open fracture-subluxation	
Aurum and GOLD	263102004	391219013	565681000006119	100350	S4C2y00	closed fracture-subluxation other carpal	
Aurum and GOLD	263105002	391222010	565611000006114	60487	S4D2200	closed fracture-subluxation ipj, unspecified	
Aurum and GOLD	263151001	2536004017	2536004017	4225	S024000	closed fracture maxilla	
Aurum and GOLD	263151001	391281018	391281018	36448	S024.11	fracture of upper jaw, closed	
Aurum and GOLD	263152008	391282013	391282013	31797	S04..11	multiple face fractures	
Aurum and GOLD	263171005	391312019	391312019	5280	S028000	fracture of nasal bones	
Aurum and GOLD	263172003	391313012	391313012	11161	S028300	fracture of mandible	
Aurum and GOLD	263172003	391314018	391425014	17455	S02z.11	jaw fracture nos	
Aurum and GOLD	263178004	391324014	793681000006116	30058	S10..11	fracture of transverse process spine - no spinal cord lesion	
Aurum and GOLD	263179007	391325010	793671000006119	72324	S11..11	fracture of transverse process of spine + spinal cord lesion	
Aurum and GOLD	263188003	391338019	531581000006112	69098	S101111	c1 vertebra open fracture without spinal cord lesion	
Aurum and GOLD	263189006	391339010	531611000006116	97120	S101211	c2 vertebra open fracture without spinal cord lesion	
Aurum and GOLD	263192005	391345019	391345019	1548	S228.00	fracture of lower end of humerus	
Aurum and GOLD	263195007	391349013	391349013	2303	S237.00	fracture of upper end of radius	
Aurum and GOLD	263198009	391352017	318832012	60518	S233000	open fracture of radius, shaft, unspecified	
Aurum and GOLD	263198009	391352017	391352017	7988	S239.00	fracture of shaft of radius	
Aurum and GOLD	263198009	391352017	318829014	51364	S232000	closed fracture of radius, shaft, unspecified	
Aurum and GOLD	263199001	391354016	391354016	199	S23B.00	fracture of lower end of radius	
Aurum and GOLD	263203001	391359014	391359014	33404	S236.00	fracture of upper end of ulna	
Aurum and GOLD	263204007	391360016	391360016	8382	S238.00	fracture of shaft of ulna	
Aurum and GOLD	263207000	391363019	793501000006112	10149	S23A.00	fracture of shafts of both ulna and radius	

Aurum and GOLD	263208005	391366010	391366010	6213	S23C.00	fracture of lower end of both ulna and radius	
Aurum and GOLD	263222005	391380015	318698011	65084	S135.00	other or multiple open fracture of pelvis	
Aurum and GOLD	263222005	391380015	318697018	11639	S134z00	other or multiple closed fracture of pelvis nos	
Aurum and GOLD	263225007	391384012	391384012	1994	S30..11	hip fracture	
Aurum and GOLD	263225007	391384012	391393013	10570	S30y.11	hip fracture nos	
Aurum and GOLD	263229001	391392015	391392015	8243	S305.00	subtrochanteric fracture	
Aurum and GOLD	263231005	391394019	391394019	100771	S301311	open fracture base of neck of femur	
Aurum and GOLD	263232003	391396017	391396017	8589	S315.00	fracture of lower end of femur	
Aurum and GOLD	263233008	2536032014	222331000000113	28954	S312.00	closed fracture distal femur	
Aurum and GOLD	263233008	391397014	391397014	22329	S312.11	closed fracture of femur, distal end	
Aurum and GOLD	263233008	391397014	319166010	61802	S312z00	closed fracture of distal femur not otherwise specified	
Aurum and GOLD	263233008	391397014	319159014	53279	S312000	closed fracture of distal femur, unspecified	
Aurum and GOLD	263234002	391398016	391398016	32866	S313.11	open fracture of femur, distal end	
Aurum and GOLD	263234002	391398016	319170019	45529	S313000	open fracture distal femur, unspecified	
Aurum and GOLD	263234002	391398016	319177016	88737	S313z00	open fracture of distal femur not otherwise specified	
Aurum and GOLD	263234002	391398016	222341000000116	51170	S313.00	open fracture distal femur	
Aurum and GOLD	263237009	391401018	391401018	22761	S330012	closed fracture of tibial tuberosity	
Aurum and GOLD	263238004	391402013	391402013	49801	S331012	open fracture of tibial tuberosity	
Aurum and GOLD	263239007	391403015	391403015	93029	S331011	open fracture of tibial condyles	
Aurum and GOLD	263244000	391410014	222361000000115	2250	S344.12	pott's fracture - ankle	
Aurum and GOLD	263245004	391412018	325468010	97380	Syu9400	[x]fracture of other tarsal bones	
Aurum and GOLD	263245004	391412018	319304015	100196	S353000	open fracture of tarsal bone, unspecified	
Aurum and GOLD	263245004	391412018	107341000006118	2710	S35..12	tarsal bone fracture	
Aurum and GOLD	263246003	391413011	391413011	2442	S355.00	fracture of talus	
Aurum and GOLD	263247007	391414017	391414017	1857	S354.00	fracture of calcaneus	
Aurum and GOLD	263247007	391414017	222381000000112	15166	S350.12	os calcis fracture	
Aurum and GOLD	263247007	391416015	391416015	8263	S350.11	heel bone fracture	
Aurum and GOLD	263251009	391420016	391420016	169	S35..11	metatarsal bone fracture	
Aurum and GOLD	263251009	391421017	391421017	6062	S356.00	fracture of metatarsal bone	

Aurum and GOLD	263253007	391423019	391423019	1700	S352.11	march fracture	
Aurum and GOLD	263256004	391426010	391426010	68899	S29..13	multiple fractures of sternum	
Aurum and GOLD	26442006	1225039011	1225039011	24674	S310100	closed fracture shaft of femur	
Aurum and GOLD	26442006	44288010	319147010	6868	S310.00	closed fracture of femur, shaft or unspecified part	
Aurum and GOLD	265132005	393775016	207611000006114	5742	7K1D000	prmy open red+int fxn prox femoral #+screw/nail+plate device	
Aurum and GOLD	265132005	393775016	208701000006117	107932	7K1D016	prim open reduct # neck femur & op fix - massie nail plate	
Aurum and GOLD	265132005	393775016	208691000006117	105352	7K1D015	prim open reduct # neck femur & op fix - jewett nail plate	
Aurum and GOLD	265132005	393775016	208671000006118	58817	7K1D011	prim open reduct # neck femur & op fix - blount nail plate	
Aurum and GOLD	265132005	393775016	208641000006114	56568	7K1D017	prim open red # neck femur & op fix - mclaughlin nail plate	
Aurum and GOLD	265132005	393775016	208711000006119	46258	7K1D018	prim open reduct # neck femur & op fix - neufield nail plate	
Aurum and GOLD	265132005	393775016	208611000006110	57889	7K1D01D	prim op red # nck femur & op fix- zickel intramed nail plate	
Aurum and GOLD	265132005	393775016	208721000006110	65536	7K1D019	prim open reduct # neck femur & op fix - pugh nail plate	
Aurum and GOLD	265134006	393777012	208631000006116	47253	7K1D212	prim open red # long bone & fix - rush flexi intramedul nail	
Aurum and GOLD	265134006	393777012	208621000006119	73241	7K1D211	prim open red # long bone & fix - ender flexi intramed nail	
Aurum and GOLD	265134006	393777012	208821000006115	38741	7K1D200	prim open reduct fract long bone & fixation flexible nail	
Aurum and GOLD	265138009	393781012	207621000006118	34082	7K1E600	prmy open reduction # elbow+fixation with hook fixtn plate	
Aurum and GOLD	265139001	393782017	208581000006119	47709	7K1F200	prim fixat fragment chondral cartilage intraartic fract bone	
Aurum and GOLD	265705007	394569015	276161017	27361	7J12y00	other specified reduction of fracture of mandible	

Aurum and GOLD	265705007	394569015	411080018	17443	7J12.11	reduction of fracture of jaw nec	
Aurum and GOLD	265705007	394569015	394569015	6994	7J12.00	reduction of fracture of mandible	
Aurum and GOLD	265705007	394569015	276162012	37297	7J12z00	reduction of fracture of mandible nos	
Aurum and GOLD	265721002	394587017	761191000006114	20598	7J43.00	fixation of fracture of spine	
Aurum and GOLD	265721002	394587017	276583011	60352	7J43z00	fixation of fracture of spine nos	
Aurum and GOLD	265721002	394587017	761201000006112	63980	7J43200	fixation of fracture of spine and skull traction hfq	
Aurum and GOLD	265721002	394587017	276582018	73344	7J43y00	other specified fixation of fracture of spine	
Aurum and GOLD	265721002	394587017	503551000006112	63954	7J43211	barr skull traction for fracture of spine	
Aurum and GOLD	265721002	394587017	204991000006116	91649	7J43700	primary open reduc spinal fracture+other internal fix	
Aurum and GOLD	26646003	1225102013	1225102013	49821	S353300	open fracture cuboid	
Aurum and GOLD	267765006	399868018	506042015	38131	7K1LN00	closed reduction of fracture of upper limb	
Aurum and GOLD	267765006	399868018	506043013	31498	7K1LN11	closed reduction # arm	
Aurum and GOLD	268029009	400338014	359835018	28575	N331.11	collapse of spine nos	
Aurum and GOLD	268029009	400338014	312178013	62343	N331z00	pathological fracture nos	
Aurum and GOLD	268029009	400338014	312177015	29332	N331y00	other specified pathological fracture	
Aurum and GOLD	268029009	400338014	400338014	5526	N331.00	pathological fracture	
Aurum and GOLD	268029009	400339018	129911000006119	100677	N331.13	sponanteous fracture	
Aurum and GOLD	269057007	402827014	793761000006117	70282	S025.11	fracture of upper jaw, open	
Aurum and GOLD	269057007	402827014	402827014	48636	S025.00	fracture of malar or maxillary bones, open	
Aurum and GOLD	269061001	402831015	318495015	34166	S10z.00	fracture of spine without mention of spinal cord lesion nos	
Aurum and GOLD	269061001	402831015	402831015	4409	S10..12	fracture of vertebra without spinal cord lesion	
Aurum and GOLD	269061001	402832010	402832010	8255	S10..00	fracture of spine without mention of spinal cord injury	
Aurum and GOLD	269062008	402833017	318391013	41930	S100z00	closed fracture of cervical spine not otherwise specified	
Aurum and GOLD	269062008	402833017	318353013	15613	S100000	closed fracture of unspecified cervical vertebra	
Aurum and GOLD	269062008	402833017	402833017	11296	S100.00	closed fracture of cervical spine	

Aurum and GOLD	269063003	402834011	402834011	5445	S100100	closed fracture atlas	
Aurum and GOLD	269064009	402835012	402835012	16277	S100200	closed fracture axis	
Aurum and GOLD	269065005	402836013	402836013	60593	S100300	closed fracture of third cervical vertebra	
Aurum and GOLD	269066006	402837016	402837016	41548	S100400	closed fracture of fourth cervical vertebra	
Aurum and GOLD	269067002	402838014	402838014	27575	S100500	closed fracture of fifth cervical vertebra	
Aurum and GOLD	269068007	402839018	402839018	27654	S100600	closed fracture of sixth cervical vertebra	
Aurum and GOLD	269069004	402840016	402840016	24672	S100700	closed fracture of seventh cervical vertebra	
Aurum and GOLD	269070003	402841017	318396015	101574	S101000	open fracture of unspecified cervical vertebra	
Aurum and GOLD	269070003	402841017	402841017	55627	S101.00	open fracture of cervical spine	
Aurum and GOLD	269071004	402842012	402842012	69645	S101100	open fracture atlas	
Aurum and GOLD	269072006	402843019	402843019	53976	S101200	open fracture axis	
Aurum and GOLD	269073001	402844013	531631000006110	60382	S101311	c3 vertebra open fracture without spinal cord lesion	
Aurum and GOLD	269074007	402845014	531651000006115	112794	S101411	c4 vertebra open fracture without spinal cord lesion	
Aurum and GOLD	269075008	402846010	402846010	24671	S101500	open fracture of fifth cervical vertebra	
Aurum and GOLD	269075008	402846010	531671000006113	99151	S101511	c5 vertebra open fracture without spinal cord lesion	
Aurum and GOLD	269076009	402847018	531691000006114	62719	S101611	c6 vertebra open fracture without spinal cord lesion	
Aurum and GOLD	269076009	402847018	402847018	65300	S101600	open fracture of sixth cervical vertebra	
Aurum and GOLD	269077000	402848011	531711000006112	59996	S101711	c7 vertebra open fracture without spinal cord lesion	
Aurum and GOLD	269077000	402848011	402848011	112983	S101700	open fracture of seventh cervical vertebra	
Aurum and GOLD	269078005	402849015	402849015	43786	S11..12	fracture of vertebra with spinal cord lesion	
Aurum and GOLD	269078005	402850015	318601013	55195	S11z.00	fracture of spine with spinal cord lesion nos	
Aurum and GOLD	269078005	402850015	402850015	32063	S11..00	fracture of spine with spinal cord lesion	
Aurum and GOLD	269078005	402850015	318599011	30956	S11x.00	closed fracture of spine with spinal cord lesion unspecified	
Aurum and GOLD	269080004	402852011	318792016	33540	S224z00	closed fracture of distal humerus, not otherwise specified	

Aurum and GOLD	269080004	402852011	318786010	32348	S224400	closed fracture of distal humerus, condyle(s) unspecified	
Aurum and GOLD	269080004	402854012	402854012	15376	S224.00	closed fracture of the distal humerus	
Aurum and GOLD	26908008	1225141010	1225141010	18584	S345.00	open fracture ankle, bimalleolar	
Aurum and GOLD	269081000	402856014	402856014	34172	S225.00	open fracture of the distal humerus	
Aurum and GOLD	269081000	402857017	318806018	47839	S225z00	open fracture of distal humerus, not otherwise specified	
Aurum and GOLD	269081000	402857017	318799013	86803	S225400	open fracture of distal humerus, condyle(s) unspecified	
Aurum and GOLD	269083002	402859019	402859019	343	S234100	closed colles' fracture	
Aurum and GOLD	269090007	402866018	696421000006117	29097	S29..00	multiple # both upper limbs & upper limb with rib + sternum	
Aurum and GOLD	269090007	402866018	696451000006114	70987	S29z.00	multiple #upper limbs & upper limb with rib + sternum nos	
Aurum and GOLD	269100000	402877012	319303014	67007	S353.00	open fracture of other tarsal and metatarsal bones	
Aurum and GOLD	269100000	402877012	319318010	39733	S35z.00	fracture of tarsal and metatarsal bones nos	
Aurum and GOLD	269100000	402877012	319314012	48925	S353z00	open fracture of tarsal and metatarsal bones nos	
Aurum and GOLD	269100000	402877012	402878019	15927	S352.00	closed fracture of other tarsal and metatarsal bones	
Aurum and GOLD	269100000	402877012	402877012	845	S35..00	fracture of one or more tarsal and metatarsal bones	
Aurum and GOLD	26938002	45058017	258471000006114	39396	S303400	open fracture of femur, intertrochanteric	
Aurum and GOLD	270507001	405143010	405143010	30616	N331000	pathological fracture of thoracic vertebra	
Aurum and GOLD	270508006	405144016	405144016	30352	N331100	pathological fracture of lumbar vertebra	
Aurum and GOLD	272300006	407419015	210031000006112	55386	7K1JB00	primary cls red+int fxn prox fem #+screw/nail device alone	
Aurum and GOLD	274156000	409924017	570631000006115	100110	S110500	cls spinal # with incomplete cervical cord lesion, c1-4 nos	
Aurum and GOLD	274156000	409924017	570641000006113	101956	S110B00	cls spinal # with incomplete cervical cord lesion, c5-7 nos	
Aurum and GOLD	274157009	409925016	570651000006110	108469	S112500	cls spinal # with incomplete thoracic cord lesion, t1-6 nos	

Aurum and GOLD	274157009	409925016	43221000006113	101299	S113000	opn spinal fracture with unspec thoracic cord lesion, t1-6	
Aurum and GOLD	274157009	409925016	570831000006116	43091	S112600	cls spinal fracture with unspec thoracic cord lesion, t7-12	
Aurum and GOLD	274157009	409925016	570661000006112	70475	S112B00	cls spinal # with incomplete thoracid cord lesion, t7-12 nos	
Aurum and GOLD	274157009	409925016	570841000006114	108484	S112000	cls spinal fracture with unspec thoracic cord lesion,t1-6	
Aurum and GOLD	274158004	409926015	318557010	49567	S114000	closed spinal fracture with unspecified lumbar cord lesion	
Aurum and GOLD	274160002	2536029011	2536029011	7500	S262.00	fracture of thumb	
Aurum and GOLD	274160002	2536029011	100941000006118	482	S26..12	thumb fracture excluding base	
Aurum and GOLD	274474001	410281019	277163010	99994	7K1K900	other primary external immobilisation of fracture	
Aurum and GOLD	27477003	1225363010	402828016	25631	S02z.00	fracture of facial bone nos	
Aurum and GOLD	27477003	1225363010	45939011	9103	S02..00	fracture of face bones	
Aurum and GOLD	27477003	45939011	318235016	68940	S025z00	fracture of malar or maxillary bones, open, nos	
Aurum and GOLD	27477003	45939011	318252017	35312	S02yz00	fracture of other facial bones,open, nos	
Aurum and GOLD	27477003	45939011	318229016	49644	S024z00	fracture of malar or maxillary bones, closed, nos	
Aurum and GOLD	274986006	410903011	410903011	29582	7K1D511	k wiring of fracture	
Aurum and GOLD	275093002	411019014	411019014	62489	7J43.11	internal fixation of fracture of spine	
Aurum and GOLD	275337006	411304016	411304016	10622	S221.11	shoulder fracture - open	
Aurum and GOLD	275338001	411305015	411305015	68229	S300y11	closed fracture of femur, subcapital	
Aurum and GOLD	275338001	411305015	570551000006114	17019	S300500	cls # prox femur, subcapital, garden grade unspec.	
Aurum and GOLD	275339009	411306019	411306019	73234	S301y11	open fracture of femur, subcapital	
Aurum and GOLD	275340006	411307011	411307011	19387	S302011	closed fracture of femur, greater trochanter	
Aurum and GOLD	275341005	411308018	411308018	48337	S302012	closed fracture of femur, lesser trochanter	
Aurum and GOLD	275342003	411309014	411309014	112991	S303012	open fracture of femur, lesser trochanter	
Aurum and GOLD	275343008	411310016	411310016	96644	S303011	open fracture of femur, greater trochanter	

Aurum and GOLD	27644009	46274013	560031000006116	102074	S010600	closed #skull bse no intracranial inj, loc unspec duration	
Aurum and GOLD	27644009	46274013	560061000006113	94450	S010200	closed #skull bse no intracranial injury, <1hr loss of consc	
Aurum and GOLD	27644009	46274013	561661000006110	62977	S010.00	closed fracture base of skull without intracranial injury	
Aurum and GOLD	27644009	46274013	560091000006117	58957	S010100	closed #skull bse no intracranial injury, no loss of consc	
Aurum and GOLD	27644009	46274013	560051000006111	96406	S010z00	closed #skull bse no intracranial injury + concussion unspec	
Aurum and GOLD	27644009	46274013	560071000006118	102084	S010400	closed #skull bse no intracranial injury, >24hr loc+recovery	
Aurum and GOLD	278537006	415489016	415489016	10007	S338.00	fracture of lower end of tibia	
Aurum and GOLD	27930000	46755018	46755018	4641	7K1T100	debridement of open fracture	
Aurum and GOLD	28012007	46894013	564861000006114	34021	S332000	closed fracture shaft of tibia	
Aurum and GOLD	28078000	3331778016	318831017	33808	S232z00	closed fracture of radius and ulna, shaft, nos	
Aurum and GOLD	28081005	1225771011	1225771011	71452	S121900	open fracture multiple ribs	
Aurum and GOLD	281531008	419562016	419562016	5009	S348.00	fracture of medial malleolus	
Aurum and GOLD	281535004	419567010	419567010	6731	S349.00	fracture of lateral malleolus	
Aurum and GOLD	281797006	419891012	567011000006114	36497	7K1Jz00	closed reduction of bone fracture and internal fixation nos	
Aurum and GOLD	281797006	419891012	166521000006116	40779	7K1Ja00	revision to closed reduction # + other internal fixation	History
Aurum and GOLD	281797006	419891012	567021000006118	42186	7K1Jy00	closed reduction of bone fracture and internal fixation os	
Aurum and GOLD	281797006	419891012	209721000006119	12601	7K1JT00	primary closed reduction #+other internal fixation	
Aurum and GOLD	281807006	419908011	42961000006117	20379	7K6F400	open reduction of # dislocation of joint+fxn of jnt,unspec	
Aurum and GOLD	28359007	1225880017	1225880017	52499	S330800	closed fracture fibula, head	
Aurum and GOLD	28576007	47832017	319141011	58642	S30x.00	open fracture of unspecified proximal femur	
Aurum and GOLD	28753006	48140010	531571000006114	42561	S100111	c1 vertebra closed fracture - no spinal cord lesion	

Aurum and GOLD	28753006	48141014	391329016	112617	S100.11	closed fracture of atlas without spinal cord lesion	
Aurum and GOLD	29014003	48573010	48573010	65141	S241.00	open fracture of carpal bone	
Aurum and GOLD	29014003	48573010	318926018	68085	S241000	open fracture of carpal bone, unspecified	
Aurum and GOLD	29014003	48573010	318935013	102013	S241z00	open fracture of carpal bone nos	
Aurum and GOLD	29045004	48625014	48625014	2662	S230300	closed monteggia's fracture	
Aurum and GOLD	297282004	437774015	207701000006116	17508	7K1E000	prmy open reduction of #+internal fixation with plate nec	
Aurum and GOLD	29749002	49786013	49786013	27620	S210.00	closed fracture of scapula	
Aurum and GOLD	29749002	49786013	318735017	38028	S210000	closed fracture of scapula, unspecified part	
Aurum and GOLD	29749002	49786013	318741012	57592	S210z00	closed fracture of scapula nos	
Aurum and GOLD	301050003	442280015	209681000006114	99508	7K6Gd00	primary closed reduction # dislocation+other extrnal immobil	
Aurum and GOLD	301050003	442280015	277940018	61653	7K6GX00	primary closed reduction of fracture dislocation alone	
Aurum and GOLD	301050003	442280015	204771000006119	23966	7K6G200	primary manipulative closed reduct fract dislocat joint nec	
Aurum and GOLD	301051004	442281016	166551000006113	101295	7K6HW00	revision to closed reduction # dislocation+other ext immobil	History
Aurum and GOLD	301051004	442281016	277956011	35866	7K6H400	revision to closed reduction of fracture dislocation alone	History
Aurum and GOLD	301051004	442281016	277957019	69362	7K6H411	remanipulation of fracture dislocation alone	History
Aurum and GOLD	301052006	442282011	277994011	99297	7K6HX00	revision to open reduction fracture dislocation alone	History
Aurum and GOLD	301052006	442282011	151881000006116	54198	7K6H200	secondary open reduction fracture dislocation of joint nec	
Aurum and GOLD	301052006	442282011	167061000006115	113257	7K6Hg00	revision to open reduction # dislocatn+oth stabilisation jt	History
Aurum and GOLD	301053001	442283018	277885018	24611	7K6F200	primary open reduction of fracture dislocation of joint nec	
Aurum and GOLD	301053001	442283018	277068016	16141	7K1Gz00	other primary open reduction of fracture of bone nos	
Aurum and GOLD	301053001	442283018	277900012	55930	7K6FE00	primary open reduction of fracture dislocation alone	

Aurum and GOLD	301053001	442283018	205021000006118	88761	7K6FP00	primary open reduction # dislocation+other jnt stabilisation	
Aurum and GOLD	302222008	443804014	318783019	33720	S224000	closed fracture of elbow, unspecified part	
Aurum and GOLD	302222008	443804014	443804014	1250	S224.11	elbow fracture - closed	
Aurum and GOLD	302232001	443817012	443817012	7754	S225.11	elbow fracture - open	
Aurum and GOLD	302232001	443817012	318796018	44142	S225000	open fracture of elbow, unspecified part	
Aurum and GOLD	302472007	2642653017	276103016	3095	7J03100	reduction of fracture of nasal bones nec	
Aurum and GOLD	306171000000106	545121000000114	545121000000114	93497	N331N00	fragility fracture	
Aurum and GOLD	306171000000106	557111000000111	557111000000111	93981	N331N11	minimal trauma fracture	
Aurum and GOLD	30632004	1227288016	1227288016	28413	S240300	closed fracture triquetral	
Aurum and GOLD	307285000	450511010	276165014	51392	7J13100	open reduction of fracture of maxilla nec	
Aurum and GOLD	307288003	450514019	276166010	35889	7J13200	closed reduction of fracture of maxilla nec	
Aurum and GOLD	307289006	450515018	276159014	28926	7J12100	open reduction of fracture of mandible nec	
Aurum and GOLD	307372000	450619018	566921000006117	97838	7K1Kz11	closed reduction bone fract & fix with gissane spike fixator	
Aurum and GOLD	307385002	450635013	208811000006111	57558	7K1E013	prim open reduct fract long bone & fix using hicks plate	
Aurum and GOLD	307386001	450636014	208801000006113	60121	7K1E012	prim open reduct fract long bone & fix using ellis plate	
Aurum and GOLD	307387005	450637017	208791000006112	36872	7K1E011	prim open reduct fract long bone & fix using eggers plate	
Aurum and GOLD	307712005	451060018	451060018	18301	S235F00	open barton's fracture	
Aurum and GOLD	307713000	451061019	451061019	10033	S234F00	closed barton's fracture	
Aurum and GOLD	307727005	451079015	451079015	12369	S339100	open fracture of distal fibula	
Aurum and GOLD	308145001	451463012	451463012	37945	N331C00	pathological fracture of cervical vertebra	
Aurum and GOLD	308153009	451471011	451471011	6839	S339000	closed fracture of distal fibula	
Aurum and GOLD	308756007	452079017	452079017	9319	N331F00	collapse of thoracic vertebra	
Aurum and GOLD	308756007	452079017	572221000006119	15837	N331011	collapse of thoracic vertebra	
Aurum and GOLD	308757003	452080019	452080019	17008	N331E00	collapse of cervical vertebra	
Aurum and GOLD	308757003	452080019	572141000006117	110999	N331C11	collapse of cervical vertebra	
Aurum and GOLD	308758008	452081015	452081015	11543	N331G00	collapse of lumbar vertebra	

Aurum and GOLD	308758008	452081015	572171000006113	23686	N331111	collapse of lumbar vertebra	
Aurum and GOLD	30905007	1227400011	1227400011	5332	S312300	closed fracture distal femur, supracondylar	
Aurum and GOLD	310865004	454383013	205051000006110	7930	7K1F500	primary open reduction fracture patella fixat tension band	
Aurum and GOLD	311408004	454977016	454977016	65151	S124000	closed flail chest	
Aurum and GOLD	311426001	454997014	166961000006116	28972	7K1HS00	revision to open red #+other int fxn	History
Aurum and GOLD	311426001	454997014	167991000006110	28464	7K1H600	revsn to opn red+int fxtn prox fem #+screw/nail device alone	History
Aurum and GOLD	311428000	454999012	1702461000006117	104338	7K1Yz00	second closed reduc fract of bone and internal fixation nos	
Aurum and GOLD	311428000	454999012	1702451000006119	107718	7K1Yy00	os second closed reduct fracture bone and internal fixation	
Aurum and GOLD	311428000	454999012	1702411000006115	104066	7K1Y100	remanip fracture long bone and rigid internal fixation nec	History
Aurum and GOLD	311428000	746831000000111	1702391000006115	102254	7K1Y.00	second closed reduction fracture bone and internal fixation	
Aurum and GOLD	311488002	455069010	276510019	110247	7J42J00	primary closed reduction spinal fracture alone	
Aurum and GOLD	311806008	455433012	455433012	45736	N331H00	collapse of cervical vertebra due to osteoporosis	
Aurum and GOLD	311814002	455444016	455444016	35260	S150000	closed multiple fractures of thoracic spine	
Aurum and GOLD	311815001	455445015	455445015	66322	S150100	open multiple fracture of thoracic spine	
Aurum and GOLD	311816000	455446019	455446019	40976	S292000	closed multiple fractures of clavicle, scapula and humerus	
Aurum and GOLD	311817009	455447011	455447011	66237	S292100	open multiple fractures of clavicle, scapula and humerus	
Aurum and GOLD	311819007	455449014	557941000006115	95633	S294000	cl fractures involving multiple regions of both upper limbs	
Aurum and GOLD	311821002	455451013	455451013	28604	S362000	closed fracture of great toe	
Aurum and GOLD	311822009	455452018	455452018	31847	S362100	open fracture of great toe	
Aurum and GOLD	311847006	455485014	455485014	73786	SR10000	closed fractures involving head with neck	

Aurum and GOLD	311849009	455487018	565921000006111	92830	SR12000	closed fractures involving multiple regions of one upp limb	
Aurum and GOLD	311857007	455496018	557951000006118	103049	SR15000	cl fractures involving multiple regions upper with lower lmb	
Aurum and GOLD	311862008	455501017	562351000006114	57223	SR16000	closed fracture inv thorax wth low back and pelvis and limbs	
Aurum and GOLD	311890007	455534015	455534015	5841	N331J00	collapse of lumbar vertebra due to osteoporosis	
Aurum and GOLD	311891006	455535019	455535019	19048	N331K00	collapse of thoracic vertebra due to osteoporosis	
Aurum and GOLD	313330006	457218015	567461000006115	39322	7K1Jd00	closed reduction of intracapsular # nof internal fixat dhs	
Aurum and GOLD	316890006	461550019	318521000006110	111268	Zw02D00	[q] open fracture grade 1	
Aurum and GOLD	316891005	461553017	318531000006113	95619	Zw02E00	[q] open fracture grade 2	
Aurum and GOLD	316892003	461554011	318541000006115	113178	Zw02F00	[q] open fracture grade 3	
Aurum and GOLD	31975004	53424011	793411000006114	553	S242000	fracture of scaphoid	
Aurum and GOLD	31978002	53428014	53428014	78444	S33A.00	fracture of tibia	
Aurum and GOLD	31984004	53444017	318721019	60608	S140.00	closed fracture of ill-defined bone of trunk	
Aurum and GOLD	3228009	1227930011	1227930011	42864	S232100	closed fracture of the radial shaft	
Aurum and GOLD	32805004	54758014	319337018	45517	S3x..00	other, multiple and ill-defined fractures of lower limb	
Aurum and GOLD	3291007	6555013	6555013	40533	S120200	closed fracture of two ribs	
Aurum and GOLD	33041006	1228028014	1228028014	34426	S230500	closed fracture of the proximal ulna	
Aurum and GOLD	33118001	1228040014	1228040014	1591	S130.00	closed fracture acetabulum	
Aurum and GOLD	33118001	55266013	318662015	59904	S130y00	other specified closed fracture acetabulum	
Aurum and GOLD	33118001	55266013	318661010	98267	S130600	closed fracture acetabulum, double column unspecified	
Aurum and GOLD	33118001	55266013	318663013	45527	S130z00	closed fracture acetabulum nos	
Aurum and GOLD	33173003	55358019	318730010	44715	S200000	closed fracture of clavicle, unspecified part	
Aurum and GOLD	33173003	55358019	55358019	33749	S200.00	closed fracture of clavicle	
Aurum and GOLD	33173003	55358019	318731014	28179	S200z00	closed fracture of clavicle nos	
Aurum and GOLD	33192001	1490703015	1490703015	28708	S234600	closed fracture radius and ulna, distal	

Aurum and GOLD	33192001	485443016	485443016	18299	S234.00	closed fracture of radius and ulna, lower end	
Aurum and GOLD	33192001	55392016	318838011	18389	S234000	closed fracture of forearm, lower end, unspecified	
Aurum and GOLD	335771000000106	622771000000113	622771000000113	95674	N1y2.00	pars interarticularis stress fracture	
Aurum and GOLD	33737001	1228101019	391372010	11378	S12z.11	rib fracture nos	
Aurum and GOLD	33737001	56330019	56330019	9688	S127.00	fracture of rib	
Aurum and GOLD	3381004	6701012	6701012	39758	S353100	open fracture of talus	
Aurum and GOLD	3421000	1228153010	1228153010	33459	S027.00	open orbital blow-out fracture	
Aurum and GOLD	34268009	1228159014	1228159014	7340	S342.00	closed fracture ankle, lateral malleolus	
Aurum and GOLD	34578006	57701019	57701019	10246	S231300	open monteggia's fracture	
Aurum and GOLD	34649000	57835013	792961000006113	14878	S024.00	fracture of malar or maxillary bones, closed	
Aurum and GOLD	34656006	1228196017	1228196017	69213	S241800	open fracture hamate	
Aurum and GOLD	34813004	58102016	218101000006119	61757	S01..17	posterior fossa fracture	
Aurum and GOLD	35442005	59130011	318864017	27591	S234z00	closed fracture of forearm, lower end, nos	
Aurum and GOLD	357009	1228300012	1228300012	35837	S240600	closed fracture trapezoid	
Aurum and GOLD	359820003	475220015	402874017	18273	S30y.00	closed fracture of neck of femur nos	
Aurum and GOLD	361119006	477343011	319145019	38054	S30z.00	open fracture of neck of femur nos	
Aurum and GOLD	36127009	60285012	60285012	2328	S10B500	fracture of pubis	
Aurum and GOLD	36127009	60285012	318686018	70674	S133z00	open fracture of pubis nos	
Aurum and GOLD	36127009	60285012	318685019	94127	S133y00	other specified open fracture of pubis	
Aurum and GOLD	36417005	2551528018	2551528018	34708	S134100	closed fracture pelvis, ischium	
Aurum and GOLD	36924003	1228726016	1228726016	3937	S352700	closed fracture metatarsal	
Aurum and GOLD	37027003	1228733016	749221000006111	68981	S02C.00	le fort iii fracture maxilla	
Aurum and GOLD	371161001	1209863015	264331000006115	65532	S032z00	open #skull nos no intracranial inj + concussion unspec	
Aurum and GOLD	371161001	1209863015	318285013	67603	S033.00	open fracture of skull nos with intracranial injury	
Aurum and GOLD	371161001	1209863015	264411000006116	94411	S032.00	open #skull nos without mention of intracranial injury	
Aurum and GOLD	371162008	1209864014	560161000006112	64550	S031600	closed #skull nos + intracranial inj, loc unspec duration	

Aurum and GOLD	371162008	1209864014	560151000006110	69491	S031300	closed #skull nos + intracranial inj, 1-24hrs loss of consc	
Aurum and GOLD	371162008	1209864014	560251000006119	112781	S030000	closed #skull nos no intracranial inj, unspec state of consc	
Aurum and GOLD	371162008	1209864014	318266010	27492	S031.00	closed fracture of skull nos with intracranial injury	
Aurum and GOLD	371162008	1209864014	560241000006116	66763	S030100	closed #skull nos no intracranial inj, no loss of consc	
Aurum and GOLD	371162008	1209864014	560191000006116	89688	S030z00	closed #skull nos no intracranial inj + concussion unspec	
Aurum and GOLD	371162008	1209864014	560121000006118	67890	S031200	closed #skull nos + intracranial inj, <1hr loss of consc	
Aurum and GOLD	371162008	1209864014	560201000006118	110319	S030200	closed #skull nos no intracranial inj, <1hr loss of consc	
Aurum and GOLD	371162008	1209864014	560221000006111	98578	S030300	closed #skull nos no intracranial inj, 1-24hr loss of consc	
Aurum and GOLD	371566003	1210202016	567001000006111	10102	7K1Ky00	closed reduction of bone fracture and external fixation os	
Aurum and GOLD	371566003	1210202016	566991000006115	64222	7K1Kz00	closed reduction of bone fracture and external fixation nos	
Aurum and GOLD	371566003	1210202016	566951000006114	64862	7K1K000	closed reduction fracture bone and fixation to skeleton hfq	
Aurum and GOLD	371566003	1210202016	560591000006111	24715	7K1K.00	closed (or no) reduction of fracture and external fixation	
Aurum and GOLD	37174005	1229092011	1229092011	31999	S240800	closed fracture hamate	
Aurum and GOLD	37418005	62415013	62415013	33908	S27..00	multiple fractures of hand bones	
Aurum and GOLD	37418005	62415013	319056016	53923	S27z.00	multiple fractures of hand bones nos	
Aurum and GOLD	37449000	62465016	318913017	28741	S23y200	open fracture of ulna (alone), unspecified	
Aurum and GOLD	37633006	1229331011	1229331011	10357	S240200	closed fracture lunate	
Aurum and GOLD	38404000	1229426013	1229426013	55687	S211100	open fracture scapula, acromion	
Aurum and GOLD	38567007	64952019	41971000006117	37609	S01..16	orbital roof fracture	
Aurum and GOLD	39335003	65958012	65958012	34197	S120400	closed fracture of four ribs	
Aurum and GOLD	39706009	66948017	66948017	6379	7K1LF00	closed reduction of fracture of humerus	
Aurum and GOLD	39993000	492363016	492363016	3748	S233.00	open fracture of radius and ulna, shaft	

Aurum and GOLD	39993000	66609019	318834013	27784	S233z00	open fracture of radius and ulna, shaft, nos	
Aurum and GOLD	405817008	2157591015	319053012	4582	S26z.00	fracture of one or more phalanges of hand nos	
Aurum and GOLD	405817008	2157591015	793161000006116	441	S26..00	fracture of one or more phalanges of hand	
Aurum and GOLD	40613008	1229658014	1229658014	3408	S021.00	open fracture nose	
Aurum and GOLD	40613008	1229659018	1229659018	37192	S021.11	open fracture nasal bone	
Aurum and GOLD	41036008	1229710011	1229710011	9165	S234300	closed fracture of ulna, styloid process	
Aurum and GOLD	41191003	68712015	259461000006112	73210	S301400	open fracture head, femur	
Aurum and GOLD	413875004	2533501014	564541000006113	28739	S220400	closed fracture proximal humerus, head	
Aurum and GOLD	413877007	2533502019	319243019	4572	S33x200	closed fracture of tibia and fibula, unspecified part	
Aurum and GOLD	413877007	2533502019	402876015	41971	S33xz00	closed fracture of tibia and fibula, unspecified part, nos	
Aurum and GOLD	413877007	2533502019	564071000006114	29109	S33x.00	closed fracture of tibia and fibula, unspecified part, nos	
Aurum and GOLD	414292006	2533504018	391381016	35253	S33x.11	lower leg fracture nos	
Aurum and GOLD	414292006	2533504018	325450012	96939	Syu8300	[x]fractures of other parts of lower leg	
Aurum and GOLD	414292006	2533504018	325460015	103024	Syu8D00	[x]fracture of lower leg, part unspecified	
Aurum and GOLD	414292006	2533504018	792901000006112	11275	S3X..00	fracture of lower leg, part unspecified	
Aurum and GOLD	414293001	2533505017	2533505017	2630	S33..00	fracture of tibia and fibula	
Aurum and GOLD	414293001	2533505017	319250015	25485	S33z.00	fracture of tibia and fibula, nos	
Aurum and GOLD	414943006	2533506016	319248011	29084	S33y200	open fracture of tibia and fibula, unspecified part	
Aurum and GOLD	41511005	1229766011	1229766011	28197	S261R00	open fracture finger distal phalanx	
Aurum and GOLD	41977005	1229819019	1229819019	49847	S352600	closed fracture lateral cuneiform	
Aurum and GOLD	42157000	70347013	318493010	3573	S10x.00	closed fracture of spine, unspecified,	
Aurum and GOLD	42188001	70403018	319271013	4737	S34x.00	closed fracture ankle, unspecified	
Aurum and GOLD	42261000	70514017	702621000006114	24135	S01..14	middle fossa fracture	
Aurum and GOLD	42306005	1229848013	1229848013	36304	S352H00	closed fracture of cuneiforms	
Aurum and GOLD	423125000	2649521016	319351018	37310	S3z0.00	closed fracture of bones, unspecified	
Aurum and GOLD	424648000	2645978011	312321000000110	28875	S352J00	closed fracture of base of fifth metatarsal	
Aurum and GOLD	424817005	2645979015	2645979015	67239	S353J00	open fracture of base of fifth metatarsal	

Aurum and GOLD	42591009	1229890019	1229890019	98933	S241600	open fracture trapezoid	
Aurum and GOLD	426236005	2675982016	2675982016	89101	7206800	internal fixation of fracture of orbit	
Aurum and GOLD	426243004	2674037014	2674037014	90472	7J41500	balloon kyphoplasty of fracture of spine	
Aurum and GOLD	42636007	1229897016	1229897016	11222	S220.00	closed fracture of the proximal humerus	
Aurum and GOLD	42636007	71124012	318758019	38353	S220z00	closed fracture of proximal humerus not otherwise specified	
Aurum and GOLD	42636007	71124012	318750014	44721	S220000	closed fracture of proximal humerus, unspecified part	
Aurum and GOLD	42760000	1229927010	1229927010	70503	S233200	open fracture of the ulnar shaft	
Aurum and GOLD	42818005	1229933018	1229933018	6168	S240100	closed fracture of the scaphoid	
Aurum and GOLD	428257007	2694844010	1756451000006110	101031	S336000	fracture tibial plateau	
Aurum and GOLD	429581009	2695635019	1554031000006118	91546	7K6FR00	primary open reduct # dislocat joint comb int external fix	
Aurum and GOLD	430984009	2770946011	318247010	26408	S02xz00	fracture of other facial bones, closed, nos	
Aurum and GOLD	430984009	2770946011	318244015	29119	S02x.00	closed fracture other facial bone	
Aurum and GOLD	43295006	72228016	318773018	36464	S222.00	closed fracture of humerus, shaft or unspecified part	
Aurum and GOLD	43295006	72228016	318774012	19186	S222000	closed fracture of humerus nos	
Aurum and GOLD	43337002	1494730015	1494730015	106283	S023700	open fracture of mandible, alveolar border of body	
Aurum and GOLD	43767005	1230026018	1230026018	5354	S240400	closed fracture pisiform	
Aurum and GOLD	441546003	2816901016	291971000006117	36524	SP22X00	# bone follow insertn/orthopaed implnt,jnt pros,bne plate	
Aurum and GOLD	442072000	2118121000000116	1702521000006110	103307	7K6GK00	prim closed reduc fract dislocat joint and internal fixation	
Aurum and GOLD	442085002	2818315017	807141000006110	455	S3z0000	greenstick fracture	
Aurum and GOLD	443165006	2837900019	312681016	57301	NyuB000	[x]other osteoporosis with pathological fracture	
Aurum and GOLD	443165006	2837900019	312689019	18825	NyuB800	[x]unspecified osteoporosis with pathological fracture	
Aurum and GOLD	443165006	2837900019	216206018	11503	N331M00	fragility fracture due to unspecified osteoporosis	
Aurum and GOLD	443165006	2837900019	556881000000111	93705	N331M11	minimal trauma fracture due to unspecified osteoporosis	

Aurum and GOLD	443611000000103	957761000000116	461544012	94530	Zw02500	[q] refracture	
Aurum and GOLD	44504005	1230102017	1230102017	64725	S241700	open fracture capitate	
Aurum and GOLD	445311003	1666481000000110	1175111000000113	100640	S33B.00	open fracture of distal tibia and fibula	
Aurum and GOLD	445410003	1666471000000113	1175081000000117	100202	S33C.00	closed fracture of distal tibia and fibula	
Aurum and GOLD	446979005	2882648014	319246010	29164	S33y000	open fracture of tibia, unspecified part, nos	
Aurum and GOLD	446980008	2884198012	1668051000000112	101840	S331A00	open fracture tibial plateau	
Aurum and GOLD	447138000	2883829019	319292013	20253	S352000	closed fracture of tarsal bone, unspecified	
Aurum and GOLD	447139008	2883830012	319241017	971	S33x000	closed fracture of tibia, unspecified part, nos	
Aurum and GOLD	447395005	2883200010	319242012	4304	S33x100	closed fracture of fibula, unspecified part, nos	
Aurum and GOLD	448037009	2899588010	566941000006112	15952	7K1J100	closed reduction fract long bone & rigid internal fixatn nec	
Aurum and GOLD	448355005	2900823018	1730651000000118	102302	S234G00	greenstick fracture of distal radius	
Aurum and GOLD	448572002	2899314017	1553421000006114	70533	7K1HV00	secondary open reduction # bone and internal fixation hfq	
Aurum and GOLD	448838000	2902340010	318859016	19058	S234D00	closed fracture distal radius, extra-articular, other type	
Aurum and GOLD	448873003	2900927018	966691000006118	32723	7K1EA00	prim open reduct fract ankle & extramedull fixat nec	
Aurum and GOLD	448873003	2900927018	208761000006116	29778	7K1E400	prim open reduct fract ankle & complex extramedull fixat nec	
Aurum and GOLD	44916009	1230159017	749211000006115	44949	S02B.00	le fort ii fracture maxilla	
Aurum and GOLD	44932007	74959012	74959012	112701	S121100	open fracture of one rib	
Aurum and GOLD	449557005	2902355018	966701000006118	50907	7K1EB00	prim open reduct fract ankle & complex extramedull fixat nec	
Aurum and GOLD	450817003	2917014011	166641000006112	67030	7K1KJ00	revision to closed reduction of # + oth ext immobilisation	History
Aurum and GOLD	45356009	75611015	75611015	16494	S120100	closed fracture of one rib	
Aurum and GOLD	45678008	494318018	494318018	31708	S29..11	multiple fractures of arm	
Aurum and GOLD	45910007	1230277019	1230277019	48224	S121.00	open fracture rib	
Aurum and GOLD	45910007	76554010	318624018	101560	S121z00	open fracture of rib(s) nos	

Aurum and GOLD	45910007	76554010	318614014	73613	S121000	open fracture of rib, unspecified	
Aurum and GOLD	46020007	1230293011	1230293011	40658	S353H00	open fracture cuneiforms	
Aurum and GOLD	46422008	77387018	562171000006118	25519	S250300	closed fracture finger metacarpal shaft	
Aurum and GOLD	4673003	1230391011	1230391011	18388	S343.00	open fracture ankle, lateral malleolus	
Aurum and GOLD	46866001	1230400018	78112010	8891	S3...00	fracture of lower limb	
Aurum and GOLD	46866001	1230401019	1230401019	2603	S3...11	leg fracture	
Aurum and GOLD	46866001	78112010	257181000006118	28233	S33y.00	open fracture of tibia and fibula, unspecified part, nos	
Aurum and GOLD	46866001	78112010	696441000006112	73700	S3yz.00	multiple #both legs, leg + arm, leg + rib + sternum nos	
Aurum and GOLD	46866001	78112010	319343016	14746	S3xz.00	other, multiple and ill-defined fractures of lower limb nos	
Aurum and GOLD	46866001	78112010	319249015	62787	S33yz00	open fracture of tibia and fibula, unspecified part, nos	
Aurum and GOLD	46866001	78112010	319333019	33903	S37..00	fracture of lower limb, level unspecified	
Aurum and GOLD	46866001	78112010	455453011	40368	S370.00	closed fracture of lower limb, level unspecified	
Aurum and GOLD	46866001	78112010	696431000006119	56299	S3y..00	multiple #both legs, leg + arm ,leg + rib + sternum	
Aurum and GOLD	46866001	78112010	455454017	44245	S371.00	open fracture of lower limb, level unspecified	
Aurum and GOLD	46995009	78317012	570821000006119	72711	S110600	cls spinal fracture with unspec cervical cord lesion, c5-7	
Aurum and GOLD	47264000	78788012	560601000006115	6248	7K1J.00	closed (or no) reduction of fracture and internal fixation	
Aurum and GOLD	47264000	78788012	566931000006119	99527	7K1J200	closed reduction fract long bone & flexible intern fixat hfq	
Aurum and GOLD	47402002	79031011	79031011	44826	S121200	open fracture of two ribs	
Aurum and GOLD	4750004	8921014	1230477016	16866	S225700	open fracture distal humerus, medial epicondyle	
Aurum and GOLD	4750004	8921014	259881000006117	69312	S225300	open fracture distal humerus, medial condyle	
Aurum and GOLD	47848000	79735012	564111000006118	53951	S330011	closed fracture of tibial condyles	
Aurum and GOLD	47864008	79758010	318742017	73768	S211000	open fracture of scapula, unspecified part	
Aurum and GOLD	47864008	79758010	318748018	94435	S211z00	open fracture of scapula nos	
Aurum and GOLD	47864008	79758010	79758010	35386	S211.00	open fracture of scapula	

Aurum and GOLD	4788002	1230529018	1230529018	2251	S024100	closed fracture zygoma	
Aurum and GOLD	48187004	80287011	319272018	38765	S34y.00	open fracture ankle, unspecified	
Aurum and GOLD	48466003	80749019	80749019	15184	S00..00	fracture of vault of skull	
Aurum and GOLD	48466003	80749019	318144013	20195	S00z.00	fracture of vault of skull nos	
Aurum and GOLD	48561006	1230612011	1230612011	44711	S200100	closed fracture clavicle, medial end	
Aurum and GOLD	48752009	81242018	81242018	40078	S125100	closed fracture of hyoid bone	
Aurum and GOLD	49128003	81844012	694981000006118	39859	S04..12	multiple skull fractures	
Aurum and GOLD	49346003	1230706011	1230706011	24790	S026.00	closed orbital blow-out fracture	
Aurum and GOLD	50397009	83962012	318846012	40476	S234500	closed fracture distal ulna, unspecified	
Aurum and GOLD	50890004	84797011	84797011	30659	S227.00	fracture of shaft of humerus	
Aurum and GOLD	51037009	85039010	291981000006119	1093	S32..11	#knee-cap	
Aurum and GOLD	51037009	85039010	85039010	235	S32..00	fracture of patella	
Aurum and GOLD	51037009	85039010	319194019	35011	S32z.00	fracture of patella, nos	
Aurum and GOLD	52271005	86982016	134471000006115	37686	S01..18	sphenoid bone fracture	
Aurum and GOLD	52603002	87542010	696351000006119	105691	S3y0.00	multiple closed #both legs, leg + arm, leg + rib + sternum	
Aurum and GOLD	52622006	87580011	531641000006117	67358	S100411	c4 vertebra closed fracture without spinal cord lesion	
Aurum and GOLD	53256003	1231198017	1231198017	68765	S241200	open fracture lunate	
Aurum and GOLD	53627009	496729019	496729019	4359	S23x300	closed fracture of the radius and ulna	
Aurum and GOLD	53627009	89155012	318901010	15764	S23x.00	closed fracture of radius and ulna, unspecified part	
Aurum and GOLD	53627009	89155012	318909012	36328	S23xz00	closed fracture of radius and ulna, nos	
Aurum and GOLD	53792000	1231252012	1231252012	44790	S232200	closed fracture of the ulnar shaft	
Aurum and GOLD	54075005	1231287012	1231287012	66231	S353400	open fracture medial cuneiform	
Aurum and GOLD	54355006	90339018	768961000006113	32298	S6z..00	intracranial injury, excluding those with skull fracture nos	
Aurum and GOLD	54355006	90339018	768881000006110	35456	S6...00	intracranial injury excluding those with skull fracture	
Aurum and GOLD	54441004	90489010	90489010	8646	S314.00	fracture of shaft of femur	
Aurum and GOLD	54530004	1231346013	1231346013	33974	S341.00	open fracture ankle, medial malleolus	
Aurum and GOLD	54556006	90678013	391357011	1073	S23x211	fracture of ulna nos	
Aurum and GOLD	5468008	10127011	560441000006115	50687	S041300	closed #skull/face, mult + intracranial inj, 1-24hrs loc	

Aurum and GOLD	5468008	10127011	560551000006117	96681	S041600	closed #skull/face,mult + intracran inj, loc unspec duration	
Aurum and GOLD	5468008	10127011	696131000006112	73411	S042.00	mult #skull/face + other bones, open, no intracranial injury	
Aurum and GOLD	5468008	10127011	560561000006115	112954	S040z00	closed #skull/face,mult,no intracran inj, concussion unspec	
Aurum and GOLD	5468008	10127011	402830019	46142	S04..00	multiple fractures involving skull or face with other bones	
Aurum and GOLD	5468008	10127011	560541000006119	71866	S041z00	closed #skull/face,mult + intracran inj, concussion unspec	
Aurum and GOLD	5468008	10127011	696151000006117	92354	S040.00	mult #skull/face+other bones, closed, no intracranial injury	
Aurum and GOLD	5468008	10127011	263941000006114	62835	S043000	open #skull/face, mult + intracranial inj, unspec consc	
Aurum and GOLD	5468008	10127011	560511000006118	104696	S040000	closed #skull/face, mult, no intracranial inj, unspec consc	
Aurum and GOLD	5468008	10127011	560501000006116	103132	S040100	closed #skull/face, mult, no intracranial inj, no loss consc	
Aurum and GOLD	5468008	10127011	560461000006116	93804	S041000	closed #skull/face, mult + intracranial inj, unspec consc	
Aurum and GOLD	5468008	10127011	263901000006112	72710	S043400	open #skull/face, mult + intracran inj, >24hr loc + recovery	
Aurum and GOLD	5468008	10127011	695771000006119	50247	S04z.00	multiple fractures involving skull/face with other bones nos	
Aurum and GOLD	5468008	10127011	696121000006114	107041	S043.00	mult #skull/face + other bones, open + intracranial injury	
Aurum and GOLD	5468008	10127011	696141000006119	73206	S041.00	mult #skull/face+other bones, closed + intracranial injury	
Aurum and GOLD	5468008	10127011	560481000006114	72400	S040200	closed #skull/face, mult, no intracranial inj, <1hr loc	
Aurum and GOLD	54819005	91112014	483745010	26324	S232.00	closed fracture of radius and ulna, shaft	
Aurum and GOLD	56863004	94566013	318246018	4978	S02x100	fracture of orbit nos, closed	
Aurum and GOLD	57114005	1231685010	1231685010	26045	S240500	closed fracture trapezium	

Aurum and GOLD	57470004	95576017	207601000006111	33624	7K1D600	prmy open red+int fxn prox femoral #+screw/nail device alone	
Aurum and GOLD	58150001	1231810014	1231810014	4211	S20..11	collar bone fracture	
Aurum and GOLD	58150001	96628016	96628016	483	S20..00	fracture of clavicle	
Aurum and GOLD	58150001	96628016	318734018	28066	S20z.00	fracture of clavicle nos	
Aurum and GOLD	58580000	1231853014	1231853014	6893	S224100	closed fracture distal humerus, supracondylar	
Aurum and GOLD	5913000	10843018	44341000006111	50727	S301000	opn # proximal femur, intracapsular section, unspecified	
Aurum and GOLD	5913000	10843018	10843018	2225	S30..00	fracture of neck of femur	
Aurum and GOLD	5913000	10843018	265421000006113	67633	S303000	open # of proximal femur, trochanteric section, unspecified	
Aurum and GOLD	59556008	1231972010	1231972010	33457	S331100	open fracture proximal fibula	
Aurum and GOLD	59639009	1231985013	1231985013	33656	S330100	closed fracture proximal fibula	
Aurum and GOLD	60667009	100797019	318603011	7831	S120000	closed fracture of rib, unspecified	
Aurum and GOLD	60667009	100797019	318613015	28244	S120z00	closed fracture of rib(s) nos	
Aurum and GOLD	60667009	1232117011	1232117011	280	S120.00	closed fracture rib	
Aurum and GOLD	609411003	2965628019	402851016	45723	S12z.00	fracture of rib(s), sternum, larynx or trachea nos	
Aurum and GOLD	609411003	2965628019	318602018	56961	S12..00	fracture of rib(s), sternum, larynx and trachea	
Aurum and GOLD	61202000	1494827019	1494827019	104931	S023600	open fracture of mandible, symphysis of body	
Aurum and GOLD	62356006	1232322015	1232322015	31760	S225100	open fracture distal humerus, supracondylar	
Aurum and GOLD	63669006	1232490018	749201000006118	44343	S02A.00	le fort i fracture maxilla	
Aurum and GOLD	64455005	107139013	107139013	9072	S10B400	fracture of acetabulum	
Aurum and GOLD	64569008	107298010	253061000006117	37291	S3x0.00	other, multiple and ill-defined closed fractures lower limb	
Aurum and GOLD	64665009	107463010	107463010	8276	S350.00	closed fracture of calcaneus	
Aurum and GOLD	649002	2150010	318877014	49796	S235500	open fracture distal ulna - other	
Aurum and GOLD	65354004	108587012	792591000006110	54353	S12X.00	fracture of bony thorax, part unspecified	
Aurum and GOLD	65354004	108587012	318720018	63242	S14..00	fracture of ill-defined bones of trunk	
Aurum and GOLD	65354004	108587012	318723016	57981	S14z.00	fracture of ill-defined bone of trunk nos	

Aurum and GOLD	65354004	108587012	325342016	111042	Syu2800	[x]fracture of bony thorax, part unspecified	
Aurum and GOLD	65752003	109229016	563311000006118	41730	S022200	closed fracture of mandible, subcondylar	
Aurum and GOLD	65775005	109271015	318828018	55201	S231z00	open fracture of forearm, upper end, nos	
Aurum and GOLD	65775005	109271015	318821012	50223	S231000	open fracture of proximal forearm, unspecified	
Aurum and GOLD	65775005	1495384010	1495384010	37875	S231A00	open fracture radius and ulna, proximal	
Aurum and GOLD	65775005	500216016	500216016	45695	S231.00	open fracture of proximal radius and ulna	
Aurum and GOLD	65966004	109556018	325400017	52977	Syu5400	[x]fracture of forearm, unspecified	
Aurum and GOLD	65966004	109556018	318868019	60630	S235000	open fracture of forearm, lower end, unspecified	
Aurum and GOLD	65966004	109556018	500272010	10640	S23..11	forearm fracture	
Aurum and GOLD	65966004	109556018	325399012	102916	Syu5300	[x]fracture of other parts of forearm	
Aurum and GOLD	66112004	1232763011	1232763011	3983	S122.00	closed fracture sternum	
Aurum and GOLD	66246007	1232779012	1232779012	66233	S233100	open fracture of the radial shaft	
Aurum and GOLD	6628008	1232781014	1232781014	10095	S311100	open fracture shaft of femur	
Aurum and GOLD	66308002	110116013	110116013	517	S22..00	fracture of humerus	
Aurum and GOLD	66308002	110116013	318810015	10382	S22z.00	fracture of humerus nos	
Aurum and GOLD	66308002	110116013	318775013	61378	S222z00	closed fracture of humerus, shaft or unspecified part nos	
Aurum and GOLD	66308002	110116013	318779019	73426	S223z00	open fracture of humerus, shaft or unspecified part nos	
Aurum and GOLD	66926007	111159013	319160016	6320	S312100	closed fracture of femoral condyle, unspecified	
Aurum and GOLD	66973001	1232866011	1232866011	108242	S353600	open fracture lateral cuneiform	
Aurum and GOLD	6698000	1232867019	1232867019	10009	S346.00	closed fracture ankle, trimalleolar	
Aurum and GOLD	67130000	1232881017	1232881017	56311	S241500	open fracture trapezium	
Aurum and GOLD	67422008	1232935018	1232935018	34954	S352400	closed fracture medial cuneiform	
Aurum and GOLD	68076002	1233006016	1233006016	17286	S241100	open fracture of the scaphoid	
Aurum and GOLD	68819003	1233086019	1233086019	17822	S230200	closed fracture of ulna, coronoid	
Aurum and GOLD	68854005	1233093015	1233093015	7009	S230600	closed fracture radius, head	
Aurum and GOLD	69029002	114663015	114663015	73956	S121700	open fracture of seven ribs	
Aurum and GOLD	69166006	114882014	564031000006111	42978	S330z00	closed fracture of tibia and fibula, proximal nos	

Aurum and GOLD	69427008	1233163011	1233163011	7636	S231600	open fracture radial head	
Aurum and GOLD	69866009	116029016	560401000006117	62955	S000100	closed #skull vlt no intracranial injury, no loss of consc	
Aurum and GOLD	69866009	116029016	560411000006119	65724	S000000	closed #skull vlt no intracranial injury, unspec state consc	
Aurum and GOLD	69866009	116029016	560351000006113	52871	S000500	closed #skull vlt no intracranial inj,>24hr loc not restored	
Aurum and GOLD	69866009	116029016	560391000006119	66765	S000300	closed #skull vlt no intracranial injury, 1-24hr loss consc	
Aurum and GOLD	69866009	116029016	560371000006115	50524	S000200	closed #skull vlt no intracranial injury, <1hr loss of consc	
Aurum and GOLD	69866009	116029016	565191000006110	47842	S000.00	closed fracture vault of skull without intracranial injury	
Aurum and GOLD	69866009	116029016	560381000006117	113103	S000400	closed #skull vlt no intracranial injury, >24hr loc+recovery	
Aurum and GOLD	69866009	116029016	560361000006110	73430	S000z00	closed #skull vlt no intracranial injury + concussion unspec	
Aurum and GOLD	69866009	116029016	560341000006111	93438	S000600	closed #skull vlt no intracranial inj, loc unspec duration	
Aurum and GOLD	6990005	12536011	12536011	7723	S337.00	fracture of shaft of tibia	
Aurum and GOLD	70060006	2792577018	567411000006118	7339	7K1LA00	closed reduction of fracture of toe	
Aurum and GOLD	70590006	1233283015	1233283015	34632	7J02200	elevation of depressed fracture of cranium	
Aurum and GOLD	708898008	3037770015	557961000006116	53670	7K1J011	cl red intracaps frac neck femur fix-garden cannulated screw	
Aurum and GOLD	708898008	3037770015	570621000006118	8719	7K1J000	cls red+int fxn proximal femoral #+screw/nail device alone	
Aurum and GOLD	71039006	1233343014	1233343014	17956	S201200	open fracture clavicle, shaft	
Aurum and GOLD	7108004	12725019	276867016	72908	7K15y00	other specified other surgical fracture of bone	
Aurum and GOLD	7108004	12725019	276868014	33357	7K15z00	other surgical fracture of bone nos	
Aurum and GOLD	7108004	12725019	276865012	7393	7K15.00	other surgical fracture of bone	
Aurum and GOLD	71139009	1233358018	1233358018	33883	S230900	closed fracture of the proximal radius	
Aurum and GOLD	71220005	118266012	118266012	50749	S133.00	open fracture of pubis	

Aurum and GOLD	71456006	118712017	562361000006111	40394	S125000	closed fracture larynx	
Aurum and GOLD	71555008	118879014	402864015	24621	S23x200	closed fracture of ulna (alone), unspecified	
Aurum and GOLD	71620000	118977010	319152017	52318	S310z00	closed fracture of shaft or unspecified part, nos	
Aurum and GOLD	71620000	118977010	325439014	73113	Syu7200	[x]fractures of other parts of femur	
Aurum and GOLD	71620000	118977010	391382011	20893	S310012	upper leg fracture nos	
Aurum and GOLD	71620000	118977010	319146018	8040	S31..00	other fracture of femur	
Aurum and GOLD	71620000	118977010	319155015	94360	S311z00	open fracture of femur, shaft or unspecified part, nos	
Aurum and GOLD	71620000	118977010	319153010	42972	S311.00	open fracture of femur, shaft or unspecified part	
Aurum and GOLD	71620000	118977010	105741000006114	12791	S310011	thigh fracture nos	
Aurum and GOLD	71620000	118977010	319154016	34106	S311000	open fracture of femur, unspecified part	
Aurum and GOLD	71620000	118978017	319180015	520	S31z.00	fracture of femur, nos	
Aurum and GOLD	71642004	119023019	402829012	57328	S03z.00	skull fracture nos	
Aurum and GOLD	71642004	119023019	318343012	33692	S0z..00	fracture of skull nos	
Aurum and GOLD	71642004	119023019	563791000006110	57644	S030.00	closed fracture of skull nos without intracranial injury	
Aurum and GOLD	71642004	119023019	318256019	61388	S03..00	other and unqualified skull fractures	
Aurum and GOLD	71642004	119023019	119023019	721	S0...00	fracture of skull	
Aurum and GOLD	71642004	119023019	613741000006112	23780	S03z.11	depressed skull fracture nos	
Aurum and GOLD	72224003	120008015	259321000006112	52305	S251300	open fracture finger metacarpal shaft	
Aurum and GOLD	72435006	1233503017	1233503017	68556	S201300	open fracture clavicle, lateral end	
Aurum and GOLD	72497001	1233512015	559861000006119	34286	S230711	closed # radius neck	
Aurum and GOLD	72497001	1233512015	1233512015	7660	S230700	closed fracture radius, neck	
Aurum and GOLD	73015009	1233578010	1233578010	40992	S353200	open fracture navicular	
Aurum and GOLD	74395007	1233739013	258091000006115	37865	S361.00	open fracture of one or more phalanges of foot	
Aurum and GOLD	75308009	1233864019	1233864019	11635	S352200	closed fracture navicular	
Aurum and GOLD	75591007	125543012	792711000006112	806	S339.00	fracture of fibula alone	
Aurum and GOLD	75591007	125543012	319247018	28352	S33y100	open fracture of fibula, unspecified part, nos	
Aurum and GOLD	75857000	125993015	125993015	6825	S23..00	fracture of radius and ulna	
Aurum and GOLD	75857000	125994014	318915012	909	S23z.00	fracture of radius and ulna, nos	

Aurum and GOLD	75986001	126211018	42991000006113	23845	7K6F300	open reduction of dislocation of jt+fxn of joint, unspec	
Aurum and GOLD	76174009	2478835016	2478835016	57190	S022700	closed fracture of mandible, alveolar border of body	
Aurum and GOLD	76542006	127115011	794941000006115	41675	S01..13	frontal sinus fracture	
Aurum and GOLD	76865005	1234036019	1234036019	29117	S260R00	closed fracture finger distal phalanx	
Aurum and GOLD	7687006	13705013	13705013	27922	S10B300	fracture of ilium	
Aurum and GOLD	7687006	13705013	318699015	96984	S135000	open fracture of ilium, unspecified	
Aurum and GOLD	7687006	13705013	318688017	40643	S134000	closed fracture of ilium, unspecified	
Aurum and GOLD	76911006	1495445016	1495445016	108000	S135100	open fracture pelvis, ischium	
Aurum and GOLD	769141000000107	1716471000000115	563401000006113	43153	S352z00	closed fracture of one or more tarsal + metatarsal bones nos	
Aurum and GOLD	76974008	503422014	503422014	8199	S264.00	multiple fractures of fingers	
Aurum and GOLD	77493009	128645011	318709018	99203	S135y00	other open fracture of pelvis	
Aurum and GOLD	77493009	128645011	318719012	64139	S13z.00	open fracture of pelvis nos	
Aurum and GOLD	77493009	128645011	318687010	33961	S134.00	other or multiple closed fracture of pelvis	
Aurum and GOLD	77493009	128645011	253131000006110	35018	S135z00	other/multiple open fracture of pelvis nos	
Aurum and GOLD	77551005	1234127016	1234127016	55939	S353700	open fracture metatarsal	
Aurum and GOLD	77598002	1234136017	1234136017	53566	S131.00	open fracture acetabulum	
Aurum and GOLD	77598002	128798015	318672017	64777	S131z00	open fracture acetabulum nos	
Aurum and GOLD	77598002	128798015	318671012	62562	S131y00	other specified open fracture acetabulum	
Aurum and GOLD	77803008	1234168013	1234168013	28426	S332100	closed fracture shaft of fibula	
Aurum and GOLD	78011002	129465015	129465015	36249	S124.00	flail chest	
Aurum and GOLD	78490005	1234261013	1234261013	61374	S231500	open fracture of the proximal ulna	
Aurum and GOLD	78516000	130281012	130281012	11969	S128.00	fracture of sternum	
Aurum and GOLD	78516000	130284016	391373017	27818	S12z.12	sternum fracture nos	
Aurum and GOLD	78980006	131054012	318893017	66774	S235z00	open fracture of forearm, lower end, nos	
Aurum and GOLD	7913001	1234315010	1234315010	51666	S241300	open fracture triquetral	
Aurum and GOLD	79546008	131972016	131972016	56384	S120300	closed fracture of three ribs	
Aurum and GOLD	79626009	132103012	132103012	30611	S352111	closed fracture of astragalus	
Aurum and GOLD	79626009	132104018	132104018	15079	S352100	closed fracture of talus	

Aurum and GOLD	79978005	1234424019	1234424019	42805	S313300	open fracture distal femur, supracondylar	
Aurum and GOLD	80411001	133435011	133435011	6074	S235100	open colles' fracture	
Aurum and GOLD	80756009	133953010	563861000006112	33393	S320.00	closed fracture of the patella	
Aurum and GOLD	81576005	1234601017	563371000006110	4306	S360.00	closed fracture of one or more phalanges of foot	
Aurum and GOLD	81639003	1234611012	1234611012	9771	S020.11	closed fracture nasal bone	
Aurum and GOLD	81639003	1234612017	1234612017	417	S020.00	closed fracture nose	
Aurum and GOLD	81966000	135964010	318914011	65301	S23yz00	open fracture of radius and ulna, nos	
Aurum and GOLD	81966000	135964010	257831000006114	8704	S23y300	open fracture of the radius and ulna	
Aurum and GOLD	81966000	135964010	318910019	54780	S23y.00	open fracture of radius and ulna, unspecified part	
Aurum and GOLD	82065001	1234676011	1234676011	10167	S24..11	hand fracture - carpal bone	
Aurum and GOLD	82065001	136121013	325417012	102155	Syu6300	[x]fracture of other carpal bone(s)	
Aurum and GOLD	82065001	136121013	318940017	43792	S24z.00	fracture of carpal bone nos	
Aurum and GOLD	82065001	136121013	136121013	22375	S24..00	fracture of carpal bone	
Aurum and GOLD	82419009	136707019	318494016	66164	S10y.00	open fracture of spine, unspecified,	
Aurum and GOLD	8303001	14689016	531681000006111	33503	S100611	c6 vertebra closed fracture without spinal cord lesion	
Aurum and GOLD	83385002	138306017	244171000006112	8573	S00..12	parietal bone fracture	
Aurum and GOLD	83969004	139245018	108621000006117	5567	S01..19	temporal bone fracture	
Aurum and GOLD	84111004	139472011	256651000006118	12462	S025100	open fracture zygoma	
Aurum and GOLD	84138006	139537010	451464018	38728	N331D00	collapsed vertebra nos	
Aurum and GOLD	84138006	139537010	359834019	2793	N331.12	collapse of vertebra nos	
Aurum and GOLD	849671000000101	2201831000000117	318890019	53698	S235D00	open fracture distal radius, extra-articular other type	
Aurum and GOLD	849681000000104	2201851000000112	318891015	63588	S235E00	open fracture distal radius, intra-articular other type	
Aurum and GOLD	852231000000109	2207421000000112	1843951000006113	105803	7K1DE00	prim op red frac neck fem op fix us prox fem nail antirotatn	
Aurum and GOLD	86052008	142673013	277223016	70919	7K1Ly00	other specified other closed reduction of fracture of bone	
Aurum and GOLD	86052008	142673013	166681000006118	67796	7K1L200	revision to closed reduction of # and skeletal traction nec	History

Aurum and GOLD	86052008	142673013	1221611016	104067	7K1L211	remanipulation of fracture and skeletal traction nec	History
Aurum and GOLD	86052008	142673013	209771000006118	48139	7K1L000	primary closed reduction of # and skeletal traction nec	
Aurum and GOLD	86052008	142673013	277224010	15085	7K1Lz00	other closed reduction of fracture of bone nos	
Aurum and GOLD	86052008	142673013	142673013	10737	82...11	closed reduction of fracture	
Aurum and GOLD	86052008	142673013	719361000006115	55308	7K1L011	manipulation of fracture and skeletal traction nec	
Aurum and GOLD	86052008	142673013	277211010	35052	7K1LX00	revision to closed reduction of fracture alone	History
Aurum and GOLD	86052008	142673013	277180014	4629	7K1L.00	other closed reduction of fracture of bone	
Aurum and GOLD	86052008	142673013	277209018	11872	7K1LV00	primary closed reduction of fracture alone	
Aurum and GOLD	86052008	142673013	277184017	30213	7K1L300	remanipulation of fracture of bone nec	History
Aurum and GOLD	86052008	142675018	277182018	2887	7K1L100	manipulation of fracture of bone nec	
Aurum and GOLD	86357008	1235130019	1235130019	52614	S231200	open fracture of ulna, coronoid	
Aurum and GOLD	86480004	143414012	277057016	40321	7K1G300	primary open reduction of fracture alone	
Aurum and GOLD	86480004	143414012	277081011	57626	7K1H900	revision to open reduction of fracture alone	History
Aurum and GOLD	86480004	143414012	277053017	4528	7K1G.00	other primary open reduction of fracture of bone	
Aurum and GOLD	86704005	143783013	486231000006119	59798	S01..11	anterior fossa fracture	
Aurum and GOLD	86731008	143833013	318701015	72567	S135200	open multiple disruptions of pelvis	
Aurum and GOLD	86844009	144023010	318772011	45275	S221z00	open fracture of proximal humerus not otherwise specified	
Aurum and GOLD	86844009	144023010	257781000006117	9420	S221.00	open fracture of the proximal humerus	
Aurum and GOLD	86844009	144023010	318762013	53688	S221000	open fracture of proximal humerus, unspecified part	
Aurum and GOLD	87225004	1235211014	1235211014	63982	S123.00	open fracture sternum	
Aurum and GOLD	87376003	1235228012	1235228012	28307	S200200	closed fracture clavicle, shaft	
Aurum and GOLD	87804006	145572015	531661000006118	34873	S100511	c5 vertebra closed fracture without spinal cord lesion	
Aurum and GOLD	88116004	1495502013	1495502013	10022	S235.11	wrist fracture - open	
Aurum and GOLD	88116004	1495503015	1495503015	38398	S235600	open fracture radius and ulna, distal	
Aurum and GOLD	88116004	507904017	507904017	27590	S235.00	open fracture of radius and ulna, lower end	

Aurum and GOLD	8840000	15592018	570811000006110	62337	S110000	cls spinal fracture with unspec cervical cord lesion, c1-4	
Aurum and GOLD	88835002	147293015	147293015	32638	S125200	closed fracture of thyroid cartilage	
Aurum and GOLD	89161006	1235429018	1235429018	44156	S241400	open fracture pisiform	
Aurum and GOLD	89294002	148070018	318777017	33680	S223.00	open fracture of humerus, shaft or unspecified part	
Aurum and GOLD	89294002	148070018	318778010	48961	S223000	open fracture of humerus nos	
Aurum and GOLD	89636007	148612017	148612017	65484	S120600	closed fracture of six ribs	
Aurum and GOLD	89820008	148902010	562981000006117	8648	S302400	closed fracture of femur, intertrochanteric	
Aurum and GOLD	90114000	1235538018	1235538018	17606	S240700	closed fracture capitate	
Aurum and GOLD	90235006	1235549012	1235549012	27886	S222100	closed fracture of humerus, shaft	
Aurum and GOLD	90338005	1235556018	1235556018	9917	S347.00	open fracture ankle, trimalleolar	
Aurum and GOLD	90863004	150580019	150580019	68652	S120800	closed fracture of eight or more ribs	
Aurum and GOLD	91037003	150859016	318718016	28375	S13y.00	closed fracture of pelvis nos	
Aurum and GOLD	91296001	151267013	318911015	70590	S23y000	open fracture of forearm, unspecified	
Aurum and GOLD	91419009	151450016	318902015	50654	S23x000	closed fracture of forearm, unspecified	
Aurum and GOLD	9217002	509878013	253461000006114	30186	7403600	outfracture of turbinates of nose	
Aurum and GOLD	9217002	511731000000116	511731000000116	89236	7403900	surgical outfracture of turbinate of nose	
Aurum and GOLD	9275003	510090013	510090013	4725	S242300	multiple fractures of metacarpal bones	
Aurum and GOLD	9344009	16393016	257921000006119	40069	S331.00	open fracture of tibia and fibula, proximal	
Aurum and GOLD	9344009	16393016	319219017	57439	S331z00	open fracture of tibia and fibula, proximal nos	
Aurum and GOLD	9344009	16393016	257931000006116	54145	S331200	open fracture of tibia and fibula, proximal	
Aurum and GOLD	9468002	16583010	54541000006116	203	S234.11	wrist fracture - closed	
Aurum and GOLD	9468002	511384012	318916013	57979	S240000	closed fracture of carpal bone, unspecified	
Aurum and GOLD	9468002	511384012	318924015	56886	S240y00	closed fracture of other carpal bone	
Aurum and GOLD	9468002	511384012	318925019	33929	S240z00	closed fracture of carpal bone nos	
Aurum and GOLD	9468002	511384012	511384012	15666	S240.00	closed fracture of carpal bone	
Aurum and GOLD	95851007	158762014	318250013	31153	S02y100	fracture of orbit nos, open	
Aurum and GOLD	9682006	16926014	16926014	1177	S21..00	fracture of scapula	
Aurum and GOLD	9682006	16926014	318749014	33870	S21z.00	fracture of scapula nos	

Aurum and GOLD	9682006	16926014	140571000006110	10735	S21..11	shoulder blade fracture	
Aurum and GOLD	9808005	1235997015	1235997015	4310	S352300	closed fracture cuboid	

Code list for pneumonia – ICD10

ICD10	Description
J10.0	Influenza due to other identified influenza virus with pneumonia
J11.0	Influenza due to unidentified influenza virus with pneumonia
J12	Viral pneumonia, not elsewhere classified
J13	Pneumonia due to streptococcus pneumoniae
J14	Pneumonia due to haemophilus influenzae
J15	Bacterial pneumonias, not elsewhere classified
J15.0	Pneumonia due to klebsiella pneumoniae
J15.1	Pneumonia due to pseudomonas
J15.2	Pneumonia due to staphylococcus
J15.3	Pneumonia due to streptococcus group B
J15.4	Pneumonia due to other streptococci
J15.5	Pneumonia due to e coli
J15.6	Pneumonia due to other gram-negative bacteria
J15.8	Other bacterial pneumia
J15.9	Bacterial pneumonia, unspecified
J16	Pneumonia due to other infectious organisms, not elsewhere classified
J16.0	Chlamydial pneumonia
J16.8	Pneumonia due to other specified infectious organisms
J17.0	Pneumonia in bacterail diseases classified elsewhere
J18	Pneumonia, organism unspecified
J18.0	Bronchonpneumonia, unspecified
J18.1	Lobar pneumonia, unspecifed
J18.8	Other pneumonia, organism unspecified
J18.9	Pneumonia, unspecified
J85.1	Abscess of lung with pneumonia

Code list for acute kidney injury (AKI) – ICD10

ICD10	Description
N17	Acute renal failure
N17.0	Acute renal failure with tubular necrosis
N17.1	Acute renal failure with acute cortical necrosis
N17.2	Acute renal failure with medullary necrosis
N17.8	Other acute renal failure
N17.9	Acute renal failure, unspecified

Code list for appendicitis and cholecystitis (negative control outcome) – ICD10

ICD10	Description
K35	Acute appendicitis
K35.2	Acute appendicitis with generalized peritonitis
K35.3	Acute appendicitis with localized peritonitis
K35.8	Acute appendicitis, other and unspecified
K37	Unspecified appendicitis
K81	Cholecystitis
K81.0	Acute cholecystitis
K81.9	Cholecystitis, unspecified

Code list for appendicitis and cholecystitis (negative control outcome) – Aurum and GOLD

Source	SNOMED_CT_Concept_id	SNOMED_CT_Description_id	Medcode_Aurum	Medcode_GOLD	Readcode	Term	History_only	type
Aurum and GOLD	161532008	251727010	251727010	7210	14C2.00	H/O: appendicitis	History	Appendicitis
Aurum and GOLD	174036004	269573010	269573010	10830	7700.00	Emergency excision of appendix		Appendicitis
Aurum and GOLD	174036004	269573010	640031000006115	23344	7700000	Emergency excision of abnormal appendix and drainage HFQ		Appendicitis
Aurum and GOLD	174036004	269573010	269582016	33826	7700z00	Emergency excision of appendix NOS		Appendicitis
Aurum and GOLD	174036004	269573010	269581011	60360	7700y00	Other specified emergency excision of appendix		Appendicitis
Aurum and GOLD	174036004	269574016	269578018	7832	7700300	Emergency appendicectomy NEC		Appendicitis
Aurum and GOLD	174036004	269574016	269576019	33538	7700100	Emergency appendectomy		Appendicitis
Aurum and GOLD	174036004	269574016	269574016	3141	7700.11	Emergency appendicectomy		Appendicitis
Aurum and GOLD	174041007	269580012	269580012	9206	7700400	Endoscopic emergency appendicectomy		Appendicitis
Aurum and GOLD	174052001	269595014	628511000006117	2394	7702000	Drainage of abscess of appendix		Appendicitis
Aurum and GOLD	196781001	302683013	302683013	1999	J200.00	Acute appendicitis with peritonitis		Appendicitis
Aurum and GOLD	266439004	396348018	396348018	16040	J201.00	Acute appendicitis with appendix abscess		Appendicitis
Aurum and GOLD	28845006	483992016	483992016	104496	J203.00	Acute appendicitis with generalised peritonitis		Appendicitis
Aurum and GOLD	307582003	450880012	269597018	29890	7702100	Drainage of appendix		Appendicitis
Aurum and GOLD	50846009	84731010	84731010	17014	J20z100	Acute gangrenous appendicitis		Appendicitis
Aurum and GOLD	6025007	1207798017	269589013	28587	7701400	Laparoscopic appendicectomy		Appendicitis

Aurum and GOLD	62224002	1232298010	221031000000117	4130	J201.12	Appendiceal abscess		Appendicitis
Aurum and GOLD	6503008	11785019	11785019	3472	J222.00	Relapsing appendicitis	History	Appendicitis
Aurum and GOLD	67365005	111955018	111955018	15393	J223.00	Recurrent appendicitis	History	Appendicitis
Aurum and GOLD	698294004	2973821014	2116831000000110	104722	J204.00	Acute appendicitis with localised peritonitis		Appendicitis
Aurum and GOLD	72048003	119720012	119720012	29882	J202.00	Acute appendicitis without peritonitis		Appendicitis
Aurum and GOLD	74400008	123558018	303753017	52383	Jyu2000	[X]Other appendicitis		Appendicitis
Aurum and GOLD	74400008	123558018	302691016	35406	J22..00	Other appendicitis		Appendicitis
Aurum and GOLD	74400008	123558018	302690015	25856	J21..00	Appendicitis		Appendicitis
Aurum and GOLD	74400008	123558018	302692011	37817	J22z.00	Other appendicitis NOS		Appendicitis
Aurum and GOLD	80146002	132973012	445492014	422	7701z11	Appendicectomy		Appendicitis
Aurum and GOLD	85189001	141235016	141235016	698	J20..00	Acute appendicitis		Appendicitis
Aurum and GOLD	85189001	141235016	302689012	15490	J20z.00	Acute appendicitis NOS		Appendicitis
Aurum only	123601005	192090017	4353611000006111			Focal appendicitis		Appendicitis
Aurum only	161532008	2986802017	4540811000006118			History of appendicitis	History	Appendicitis
Aurum only	174036004	1207867012	11924541000006112			Emergency appendectomy		Appendicitis
Aurum only	174041007	1207869010	4659621000006112			Laparoscopic emergency appendectomy		Appendicitis
Aurum only	174041007	2475171016	4659631000006110			Endoscopic emergency appendectomy		Appendicitis
Aurum only	174041007	269579014	4659601000006119			Laparoscopic emergency appendicectomy		Appendicitis
Aurum only	174052001	269595014	4659661000006118			Drainage of appendix abscess		Appendicitis
Aurum only	174052001	863541000006110	863541000006110		7702099	Appendix abscess drainage		Appendicitis
Aurum only	235769005	353434016	5085321000006110			Acute focal appendicitis		Appendicitis

Aurum only	235770006	353435015	5085331000006113			Acute suppurative appendicitis		Appendicitis
Aurum only	25598004	42911019	2909461000006115			Retrocecal appendicitis		Appendicitis
Aurum only	266439004	886101000006110	886101000006110		J201.99	Acute appendic abscess		Appendicitis
Aurum only	26826005	44894011	2929611000006112			Amebic appendicitis		Appendicitis
Aurum only	28358004	47474016	2955341000006117			Acute obstructive appendicitis with perforation AND peritonitis		Appendicitis
Aurum only	286967008	426465018	5721251000006115			Acute perforated appendicitis		Appendicitis
Aurum only	286967008	886091000006116	886091000006116		J200.99	Acute perforated appendicitis		Appendicitis
Aurum only	28845006	48298010	2963741000006113			Acute appendicitis with generalized peritonitis		Appendicitis
Aurum only	37402004	62393016	3102631000006110			Incision and drainage of abscess of appendix		Appendicitis
Aurum only	42640003	71132016	3187371000006116			Suppurative appendicitis		Appendicitis
Aurum only	42640003	71133014	3187381000006118			Purulent appendicitis		Appendicitis
Aurum only	428251008	2694784013	7085631000006119			History of appendectomy	History	Appendicitis
Aurum only	428251008	2695558013	7085621000006117			History of appendicectomy	History	Appendicitis
Aurum only	443037006	2841715018	7271031000006114			Percutaneous drainage of abscess of appendix using computed tomography guidance		Appendicitis
Aurum only	47693006	79478018	3271191000006115			Appendicitis with perforation		Appendicitis
Aurum only	49438003	1230727018	3299971000006113			Appendicectomy and drainage		Appendicitis
Aurum only	49438003	2475355011	3299981000006111			Appendectomy and drainage		Appendicitis
Aurum only	4998000	9373011	2579211000006119			Acute obstructive appendicitis		Appendicitis

Aurum only	51036000	85038019	3327001000006110			Acute appendicitis with peritoneal abscess		Appendicitis
Aurum only	528801000000102	1181341000000116	12718661000006119			Appendicitis, unqualified		Appendicitis
Aurum only	528801000000102	989161000006111	989161000006111		J21..99	Appendicitis		Appendicitis
Aurum only	528801000000102	990761000006117	990761000006117		J21..98	Appendicitis NOS		Appendicitis
Aurum only	5596004	10337012	2588761000006113			Atypical appendicitis		Appendicitis
Aurum only	591681000000105	1311881000000111	12721851000006117			Appendicitis and other disorders of the appendix		Appendicitis
Aurum only	591681000000105	886081000006119	886081000006119		J2...99	Appendicitis		Appendicitis
Aurum only	591751000000108	886131000006119	886131000006119		J2z..99	Appendicitis NOS		Appendicitis
Aurum only	6025007	11010012	2595751000006115			Laparoscopic appendectomy		Appendicitis
Aurum only	6025007	1232064013	2595761000006118			Endoscopic appendicectomy		Appendicitis
Aurum only	6025007	2475356012	2595771000006113			Endoscopic appendectomy		Appendicitis
Aurum only	641671000000105	1412821000000114	12726771000006113			Other appendicitis		Appendicitis
Aurum only	641671000000105	886111000006113	886111000006113		J22..99	Appendicitis NOS		Appendicitis
Aurum only	64252005	106804019	3544911000006115			Acute gangrenous appendicitis with perforation AND peritonitis		Appendicitis
Aurum only	64994000	108029018	3557151000006111			Acute fulminating appendicitis with perforation AND peritonitis		Appendicitis
Aurum only	661131000000108	863521000006115	863521000006115		7701.99	Appendicectomy		Appendicitis
Aurum only	698294004	2973833016	7507281000006111			Acute appendicitis with localized peritonitis		Appendicitis
Aurum only	72048003	119721011	3672121000006112			Inflamed acute appendicitis without peritonitis		Appendicitis
Aurum only	735591005	3525028015	3525028015			Acute phlegmonous appendicitis		Appendicitis
Aurum only	84534001	140165012	3875101000006116			Acute fulminating appendicitis		Appendicitis

Aurum only	8744003	15421019	2639581000006119			Catarrhal appendicitis		Appendicitis
Aurum only	906021000006106	906021000006110	906021000006110			[RFC] Appendicitis		Appendicitis
Aurum only	91313006	151301014	3983631000006118			Pelvic appendicitis		Appendicitis
Aurum only	91313006	2672518010	3983641000006111			Appendicitis of a pelvic appendix		Appendicitis
Aurum only	95547004	158257012	4058361000006110			Ruptured suppurative appendicitis		Appendicitis
Aurum only	95547004	158258019	4058371000006115			Ruptured purulent appendicitis		Appendicitis
Aurum and GOLD	197377009	303541018	797961000006111	63208	J640000	Gallbladder calculus with acute cholecystitis and no obstruction		Cholecystitis
Aurum and GOLD	197378004	303542013	797951000006114	48587	J640100	Gallbladder calculus with acute cholecystitis and obstruction		Cholecystitis
Aurum and GOLD	197389006	303553019	303553019	34683	J643.00	Bile duct calculus with acute cholecystitis		Cholecystitis
Aurum and GOLD	197389006	303553019	511631000006119	96679	J643z00	Bile duct calculus + acute cholecystitis - obstruct NOS		Cholecystitis
Aurum and GOLD	197390002	303554013	511641000006112	39025	J643000	Bile duct calculus with acute cholecystitis and no obstruction		Cholecystitis
Aurum and GOLD	197391003	303555014	511651000006114	31650	J643100	Bile duct calculus + acute cholecystitis and obstruction		Cholecystitis
Aurum and GOLD	197410004	303579011	303579011	72904	J650100	Acute angiocholecystitis		Cholecystitis
Aurum and GOLD	235919008	353656010	798011000006111	71134	J641z00	Gallbladder calculus with other cholecystitis - obstruct NOS		Cholecystitis
Aurum and GOLD	235919008	353656010	797991000006115	47536	J641100	Gallbladder calculus with other cholecystitis + obstruct		Cholecystitis

Aurum and GOLD	235919008	353656010	798001000006113	49202	J641000	Gallbladder calculus with other cholecystitis +no obstruct		Cholecystitis
Aurum and GOLD	25924004	43438012	303544014	49106	J641.00	Calculus of gallbladder with cholecystitis		Cholecystitis
Aurum and GOLD	30093007	50351011	303557018	26889	J644.00	Bile duct calculus with other cholecystitis		Cholecystitis
Aurum and GOLD	30093007	50351011	511681000006118	44649	J644100	Bile duct calculus + other cholecystitis and obstruction		Cholecystitis
Aurum and GOLD	30093007	50351011	511661000006111	61298	J644z00	Bile duct calculus + other cholecystitis - obstruction NOS		Cholecystitis
Aurum and GOLD	30093007	50351011	511671000006116	107004	J644000	Bile duct calculus + other cholecystitis and no obstruction		Cholecystitis
Aurum and GOLD	32038009	53540011	53540011	44922	J650.11	Abscess of gallbladder		Cholecystitis
Aurum and GOLD	32067005	53589018	53589018	46248	J650200	Acute emphysematous cholecystitis		Cholecystitis
Aurum and GOLD	428882003	2693757016	2693757016	100084	14ND.00	History of cholecystectomy	History	Cholecystitis
Aurum and GOLD	59771005	1231999012	1231999012	35046	J640.00	Gallbladder calculus with acute cholecystitis		Cholecystitis
Aurum and GOLD	59771005	99287019	797971000006116	64132	J640z00	Gallbladder calculus with acute cholecystitis - obst NOS		Cholecystitis
Aurum and GOLD	65275009	108454010	303580014	15735	J650z00	Acute cholecystitis NOS		Cholecystitis
Aurum and GOLD	65275009	108454010	303578015	44605	J650000	Acute cholecystitis unspecified		Cholecystitis
Aurum and GOLD	65275009	108454010	108454010	505	J650.00	Acute cholecystitis		Cholecystitis
Aurum and GOLD	72533002	120482010	120482010	41018	J650400	Acute gangrenous cholecystitis		Cholecystitis
Aurum and GOLD	73125001	121449018	121449018	932	J650.12	Empyema of gallbladder		Cholecystitis
Aurum and GOLD	76581006	127173011	303581013	45172	J651.00	Other cholecystitis		Cholecystitis

Aurum and GOLD	76581006	127173011	303583011	1924	J651z00	Cholecystitis		Cholecystitis
Aurum and GOLD	76581006	127173011	303790016	101538	Jyu8100	[X]Other cholecystitis		Cholecystitis
Aurum and GOLD	89628003	148601017	148601017	38289	J650300	Acute suppurative cholecystitis		Cholecystitis
Aurum only	19335008	32594019	2806831000006111			Calculus of cystic duct with acute cholecystitis		Cholecystitis
Aurum only	197377009	2475610012	4787661000006112			Cholelithiasis AND acute cholecystitis without obstruction		Cholecystitis
Aurum only	197378004	2475611011	4787681000006119			Cholelithiasis AND acute cholecystitis with obstruction		Cholecystitis
Aurum only	197389006	2475612016	4787701000006116			Calculus of bile duct with acute cholecystitis		Cholecystitis
Aurum only	197391003	303555014	4787721000006114			Bile duct calculus with acute cholecystitis and obstruction		Cholecystitis
Aurum only	19968009	33615015	2817181000006119			Cholecystitis without calculus		Cholecystitis
Aurum only	25924004	43442010	2914881000006114			Biliary calculus with cholecystitis		Cholecystitis
Aurum only	25924004	43443017	2914891000006112			Cholelithiasis with cholecystitis		Cholecystitis
Aurum only	29484002	49344016	2974051000006116			Cholelithiasis AND cholecystitis without obstruction		Cholecystitis
Aurum only	34346002	57335018	3053051000006118			Acute cholecystitis without calculus		Cholecystitis
Aurum only	34346002	57336017	3053061000006116			Acute acalculous cholecystitis		Cholecystitis
Aurum only	36483003	2989913019	3088081000006112			Choledocolithiasis with cholecystitis		Cholecystitis
Aurum only	36483003	60898013	3088071000006114			Calculus of bile duct with cholecystitis		Cholecystitis
Aurum only	38871000119104	3531543012	3531543012			Cholelithiasis and choledocholithiasis with acute on chronic cholecystitis		Cholecystitis

Aurum only	38871000119104	3531546016	3531546016			Acute and chronic cholecystitis co-occurrent and due to calculus of gallbladder and bile duct		Cholecystitis
Aurum only	396335001	1776287011	6567981000006117			Acute and chronic cholecystitis		Cholecystitis
Aurum only	396335001	1785554014	6567991000006119			Acute on chronic cholecystitis		Cholecystitis
Aurum only	40331000119107	3289989019	8016471000006118			Acute cholecystitis due to biliary calculus		Cholecystitis
Aurum only	48413001	495085012	3283261000006113			Acute haemorrhagic cholecystitis		Cholecystitis
Aurum only	48413001	80658019	3283271000006118			Acute hemorrhagic cholecystitis		Cholecystitis
Aurum only	50450007	3028139014	3317031000006113			Gallstone AND cholecystitis with obstruction		Cholecystitis
Aurum only	59771005	99287019	3470731000006110			Calculus of gallbladder with acute cholecystitis		Cholecystitis
Aurum only	59771005	99289016	3470741000006117			Cholelithiasis with acute cholecystitis		Cholecystitis
Aurum only	60127009	99891017	3476471000006116			Calculus of bile duct with acute cholecystitis without obstruction		Cholecystitis
Aurum only	699050007	2982514015	7516391000006115			Calculus of gallbladder with acute and chronic cholecystitis		Cholecystitis
Aurum only	699050007	2982530011	7516401000006118			Cholelithiasis with acute and chronic cholecystitis		Cholecystitis
Aurum only	72053008	119728017	3672161000006118			Calculus of common bile duct with acute cholecystitis without obstruction		Cholecystitis
Aurum only	78754002	130688011	3781071000006116			Angiocholecystitis		Cholecystitis
Aurum only	81115009	134578014	3819341000006114			Calculus of common bile duct with acute		Cholecystitis

						cholecystitis with obstruction		
Aurum only	89251007	147999011	3950771000006114			Calculus of common bile duct with acute cholecystitis		Cholecystitis
Aurum only	89251007	148000017	3950781000006112			Choledocholithiasis with acute cholecystitis		Cholecystitis
Aurum only	95558008	158277017	4058561000006115			Pneumocholecystiti s		Cholecystitis
Aurum only	95558008	201775010	4058591000006111			Gaseous cholecystitis		Cholecystitis
