# Supplementary material for: Multiple adverse outcomes associated with antipsychotic use in people with dementia: population based matched cohort study
Source: BMJ. 2024 Apr 17;385:e076268. doi: 10.1136/bmj-2023-076268 (PMC11022137; doi:10.1136/bmj-2023-076268)
Supplement: Supplementary file 2 — Supplementary material: Additional information, figures S1-S7, and tables S1-S14 [file mokp076268.ww2.pdf]

Appendix

Contents

Defining antipsychotic use as a time varying variable and stratification of follow-up time.............. 3

Propensity score methods to control for potential confounding 5

Supplementary Figure S1. Delineation of CPRD Aurum cohort of patients with a diagnosis of dementia 7

Supplementary Figure S2. Delineation of CPRD GOLD cohort of patients with a diagnosis of dementia 8

Supplementary Figure S3. Graphical depiction of study design 9

Supplementary Figure S4. Hazard ratios (adjusted for IPT weights) of adverse outcomes associated with current, recent, and past antipsychotic use; with current use being defined as the first 60 days from the date of an antipsychotic prescription, recent use as up to 120 days after current use ended, and past use as after recent use. 10

Supplementary Figure S5. Hazard ratios (adjusted for IPT weights) of adverse outcomes associated with current, recent, and past antipsychotic use; with current use being defined as the first 30 days from the date of an antipsychotic prescription, recent use as up to 60 days after current use ended, and past use as after recent use. 11

Supplementary Figure S6. Hazard ratios (adjusted for IPT weights) of adverse outcomes associated with current, recent, and past antipsychotic use excluding levomepromazine, with current use being defined as the first 90 days from the date of an antipsychotic prescription, recent use as up to 180 days after current use ended, and past use as after recent use. 12

Supplementary Figure S7. Subhazard ratios (adjusted for IPT weights) of adverse outcomes associated with current, recent, and past antipsychotic use; with current use being defined as the first 90 days from the date of an antipsychotic prescription, recent use as up to 180 days after current use ended, and past use as after recent use. 13

Supplementary Table S1. Number of prescriptions by antipsychotic drug substance 14

Supplementary Table S2. Baseline characteristics of antipsychotic users and matched comparators included in the analysis of venous thromboembolism (CPRD Aurum and GOLD combined data) .. 15

Supplementary Table S3. Baseline characteristics of antipsychotic users and matched comparators included in the analysis of myocardial infarction (CPRD Aurum and GOLD combined data)........... 17

Supplementary Table S4. Baseline characteristics of antipsychotic users and matched comparators included in the analysis of heart failure (CPRD Aurum and GOLD combined data) 19

Supplementary Table S5. Baseline characteristics of antipsychotic users and matched comparators included in the analysis of ventricular arrhythmia (CPRD Aurum and GOLD combined data)......... 21

Supplementary Table S6. Baseline characteristics of antipsychotic users and matched comparators included in the analysis of fracture (CPRD Aurum and GOLD combined data) 23

Supplementary Table S7. Baseline characteristics of antipsychotic users and matched comparators included in the analysis of pneumonia (CPRD Aurum and GOLD combined data).......................... 25

Supplementary Table S8. Baseline characteristics of antipsychotic users and matched comparators included in the analysis of acute kidney injury (CPRD Aurum and GOLD combined data) 27

Supplementary Table S9. Baseline characteristics of antipsychotic users and matched comparators included in the analysis of unrelated outcomes (appendicitis and cholecystitis, CPRD Aurum and GOLD combined data).. 29

Supplementary Table S10. Hazard ratios (adjusted for IPT weights) of adverse outcomes associated with current, recent, and past use of typical and atypical antipsychotics; with current use being defined as 90 days from the date of an antipsychotic prescription, recent use as up to 180 days after current use ended, and past use as after recent use. 31

Supplementary Table S11. Hazard ratios (adjusted for IPT weights) of adverse outcomes associated with the use of risperidone, quetiapine, haloperidol, and other antipsychotics; with current use being defined as 90 days from the date of an antipsychotic prescription, recent use as up to 180 days after current use ended, and past use as after recent use. 33

Supplementary Table S12. Subhazard ratios (adjusted for IPT weights) of adverse outcomes associated with current, recent, and past antipsychotic use stratified by follow-up period; with current use being defined as the first 90 days from the date of an antipsychotic prescription, recent use as up to 180 days after current use ended, and past use as after recent use. [a]............ 36

Supplementary Table S13. Sex-specific incidence rates of adverse outcomes associated with antipsychotic use during the first 2 years of follow-up period... 37

Supplementary Table S14. Sex-specific hazard ratios (adjusted for IPT weights) of adverse outcomes associated with current, recent, and past antipsychotic use; with current use being defined as the first 90 days from the date of an antipsychotic prescription, recent use as up to 180 days after current use ended, and past use as after recent use. 38

Defining antipsychotic use as a time varying variable and stratification of follow-up time

Exposure to antipsychotics was treated as a time-varying variable, classified as current, recent and past use. For the main analysis, current use was defined as the first 90 days from the date of an antipsychotic prescription, recent use as up to 180 days after current use ended, and past use as the time after the recent use period had ended. Since patients could have multiple prescriptions over time, they could move between the three exposure categories during follow-up, and could therefore be defined as current/recent/past users more than once.

The following example of a fictitious patient illustrates how this is applied. For this fictitious patient, consider if they had a total of five prescriptions of an antipsychotic during the study period, issued on the following dates:
First prescription: 6th July 2006
Second prescription: 7th September 2006
Third prescription: 5th November 2006
Fourth prescription: 13th March 2008
Fifth prescription: 14th April 2008

Follow up of the patient ended on 5th July 2008, i.e. two years after antipsychotics initiation. The status of their antipsychotic exposure during follow-up would be:

Time period		Time gap between T1 and T2	Antipsychotic use status	Note
From (T1)	To (T2)			
6th July 2006 (First prescription, i.e. start of follow-up)	7th September 2006	63 days	Current use	The number of days between the first and second prescription is less than 90 days.
7th September 2006 (Second prescription)	5th November 2006	59 days	Current use	The number of days between the second and third prescription is less than 90 days.
5th November 2006 (Third prescription)	3rd February 2007	90 days	Current use	90 days of current use period started from 5th November 2006 and ended on 3rd February 2007.
3rd February 2007	2nd August 2007	180 days	Recent use	180 days of recent use period beginning after the current use period has ended.
2nd August 2007	13th March 2008	224 days	Past use	Time after the recent use period has ended up to the next prescription.
13th March 2008 (Fourth prescription)	14th April 2008	32 days	Current use	The number of days between the fourth and fifth prescription is less than 90 days.
14th April 2008 (Fifth prescription)	5th July 2008 (End of follow-up)	82 days	Current use	82 days of current use period started from 14th April 2008 and ended on 5th July 2008 when follow-up ended.

The total number of days of follow-up from 6th July 2006 to 5th July 2008 would be 730 days. The figure below shows how the antipsychotic use status of this fictitious patient changes during follow-up, with the total time of follow-up stratified by the following time windows: the first 7 days, 8 to 30 days, 31 to 180 days, 181 to 365 days, and 366 days to 2 years (see 'Statistical analysis' subsection of Methods in the main manuscript).

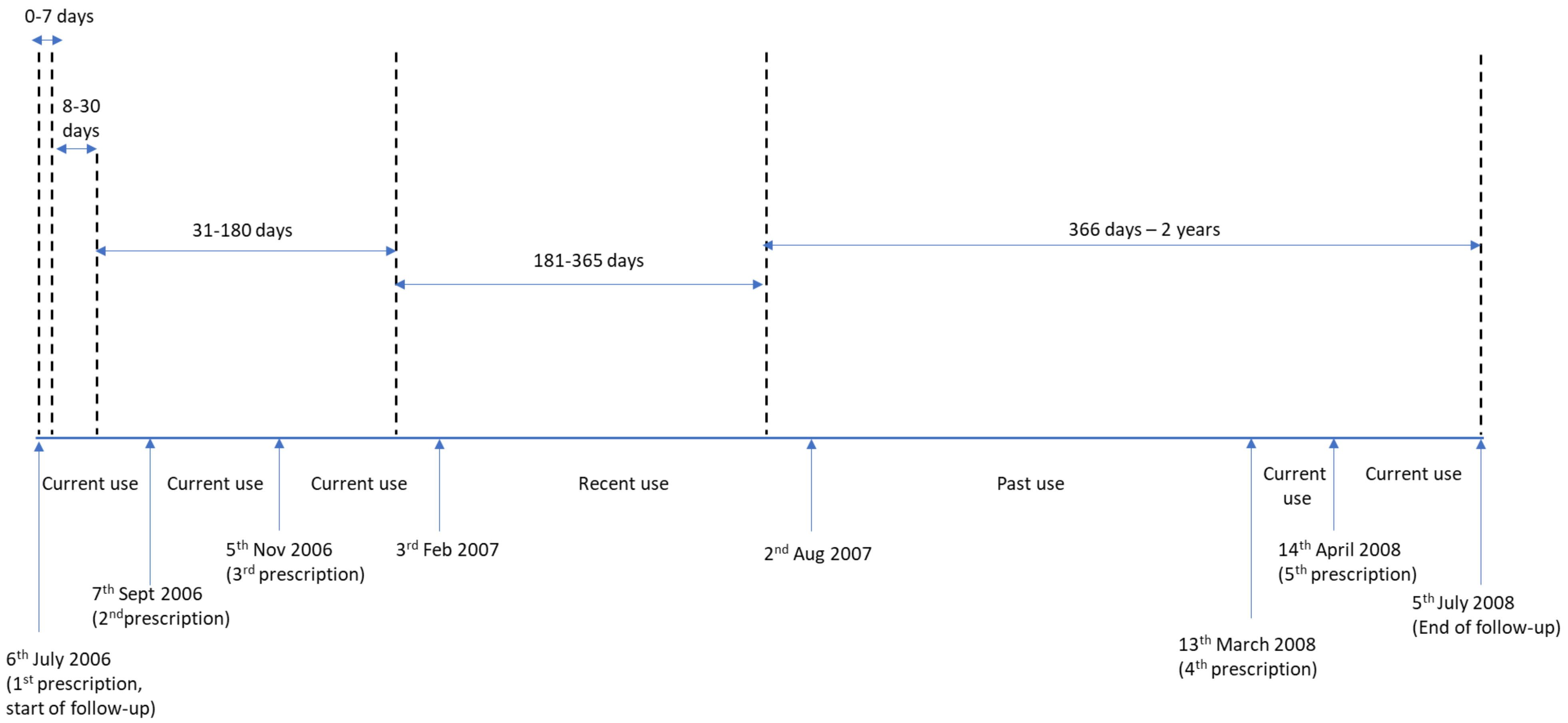

Illustration of the antipsychotic use status of a fictitious patient during follow-up, with the total time of follow-up stratified according to: the first 7 days, 8 to 30 days, 31 to 180 days, 181 to 365 days, and 366 days to 2 years.

Propensity score methods to control for potential confounding

We used propensity score methods to control for imbalances in measurable patient characteristics between exposed and their matched unexposed patients. Propensity scores is the probability of receiving a treatment based on observed characteristics, and the method is commonly used to address confounding bias in medical research (Austin 2011). Propensity scores are generated using statistical models with treatment assignment as the outcome and the observed characteristics as predictors. The scores derived from the model are then used to balance the distribution of the observed characteristics between the treated and untreated subjects.

For each patient included in this study, we derived a propensity score representing their probability of receiving antipsychotic treatment. Propensity scores models were run for each outcome, and for patients in the Aurum and GOLD cohorts separately. The procedures for the propensity analysis used was based on Lunt (2014), using the Stata programs therein. The propensity scores were calculated using multivariable logistic regression with exposure to antipsychotic treatment as the dependent variable (1=treated; 0=not treated) and a number of observable patient characteristics as predictors. These covariates included patient demographics, lifestyle, comorbidities, and prescribed medications - see the 'Covariates' subsection of Methods in the main manuscript for the full list.

To begin with, the standardised difference between the antipsychotic users (i.e. the treated) and their matched comparators (non-treated) for each of the covariates were checked. Multivariable logistic regression models were then used to generate a propensity score for each patient. We used the Hosmer-Lemeshow test and likelihood ratio test to assess the model fits, and additionally included interaction terms to improve the fit where necessary. To reduce potential bias associated with very large or small propensity scores, we excluded patients with scores that were outside the 'common support range', i.e. the range of scores observed for both treated and untreated subjects. In other words, we restricted our analysis to individuals whose propensity scores were within the common support range observed for both antipsychotic users and their matched comparators.

From the propensity scores, an inverse probability treatment weight (IPTW) was then generated for each patient. We derived standardised weights (also referred to as stabilized weights), which have a mean of 1 for both the treated and untreated groups (Lunt, 2014; Xu et al, 2010), as shown in the table below:

Outcomes	Groups	Mean weight (SD)	min	max	median
Stroke	Treated	1 (0.3978001)	0.14273	2.889276	0.9830475
	Non-treated	1 (0.0358866)	0.9551931	1.744002	0.9909414
VTE	Treated	1 (0.3743354)	0.11977	2.836945	0.9950411
	Non-treated	1 (0.0331883)	0.9565744	1.617068	0.9913114
MI	Treated	1 (0.3801787)	0.1588146	2.816721	0.985475
	Non-treated	1 (0.0331967)	0.9565395	1.653198	0.9916268
Heart failure	Treated	1 (0.3856317)	0.1542769	3.002008	0.9846388
	Non-treated	1 (0.0339143)	0.9550505	1.592077	0.9913915
Ventricular arrhythmia	Treated	1 (0.3772776)	0.1458979	2.891147	0.9858463
	Non-treated	1 (0.0329168)	0.9562746	1.689095	0.9917167
Fracture	Treated	1 (0.4112177)	0.1217533	5.393023	0.97565
	Non-treated	1 (0.0373307)	0.9440135	1.804627	0.990537
Pneumonia	Treated	1 (0.377883)	0.147601	3.00175	0.9875194
	Non-treated	1 (0.0330308)	0.9552449	1.656494	0.9917017
AKI	Treated	1 (0.3738156)	0.1485216	2.751141	0.9880923
	Non-treated	1 (0.0327157)	0.9572864	1.661161	0.9917589
Unrelated outcome	Treated	1 (0.3830144)	0.1483015	2.824021	0.9836412
	Non-treated	1 (0.0334483)	0.9565837	1.61189	0.9915277

We then used these weights to reweigh the data and rechecked the standardised differences between the antipsychotic users and their matched comparators for all the covariates. Table 1 and Supplementary Tables S2-S9 show that after IPT weighting, the standardised differences were <0.1 for all covariates, indicating there were good balance between the treated and non-treated groups in these covariates (Austin 2008). The IPTW were then used in the Cox regression models to estimate the risks of each outcome associated with antipsychotic use relative to the comparator cohort, with the hazard ratios calculated with IPTW adjustments.

References

Austin PC. Goodness-of-fit diagnostics for the propensity score model when estimating treatment effects using covariate adjustment with the propensity score. *Pharmacoepidem Drug Safe* 2008;17: 1202-17.

Austin PC. An introduction to propensity score methods for reducing the effects of confounding in observational studies. *Multivariate Behav Res* 2011;46:399-424.

Lunt M. Propensity analysis in Stata, revision: 1.1. 2014. University of Manchester. http://personalpages.manchester.ac.uk/staff/mark.lunt/propensity_guide.pdf [Assessed 2nd Sept 24th Oct 2023]

Xu S, Ross C, Raebel MA, Shetterly S, Blanchette C, Smith D. Use of stabilized inverse propensity scores as weights to directly estimate relative risk and its confidence intervals. *Value Health* 2010;13:273-7.

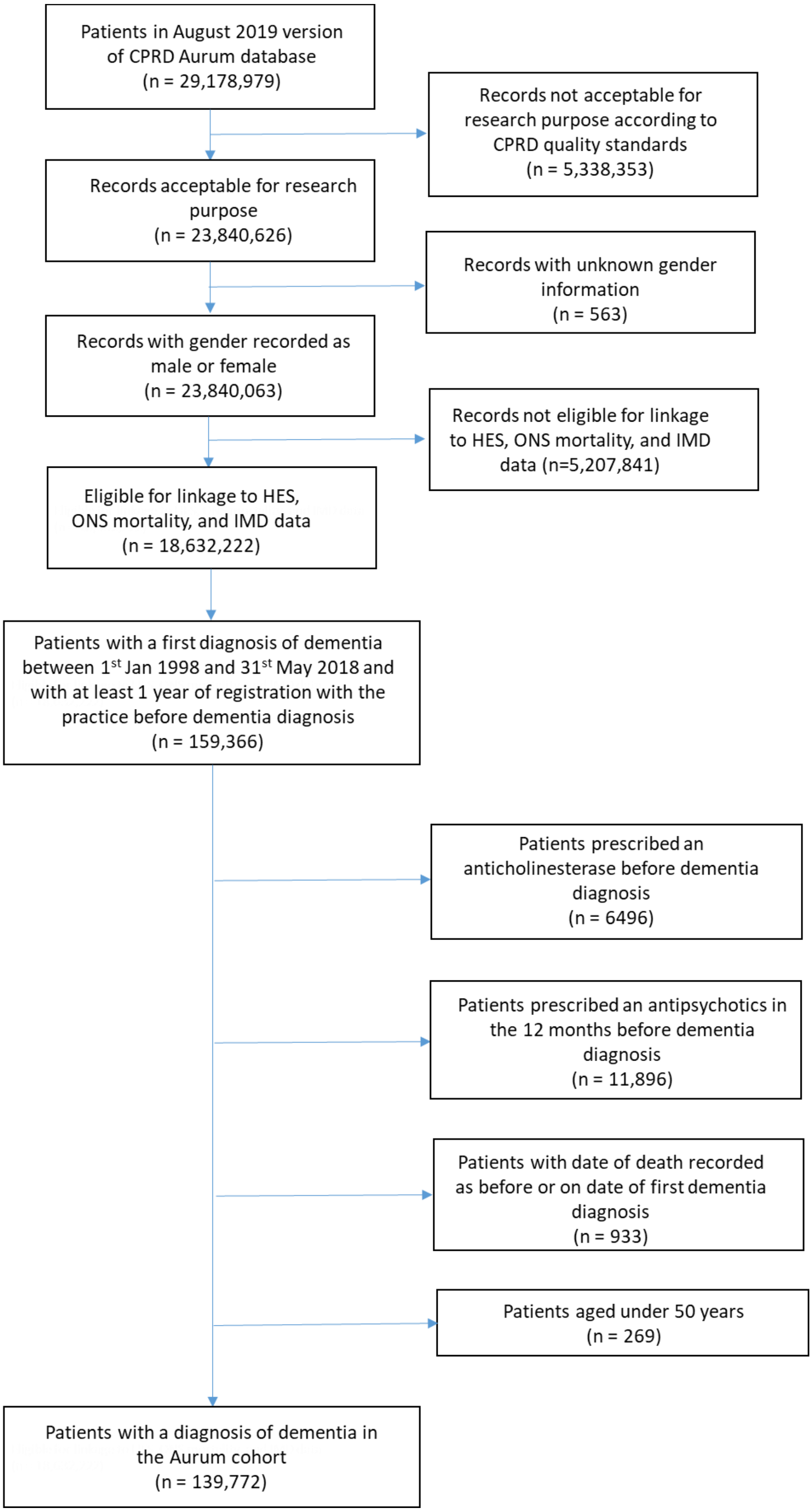

Supplementary Figure S1. Delineation of CPRD Aurum cohort of patients with a diagnosis of dementia

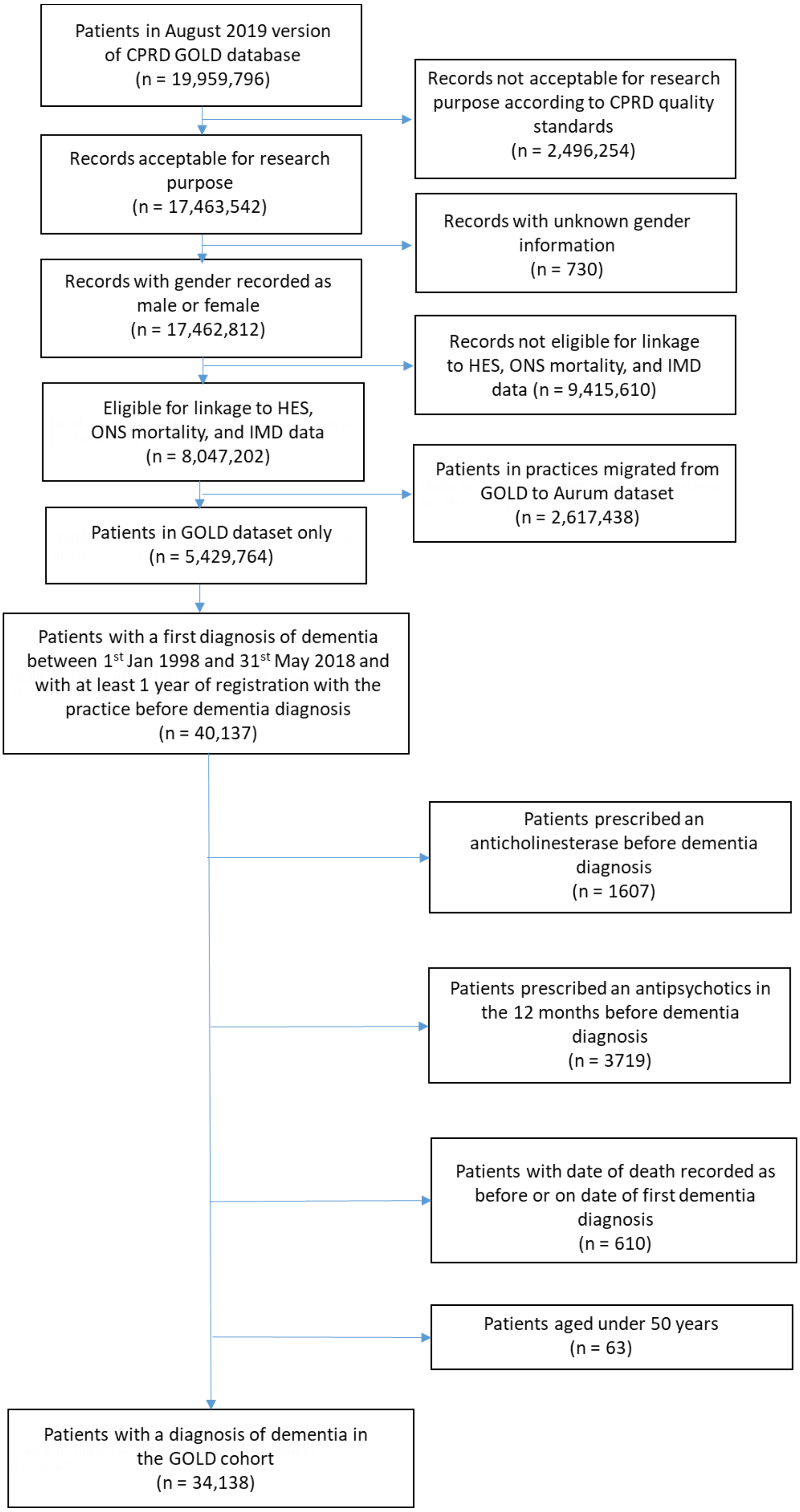

Supplementary Figure S2. Delineation of CPRD GOLD cohort of patients with a diagnosis of dementia

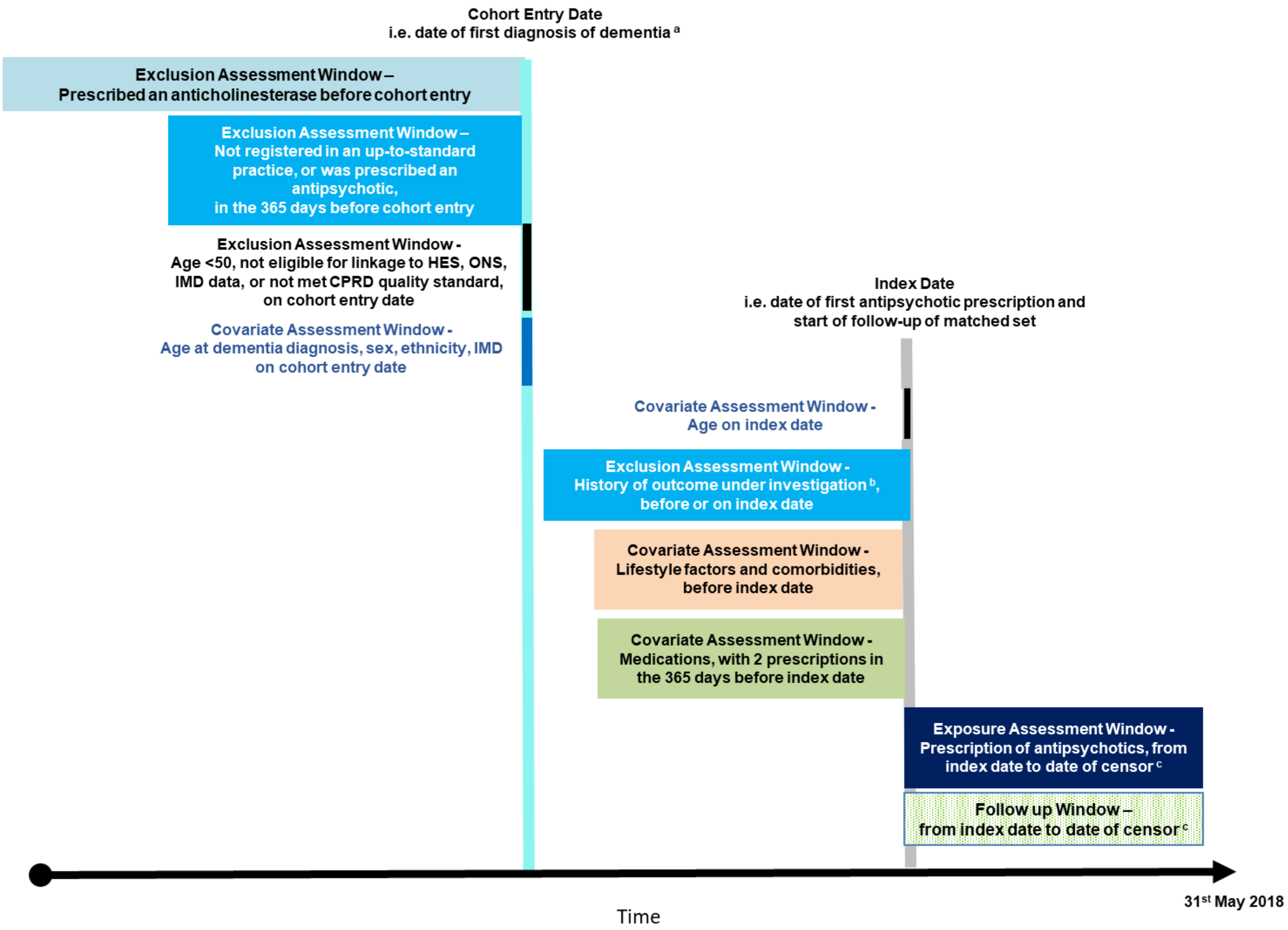

Supplementary Figure S3. Graphical depiction of study design

[a] Each antipsychotic user was matched with up to 15 non-users on date of dementia diagnosis using incidence sampling.
[b] For acute kidney injury (AKI), diagnosis of end stage kidney disease before or on index date was an additional criteria for exclusion.
[c] Earliest of: outcome of interest, last practice collection date, transfer out of practice, death, two years from the date of antipsychotics initiation, study end (31st May 2018), diagnosis of end stage kidney disease (AKI only). For matched comparators, date of first antipsychotic prescription was an additional criteria for censoring

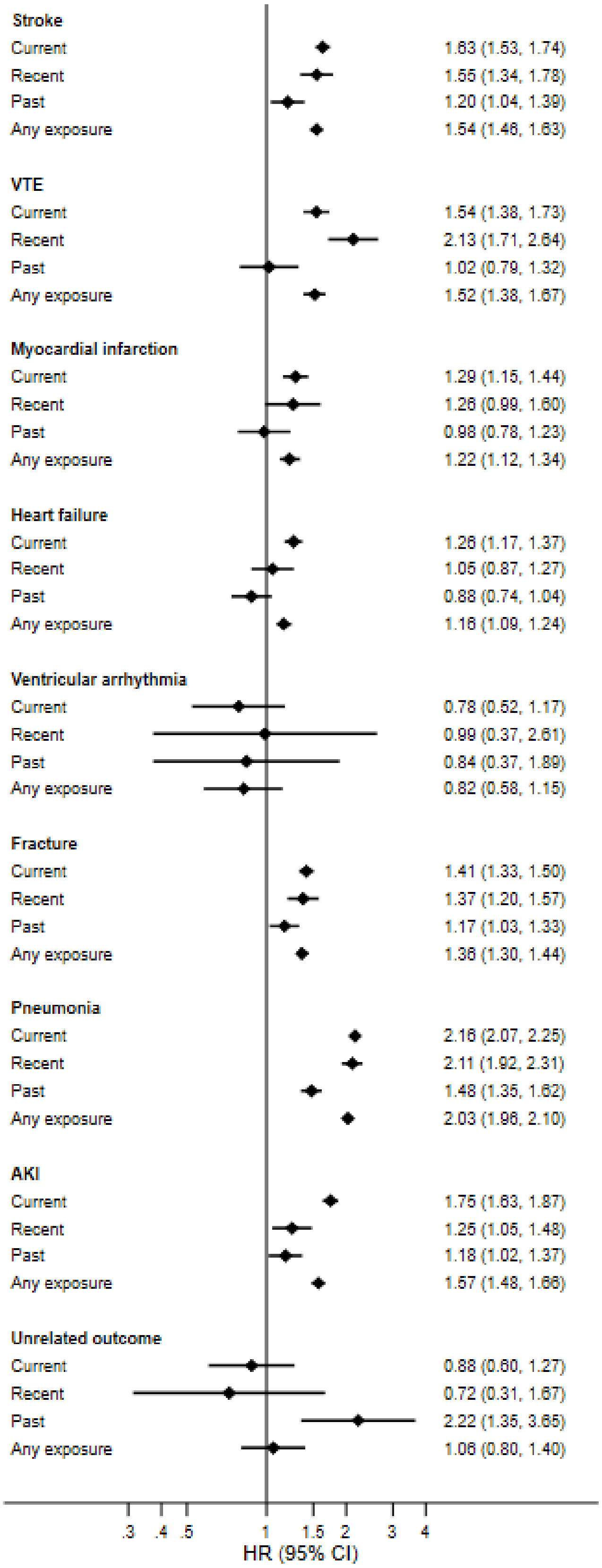

Supplementary Figure S4. Hazard ratios (adjusted for IPT weights) of adverse outcomes associated with current, recent, and past antipsychotic use; with current use being defined as the first 60 days from the date of an antipsychotic prescription, recent use as up to 120 days after current use ended, and past use as after recent use.

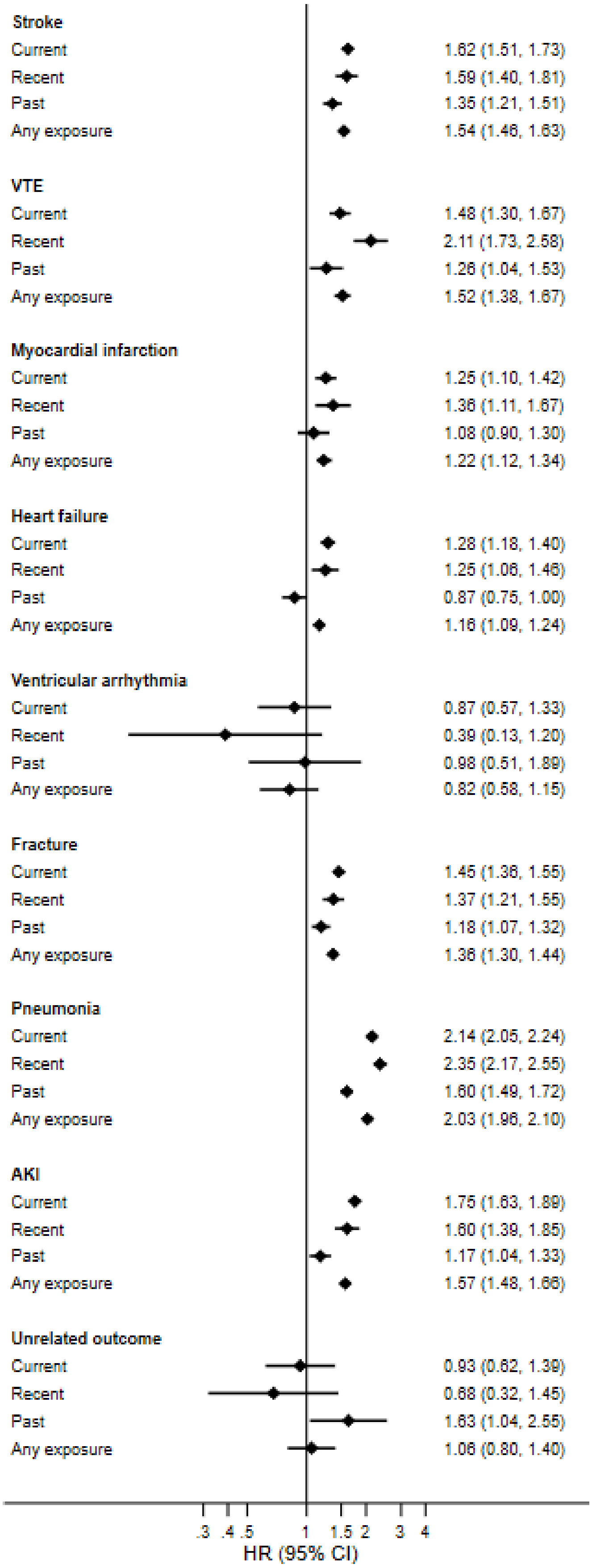

Supplementary Figure S5. Hazard ratios (adjusted for IPT weights) of adverse outcomes associated with current, recent, and past antipsychotic use; with current use being defined as the first 30 days from the date of an antipsychotic prescription, recent use as up to 60 days after current use ended, and past use as after recent use.

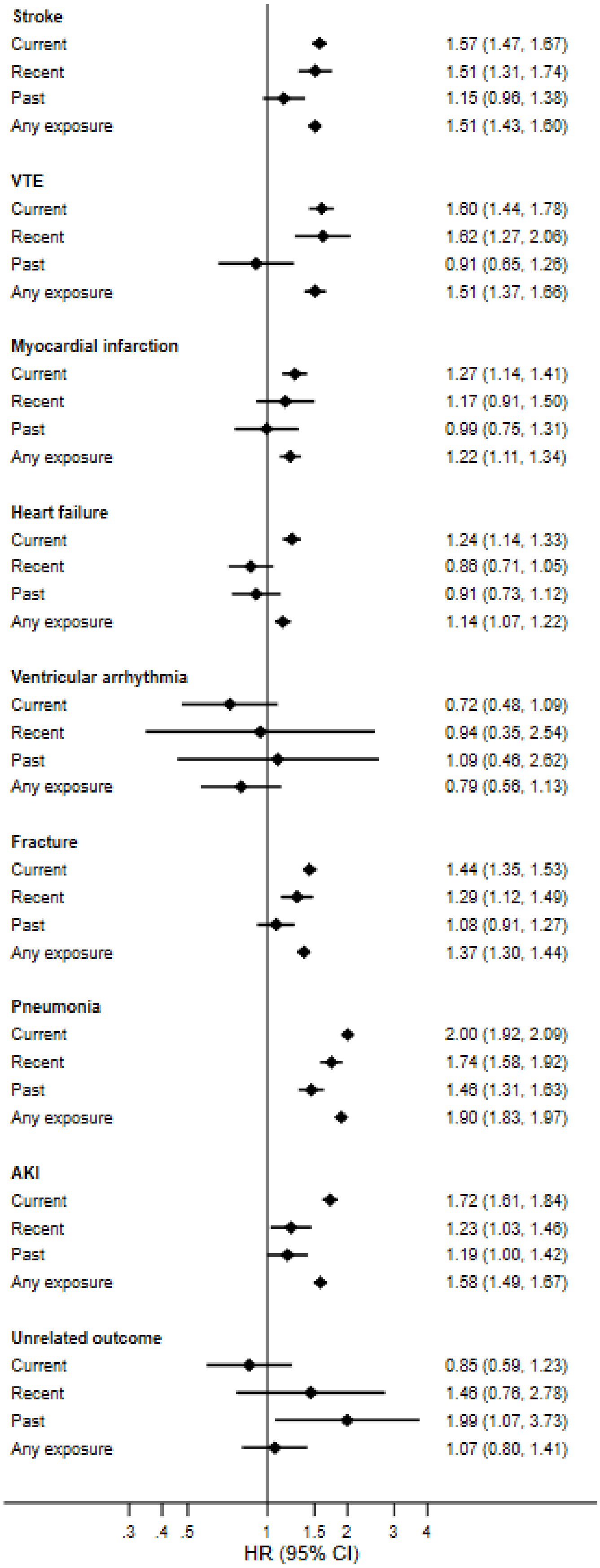

Supplementary Figure S6. Hazard ratios (adjusted for IPT weights) of adverse outcomes associated with current, recent, and past antipsychotic use excluding levomepromazine, with current use being defined as the first 90 days from the date of an antipsychotic prescription, recent use as up to 180 days after current use ended, and past use as after recent use.

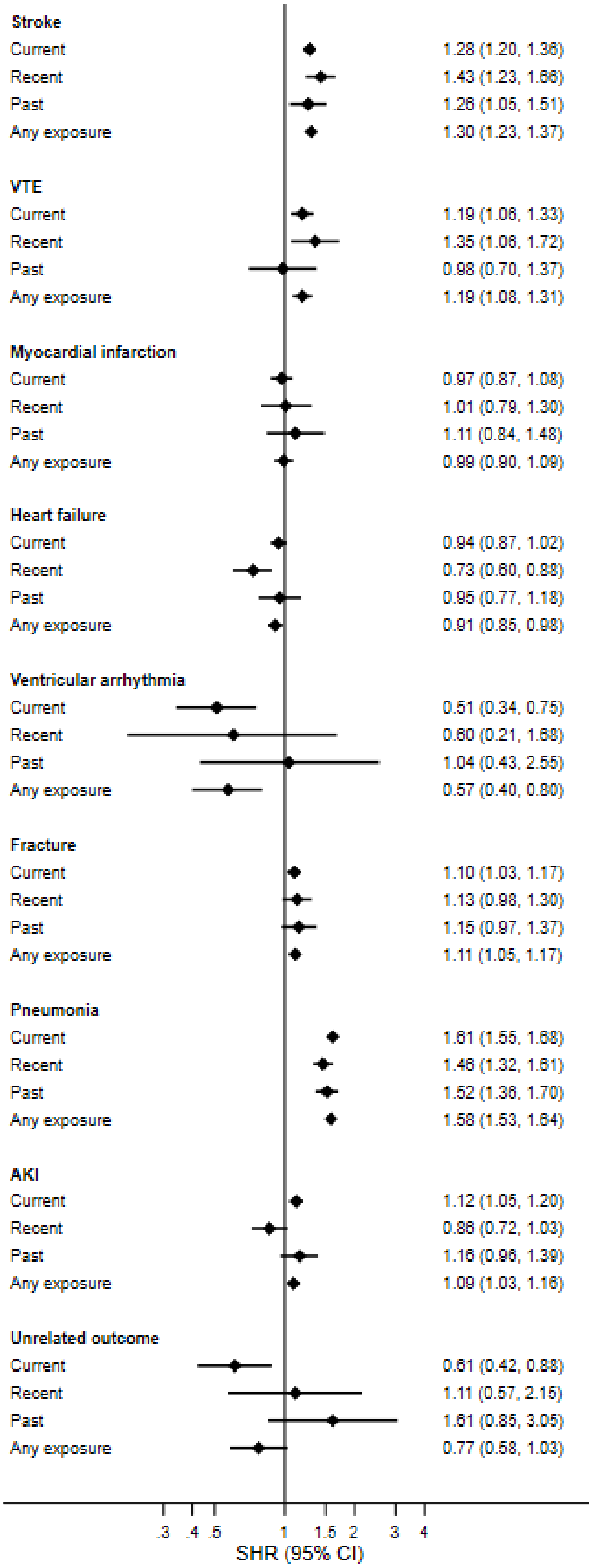

Supplementary Figure S7. Subhazard ratios (adjusted for IPT weights) of adverse outcomes associated with current, recent, and past antipsychotic use; with current use being defined as the first 90 days from the date of an antipsychotic prescription, recent use as up to 180 days after current use ended, and past use as after recent use.

Supplementary Table S1. Number of prescriptions by antipsychotic drug substance

Drug substance	Typical or atypical antipsychotic	Number of prescriptions issued [a]	Percentage of total prescriptions (%)
Amisulpride	Atypical	30,960	5.7
Aripiprazole	Atypical	8981	1.7
Benperidol	Typical	597	0.1
Chlorpromazine	Typical	7934	1.5
Clozapine	Atypical	28	0.0
Droperidol	Typical	66	0.0
Flupentixol	Typical	2446	0.4
Fluphenazine	Typical	61	0.0
Haloperidol	Typical	57,095	10.5
Levomepromazine	Typical	7031	1.3
Olanzapine	Atypical	48,109	8.8
Paliperidone	Atypical	14	0.0
Pericyazine	Typical	2346	0.4
Perphenazine	Typical	672	0.1
Pimozide	Typical	<10	0.0
Pipotiazine	Typical	13	0.0
Promazine	Typical	33,866	6.2
Quetiapine	Atypical	156,248	28.7
Risperidone	Atypical	162,174	29.8
Sulpiride	Typical	6600	1.2
Thioridazine	Typical	9279	1.7
Trifluoperazine	Typical	8097	1.5
Zotepine	Atypical	<10	0.0
Zuclopenthixol	Typical	1578	0.3
Total		544,203	100.0

[a] Total for Aurum and GOLD

Supplementary Table S2. Baseline characteristics of antipsychotic users and matched comparators included in the analysis of venous thromboembolism (CPRD Aurum and GOLD combined data)

	Before IPT weighting			After IPT weighting		
	Antipsychotics users (%) (n=30,565)	Matched comparators (%) (n=435,258)	Standardised difference	Antipsychotics users (%) (n=30,565)	Matched comparators (%) (n=435,258)	Standardised difference
Demographics						
Sex						
Male	11,464 (37.5)	156,832 (36.0)	0.031	10,991 (36.0)	157,249 (36.1)	-0.003
Female	19,101 (62.5)	278,426 (64.0)	-0.031	19,574 (64.0)	278,009 (63.9)	0.003
Age						
Mean (SD) age at dementia diagnosis	81.3 (8.1)	80.5 (8.0)	0.096	80.7 (8.0)	80.6 (8.0)	0.007
Mean (SD) age at start of follow up	83.0 (8.0)	82.0 (7.9)	0.128	82.0 (7.9)	82.0 (7.9)	0.002
Ethnicity						
White	22,384 (73.2)	329,936 (75.8)	-0.059	22,839 (74.7)	329,162 (75.6)	-0.021
Non-White	633 (2.1)	11,714 (2.7)	-0.041	816 (2.7)	11,536 (2.7)	0.001
Unknown	7548 (24.7)	93,608 (21.5)	0.076	6910 (22.6)	94,560 (21.7)	0.021
IMD quintile						
1 (least deprived)	6589 (21.6)	98,573 (22.6)	-0.026	6844 (22.4)	98,258 (22.6)	-0.004
2	6816 (22.3)	99,450 (22.8)	-0.013	6940 (22.7)	99,288 (22.8)	-0.002
3	6493 (21.2)	88,698 (20.4)	0.021	6178 (20.2)	88,936 (20.4)	-0.005
4	5667 (18.5)	79,973 (18.4)	0.004	5671 (18.6)	80,027 (18.4)	0.004
5 (most deprived)	4975 (16.3)	68,200 (15.7)	0.017	4906 (16.1)	68,386 (15.7)	0.009
Unknown	25 (0.1)	364 (0.1)	-0.001	25 (0.1)	364 (0.1)	0.000
Lifestyle						
Smoking						
Current smoker	5243 (17.2)	75,969 (17.5)	-0.008	5378 (17.6)	75,894 (17.4)	0.004
Ex-smoker	13,406 (43.9)	191,830 (44.1)	-0.004	13,297 (43.5)	191,745 (44.1)	-0.011
Never smoker	10,078 (33.0)	144,561 (33.2)	-0.005	10,204 (33.4)	144,496 (33.2)	0.004
Unknown	1838 (6.0)	22,898 (5.3)	0.033	1685 (5.5)	23,123 (5.3)	0.009
Alcohol use						
Non-drinker	4880 (16.0)	66,342 (15.2)	0.020	4763 (15.6)	66,561 (15.3)	0.008
Light drinker	3428 (11.2)	50,766 (11.7)	-0.014	3557 (11.6)	50,636 (11.6)	0.000

	Before IPT weighting			After IPT weighting		
	Antipsychotics users (%) (n=30,565)	Matched comparators (%) (n=435,258)	Standardised difference	Antipsychotics users (%) (n=30,565)	Matched comparators (%) (n=435,258)	Standardised difference
Former drinker	1326 (4.3)	16,615 (3.8)	0.026	1189 (3.9)	16,766 (3.9)	0.002
Moderate drinker	11,419 (37.4)	170,370 (39.1)	-0.037	11,693 (38.3)	169,828 (39.0)	-0.016
Heavy drinker	1300 (4.3)	19,808 (4.6)	-0.015	1390 (4.5)	19,724 (4.5)	0.001
Unknown	8212 (26.9)	111,357 (25.6)	0.029	7972 (26.1)	111,742 (25.7)	0.009
Comorbidities [a]						
Hypertension	12,297 (40.2)	180,866 (41.6)	-0.027	12,549 (41.1)	180,471 (41.5)	-0.008
Diabetes	4434 (14.5)	65,575 (15.1)	-0.016	4571 (15.0)	65,413 (15.0)	-0.002
COPD	5673 (18.6)	75,437 (17.3)	0.032	5370 (17.6)	75,790 (17.4)	0.004
Rheumatoid arthritis	609 (2.0)	8332 (1.9)	0.006	599 (2.0)	8354 (1.9)	0.003
Moderate/severe renal disease	6935 (22.7)	91,329 (21.0)	0.041	6343 (20.8)	91,804 (21.1)	-0.008
Moderate/severe liver disease	237 (0.8)	3314 (0.8)	0.002	235 (0.8)	3319 (0.8)	0.001
Atrial fibrillation	4438 (14.5)	57,419 (13.2)	0.038	4071 (13.3)	57,802 (13.3)	0.001
Cancer	4885 (16.0)	58,020 (13.3)	0.075	4128 (13.5)	58,776 (13.5)	0.000
Serious mental illness	677 (2.2)	4224 (1.0)	0.100	360 (1.2)	4596 (1.1)	0.010
Prescribed medications						
Antiplatelets	12,821 (41.9)	179,906 (41.3)	0.012	12,707 (41.6)	180,090 (41.4)	0.004
Oral anticoagulants	1888 (6.2)	26,195 (6.0)	0.007	1825 (6.0)	26,239 (6.0)	-0.002
ACE inhibitors or ARB	7790 (25.5)	124,814 (28.7)	-0.072	8620 (28.2)	123,891 (28.5)	-0.006
Alpha blockers	2076 (6.8)	31,050 (7.1)	-0.013	2132 (7.0)	30,947 (7.1)	-0.005
Beta blockers	5880 (19.2)	83,481 (19.2)	0.001	5833 (19.1)	83,496 (19.2)	-0.002
Calcium channel blockers	5825 (19.1)	91,758 (21.1)	-0.051	6379 (20.9)	91,170 (20.9)	-0.002
Diuretics	8886 (29.1)	125,438 (28.8)	0.006	8820 (28.9)	125,509 (28.8)	0.000
Lipid lowering drugs	9922 (32.5)	151,865 (34.9)	-0.051	10,452 (34.2)	151,146 (34.7)	-0.011
Insulin and antidiabetic drugs	2994 (9.8)	45,236 (10.4)	-0.020	3158 (10.3)	45,063 (10.4)	-0.001
NSAID	3743 (12.2)	51,218 (11.8)	0.015	3644 (11.9)	51,361 (11.8)	0.004
Antidepressants	10,918 (35.7)	117,722 (27.0)	0.188	8526 (27.9)	120,217 (27.6)	0.006
Benzodiazepines	4636 (15.2)	29,219 (6.7)	0.273	2293 (7.5)	31,658 (7.3)	0.007
Lithium	83 (0.3)	973 (0.2)	0.010	83 (0.3)	988 (0.2)	0.009
HRT	94 (0.3)	1962 (0.5)	-0.023	141 (0.5)	1918 (0.4)	0.003
Selective oestrogen receptor modulators	225 (0.7)	3209 (0.7)	0.000	227 (0.7)	3209 (0.7)	0.001

[a] History of the condition

Supplementary Table S3. Baseline characteristics of antipsychotic users and matched comparators included in the analysis of myocardial infarction (CPRD Aurum and GOLD combined data)

	Before IPT weighting			After IPT weighting		
	Antipsychotics users (%) (n=29,541)	Matched comparators (%) (n=419,300)	Standardised difference	Antipsychotics users (%) (n=29,541)	Matched comparators (%) (n=419,300)	Standardised difference
Demographics						
Sex						
Male	10,560 (35.7)	143,956 (34.3)	0.030	10,076 (34.1)	144,334 (34.4)	-0.007
Female	18,981 (64.3)	275,344 (65.7)	-0.030	19,465 (65.9)	274,966 (65.6)	0.007
Age						
Mean (SD) age at dementia diagnosis	81.3 (8.2)	80.5 (8.0)	0.095	80.6 (8.0)	80.6 (8.0)	0.004
Mean (SD) age at start of follow up	83.0 (8.0)	81.9 (8.0)	0.128	82.0 (7.9)	82.0 (8.0)	-0.002
Ethnicity						
White	21,661 (73.3)	317,872 (75.8)	-0.057	22,059 (74.7)	317,148 (75.6)	-0.022
Non-White	587 (2.0)	10,969 (2.6)	-0.042	776 (2.6)	10,794 (2.6)	0.003
Unknown	7293 (24.7)	90,459 (21.6)	0.074	6706 (22.7)	91,358 (21.8)	0.022
IMD quintile						
1 (least deprived)	6435 (21.8)	95,483 (22.8)	-0.024	6657 (22.5)	95,208 (22.7)	-0.004
2	6619 (22.4)	95,846 (22.9)	-0.011	6690 (22.6)	95,714 (22.8)	-0.004
3	6290 (21.3)	85,654 (20.4)	0.021	5998 (20.3)	85,885 (20.5)	-0.004
4	5435 (18.4)	76,826 (18.3)	0.002	5459 (18.5)	76,851 (18.3)	0.004
5 (most deprived)	4740 (16.0)	65,151 (15.5)	0.014	4713 (16.0)	65,304 (15.6)	0.010
Unknown	22 (0.1)	340 (0.1)	-0.002	25 (0.1)	338 (0.1)	0.001
Lifestyle						
Smoking						
Current smoker	4973 (16.8)	71,796 (17.1)	-0.008	5099 (17.3)	71,724 (17.1)	0.004
Ex-smoker	12,754 (43.2)	181,861 (43.4)	-0.004	12,635 (42.8)	181,783 (43.4)	-0.012
Never smoker	10,014 (33.9)	142,915 (34.1)	-0.004	10,122 (34.3)	142,870 (34.1)	0.004
Unknown	1800 (6.1)	22,728 (5.4)	0.029	1685 (5.7)	22,923 (5.5)	0.010
Alcohol use						
Non-drinker	4736 (16.0)	64,154 (15.3)	0.020	4632 (15.7)	64,367 (15.4)	0.009
Light drinker	3258 (11.0)	48,648 (11.6)	-0.018	3407 (11.5)	48,488 (11.6)	-0.001

	Before IPT weighting			After IPT weighting		
	Antipsychotics users (%) (n=29,541)	Matched comparators (%) (n=419,300)	Standardised difference	Antipsychotics users (%) (n=29,541)	Matched comparators (%) (n=419,300)	Standardised difference
Former drinker	1246 (4.2)	15,807 (3.8)	0.023	1131 (3.8)	15,932 (3.8)	0.001
Moderate drinker	10,971 (37.1)	161,960 (38.6)	-0.031	11,143 (37.7)	161,519 (38.5)	-0.017
Heavy drinker	1264 (4.3)	19,299 (4.6)	-0.016	1361 (4.6)	19,211 (4.6)	0.001
Unknown	8066 (27.3)	109,432 (26.1)	0.027	7867 (26.6)	109,782 (26.2)	0.010
Comorbidities [a]						
Hypertension	11,808 (40.0)	172,867 (41.2)	-0.026	12,037 (40.7)	172,504 (41.1)	-0.008
Diabetes	4046 (13.7)	60,563 (14.4)	-0.022	4264 (14.4)	60,355 (14.4)	0.001
COPD	5445 (18.4)	71,742 (17.1)	0.035	5120 (17.3)	72,111 (17.2)	0.003
Rheumatoid arthritis	605 (2.0)	8254 (2.0)	0.006	593 (2.0)	8276 (2.0)	0.002
Moderate/severe renal disease	6441 (21.8)	84,919 (20.3)	0.038	5907 (20.0)	85,335 (20.4)	-0.009
Moderate/severe liver disease	220 (0.7)	3236 (0.8)	-0.003	227 (0.8)	3229 (0.8)	0.000
Atrial fibrillation	4192 (14.2)	53,942 (12.9)	0.039	3824 (12.9)	54,308 (13.0)	0.000
Cancer	4797 (16.2)	56,589 (13.5)	0.077	4024 (13.6)	57,346 (13.7)	-0.002
Serious mental illness	672 (2.3)	4186 (1.0)	0.101	362 (1.2)	4558 (1.1)	0.011
Prescribed medications						
Antiplatelets	11,350 (38.4)	158,054 (37.7)	0.015	11,206 (37.9)	158,261 (37.7)	0.004
Oral anticoagulants	2077 (7.0)	28,500 (6.8)	0.009	1992 (6.7)	28,562 (6.8)	-0.003
ACE inhibitors or ARB	6925 (23.4)	111,154 (26.5)	-0.071	7706 (26.1)	110,297 (26.3)	-0.005
Alpha blockers	1951 (6.6)	29,099 (6.9)	-0.013	1999 (6.8)	28,999 (6.9)	-0.006
Beta blockers	4934 (16.7)	69,529 (16.6)	0.003	4887 (16.5)	69,563 (16.6)	-0.001
Calcium channel blockers	5524 (18.7)	86,617 (20.7)	-0.049	6050 (20.5)	86,069 (20.5)	-0.001
Diuretics	8434 (28.6)	118,388 (28.2)	0.007	8359 (28.3)	118,476 (28.3)	0.001
Lipid lowering drugs	8663 (29.3)	133,182 (31.8)	-0.053	9208 (31.2)	132,486 (31.6)	-0.009
Insulin and antidiabetic drugs	2702 (9.1)	41,604 (9.9)	-0.026	2933 (9.9)	41,389 (9.9)	0.002
NSAID	3645 (12.3)	49,480 (11.8)	0.017	3548 (12.0)	49,636 (11.8)	0.005
Antidepressants	10,569 (35.8)	113,998 (27.2)	0.186	8295 (28.1)	116,390 (27.8)	0.007
Benzodiazepines	4468 (15.1)	28,036 (6.7)	0.273	2201 (7.4)	30,389 (7.2)	0.007
Lithium	88 (0.3)	944 (0.2)	0.014	79 (0.3)	965 (0.2)	0.007

[a] History of the condition

Supplementary Table S4. Baseline characteristics of antipsychotic users and matched comparators included in the analysis of heart failure (CPRD Aurum and GOLD combined data)

	Before IPT weighting			After IPT weighting		
	Antipsychotics users (%) (n=28,348)	Matched comparators (%) (n=401,058)	Standardised difference	Antipsychotics users (%) (n=28,348)	Matched comparators (%) (n=401,058)	Standardised difference
Demographics						
Sex						
Male	10,483 (37.0)	142,816 (35.6)	0.028	10,019 (35.3)	143,164 (35.7)	-0.007
Female	17,865 (63.0)	258,242 (64.4)	-0.028	18,329 (64.7)	257,895 (64.3)	0.007
Age						
Mean (SD) age at dementia diagnosis	80.9 (8.2)	80.3 (8.0)	0.084	80.3 (8.0)	80.3 (8.0)	0.003
Mean (SD) age at start of follow up	82.6 (8.0)	81.7 (8.0)	0.119	81.7 (7.9)	81.7 (8.0)	-0.003
Ethnicity						
White	20,848 (73.5)	305,770 (76.2)	-0.062	21,308 (75.2)	305,021 (76.1)	-0.021
Non-White	555 (2.0)	10,482 (2.6)	-0.044	745 (2.6)	10,307 (2.6)	0.004
Unknown	6945 (24.5)	84,806 (21.1)	0.080	6296 (22.2)	85,729 (21.4)	0.020
IMD quintile						
1 (least deprived)	6196 (21.9)	92,113 (23.0)	-0.027	6433 (22.7)	91,817 (22.9)	-0.005
2	6385 (22.5)	91,559 (22.8)	-0.007	6430 (22.7)	91,472 (22.8)	-0.003
3	6007 (21.2)	82,015 (20.4)	0.018	5767 (20.3)	82,205 (20.5)	-0.004
4	5194 (18.3)	73,236 (18.3)	0.002	5220 (18.4)	73,256 (18.3)	0.004
5 (most deprived)	4543 (16.0)	61,817 (15.4)	0.017	4475 (15.8)	61,990 (15.5)	0.009
Unknown	23 (0.1)	318 (0.1)	0.001	23 (0.1)	319 (0.1)	0.001
Lifestyle						
Smoking						
Current smoker	4870 (17.2)	69,857 (17.4)	-0.006	4975 (17.5)	69,804 (17.4)	0.004
Ex-smoker	12,296 (43.4)	175,394 (43.7)	-0.007	12,230 (43.1)	175,274 (43.7)	-0.011
Never smoker	9489 (33.5)	134,786 (33.6)	-0.003	9574 (33.8)	134,756 (33.6)	0.004
Unknown	1693 (6.0)	21,021 (5.2)	0.032	1569 (5.5)	21,224 (5.3)	0.011
Alcohol use						
Non-drinker	4508 (15.9)	60,468 (15.1)	0.023	4379 (15.4)	60,696 (15.1)	0.009
Light drinker	3200 (11.3)	46,988 (11.7)	-0.013	3303 (11.7)	46,873 (11.7)	-0.001

	Before IPT weighting			After IPT weighting		
	Antipsychotics users (%) (n=28,348)	Matched comparators (%) (n=401,058)	Standardised difference	Antipsychotics users (%) (n=28,348)	Matched comparators (%) (n=401,058)	Standardised difference
Former drinker	1224 (4.3)	15,268 (3.8)	0.026	1096 (3.9)	15,405 (3.8)	0.001
Moderate drinker	10,599 (37.4)	156,955 (39.1)	-0.036	10,830 (38.2)	156,463 (39.0)	-0.017
Heavy drinker	1242 (4.4)	18,746 (4.7)	-0.014	1330 (4.7)	18,670 (4.7)	0.002
Unknown	7575 (26.7)	102,633 (25.6)	0.026	7410 (26.1)	102,951 (25.7)	0.011
Comorbidities [a]						
Hypertension	11,136 (39.3)	163,220 (40.7)	-0.029	11,403 (40.2)	162,833 (40.6)	-0.008
Diabetes	3775 (13.3)	56,878 (14.2)	-0.025	4018 (14.2)	56,650 (14.1)	0.001
COPD	4789 (16.9)	64,509 (16.1)	0.022	4604 (16.2)	64,726 (16.1)	0.003
Rheumatoid arthritis	562 (2.0)	7678 (1.9)	0.005	562 (2.0)	7697 (1.9)	0.005
Moderate/severe renal disease	5834 (20.6)	77,207 (19.3)	0.033	5382 (19.0)	77,549 (19.3)	-0.009
Moderate/severe liver disease	195 (0.7)	2905 (0.7)	-0.004	201 (0.7)	2895 (0.7)	-0.002
Atrial fibrillation	3173 (11.2)	40,810 (10.2)	0.033	2882 (10.2)	41,075 (10.2)	-0.002
Cancer	4589 (16.2)	53,683 (13.4)	0.079	3830 (13.5)	54,422 (13.6)	-0.002
Serious mental illness	661 (2.3)	3920 (1.0)	0.106	342 (1.2)	4295 (1.1)	0.011
Prescribed medications						
Antiplatelets	11,244 (39.7)	157,985 (39.4)	0.006	11,188 (39.5)	158,062 (39.4)	0.001
Oral anticoagulants	1569 (5.5)	21,671 (5.4)	0.006	1509 (5.3)	21,701 (5.4)	-0.004
ACE inhibitors or ARB	6326 (22.3)	102,764 (25.6)	-0.078	7123 (25.1)	101,876 (25.4)	-0.006
Alpha blockers	1851 (6.5)	27,758 (6.9)	-0.016	1914 (6.8)	27,648 (6.9)	-0.006
Beta blockers	4975 (17.5)	69,850 (17.4)	0.004	4927 (17.4)	69,885 (17.4)	-0.001
Calcium channel blockers	5444 (19.2)	85,406 (21.3)	-0.052	5983 (21.1)	84,847 (21.2)	-0.001
Diuretics	6842 (24.1)	99,203 (24.7)	-0.014	6992 (24.7)	99,042 (24.7)	-0.001
Lipid lowering drugs	8804 (31.1)	135,151 (33.7)	-0.056	9363 (33.0)	134,429 (33.5)	-0.011
Insulin and antidiabetic drugs	2505 (8.8)	38,931 (9.7)	-0.030	2754 (9.7)	38,701 (9.6)	0.002
NSAID	3494 (12.3)	47,469 (11.8)	0.015	3413 (12.0)	47,605 (11.9)	0.005
Antidepressants	10,113 (35.7)	107,907 (26.9)	0.190	7881 (27.8)	110,249 (27.5)	0.007
Benzodiazepines	4272 (15.1)	26,292 (6.6)	0.277	2074 (7.3)	28,567 (7.1)	0.006
Lithium	85 (0.3)	906 (0.2)	0.014	77 (0.3)	927 (0.2)	0.008

[a] History of the condition

Supplementary Table S5. Baseline characteristics of antipsychotic users and matched comparators included in the analysis of ventricular arrhythmia (CPRD Aurum and GOLD combined data)

	Before IPT weighting			After IPT weighting		
	Antipsychotics users (%) (n=32,414)	Matched comparators (%) (n=463,525)	Standardised difference	Antipsychotics users (%) (n=32,414)	Matched comparators (%) (n=463,525)	Standardised difference
Demographics						
Sex						
Male	12,047 (37.2)	165,688 (35.7)	0.030	11,593 (35.8)	166,117 (35.8)	-0.002
Female	20,367 (62.8)	297,837 (64.3)	-0.030	20,821 (64.2)	297,408 (64.2)	0.002
Age						
Mean (SD) age at dementia diagnosis	81.4 (8.1)	80.6 (7.9)	0.100	80.7 (7.9)	80.7 (8.0)	0.001
Mean (SD) age at start of follow up	83.1 (8.0)	82.0 (7.9)	0.131	82.1 (7.9)	82.1 (7.9)	-0.004
Ethnicity						
White	23,884 (73.7)	353,472 (76.3)	-0.059	24,366 (75.2)	352,653 (76.1)	-0.021
Non-White	667 (2.1)	12,334 (2.7)	-0.040	857 (2.6)	12,150 (2.6)	0.001
Unknown	7863 (24.3)	97,719 (21.1)	0.076	7192 (22.2)	98,722 (21.3)	0.022
IMD quintile						
1 (least deprived)	6987 (21.6)	104,758 (22.6)	-0.025	7238 (22.3)	104,438 (22.5)	-0.005
2	7256 (22.4)	105,607 (22.8)	-0.010	7326 (22.6)	105,479 (22.8)	-0.004
3	6901 (21.3)	94,892 (20.5)	0.020	6593 (20.3)	95,132 (20.5)	-0.005
4	5995 (18.5)	85,145 (18.4)	0.003	6013 (18.6)	85,190 (18.4)	0.004
5 (most deprived)	5250 (16.2)	72,755 (15.7)	0.014	5217 (16.1)	72,919 (15.7)	0.010
Unknown	25 (0.1)	368 (0.1)	-0.001	25 (0.1)	367 (0.1)	0.000
Lifestyle						
Smoking						
Current smoker	5532 (17.1)	80,679 (17.4)	-0.009	5695 (17.6)	80,587 (17.4)	0.005
Ex-smoker	14,283 (44.1)	205,370 (44.3)	-0.005	14,188 (43.8)	205,275 (44.3)	-0.010
Never smoker	10,701 (33.0)	153,652 (33.1)	-0.003	10,782 (33.3)	153,613 (33.1)	0.003
Unknown	1898 (5.9)	23,824 (5.1)	0.031	1749 (5.4)	24,050 (5.2)	0.009
Alcohol use						
Non-drinker	5217 (16.1)	71,202 (15.4)	0.020	5090 (15.7)	71,436 (15.4)	0.008
Light drinker	3626 (11.2)	54,137 (11.7)	-0.015	3770 (11.6)	53,986 (11.6)	-0.001

	Before IPT weighting			After IPT weighting		
	Antipsychotics users (%) (n=32,414)	Matched comparators (%) (n=463,525)	Standardised difference	Antipsychotics users (%) (n=32,414)	Matched comparators (%) (n=463,525)	Standardised difference
Former drinker	1419 (4.4)	17,906 (3.9)	0.026	1283 (4.0)	18,065 (3.9)	0.003
Moderate drinker	12,086 (37.3)	180,959 (39.0)	-0.036	12,388 (38.2)	180,397 (38.9)	-0.014
Heavy drinker	1382 (4.3)	21,090 (4.5)	-0.014	1481 (4.6)	21,005 (4.5)	0.002
Unknown	8684 (26.8)	118,231 (25.5)	0.029	8402 (25.9)	118,636 (25.6)	0.007
Comorbidities [a]						
Hypertension	13,101 (40.4)	193,479 (41.7)	-0.027	13,364 (41.2)	193,061 (41.7)	-0.009
Diabetes	4746 (14.6)	70,722 (15.3)	-0.017	4922 (15.2)	70,533 (15.2)	-0.001
COPD	6180 (19.1)	82,535 (17.8)	0.032	5859 (18.1)	82,921 (17.9)	0.005
Rheumatoid arthritis	664 (2.0)	9116 (2.0)	0.006	652 (2.0)	9141 (2.0)	0.003
Moderate/severe renal disease	7518 (23.2)	99,662 (21.5)	0.041	6880 (21.2)	100,162 (21.6)	-0.009
Moderate/severe liver disease	263 (0.8)	3661 (0.8)	0.002	256 (0.8)	3668 (0.8)	0.000
Atrial fibrillation	4774 (14.7)	62,292 (13.4)	0.037	4375 (13.5)	62,683 (13.5)	-0.001
Cancer	5324 (16.4)	63,253 (13.6)	0.078	4478 (13.8)	64,094 (13.8)	0.000
Serious mental illness	729 (2.2)	4524 (1.0)	0.101	385 (1.2)	4927 (1.1)	0.010
Prescribed medications						
Antiplatelets	13,517 (41.7)	190,455 (41.1)	0.012	13,394 (41.3)	190,648 (41.1)	0.004
Oral anticoagulants	2381 (7.3)	33,226 (7.2)	0.007	2291 (7.1)	33,275 (7.2)	-0.004
ACE inhibitors or ARB	8282 (25.6)	133,392 (28.8)	-0.073	9177 (28.3)	132,403 (28.6)	-0.006
Alpha blockers	2193 (6.8)	33,147 (7.2)	-0.015	2271 (7.0)	33,024 (7.1)	-0.005
Beta blockers	6204 (19.1)	88,383 (19.1)	0.002	6147 (19.0)	88,404 (19.1)	-0.003
Calcium channel blockers	6190 (19.1)	97,742 (21.1)	-0.050	6771 (20.9)	97,131 (21.0)	-0.002
Diuretics	9594 (29.6)	135,556 (29.2)	0.008	9484 (29.3)	135,662 (29.3)	0.000
Lipid lowering drugs	10,562 (32.6)	162,892 (35.1)	-0.054	11,178 (34.5)	162,091 (35.0)	-0.010
Insulin and antidiabetic drugs	3211 (9.9)	48,814 (10.5)	-0.021	3401 (10.5)	48,623 (10.5)	0.000
NSAID	4002 (12.3)	54,610 (11.8)	0.017	3880 (12.0)	54,789 (11.8)	0.005
Antidepressants	11,686 (36.1)	126,887 (27.4)	0.187	9170 (28.3)	129,538 (27.9)	0.007
Benzodiazepines	4907 (15.1)	31,203 (6.7)	0.272	2434 (7.5)	33,776 (7.3)	0.007
Lithium	91 (0.3)	1038 (0.2)	0.011	88 (0.3)	1057 (0.2)	0.009

[a] History of the condition

Supplementary Table S6. Baseline characteristics of antipsychotic users and matched comparators included in the analysis of fracture (CPRD Aurum and GOLD combined data)

	Before IPT weighting			After IPT weighting		
	Antipsychotics users (%) (n=20,508)	Matched comparators (%) (n=279,601)	Standardised difference	Antipsychotics users (%) (n=20,508)	Matched comparators (%) (n=279,601)	Standardised difference
Demographics						
Sex						
Male	9027 (44.0)	115,490 (41.3)	0.055	8485 (41.4)	116,008 (41.5)	-0.002
Female	11,481 (56.0)	164,111 (58.7)	-0.055	12,023 (58.6)	163,593 (58.5)	0.002
Age						
Mean (SD) age at dementia diagnosis	80.7 (8.1)	80.0 (8.0)	0.080	80.1 (7.9)	80.1 (8.0)	0.003
Mean (SD) age at start of follow up	82.2 (7.9)	81.2 (7.9)	0.124	81.3 (7.9)	81.3 (7.9)	-0.002
Ethnicity						
White	14,371 (70.1)	202,834 (72.5)	-0.055	14,618 (71.3)	202,331 (72.4)	-0.024
Non-White	505 (2.5)	8878 (3.2)	-0.043	659 (3.2)	8742 (3.1)	0.005
Unknown	5632 (27.5)	67,889 (24.3)	0.073	5231 (25.5)	68,528 (24.5)	0.023
IMD quintile						
1 (least deprived)	4456 (21.7)	63,709 (22.8)	-0.025	4590 (22.4)	63,503 (22.7)	-0.008
2	4587 (22.4)	63,747 (22.8)	-0.010	4652 (22.7)	63,661 (22.8)	-0.002
3	4376 (21.3)	57,320 (20.5)	0.021	4176 (20.4)	57,474 (20.6)	-0.005
4	3738 (18.2)	51,028 (18.3)	-0.001	3773 (18.4)	51,026 (18.2)	0.004
5 (most deprived)	3338 (16.3)	43,614 (15.6)	0.019	3298 (16.1)	43,755 (15.6)	0.012
Unknown	13 (0.1)	183 (0.1)	-0.001	20 (0.1)	182 (0.1)	0.011
Lifestyle						
Smoking						
Current smoker	3531 (17.2)	49,060 (17.5)	-0.009	3644 (17.8)	49,009 (17.5)	0.006
Ex-smoker	8966 (43.7)	121,882 (43.6)	0.003	8799 (42.9)	121,887 (43.6)	-0.014
Never smoker	6658 (32.5)	92,116 (32.9)	-0.010	6794 (33.1)	92,025 (32.9)	0.005
Unknown	1353 (6.6)	16,543 (5.9)	0.028	1271 (6.2)	16,680 (6.0)	0.010
Alcohol use						
Non-drinker	3233 (15.8)	41,594 (14.9)	0.025	3120 (15.2)	41,770 (14.9)	0.008
Light drinker	2336 (11.4)	32,970 (11.8)	-0.013	2400 (11.7)	32,893 (11.8)	-0.002
Former drinker	851 (4.1)	10,270 (3.7)	0.025	758 (3.7)	10,361 (3.7)	0.000

	Before IPT weighting			After IPT weighting		
	Antipsychotics users (%) (n=20,508)	Matched comparators (%) (n=279,601)	Standardised difference	Antipsychotics users (%) (n=20,508)	Matched comparators (%) (n=279,601)	Standardised difference
Moderate drinker	7793 (38.0)	110,118 (39.4)	-0.028	7898 (38.5)	109,831 (39.3)	-0.016
Heavy drinker	798 (3.9)	11,718 (4.2)	-0.015	884 (4.3)	11,665 (4.2)	0.007
Unknown	5497 (26.8)	72,931 (26.1)	0.016	5448 (26.6)	73,081 (26.1)	0.010
Comorbidities [a]						
Hypertension	7971 (38.9)	113,073 (40.4)	-0.032	8192 (39.9)	112,760 (40.3)	-0.008
Diabetes	3086 (15.0)	43,737 (15.6)	-0.017	3178 (15.5)	43,621 (15.6)	-0.003
COPD	3576 (17.4)	45,187 (16.2)	0.034	3370 (16.4)	45,434 (16.2)	0.005
Rheumatoid arthritis	360 (1.8)	5049 (1.8)	-0.004	377 (1.8)	5039 (1.8)	0.003
Moderate/severe renal disease	4336 (21.1)	54,037 (19.3)	0.045	3906 (19.0)	54,375 (19.4)	-0.010
Moderate/severe liver disease	135 (0.7)	1748 (0.6)	0.004	122 (0.6)	1754 (0.6)	-0.004
Atrial fibrillation	2853 (13.9)	35,578 (12.7)	0.035	2630 (12.8)	35,806 (12.8)	0.001
Cancer	3245 (15.8)	36,241 (13.0)	0.082	2679 (13.1)	36,788 (13.2)	-0.003
Serious mental illness	444 (2.2)	2605 (0.9)	0.100	234 (1.1)	2852 (1.0)	0.010
inflammatory bowel disease	285 (1.4)	4030 (1.4)	-0.004	298 (1.5)	4022 (1.4)	0.001
Prescribed medications						
Antiplatelets	8530 (41.6)	114,425 (40.9)	0.014	8446 (41.2)	114,563 (41.0)	0.004
Oral anticoagulants	1462 (7.1)	19,470 (7.0)	0.006	1415 (6.9)	19,500 (7.0)	-0.003
ACE inhibitors or ARB	5322 (26.0)	80,942 (28.9)	-0.067	5855 (28.5)	80,365 (28.7)	-0.004
Alpha blockers	1518 (7.4)	21,318 (7.6)	-0.008	1541 (7.5)	21,272 (7.6)	-0.003
Beta blockers	3954 (19.3)	53,909 (19.3)	0.000	3951 (19.3)	53,911 (19.3)	0.000
Calcium channel blockers	3985 (19.4)	58,907 (21.1)	-0.041	4315 (21.0)	58,593 (21.0)	0.002
Diuretics	5975 (29.1)	81,105 (29.0)	0.003	6005 (29.3)	81,137 (29.0)	0.006
Lipid lowering drugs	6656 (32.5)	95,135 (34.0)	-0.033	6855 (33.4)	94,822 (33.9)	-0.010
Insulin and antidiabetic drugs	2118 (10.3)	30,621 (11.0)	-0.020	2237 (10.9)	30,500 (10.9)	0.000
NSAID	2467 (12.0)	32,660 (11.7)	0.011	2434 (11.9)	32,733 (11.7)	0.005
Antidepressants	6883 (33.6)	70,838 (25.3)	0.181	5361 (26.1)	72,418 (25.9)	0.005
Benzodiazepines	2961 (14.4)	17,723 (6.3)	0.268	1441 (7.0)	19,279 (6.9)	0.004
Lithium	63 (0.3)	687 (0.2)	0.012	59 (0.3)	700 (0.3)	0.007

[a] History of the condition

Supplementary Table S7. Baseline characteristics of antipsychotic users and matched comparators included in the analysis of pneumonia (CPRD Aurum and GOLD combined data)

	Before IPT weighting			After IPT weighting		
	Antipsychotics users (%) (n=29,509)	Matched comparators (%) (n=419,978)	Standardised difference	Antipsychotics users (%) (n=29,509)	Matched comparators (%) (n=419,978)	Standardised difference
Demographics						
Sex						
Male	10,810 (36.6)	148,662 (35.4)	0.026	10,454 (35.4)	149,002 (35.5)	-0.001
Female	18,699 (63.4)	271,316 (64.6)	-0.026	19,055 (64.6)	270,976 (64.5)	0.001
Age						
Mean (SD) age at dementia diagnosis	81.2 (8.1)	80.5 (7.9)	0.091	80.5 (7.9)	80.5 (8.0)	0.001
Mean (SD) age at start of follow up	82.8 (8.0)	81.8 (7.9)	0.123	81.9 (7.9)	81.9 (7.9)	-0.005
Ethnicity						
White	21,294 (72.2)	315,102 (75.0)	-0.065	21,818 (73.9)	314,275 (74.8)	-0.021
Non-White	569 (1.9)	10,635 (2.5)	-0.041	740 (2.5)	10,467 (2.5)	0.001
Unknown	7646 (25.9)	94,241 (22.4)	0.081	6951 (23.6)	95,236 (22.7)	0.021
IMD quintile						
1 (least deprived)	6420 (21.8)	95,724 (22.8)	-0.025	6648 (22.5)	95,435 (22.7)	-0.005
2	6627 (22.5)	96,314 (22.9)	-0.011	6720 (22.8)	96,179 (22.9)	-0.003
3	6314 (21.4)	86,100 (20.5)	0.022	5993 (20.3)	86,336 (20.6)	-0.006
4	5441 (18.4)	76,749 (18.3)	0.004	5455 (18.5)	76,800 (18.3)	0.005
5 (most deprived)	4686 (15.9)	64,774 (15.4)	0.013	4670 (15.8)	64,912 (15.5)	0.010
Unknown	21 (0.1)	317 (0.1)	-0.002	23 (0.1)	316 (0.1)	0.001
Lifestyle						
Smoking						
Current smoker	4942 (16.7)	72,278 (17.2)	-0.012	5120 (17.3)	72,159 (17.2)	0.004
Ex-smoker	12,757 (43.2)	182,463 (43.4)	-0.004	12,665 (42.9)	182,384 (43.4)	-0.010
Never smoker	9965 (33.8)	142,180 (33.9)	-0.002	10,028 (34.0)	142,160 (33.8)	0.003
Unknown	1845 (6.3)	23,057 (5.5)	0.032	1697 (5.7)	23,275 (5.5)	0.009
Alcohol use						
Non-drinker	4699 (15.9)	63,561 (15.1)	0.022	4564 (15.5)	63,790 (15.2)	0.008
Light drinker	3330 (11.3)	49,851 (11.9)	-0.018	3493 (11.8)	49,688 (11.8)	0.000
Former drinker	1262 (4.3)	15,926 (3.8)	0.025	1140 (3.9)	16,062 (3.8)	0.002
Moderate drinker	11,019 (37.3)	163,671 (39.0)	-0.034	11,262 (38.2)	163,194 (38.9)	-0.014

	Before IPT weighting			After IPT weighting		
	Antipsychotics users (%) (n=29,509)	Matched comparators (%) (n=419,978)	Standardised difference	Antipsychotics users (%) (n=29,509)	Matched comparators (%) (n=419,978)	Standardised difference
Heavy drinker	1226 (4.2)	18,668 (4.4)	-0.014	1313 (4.4)	18,589 (4.4)	0.001
Unknown	7973 (27.0)	108,301 (25.8)	0.028	7738 (26.2)	108,655 (25.9)	0.008
Comorbidities [a]						
Hypertension	11,733 (39.8)	172,588 (41.1)	-0.027	11,969 (40.6)	172,203 (41.0)	-0.009
Diabetes	4082 (13.8)	61,115 (14.6)	-0.021	4280 (14.5)	60,916 (14.5)	0.000
COPD	4913 (16.6)	67,144 (16.0)	0.018	4795 (16.2)	67,330 (16.0)	0.006
Rheumatoid arthritis	559 (1.9)	7854 (1.9)	0.002	567 (1.9)	7861 (1.9)	0.004
Moderate/severe renal disease	6331 (21.5)	83,975 (20.0)	0.036	5801 (19.7)	84,362 (20.1)	-0.011
Moderate/severe liver disease	195 (0.7)	2846 (0.7)	-0.002	199 (0.7)	2842 (0.7)	0.000
Atrial fibrillation	4018 (13.6)	53,009 (12.6)	0.029	3737 (12.7)	53,283 (12.7)	-0.001
Cancer	4636 (15.7)	55,408 (13.2)	0.072	3929 (13.3)	56,101 (13.4)	-0.001
Serious mental illness	657 (2.2)	4107 (1.0)	0.100	350 (1.2)	4466 (1.1)	0.010
inflammatory bowel disease	413 (1.4)	6195 (1.5)	-0.006	442 (1.5)	6176 (1.5)	0.002
Prescribed medications						
Antiplatelets	12,143 (41.2)	171,317 (40.8)	0.007	12,096 (41.0)	171,422 (40.8)	0.004
Oral anticoagulants	1982 (6.7)	27,695 (6.6)	0.005	1921 (6.5)	27,726 (6.6)	-0.004
ACE inhibitors or ARB	7438 (25.2)	118,872 (28.3)	-0.070	8225 (27.9)	118,008 (28.1)	-0.005
Alpha blockers	1932 (6.5)	29,043 (6.9)	-0.015	1997 (6.8)	28,936 (6.9)	-0.005
Beta blockers	5538 (18.8)	78,706 (18.7)	0.001	5499 (18.6)	78,712 (18.7)	-0.003
Calcium channel blockers	5599 (19.0)	87,703 (20.9)	-0.048	6097 (20.7)	87,168 (20.8)	-0.002
Diuretics	8496 (28.8)	120,659 (28.7)	0.001	8477 (28.7)	120,675 (28.7)	0.000
Lipid lowering drugs	9319 (31.6)	142,692 (34.0)	-0.051	9829 (33.3)	142,007 (33.8)	-0.011
Insulin and antidiabetic drugs	2741 (9.3)	41,994 (10.0)	-0.024	2943 (10.0)	41,797 (10.0)	0.001
NSAID	3656 (12.4)	49,784 (11.9)	0.016	3553 (12.0)	49,939 (11.9)	0.005
Antidepressants	10,424 (35.3)	112,120 (26.7)	0.187	8139 (27.6)	114,518 (27.3)	0.007
Benzodiazepines	4426 (15.0)	28,006 (6.7)	0.270	2184 (7.4)	30,322 (7.2)	0.006
Lithium	81 (0.3)	954 (0.2)	0.009	80 (0.3)	968 (0.2)	0.008
Immunosuppressants	141 (0.5)	2103 (0.5)	-0.003	146 (0.5)	2097 (0.5)	-0.001
Oral corticosteroids	1624 (5.5)	20,399 (4.9)	0.029	1416 (4.8)	20,575 (4.9)	-0.004
Inhaled corticosteroids	1504 (5.1)	22,673 (5.4)	-0.014	1619 (5.5)	22,593 (5.4)	0.005

[a] History of the condition

Supplementary Table S8. Baseline characteristics of antipsychotic users and matched comparators included in the analysis of acute kidney injury (CPRD Aurum and GOLD combined data)

	Before IPT weighting			After IPT weighting		
	Antipsychotics users (%) (n=30,198)	Matched comparators (%) (n=430,644)	Standardised difference	Antipsychotics users (%) (n=30,198)	Matched comparators (%) (n=430,644)	Standardised difference
Demographics						
Sex						
Male	11,146 (36.9)	153,086 (35.5)	0.028	10,737 (35.6)	153,469 (35.6)	-0.002
Female	19,052 (63.1)	277,558 (64.5)	-0.028	19,461 (64.4)	277,175 (64.4)	0.002
Age						
Mean (SD) age at dementia diagnosis	81.2 (8.1)	80.5 (8.0)	0.088	80.6 (7.9)	80.5 (8.0)	0.001
Mean (SD) age at start of follow up	82.8 (8.0)	81.9 (7.9)	0.119	81.9 (7.9)	81.9 (7.9)	-0.004
Ethnicity						
White	21,801 (72.2)	323,346 (75.1)	-0.066	22,335 (74.0)	322,493 (74.9)	-0.021
Non-White	580 (1.9)	10,536 (2.4)	-0.036	737 (2.4)	10,386 (2.4)	0.002
Unknown	7817 (25.9)	96,762 (22.5)	0.080	7125 (23.6)	97,766 (22.7)	0.021
IMD quintile						
1 (least deprived)	6577 (21.8)	97,917 (22.7)	-0.023	6784 (22.5)	97,643 (22.7)	-0.005
2	6807 (22.5)	98,416 (22.9)	-0.007	6846 (22.7)	98,321 (22.8)	-0.004
3	6432 (21.3)	88,330 (20.5)	0.019	6142 (20.3)	88,544 (20.6)	-0.006
4	5562 (18.4)	78,968 (18.3)	0.002	5586 (18.5)	78,997 (18.3)	0.004
5 (most deprived)	4799 (15.9)	66,677 (15.5)	0.011	4818 (16.0)	66,806 (15.5)	0.012
Unknown	21 (0.1)	336 (0.1)	-0.003	23 (0.1)	334 (0.1)	0.000
Lifestyle						
Smoking						
Current smoker	5131 (17.0)	74,541 (17.3)	-0.008	5270 (17.5)	74,460 (17.3)	0.004
Ex-smoker	13,001 (43.1)	187,261 (43.5)	-0.008	12,977 (43.0)	187,130 (43.5)	-0.010
Never smoker	10,159 (33.6)	145,100 (33.7)	-0.001	10,207 (33.8)	145,086 (33.7)	0.002
Unknown	1897 (6.3)	23,742 (5.5)	0.033	1744 (5.8)	23,968 (5.6)	0.009
Alcohol use						
Non-drinker	4758 (15.8)	65,071 (15.1)	0.018	4653 (15.4)	65,262 (15.2)	0.007
Light drinker	3396 (11.2)	50,752 (11.8)	-0.017	3540 (11.7)	50,598 (11.7)	-0.001

	Before IPT weighting			After IPT weighting		
	Antipsychotics users (%) (n=30,198)	Matched comparators (%) (n=430,644)	Standardised difference	Antipsychotics users (%) (n=30,198)	Matched comparators (%) (n=430,644)	Standardised difference
Former drinker	1292 (4.3)	16,209 (3.8)	0.026	1154 (3.8)	16,355 (3.8)	0.001
Moderate drinker	11,221 (37.2)	167,120 (38.8)	-0.034	11,482 (38.0)	166,628 (38.7)	-0.014
Heavy drinker	1275 (4.2)	19,371 (4.5)	-0.014	1369 (4.5)	19,296 (4.5)	0.003
Unknown	8256 (27.3)	112,121 (26.0)	0.029	8000 (26.5)	112,506 (26.1)	0.008
Comorbidities [a]						
Hypertension	11,772 (39.0)	175,181 (40.7)	-0.035	12,117 (40.1)	174,684 (40.6)	-0.009
Diabetes	4071 (13.5)	61,405 (14.3)	-0.023	4287 (14.2)	61,182 (14.2)	0.000
COPD	5463 (18.1)	73,195 (17.0)	0.029	5208 (17.2)	73,507 (17.1)	0.005
Rheumatoid arthritis	602 (2.0)	8308 (1.9)	0.005	600 (2.0)	8327 (1.9)	0.004
Moderate/severe renal disease	5885 (19.5)	81,081 (18.8)	0.017	5597 (18.5)	81,256 (18.9)	-0.008
Moderate/severe liver disease	196 (0.6)	2874 (0.7)	-0.002	204 (0.7)	2870 (0.7)	0.001
Atrial fibrillation	4181 (13.8)	55,798 (13.0)	0.026	3918 (13.0)	56,048 (13.0)	-0.001
Cancer	4765 (15.8)	57,063 (13.3)	0.072	4033 (13.4)	57,773 (13.4)	-0.002
Serious mental illness	671 (2.2)	4151 (1.0)	0.101	355 (1.2)	4522 (1.1)	0.010
Prescribed medications						
Antiplatelets	12,459 (41.2)	176,052 (40.9)	0.008	12,407 (41.1)	176,165 (40.9)	0.004
Oral anticoagulants	2028 (6.7)	28,871 (6.7)	0.000	2003 (6.6)	28,872 (6.7)	-0.003
ACE inhibitors or ARB	7467 (24.7)	121,338 (28.2)	-0.078	8387 (27.8)	120,356 (27.9)	-0.004
Alpha blockers	1933 (6.4)	29,161 (6.8)	-0.015	1991 (6.6)	29,049 (6.7)	-0.006
Beta blockers	5498 (18.2)	79,544 (18.5)	-0.007	5557 (18.4)	79,470 (18.5)	-0.001
Calcium channel blockers	5626 (18.6)	88,812 (20.6)	-0.050	6169 (20.4)	88,243 (20.5)	-0.002
Diuretics	8660 (28.7)	124,026 (28.8)	-0.003	8689 (28.8)	123,991 (28.8)	0.000
Lipid lowering drugs	9400 (31.1)	145,148 (33.7)	-0.055	10,001 (33.1)	144,398 (33.5)	-0.009
Insulin and antidiabetic drugs	2722 (9.0)	42,037 (9.8)	-0.026	2934 (9.7)	41,824 (9.7)	0.000
NSAID	3734 (12.4)	50,907 (11.8)	0.017	3627 (12.0)	51,068 (11.9)	0.005
Antidepressants	10,769 (35.7)	115,861 (26.9)	0.190	8399 (27.8)	118,351 (27.5)	0.007
Benzodiazepines	4574 (15.1)	29,245 (6.8)	0.270	2275 (7.5)	31,622 (7.3)	0.006
Lithium	84 (0.3)	969 (0.2)	0.011	83 (0.3)	985 (0.2)	0.009

[a] History of the condition

Supplementary Table S9. Baseline characteristics of antipsychotic users and matched comparators included in the analysis of unrelated outcomes (appendicitis and cholecystitis, CPRD Aurum and GOLD combined data)

	Before IPT weighting			After IPT weighting		
	Antipsychotics users (%) (n=30,408)	Matched comparators (%) (n=432,605)	Standardised difference	Antipsychotics users (%) (n=30,408)	Matched comparators (%) (n=432,605)	Standardised difference
Demographics						
Sex						
Male	11,508 (37.8)	156,977 (36.3)	0.032	11,031 (36.3)	157,419 (36.4)	-0.002
Female	18,900 (62.2)	275,628 (63.7)	-0.032	19,377 (63.7)	275,186 (63.6)	0.002
Age						
Mean (SD) age at dementia diagnosis	81.4 (8.1)	80.6 (7.9)	0.097	80.7 (7.9)	80.7 (8.0)	0.002
Mean (SD) age at start of follow up	83.1 (7.9)	82.0 (7.9)	0.130	82.1 (7.9)	82.1 (7.9)	-0.004
Ethnicity						
White	22,256 (73.2)	327,288 (75.7)	-0.056	22,656 (74.5)	326,549 (75.5)	-0.023
Non-White	647 (2.1)	11,772 (2.7)	-0.039	826 (2.7)	11,602 (2.7)	0.002
Unknown	7505 (24.7)	93,545 (21.6)	0.073	6925 (22.8)	94,454 (21.8)	0.023
IMD quintile						
1 (least deprived)	6505 (21.4)	97,088 (22.4)	-0.025	6735 (22.1)	96,787 (22.4)	-0.005
2	6789 (22.3)	98,184 (22.7)	-0.009	6831 (22.5)	98,069 (22.7)	-0.005
3	6466 (21.3)	88,305 (20.4)	0.021	6176 (20.3)	88,540 (20.5)	-0.004
4	5625 (18.5)	79,852 (18.5)	0.001	5668 (18.6)	79,869 (18.5)	0.005
5 (most deprived)	5001 (16.4)	68,826 (15.9)	0.015	4973 (16.4)	68,991 (15.9)	0.011
Unknown	22 (0.1)	350 (0.1)	-0.003	24 (0.1)	348 (0.1)	0.000
Lifestyle						
Smoking						
Current smoker	5191 (17.1)	75,408 (17.4)	-0.010	5348 (17.6)	75,318 (17.4)	0.005
Ex-smoker	13,315 (43.8)	190,360 (44.0)	-0.004	13,213 (43.5)	190,276 (44.0)	-0.011
Never smoker	10,062 (33.1)	143,654 (33.2)	-0.002	10,132 (33.3)	143,621 (33.2)	0.003
Unknown	1840 (6.1)	23,183 (5.4)	0.030	1715 (5.6)	23,390 (5.4)	0.010
Alcohol use						
Non-drinker	4875 (16.0)	66,580 (15.4)	0.018	4783 (15.7)	66,772 (15.4)	0.008
Light drinker	3377 (11.1)	50,763 (11.7)	-0.020	3543 (11.7)	50,582 (11.7)	-0.001

	Before IPT weighting			After IPT weighting		
	Antipsychotics users (%) (n=30,408)	Matched comparators (%) (n=432,605)	Standardised difference	Antipsychotics users (%) (n=30,408)	Matched comparators (%) (n=432,605)	Standardised difference
Former drinker	1313 (4.3)	16,440 (3.8)	0.026	1178 (3.9)	16,589 (3.8)	0.002
Moderate drinker	11,314 (37.2)	167,490 (38.7)	-0.031	11,518 (37.9)	167,030 (38.6)	-0.015
Heavy drinker	1306 (4.3)	19,655 (4.5)	-0.012	1393 (4.6)	19,587 (4.5)	0.003
Unknown	8223 (27.0)	111,677 (25.8)	0.028	7993 (26.3)	112,044 (25.9)	0.009
Comorbidities [a]						
Hypertension	12,187 (40.1)	179,469 (41.5)	-0.029	12,457 (41.0)	179,053 (41.4)	-0.009
Diabetes	4401 (14.5)	65,415 (15.1)	-0.018	4578 (15.1)	65,228 (15.1)	-0.001
COPD	5726 (18.8)	76,040 (17.6)	0.032	5417 (17.8)	76,399 (17.7)	0.004
Rheumatoid arthritis	611 (2.0)	8358 (1.9)	0.006	599 (2.0)	8380 (1.9)	0.002
Moderate/severe renal disease	6956 (22.9)	91,852 (21.2)	0.040	6363 (20.9)	92,306 (21.3)	-0.010
Moderate/severe liver disease	231 (0.8)	3198 (0.7)	0.002	226 (0.7)	3204 (0.7)	0.000
Atrial fibrillation	4490 (14.8)	58,323 (13.5)	0.037	4108 (13.5)	58,687 (13.6)	-0.002
Cancer	4930 (16.2)	58,147 (13.4)	0.078	4128 (13.6)	58,933 (13.6)	-0.001
Serious mental illness	669 (2.2)	4200 (1.0)	0.099	360 (1.2)	4564 (1.1)	0.010
Prescribed medications						
Antiplatelets	12,700 (41.8)	177,902 (41.1)	0.013	12,582 (41.4)	178,093 (41.2)	0.004
Oral anticoagulants	2252 (7.4)	31,178 (7.2)	0.008	2159 (7.1)	31,230 (7.2)	-0.005
ACE inhibitors or ARB	7773 (25.6)	124,437 (28.8)	-0.072	8628 (28.4)	123,517 (28.6)	-0.004
Alpha blockers	2096 (6.9)	31,083 (7.2)	-0.011	2146 (7.1)	30,994 (7.2)	-0.004
Beta blockers	5875 (19.3)	82,688 (19.1)	0.005	5774 (19.0)	82,745 (19.1)	-0.003
Calcium channel blockers	5775 (19.0)	90,690 (21.0)	-0.049	6332 (20.8)	90,124 (20.8)	0.000
Diuretics	9018 (29.7)	126,896 (29.3)	0.007	8937 (29.4)	126,987 (29.4)	0.001
Lipid lowering drugs	9835 (32.3)	150,889 (34.9)	-0.054	10,401 (34.2)	150,142 (34.7)	-0.011
Insulin and antidiabetic drugs	2976 (9.8)	45,083 (10.4)	-0.021	3162 (10.4)	44,901 (10.4)	0.001
NSAID	3776 (12.4)	50,740 (11.7)	0.021	3615 (11.9)	50,940 (11.8)	0.003
Antidepressants	10,876 (35.8)	116,700 (27.0)	0.190	8475 (27.9)	119,217 (27.6)	0.007
Benzodiazepines	4600 (15.1)	28,920 (6.7)	0.273	2269 (7.5)	31,341 (7.2)	0.007
Lithium	79 (0.3)	931 (0.2)	0.009	77 (0.3)	944 (0.2)	0.007

[a] History of the condition

Supplementary Table S10. Hazard ratios (adjusted for IPT weights) of adverse outcomes associated with current, recent, and past use of typical and atypical antipsychotics; with current use being defined as 90 days from the date of an antipsychotic prescription, recent use as up to 180 days after current use ended, and past use as after recent use.

	Antipsychotic type [a]	Current use	Recent use	Past use	Any use
Stroke	Typical vs unexposed	1.83 (1.66 to 2.03)	1.73 (1.41 to 2.11)	1.09 (0.83 to 1.43)	1.70 (1.56 to 1.86)
	Atypical vs unexposed	1.49 (1.38 to 1.61)	1.34 (1.09 to 1.64)	1.21 (0.95 to 1.54)	1.44 (1.34 to 1.54)
	Typical vs atypical	1.23 (1.09 to 1.40)	1.29 (0.97 to 1.72)	0.90 (0.63 to 1.30)	1.18 (1.06 to 1.32)
VTE [b]	Typical vs unexposed	1.52 (1.24 to 1.86)	1.27 (0.86 to 1.88)	0.98 (0.62 to 1.57)	1.38 (1.16 to 1.64)
	Atypical vs unexposed	1.64 (1.45 to 1.86)	1.85 (1.38 to 2.48)	0.83 (0.53 to 1.32)	1.58 (1.41 to 1.76)
	Typical vs atypical	0.92 (0.73 to 1.17)	0.69 (0.42 to 1.12)	1.18 (0.61 to 2.27)	0.88 (0.71 to 1.07)
Myocardial infarction	Typical vs unexposed	1.41 (1.17 to 1.69)	1.38 (0.98 to 1.93)	0.97 (0.64 to 1.47)	1.32 (1.14 to 1.54)
	Atypical vs unexposed	1.24 (1.08 to 1.41)	1.08 (0.76 to 1.52)	0.96 (0.66 to 1.40)	1.19 (1.06 to 1.33)
	Typical vs atypical	1.14 (0.91 to 1.43)	1.28 (0.79 to 2.07)	1.01 (0.58 to 1.76)	1.11 (0.92 to 1.35)
Heart failure	Typical vs unexposed	1.44 (1.26 to 1.63)	0.99 (0.75 to 1.32)	0.96 (0.71 to 1.29)	1.27 (1.14 to 1.42)
	Atypical vs unexposed	1.21 (1.10 to 1.33)	0.78 (0.60 to 1.02)	0.81 (0.59 to 1.09)	1.11 (1.02 to 1.21)
	Typical vs atypical	1.18 (1.01 to 1.39)	1.27 (0.86 to 1.88)	1.19 (0.78 to 1.82)	1.14 (0.99 to 1.31)
Ventricular arrhythmia	Typical vs unexposed	0.79 (0.39 to 1.59)	1.79 (0.59 to 5.41)	0.20 (0.03 to 1.56)	0.84 (0.47 to 1.51)
	Atypical vs unexposed	0.76 (0.47 to 1.22)	0.31 (0.04 to 2.25)	1.85 (0.71 to 4.80)	0.81 (0.53 to 1.23)
	Typical vs atypical	1.04 (0.45 to 2.44)	5.82 (0.60 to 56.49)	0.11 (0.01 to 1.04)	1.04 (0.51 to 2.14)
Fractures	Typical vs unexposed	1.63 (1.48 to 1.81)	1.32 (1.07 to 1.62)	1.02 (0.80 to 1.29)	1.47 (1.35 to 1.60)
	Atypical vs unexposed	1.34 (1.25 to 1.44)	1.28 (1.07 to 1.54)	1.10 (0.88 to 1.38)	1.31 (1.23 to 1.40)
	Typical vs atypical	1.22 (1.08 to 1.38)	1.03 (0.78 to 1.36)	0.92 (0.66 to 1.28)	1.12 (1.01 to 1.25)
Pneumonia	Typical vs unexposed	3.33 (3.13 to 3.55)	1.82 (1.58 to 2.10)	1.51 (1.28 to 1.78)	2.72 (2.57 to 2.87)
	Atypical vs unexposed	1.73 (1.65 to 1.83)	1.63 (1.42 to 1.86)	1.43 (1.24 to 1.65)	1.69 (1.61 to 1.77)
	Typical vs atypical	1.92 (1.77 to 2.08)	1.12 (0.92 to 1.36)	1.05 (0.85 to 1.31)	1.61 (1.50 to 1.73)

AKI [c]	Typical vs unexposed	1.99 (1.75 to 2.25)	1.10 (0.83 to 1.46)	1.43 (1.11 to 1.85)	1.70 (1.53 to 1.89)
	Atypical vs unexposed	1.62 (1.50 to 1.75)	1.26 (1.01 to 1.56)	0.99 (0.78 to 1.26)	1.50 (1.40 to 1.61)
	Typical vs atypical	1.22 (1.05 to 1.42)	0.88 (0.61 to 1.25)	1.45 (1.02 to 2.06)	1.13 (1.00 to 1.28)
Unrelated outcome [d]	Typical vs unexposed	0.42 (0.17 to 1.01)	0.90 (0.27 to 3.03)	0.64 (0.15 to 2.68)	0.55 (0.29 to 1.06)
	Atypical vs unexposed	1.05 (0.71 to 1.55)	1.84 (0.86 to 3.97)	3.29 (1.64 to 6.59)	1.32 (0.97 to 1.80)
	Typical vs atypical	0.40 (0.15 to 1.05)	0.49 (0.12 to 2.05)	0.19 (0.04 to 0.96)	0.42 (0.20 to 0.86)

[a] Patients who were prescribed both typical and atypical antipsychotics on the same day were censored.
[b] VTE - Venous thromboembolism
[c] AKI - Acute kidney injury
[d] Unrelated outcome - Appendicitis and cholecystitis

Supplementary Table S11. Hazard ratios (adjusted for IPT weights) of adverse outcomes associated with the use of risperidone, quetiapine, haloperidol, and other antipsychotics; with current use being defined as 90 days from the date of an antipsychotic prescription, recent use as up to 180 days after current use ended, and past use as after recent use.

	Antipsychotic drug substance [a,b]	Current use	Recent use	Past use	Any use
Stroke	Risperidone vs unexposed	1.76 (1.58 to 1.96)	1.25 (0.95 to 1.66)	1.34 (0.97 to 1.84)	1.64 (1.49 to 1.80)
	Quetiapine vs unexposed	1.13 (0.97 to 1.32)	1.29 (0.86 to 1.94)	1.06 (0.63 to 1.76)	1.14 (0.99 to 1.31)
	Haloperidol vs unexposed	1.85 (1.58 to 2.17)	1.75 (1.30 to 2.35)	1.07 (0.70 to 1.64)	1.71 (1.49 to 1.95)
	Others vs unexposed	1.69 (1.52 to 1.87)	1.71 (1.36 to 2.14)	1.11 (0.83 to 1.50)	1.62 (1.48 to 1.77)
	Haloperidol vs risperidone	1.05 (0.87 to 1.28)	1.40 (0.93 to 2.10)	0.80 (0.47 to 1.37)	1.04 (0.88 to 1.23)
	Haloperidol vs quetiapine	1.63 (1.31 to 2.04)	1.35 (0.82 to 2.24)	1.02 (0.52 to 1.97)	1.50 (1.23 to 1.81)
	Quetiapine vs risperidone	0.64 (0.53 to 0.78)	1.03 (0.63 to 1.69)	0.79 (0.43 to 1.44)	0.70 (0.59 to 0.83)
VTE [c]	Risperidone vs unexposed	2.04 (1.73 to 2.40)	2.31 (1.61 to 3.32)	0.87 (0.46 to 1.64)	1.93 (1.66 to 2.23)
	Quetiapine vs unexposed	1.00 (0.76 to 1.32)	1.44 (0.77 to 2.68)	0.88 (0.40 to 1.91)	1.04 (0.82 to 1.32)
	Haloperidol vs unexposed	1.99 (1.49 to 2.66)	1.85 (1.11 to 3.06)	1.17 (0.61 to 2.21)	1.81 (1.43 to 2.29)
	Others vs unexposed	1.52 (1.25 to 1.86)	0.93 (0.54 to 1.58)	0.80 (0.44 to 1.45)	1.33 (1.11 to 1.59)
	Haloperidol vs risperidone	0.98 (0.70 to 1.37)	0.80 (0.43 to 1.48)	1.34 (0.54 to 3.31)	0.94 (0.71 to 1.24)
	Haloperidol vs quetiapine	1.99 (1.33 to 2.97)	1.28 (0.58 to 2.85)	1.33 (0.49 to 3.64)	1.74 (1.24 to 2.44)
	Quetiapine vs risperidone	0.49 (0.36 to 0.68)	0.62 (0.30 to 1.28)	1.01 (0.37 to 2.76)	0.54 (0.41 to 0.71)
Myocardial infarction	Risperidone vs unexposed	1.24 (1.02 to 1.51)	0.96 (0.58 to 1.60)	1.23 (0.77 to 1.96)	1.20 (1.01 to 1.43)
	Quetiapine vs unexposed	1.05 (0.83 to 1.33)	1.44 (0.80 to 2.57)	0.78 (0.37 to 1.64)	1.06 (0.86 to 1.31)
	Haloperidol vs unexposed	1.38 (1.02 to 1.86)	1.01 (0.59 to 1.73)	0.61 (0.29 to 1.26)	1.15 (0.90 to 1.47)
	Others vs unexposed	1.45 (1.22 to 1.73)	1.45 (0.98 to 2.14)	1.09 (0.68 to 1.73)	1.40 (1.20 to 1.63)
	Haloperidol vs risperidone	1.11 (0.78 to 1.59)	1.05 (0.50 to 2.19)	0.49 (0.21 to 1.17)	0.95 (0.71 to 1.29)
	Haloperidol vs quetiapine	1.32 (0.90 to 1.93)	0.70 (0.32 to 1.55)	0.78 (0.27 to 2.22)	1.08 (0.78 to 1.50)
	Quetiapine vs risperidone	0.84 (0.62 to 1.15)	1.49 (0.69 to 3.23)	0.63 (0.26 to 1.52)	0.88 (0.67 to 1.15)
Hear failure	Risperidone vs unexposed	1.29 (1.13 to 1.47)	0.66 (0.45 to 0.97)	0.65 (0.42 to 1.01)	1.12 (0.99 to 1.26)
	Quetiapine vs unexposed	1.09 (0.92 to 1.30)	0.68 (0.39 to 1.19)	0.80 (0.44 to 1.46)	1.02 (0.87 to 1.20)
	Haloperidol vs unexposed	1.52 (1.24 to 1.86)	1.35 (0.92 to 1.97)	1.55 (1.05 to 2.27)	1.49 (1.27 to 1.75)
	Others vs unexposed	1.32 (1.15 to 1.50)	0.93 (0.67 to 1.29)	0.80 (0.56 to 1.15)	1.18 (1.05 to 1.33)
	Haloperidol vs risperidone	1.18 (0.93 to 1.50)	2.03 (1.19 to 3.48)	2.37 (1.32 to 4.26)	1.33 (1.09 to 1.63)
	Haloperidol vs quetiapine	1.39 (1.07 to 1.81)	1.98 (1.01 to 3.89)	1.93 (0.95 to 3.93)	1.46 (1.17 to 1.84)
	Quetiapine vs risperidone	0.85 (0.68 to 1.05)	1.03 (0.52 to 2.02)	1.23 (0.58 to 2.58)	0.91 (0.75 to 1.11)
Ventricular arrhythmia	Risperidone vs unexposed	0.95 (0.53 to 1.72)	0.00 (0.00 to 0.00)	1.68 (0.52 to 5.45)	0.90 (0.53 to 1.53)
	Quetiapine vs unexposed	0.44 (0.15 to 1.26)	1.58 (0.21 to 11.94)	3.18 (0.52 to 19.52)	0.72 (0.32 to 1.59)

	Haloperidol vs unexposed	0.73 (0.19 to 2.86)	2.67 (0.59 to 11.97)	0.00 (0.00 to 0.00)	0.83 (0.31 to 2.25)
	Others vs unexposed	0.84 (0.42 to 1.69)	0.98 (0.17 to 5.68)	0.38 (0.05 to 3.08)	0.82 (0.43 to 1.54)
	Haloperidol vs risperidone	0.77 (0.17 to 3.40)	2.51×10^{14} (4.96×10^{13} to 1.27×10^{15})	0.00 (0.00 to 0.00)	0.92 (0.30 to 2.86)
	Haloperidol vs quetiapine	1.67 (0.30 to 9.38)	1.69 (0.14 to 20.99)	0.00 (0.00 to 0.00)	1.16 (0.32 to 4.16)
	Quetiapine vs risperidone	0.46 (0.14 to 1.54)	1.49×10^{14} (1.79×10^{13} to 1.23×10^{15})	1.89 (0.22 to 16.46)	0.80 (0.31 to 2.08)
Fracture	Risperidone vs unexposed	1.31 (1.17 to 1.47)	1.25 (0.96 to 1.62)	0.99 (0.72 to 1.36)	1.26 (1.15 to 1.40)
	Quetiapine vs unexposed	1.25 (1.10 to 1.42)	1.35 (0.96 to 1.89)	1.08 (0.70 to 1.68)	1.25 (1.11 to 1.40)
	Haloperidol vs unexposed	1.87 (1.60 to 2.18)	1.13 (0.80 to 1.59)	0.93 (0.64 to 1.35)	1.54 (1.34 to 1.76)
	Others vs unexposed	1.51 (1.36 to 1.67)	1.39 (1.12 to 1.74)	1.19 (0.92 to 1.53)	1.45 (1.33 to 1.58)
	Haloperidol vs risperidone	1.42 (1.17 to 1.73)	0.90 (0.59 to 1.40)	0.94 (0.58 to 1.53)	1.22 (1.03 to 1.44)
	Haloperidol vs quetiapine	1.49 (1.22 to 1.83)	0.84 (0.52 to 1.36)	0.86 (0.48 to 1.53)	1.23 (1.03 to 1.47)
	Quetiapine vs risperidone	0.96 (0.81 to 1.13)	1.08 (0.70 to 1.66)	1.09 (0.64 to 1.88)	0.99 (0.85 to 1.15)
Pneumonia	Risperidone vs unexposed	1.98 (1.84 to 2.12)	1.54 (1.28 to 1.87)	1.39 (1.14 to 1.70)	1.84 (1.73 to 1.96)
	Quetiapine vs unexposed	1.42 (1.28 to 1.56)	1.77 (1.39 to 2.26)	1.59 (1.22 to 2.07)	1.47 (1.35 to 1.60)
	Haloperidol vs unexposed	3.58 (3.26 to 3.92)	2.14 (1.75 to 2.61)	1.82 (1.46 to 2.28)	2.98 (2.75 to 3.23)
	Others vs unexposed	2.43 (2.27 to 2.60)	1.59 (1.34 to 1.89)	1.30 (1.06 to 1.58)	2.14 (2.01 to 2.27)
	Haloperidol vs risperidone	1.81 (1.61 to 2.04)	1.38 (1.05 to 1.82)	1.31 (0.97 to 1.76)	1.62 (1.46 to 1.79)
	Haloperidol vs quetiapine	2.53 (2.21 to 2.89)	1.20 (0.88 to 1.65)	1.15 (0.81 to 1.63)	2.03 (1.81 to 2.28)
	Quetiapine vs risperidone	0.72 (0.63 to 0.81)	1.15 (0.84 to 1.56)	1.14 (0.82 to 1.59)	0.80 (0.72 to 0.89)
AKI [d]	Risperidone vs unexposed	1.72 (1.54 to 1.91)	1.28 (0.96 to 1.71)	1.03 (0.74 to 1.42)	1.57 (1.43 to 1.73)
	Quetiapine vs unexposed	1.38 (1.20 to 1.60)	1.26 (0.83 to 1.91)	1.05 (0.68 to 1.63)	1.33 (1.17 to 1.52)
	Haloperidol vs unexposed	2.23 (1.85 to 2.68)	1.38 (0.95 to 2.01)	1.67 (1.21 to 2.31)	1.93 (1.67 to 2.24)
	Others vs unexposed	1.79 (1.58 to 2.03)	0.98 (0.69 to 1.39)	1.03 (0.73 to 1.45)	1.55 (1.39 to 1.73)
	Haloperidol vs risperidone	1.30 (1.05 to 1.61)	1.08 (0.67 to 1.72)	1.63 (1.03 to 2.57)	1.23 (1.03 to 1.47)
	Haloperidol vs quetiapine	1.61 (1.28 to 2.03)	1.10 (0.63 to 1.92)	1.59 (0.92 to 2.74)	1.45 (1.19 to 1.77)
	Quetiapine vs risperidone	0.81 (0.67 to 0.96)	0.98 (0.59 to 1.63)	1.02 (0.59 to 1.76)	0.85 (0.72 to 1.00)
Unrelated outcome [e]	Risperidone vs unexposed	0.61 (0.30 to 1.21)	2.66 (1.16 to 6.08)	2.34 (0.81 to 6.72)	1.03 (0.64 to 1.66)
	Quetiapine vs unexposed	1.45 (0.77 to 2.73)	1.54 (0.21 to 11.56)	5.72 (1.93 to 16.97)	1.81 (1.08 to 3.03)
	Haloperidol vs unexposed	0.43 (0.11 to 1.74)	1.14 (0.22 to 5.79)	0.76 (0.11 to 5.28)	0.65 (0.25 to 1.65)
	Others vs unexposed	0.98 (0.54 to 1.77)	0.46 (0.07 to 3.01)	0.82 (0.18 to 3.65)	0.87 (0.51 to 1.48)
	Haloperidol vs risperidone	0.71 (0.15 to 3.39)	0.43 (0.07 to 2.65)	0.33 (0.04 to 2.96)	0.63 (0.22 to 1.80)
	Haloperidol vs quetiapine	0.30 (0.06 to 1.38)	0.74 (0.06 to 9.84)	0.13 (0.01 to 1.23)	0.36 (0.12 to 1.04)
	Quetiapine vs risperidone	2.39 (0.93 to 6.13)	0.58 (0.07 to 5.12)	2.45 (0.54 to 11.16)	1.76 (0.88 to 3.55)

[a] The ‘others’ category include all antipsychotics except risperidone, quetiapine, and haloperidol.
[b] Patients who were prescribed more than one type of drugs – risperidone, haloperidol, quetiapine, or ‘others’ - on the same day were censored.
[c] VTE - Venous thromboembolism
[d] AKI - Acute kidney injury
[e] Unrelated outcome - Appendicitis and cholecystitis

Supplementary Table S12. Subhazard ratios (adjusted for IPT weights) of adverse outcomes associated with current, recent, and past antipsychotic use stratified by follow-up period; with current use being defined as the first 90 days from the date of an antipsychotic prescription, recent use as up to 180 days after current use ended, and past use as after recent use. [a]

Subhazard ratio (95% confidence interval)	Antipsychotic use	Follow-up period				
		0-7 days	8-30 days	31-180 days	181-365 days	366 days-2 years
Stroke	Current	3.74 (2.92 to 4.78)	1.60 (1.30 to 1.97)	1.50 (1.35 to 1.68)	1.54 (1.34 to 1.76)	1.51 (1.33 to 1.70)
	Recent	-	-	1.76 (1.37 to 2.26)	1.56 (1.24 to 1.96)	1.35 (0.99 to 1.84)
	Past	-	-	-	1.62 (1.11 to 2.36)	1.05 (0.85 to 1.29)
VTE [b]	Current	2.12 (1.16 to 3.90)	1.88 (1.31 to 2.71)	1.63 (1.36 to 1.97)	1.27 (1.00 to 1.62)	1.47 (1.20 to 1.80)
	Recent	-	-	2.03 (1.36 to 3.02)	1.19 (0.79 to 1.79)	1.50 (0.94 to 2.40)
	Past	-	-	-	0.60 (0.22 to 1.61)	0.95 (0.67 to 1.36)
Myocardial infarction	Current	2.26 (1.34 to 3.82)	1.59 (1.12 to 2.25)	1.23 (1.02 to 1.48)	1.34 (1.08 to 1.67)	1.00 (0.80 to 1.25)
	Recent	-	-	0.87 (0.52 to 1.48)	1.29 (0.91 to 1.82)	1.13 (0.71 to 1.82)
	Past	-	-	-	1.26 (0.68 to 2.33)	0.96 (0.70 to 1.33)
Heart failure	Current	2.72 (2.01 to 3.69)	1.91 (1.53 to 2.38)	1.25 (1.10 to 1.42)	1.08 (0.91 to 1.27)	0.86 (0.73 to 1.01)
	Recent	-	-	0.97 (0.71 to 1.34)	0.73 (0.54 to 0.98)	0.74 (0.49 to 1.13)
	Past	-	-	-	1.23 (0.80 to 1.90)	0.79 (0.62 to 1.01)
Fracture	Current	2.23 (1.63 to 3.05)	1.47 (1.19 to 1.83)	1.30 (1.17 to 1.45)	1.25 (1.10 to 1.43)	1.46 (1.30 to 1.63)
	Recent	-	-	1.10 (0.83 to 1.46)	1.22 (0.98 to 1.51)	1.48 (1.14 to 1.91)
	Past	-	-	-	1.03 (0.68 to 1.56)	1.07 (0.88 to 1.29)
Pneumonia	Current	9.21 (7.96 to 10.6)	3.30 (2.94 to 3.71)	1.86 (1.73 to 1.99)	1.61 (1.48 to 1.77)	1.55 (1.43 to 1.68)
	Recent	-	-	1.86 (1.56 to 2.21)	1.59 (1.36 to 1.84)	1.28 (1.04 to 1.57)
	Past	-	-	-	1.39 (1.07 to 1.80)	1.41 (1.24 to 1.59)
AKI [c]	Current	3.83 (2.86 to 5.11)	2.36 (1.93 to 2.88)	1.69 (1.52 to 1.88)	1.03 (0.88 to 1.20)	1.03 (0.90 to 1.19)
	Recent	-	-	1.32 (0.99 to 1.76)	0.84 (0.64 to 1.11)	0.77 (0.52 to 1.13)
	Past	-	-	-	1.48 (1.02 to 2.15)	0.96 (0.78 to 1.18)

[a] Not reported for ventricular arrhythmia and unrelated outcome (appendicitis and cholecystitis) because of small number of cases.
[b] VTE - Venous thromboembolism
[c] AKI - Acute kidney injury

Supplementary Table S13. Sex-specific incidence rates of adverse outcomes associated with antipsychotic use during the first 2 years of follow-up period

		Males [a]			Females [b]			Male vs female incidence rate ratios (IRRs)
		Number of outcomes [c]	Person-years [c]	Incidence rates (95% CI) (per 10,000 person-years)	Number of outcomes [c]	Person-years [c]	Incidence rates (95% CI) (per 10,000 person-years)	
Stroke	Antipsychotics user	487	8051	604.9 (553.5 to 661,1)	999	16,345	611.2 (574.4 to 650.3)	0.99 (0 .89 to 1.10)
	Matched comparators	2201	55,044	399.9 (383.5 to 416.9)	6744	196,330	343.5 (335.4 to 351.8)	1.16 (1.11 to 1.22)
VTE [d]	Antipsychotics user	167	10,609	157.4 (135.3 to 183.2)	324	19,606	165.3 (148.2 to 184.3)	0.95 (0.79 to 1.15)
	Matched comparators	750	78,309	95.8 (89.2 to 102.9)	2466	234,718	105.1 (101.0 to 109.3)	0.91 (0.84 to 0.99)
Myocardial infarction	Antipsychotics user	201	9858	203.9 (177.6 to 234.1)	297	19544	152.0 (135.6 to 170.3)	1.34 (1.12 to 1.61)
	Matched comparators	1056	68,354	154.5 (145.4 to 164.1)	2860	238,282	120.0 (115.7 to 124.5)	1.29 (1.20 to 1.38)
Heart failure	Antipsychotics user	374	9892	378.1 (341.6 to 418.4)	600	18,593	322.7 (297.9 to 349.6)	1.17 (1.03 to 1.34)
	Matched comparators	2294	70,890	323.6 (310.6 to 337.1)	5680	220,559	257.5 (250.9 to 264.3)	1.26 (1.20 to 1.32)
Ventricular arrhythmia	Antipsychotics user	22	11,186	19.7 (13.0 to 29.9)	18	20,863	8.6 (5.4 to 13.7)	2.28 (1.17 to 4.51)
	Matched comparators	162	81,924	19.8 (17.0 to 23.1)	259	252,489	10.3 (9.1 to 11.6)	1.93 (1.57 to 2.35)
Fractures	Antipsychotics user	459	8392	547.0 (499.1 to 599.3)	1110	11,727	946.5 (892.5 to 1003.9)	0.58 (0.52 to 0.64)
	Matched comparators	2582	67,419	383.0 (368.5 to 398.0)	8393	122,758	683.7 (669.2 to 698.5)	0.56 (0.54 to 0.59)
Pneumonia	Antipsychotics user	1771	10,105	1752.6 (1672.8 to 1836.2)	2013	19,444	1035.3 (991.0 to 1081.5)	1.69 (1.59 to 1.81)
	Matched comparators	5548	72,729	762.8 (743.0 to 783.2)	12,123	232,502	521.4 (512.2 to 530.8)	1.46 (1.42 to 1.51)
AKI [e]	Antipsychotics user	640	10,536	607.4 (562.2 to 656.4)	649	19,940	325.5 (301.4 to 351.5)	1.87 (1.67 to 2.08)
	Matched comparators	3039	75,449	402.8 (388.7 to 417.4)	6190	237,295	260.9 (254.4 to 267.4)	1.54 (1.48 to 1.61)
Unrelated outcome [f]	Antipsychotics user	25	10,660	23.5 (15.8 to 34.7)	28	19,428	14.4 (10.0 to 20.9)	1.63 (0.91 to 2.89)
	Matched comparators	167	78,807	21.2 (18.2 to 24.7)	365	231,027	15.8 (14.3 to 17.5)	1.34 (1.11 to 1.62)

[a] Matched comparators included males only.
[b] Matched comparators included females only.
[c] For antipsychotic users, the sum of sex-specific number of outcomes as well as the sum of sex-specific person-years differed from that reported in Table 2. This is because in the sex-specific analyses, some matched sets were dropped as there were no comparators of the same sex. Patients were also dropped if their propensity scores derived for the sex-specific analyses were not in the common support range. The same also applied to the matched comparators. In addition, by removing the comparators of a different sex to the antipsychotic users they were matched to, the number of comparators and therefore the numbers of outcomes and person years were greatly reduced compared with those reported in Table 2.
[d] VTE - Venous thromboembolism
[e] AKI - Acute kidney injury
[f] Unrelated outcome - Appendicitis and cholecystitis

Supplementary Table S14. Sex-specific hazard ratios (adjusted for IPT weights) of adverse outcomes associated with current, recent, and past antipsychotic use; with current use being defined as the first 90 days from the date of an antipsychotic prescription, recent use as up to 180 days after current use ended, and past use as after recent use.

	Antipsychotic use	Hazard ratios (HRs)		Males versus females HRs
		Males [a]	Females [b]	
Stroke	Current	1.36 (1.22 to 1.52)	1.80 (1.67 to 1.94)	0.76 (0.66 to 0.87)
	Recent	1.42 (1.13 to 1.79)	1.57 (1.31 to 1.89)	0.91 (0.67 to 1.22)
	Past	1.38 (1.03 to 1.87)	1.12 (0.90 to 1.41)	1.23 (0.84 to 1.79)
	Any exposure	1.37 (1.25 to 1.51)	1.69 (1.58 to 1.80)	0.81 (0.73 to 0.91)
VTE [c]	Current	1.68 (1.38 to 2.05)	1.66 (1.46 to 1.89)	1.01 (0.80 to 1.28)
	Recent	1.54 (0.99 to 2.39)	1.37 (1.00 to 1.87)	1.12 (0.66 to 1.93)
	Past	1.46 (0.88 to 2.43)	0.84 (0.53 to 1.31)	1.75 (0.89 to 3.44)
	Any exposure	1.63 (1.37 to 1.93)	1.52 (1.36 to 1.71)	1.07 (0.87 to 1.32)
Myocardial infarction	Current	1.36 (1.15 to 1.61)	1.25 (1.09 to 1.44)	1.09 (0.88 to 1.36)
	Recent	1.05 (0.71 to 1.56)	1.22 (0.89 to 1.67)	0.87 (0.52 to 1.43)
	Past	0.51 (0.29 to 0.90)	1.09 (0.78 to 1.51)	0.47 (0.24 to 0.91)
	Any exposure	1.19 (1.02 to 1.38)	1.22 (1.08 to 1.38)	0.97 (0.80 to 1.18)
Heart failure	Current	1.33 (1.18 to 1.50)	1.33 (1.21 to 1.46)	1.00 (0.86 to 1.17)
	Recent	0.66 (0.47 to 0.93)	0.97 (0.76 to 1.24)	0.68 (0.45 to 1.04)
	Past	0.91 (0.65 to 1.28)	0.84 (0.64 to 1.09)	1.09 (0.71 to 1.68)
	Any exposure	1.16 (1.05 to 1.30)	1.21 (1.12 to 1.32)	0.96 (0.84 to 1.10)
Ventricular arrhythmia	Current	1.15 (0.67 to 1.98)	0.68 (0.36 to 1.28)	1.69 (0.74 to 3.89)
	Recent	1.34 (0.32 to 5.73)	1.05 (0.30 to 3.69)	1.28 (0.19 to 8.68)
	Past	0.99 (0.16 to 6.03)	1.50 (0.51 to 4.38)	0.66 (0.08 to 5.39)
	Any exposure	1.15 (0.71 to 1.88)	0.83 (0.51 to 1.36)	1.38 (0.69 to 2.76)
Fractures	Current	1.51 (1.35 to 1.69)	1.40 (1.30 to 1.50)	1.08 (0.95 to 1.23)
	Recent	1.02 (0.78 to 1.33)	1.36 (1.16 to 1.61)	0.74 (0.54 to 1.02)
	Past	0.88 (0.63 to 1.22)	1.14 (0.94 to 1.38)	0.77 (0.53 to 1.13)
	Any exposure	1.34 (1.21 to 1.48)	1.36 (1.28 to 1.45)	0.98 (0.87 to 1.10)
Pneumonia	Current	2.36 (2.22 to 2.51)	2.05 (1.94 to 2.16)	1.15 (1.06 to 1.25)
	Recent	1.89 (1.63 to 2.18)	1.51 (1.31 to 1.73)	1.25 (1.03 to 1.53)
	Past	1.56 (1.32 to 1.86)	1.35 (1.16 to 1.57)	1.16 (0.92 to 1.45)
	Any exposure	2.19 (2.08 to 2.31)	1.88 (1.80 to 1.98)	1.16 (1.08 to 1.25)

AKI [d]	Current	2.00 (1.82 to 2.20)	1.52 (1.39 to 1.67)	1.32 (1.15 to 1.50)
	Recent	1.14 (0.88 to 1.48)	1.13 (0.89 to 1.43)	1.01 (0.71 to 1.44)
	Past	1.01 (0.77 to 1.33)	1.16 (0.92 to 1.46)	0.88 (0.61 to 1.26)
	Any exposure	1.73 (1.59 to 1.88)	1.42 (1.31 to 1.53)	1.22 (1.08 to 1.37)
Unrelated outcome [e]	Current	0.92 (0.58 to 1.46)	0.79 (0.47 to 1.33)	1.17 (0.58 to 2.35)
	Recent	1.13 (0.39 to 3.29)	1.26 (0.52 to 3.04)	0.90 (0.23 to 3.57)
	Past	1.05 (0.29 to 3.84)	3.35 (1.47 to 7.61)	0.31 (0.07 to 1.46)
	Any exposure	0.96 (0.64 to 1.45)	1.08 (0.73 to 1.59)	0.89 (0.51 to 1.57)

[a] Matched comparators included males only.
[b] Matched comparators included females only.
[c] VTE - Venous thromboembolism
[d] AKI - Acute kidney injury
[e] Unrelated outcome - Appendicitis and cholecystitis
